# Supplementary material for: A combined experimental and computational study of ligand-controlled Chan-Lam coupling of sulfenamides
Source: Nat Commun. 2024 Jun 4;15:4747. doi: 10.1038/s41467-024-49089-0 (PMC11150460; doi:10.1038/s41467-024-49089-0)
Supplement: Supplementary file 1 — Supplementary Information [file 41467_2024_49089_MOESM1_ESM.pdf]

Supplementary Information

A Combined Experimental and Computational Study of Ligand-Controlled Chan-Lam Coupling of Sulfenamides

Kaiming Han,[1,2,5] Hong Liu,[1,5] Madeline E. Rotella,[3,5] Zeyu Xu,[1] Lizhi Tao,[1] Shufeng Chen,*[2] Marisa C. Kozlowski*[3] and Tiezheng Jia*[1,4]

[1]Research Center for Chemical Biology and Omics Analysis, Department of Chemistry, Southern University of Science and Technology, 1088 Xueyuan Blvd., Shenzhen, Guangdong, 518055, P. R. China

[2]Inner Mongolia Key Laboratory of Fine Organic Synthesis, College of Chemical Engineering, Inner Mongolia University, 010021, P. R. China

[3]Roy and Diana Vagelos Laboratories, Department of Chemistry, University of Pennsylvania, 231 South 34th Street, Philadelphia, Pennsylvania, 19104, United States

[4]State Key Laboratory of Elemento-Organic Chemistry, Nankai University, Tianjin, 300071, P. R. China

[5]These authors contributed equally: Kaiming Han, Hong Liu, and Madeline E. Rotella

E-mail: jiatz@sustech.edu.cn; marisa@sas.upenn.edu; shufengchen@imu.edu.cn

Table of Contents

1. Supplementary Notes S2

2.Supplementary Methods S2

2.1 Preparation of Sulfenamides S2

2.2 Optimization of Copper-Catalyzed Chan-Lam Coupling of Sulfenamides S2

2.3 Procedure and Characterization S5

2.4 X-ray Structure of **3bn** (CCDC 2142998) S22

2.5 Synthetic Applications S27

3.Supplementary Discussion S31

3.1 Mechanistic Studies S31

4. Supplementary Figures S66

4.1 NMR Spectra S66

5. Supplementary References S137

1. Supplementary Notes

All reactions were carried out under a dry O_2 atmosphere unless otherwise stated. Acetonitrile (MeCN), dichloromethane (DCM), 1,2-dichloroethane (DCE), tetrahydrofuran (THF), isopropyl alcohol (IPA), tertiary butyl alcohol (^{t}BuOH), toluene, acetone and 1,4-dioxane were purchased from J&K Chemicals. Unless otherwise stated, reagents were commercially available and used as purchased without further purification. Chemicals were purchased from J&K Chemicals, Adamas-beta, Macklin Reagent, Energy Chemicals, Aladdin, JiuDing Chemicals, or Bide Pharmatech Ltd. The progress of the reactions was monitored by thin-layer chromatography using Whatman Partisil K6F 250 μm precoated 60 Å silica gel plates and visualized by short-wave ultraviolet light as well as by treatment with iodine. Flash chromatography was performed with silica gel (200–300 mesh). The NMR spectra were obtained using a Brüker 400 or 600 MHz Fourier-transform NMR spectrometer. Chemical shifts are reported in units of parts per million (ppm) downfield from tetramethylsilane (TMS), and all coupling constants are reported in hertz. The infrared spectra were taken with KBr plates with a Perkin-Elmer Spectrum Vertex 80 Series spectrometer. High resolution mass spectrometry (HRMS) data were obtained on a Bruker Apex IV RTMS using electrospray ionization (ESI) in positive mode. Melting points were determined on a Mel-Temp melting point apparatus and were uncorrected. X-ray data were collected on a Bruker APEX-II CCD diffractometer area detector.

2.Supplementary Methods

2.1 Preparation of Sulfenamides

Sulfenamides were prepared according to the literature procedures.[1-3]

2.2 Optimization of Copper-Catalyzed Chan-Lam Coupling of Sulfenamides

Supplementary Table 1. Optimization of Catalyst[a]

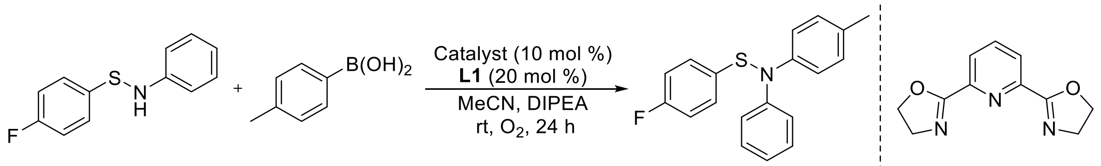

1a 2a 3aa L1

entry	catalyst	assay yield[b]/%
1	$Cu(OAc)_2$•H_2O	12
2	Cu_2O	42
3	CuF_2	39
4	CuI	15
5	$Cu(MeCN)_4BF_4$	50
6	$Cu(TFA)_2$•H_2O	65

[a]Reaction conditions: **1a** (0.1 mmol), **2a** (2.0 equiv), catalyst (10 mol %), **L1** (20 mol %), DIPEA (1.5 equiv) and MeCN (0.5 mL) at room temperature for 24 h under an O_2 atmosphere. [b]Assay yield determined by ^{19}F NMR analysis of the crude reaction mixtures using 0.1 mmol $PhCF_3$ (12.0 μL) as internal standard.

Supplementary Table 2. Optimization of Solvent[a]

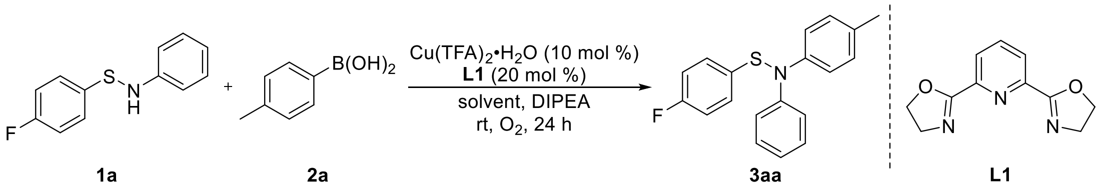

entry	solvent	assay yield[b]/%
1	DCM	22
2	DCE	17
3	THF	22
4	1,4-dioxane	36
5	toluene	36
6	IPA	46
7	^{t}BuOH	24
8	acetone	40

[a]Reaction conditions: **1a** (0.15 mmol), **2a** (2.0 equiv), $Cu(TFA)_2$•H_2O (10 mol %), **L1** (20 mol %), DIPEA (1.5 equiv) and solvent (0.5 mL) at room temperature for 24 h under an O_2 atmosphere. [b]Assay yield determined by ^{19}F NMR analysis of the crude reaction mixtures using 0.1 mmol $PhCF_3$ (12.0 µL) as internal standard.

Supplementary Table 3. Optimization of Base[a]

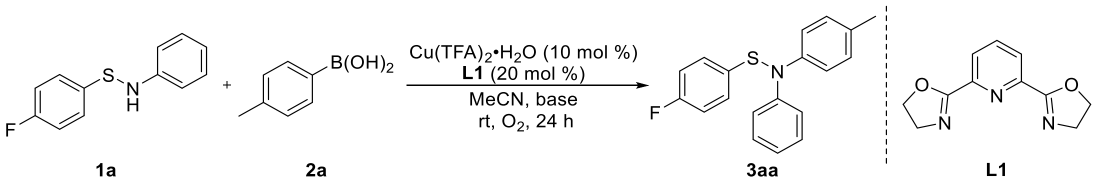

entry	base	assay yield[b]/%
1	Cy_2NMe	72
2	N-Methylpiperidine	48
3	2,6-lutidine	16
4	2-(dimethylamino)ethan-1-ol	10
5	4-Methylmorpholine	10
6	TEA	34

[a]Reaction conditions: **1a** (0.15 mmol), **2a** (2.0 equiv), $Cu(TFA)_2$•H_2O (10 mol %), **L1** (20 mol %), base (1.5 equiv) and MeCN (0.5 mL) at room temperature for 24 h under an O_2 atmosphere. [b]Assay yield determined by ^{19}F NMR analysis of the crude reaction mixtures using 0.1 mmol $PhCF_3$ (12.0 µL) as internal standard.

Supplementary Table 4. Optimization of Ligand[a]

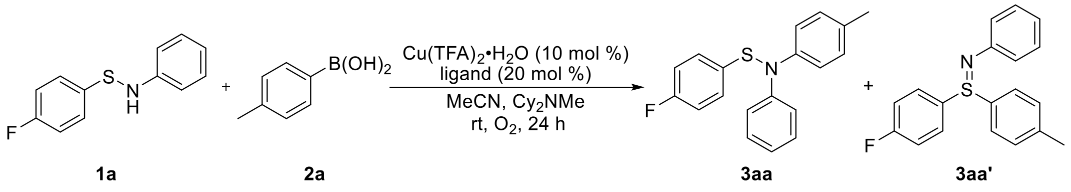

entry	ligand	**3aa** assay yield[b]/%	**3aa’** assay yield[b]/%
1	**L1**	72	7
2	**L2**	56	11
3	**L3**	87(84[c])	5
4	**L4**	26	0
5	**L5**	65	3
6	**L6**	0	0

[a]Reaction conditions: **1a** (0.15 mmol), **2a** (2.0 equiv), $Cu(TFA)_2{\bullet}H_2O$ (10 mol %), ligand (20 mol %), Cy_2NMe (1.5 equiv) and MeCN (0.5 mL) at room temperature for 24 h under an O_2 atmosphere. [b]Assay yield determined by ^{19}F NMR analysis of the crude reaction mixtures using 0.1 mmol $PhCF_3$ (12.0 μL) as internal standard. [c]Isolated yield.

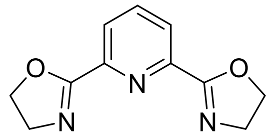

L1

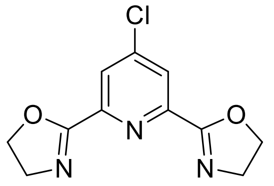

L2

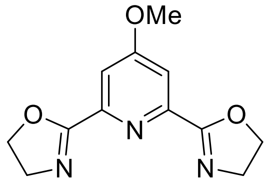

L3

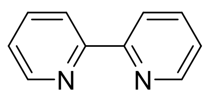

L4

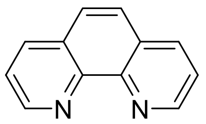

L5

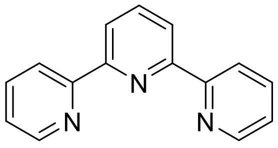

L6

Supplementary Table 5. Deviation of Standard Conditions[a]

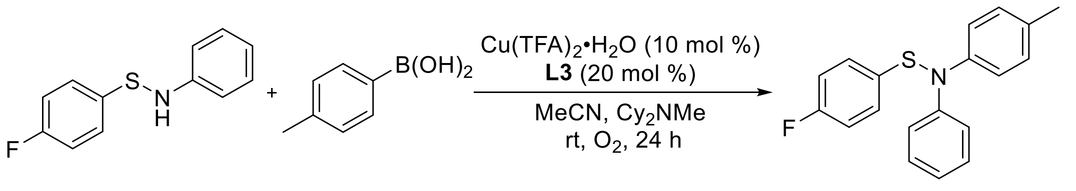

1a **2a** **3aa**

entry	deviation of standard conditions	assay yield[b]/%
1	none	87(84[c])
2	with 5 mol % $Cu(TFA)_2{\bullet}H_2O$	78
3	without $Cu(TFA)_2{\bullet}H_2O$	0
4	with 1.0 equiv Cy_2NMe	71
5	without Cy_2NMe	0
6	without **L3**	0
7	under Ar atmosphere instead of O_2	10
8	under air atmosphere instead of O_2	37

[a]Reaction conditions: **1a** (0.15 mmol), **2a** (2.0 equiv), $Cu(TFA)_2{\bullet}H_2O$ (10 mol %), **L3** (20 mol %), Cy_2NMe (1.5 equiv) and MeCN (0.5 mL) at room temperature for 24 h

under an O_2 atmosphere. [b]Assay yield determined by ^{19}F NMR analysis of the crude reaction mixtures using 0.1 mmol $PhCF_3$ (12.0 μL) as internal standard. [c]Isolated yield.

2.3 Procedure and Characterization

General Procedure for Catalysis: To an oven-dried microwave vial equipped with a stir bar was added $Cu(TFA)_2•H_2O$ (4.5 mg, 10 mol %), **L3** (7.4 mg, 20 mol %) *S*-(4-fluorophenyl)-*N*-(*p*-tolyl)thiohydroxylamine (**1a**) (35.0 mg, 0.15 mmol) and *p*-tolylboronic acid (**2a**) (40.8 mg, 0.3 mmol). Then, Cy_2NMe (48.2 μL, 0.23 mmol) and MeCN (0.5 mL) was added via syringe. The vial was sealed with a septum, and refilled by a O_2 balloon for 3 min. The solution was then stirred at room temperature for 24 h under an O_2 atmosphere. Upon completion of the reaction, the solvent was removed under vacuum to give a residue, which was further purified by flash chromatography, as oulined below, to give the pure product.

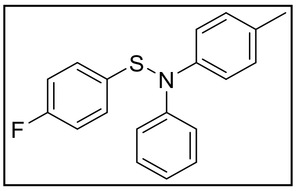

***S*-(4-Fluorophenyl)-*N*-Phenyl-*N*-(*p*-tolyl)thiohydroxylamine (3aa):** The reaction was performed following the General Procedure with *S*-(4-fluorophenyl)-*N*-(*p*-tolyl)thiohydroxylamine (35.0 mg, 0.15 mmol), **2a** (40.8 mg, 0.3 mmol), Cy_2NMe (48.2 μL, 0.23 mmol), $Cu(TFA)_2•H_2O$ (4.5 mg, 10 mol %) and **L3** (7.4 mg, 20 mol %). The crude product was purified by flash chromatography on silica gel (eluted with hexane) to give the product **3aa** (38.5 mg, 83% yield) as a colorless oil. R_f = 0.5 (hexane); ^{1}H NMR (400 MHz, $CDCl_3$) δ 7.27 – 7.20 (m, 6H), 7.17 (d, J = 8.5 Hz, 2H), 7.09 (d, J = 8.2 Hz, 2H), 6.98 (t, J = 8.8 Hz, 3H), 2.31 (s, 3H) ppm; ^{13}C NMR (100 MHz, $CDCl_3$) δ 161.6 (d, $J_{C\text{-}F}$ = 245.0 Hz), 149.2, 146.1, 136.1 (d, $J_{C\text{-}F}$ = 3.1 Hz), 133.9, 130.0, 129.2, 125.4 (d, $J_{C\text{-}F}$ = 7.9 Hz), 123.3, 122.6, 120.6, 116.2 (d, $J_{C\text{-}F}$ = 22.1 Hz), 20.9 ppm; ^{19}F NMR (376 MHz, $CDCl_3$) δ -116.5 ppm; IR (thin film): 2974, 2918, 1591, 1550, 1261, 1225, 1080, 1045, 812, 699 cm^{-1}; HRMS calculated for $C_{19}H_{17}FNS^+$ 310.1060, found 310.1064 $[M+H]^+$.

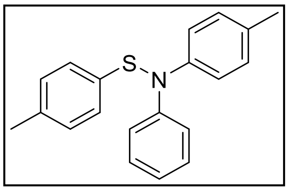

***N*-Phenyl-*N*,*S*-di-*p*-tolylthiohydroxylamine (3ba):** The reaction was performed following the General Procedure with **1b** (32.3 mg, 0.15 mmol), *p*-tolylboronic acid (40.8 mg, 0.3 mmol), Cy_2NMe (48.2 μL, 0.23 mmol), $Cu(TFA)_2•H_2O$ (4.5 mg, 10 mol %) and **L3** (7.4 mg, 20 mol %). The crude product was purified by flash chromatography on silica gel (eluted with hexane) to give the product **3ba** (36.6 mg, 80% yield) as a colorless oil. R_f = 0.5 (hexane); ^{1}H NMR (400 MHz, $CDCl_3$) δ 7.33 – 7.19 (m, 8H), 7.17 – 7.10 (m, 4H), 7.04 – 6.99 (m, 1H), 2.35 (s, 3H), 2.33 (s, 3H) ppm; ^{13}C NMR (100 MHz, $CDCl_3$) δ 149.4, 146.3, 137.6, 135.7, 133.6, 129.9, 129.8, 129.1, 123.4, 123.3, 122.3, 120.7, 21.1, 20.9 ppm; IR (thin film): 2975, 2918, 1593, 1488, 1452, 1261, 1081, 907, 801, 692 cm^{-1}; HRMS calculated for $C_{20}H_{20}NS^+$ 306.1311, found 306.1315 $[M+H]^+$.

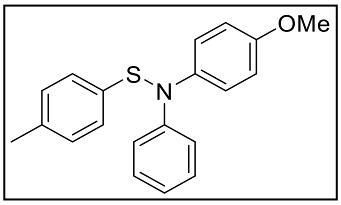

***N*-(4-Methoxyphenyl)-*N*-phenyl-*S*-(*p*-tolyl)thiohydroxylamine (3bb):** The reaction was performed following the General Procedure with **1b** (32.3 mg, 0.15 mmol), (4-methoxyphenyl)boronic acid (45.6 mg, 0.3 mmol), Cy_2NMe (48.2 μL, 0.23 mmol), $Cu(TFA)_2$•H_2O (4.5 mg, 10 mol %) and **L3** (7.4 mg, 20 mol %). The crude product was purified by flash chromatography on silica gel (eluted with EtOAc:hexane = 1:200) to give the product **3bb** (41.9 mg, 87% yield) as a red solid. R_f = 0.3 (EtOAc:hexane = 1:200); m.p. = 54.5–55.1 °C; ^{1}H NMR (400 MHz, $CDCl_3$) δ 7.26 – 7.14 (m, 8H), 7.09 (d, J = 8.1 Hz, 2H), 6.95 – 6.88 (m, 1H), 6.87 – 6.80 (m, 2H), 3.78 (s, 3H), 2.29 (s, 3H) ppm; ^{13}C NMR (100 MHz, $CDCl_3$) δ 157.0, 149.9, 141.8, 137.5, 135.9, 129.8, 129.1, 126.3, 123.6, 121.3, 118.8, 114.7, 55.6, 21.1 ppm; IR (thin film): 3020, 2920, 2361, 1590, 1487, 1239, 1031, 914, 725, 693 cm^{-1}; HRMS calculated for $C_{20}H_{20}NOS^+$ 322.1260, found 322.1266 $[M+H]^+$.

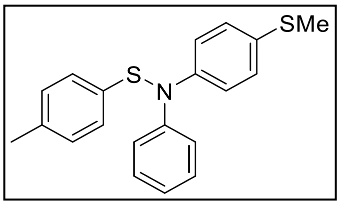

***N*-(4-(Methylthio)phenyl)-*N*-phenyl-*S*-(*p*-tolyl)thiohydroxylamine (3bc):** The reaction was performed following the General Procedure with **1b** (32.3 mg, 0.15 mmol), (4-(methylthio)phenyl)boronic acid (50.4 mg, 0.3 mmol), Cy_2NMe (48.2 μL, 0.23 mmol), $Cu(TFA)_2$•H_2O (4.5 mg, 10 mol %) and **L3** (7.4 mg, 20 mol %). The crude product was purified by flash chromatography on silica gel (eluted with EtOAc:hexane = 1:200) to give the product **3bc** (28.8 mg, 57% yield) as a colorless oil. R_f = 0.5 (EtOAc:hexane = 1:200); ^{1}H NMR (400 MHz, $CDCl_3$) δ 7.33 – 7.15 (m, 10H), 7.12 (d, J = 8.1 Hz, 2H), 7.07 – 7.02 (m, 1H), 2.48 (s, 3H), 2.32 (s, 3H) ppm; ^{13}C NMR (100 MHz, $CDCl_3$) δ 148.8, 146.7, 137.1, 135.9, 132.3, 129.8, 129.2, 128.4, 123.4, 123.0, 122.9, 121.6, 21.0, 16.9 ppm; IR (thin film): 2974, 2918, 1583, 1264, 1177, 1083, 966, 802, 693 cm^{-1}; HRMS calculated for $C_{20}H_{20}NS_2^+$ 338.1032, found 338.1038 $[M+H]^+$.

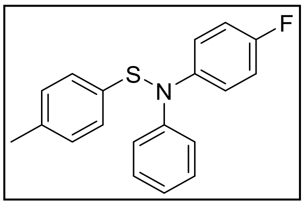

***N*-(4-Fluorophenyl)-*N*-phenyl-*S*-(*p*-tolyl)thiohydroxylamine (3bd):** The reaction was performed following the General Procedure with **1b** (32.3 mg, 0.15 mmol), (4-fluorophenyl)boronic acid (42.0 mg, 0.3 mmol), Cy_2NMe (48.2 μL, 0.23 mmol), $Cu(TFA)_2$•H_2O (4.5 mg, 10 mol %) and **L3** (7.4 mg, 20 mol %). The crude product was purified by flash chromatography on silica gel (eluted with hexane) to give the product **3bd** (37.5 mg, 81% yield) as a colorless oil. R_f = 0.5 (hexane); ^{1}H NMR (400 MHz, $CDCl_3$) δ 7.27 – 7.22 (m, 6H), 7.15 (d, J = 8.3 Hz, 2H), 7.10 (d, J = 8.1 Hz, 2H), 7.01 – 6.92 (m, 3H), 2.29 (s, 3H) ppm; ^{13}C NMR (100 MHz, $CDCl_3$) δ 159.5 (d, J_{C-F} = 243.4 Hz), 149.3, 145.0 (d, J_{C-F} = 2.8 Hz), 137.1, 136.2, 129.9, 129.3, 125.1 (d, J_{C-F} = 8.2 Hz), 123.7, 122.6, 120.5, 116.0 (d, J_{C-F} = 22.6 Hz), 21.1 ppm; ^{19}F NMR (376 MHz, $CDCl_3$) δ -119.0 ppm; IR (thin film): 2972, 1593, 1499, 1488, 1280, 1193, 1081, 949, 800, 691 cm^{-1}; HRMS calculated for $C_{19}H_{17}FNS^+$ 310.1060, found

310.1063 [M+H]$^+$.

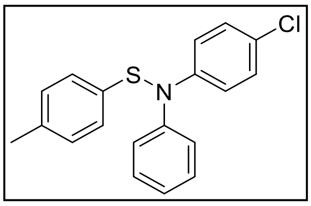

***N*-(4-Chlorophenyl)-*N*-phenyl-*S*-(*p*-tolyl)thiohydroxylamine (3be):** The reaction was performed following the General Procedure with **1b** (32.3 mg, 0.15 mmol), (4-chlorophenyl)boronic acid (46.8 mg, 0.3 mmol), Cy_2NMe (48.2 µL, 0.23 mmol), $Cu(TFA)_2•H_2O$ (4.5 mg, 10 mol %) and **L3** (7.4 mg, 20 mol %). The crude product was purified by flash chromatography on silica gel (eluted with hexane) to give the product **3be** (36.6 mg, 75% yield) as a colorless oil. R_f = 0.5 (hexane); ^{1}H NMR (400 MHz, $CDCl_3$) δ 7.30 – 7.25 (m, 4H), 7.24 – 7.17 (m, 4H), 7.14 (d, J = 8.4 Hz, 2H), 7.10 (d, J = 8.8 Hz, 2H), 7.07 – 7.03 (m, 1H), 2.30 (s, 3H) ppm; ^{13}C NMR (100 MHz, $CDCl_3$) δ 148.6, 147.8, 136.9, 136.3, 130.0, 129.4, 129.2, 128.0, 123.8, 123.7, 123.0, 122.5, 21.1 ppm; IR (thin film): 2982, 2351, 1599, 1485, 1274, 1259, 1182, 947, 798, 697 cm^{-1}; HRMS calculated for $C_{19}H_{17}ClNS^+$ 326.0765, found 326.0767 [M+H]$^+$.

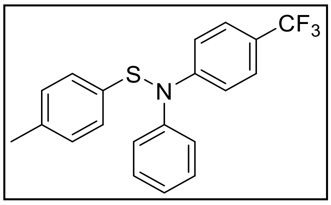

***N*-Phenyl-*S*-(*p*-tolyl)-*N*-(4-(trifluoromethyl)phenyl)thiohydroxylamine (3bf):** The reaction was performed following the General Procedure with **1b** (32.3 mg, 0.15 mmol), (4-(trifluoromethyl)phenyl)boronic acid (57.0 mg, 0.3 mmol), Cy_2NMe (48.2 µL, 0.23 mmol), $Cu(TFA)_2•H_2O$ (4.5 mg, 10 mol %) and **L3** (7.4 mg, 20 mol %). The crude product was purified by flash chromatography on silica gel (eluted with hexane) to give the product **3bf** (39.8 mg, 74% yield) as a colorless oil. R_f = 0.7 (hexane); ^{1}H NMR (400 MHz, $CDCl_3$) δ 7.47 (d, J = 8.8 Hz, 2H), 7.36 – 7.30 (m, 6H), 7.22 – 7.09 (m, 5H), 2.32 (s, 3H) ppm; ^{13}C NMR (100 MHz, $CDCl_3$) δ 152.3, 147.8, 136.6, 136.2, 130.1, 129.7, 126.4 (q, $J_{C\text{-}F}$ = 3.8 Hz), 125.6, 125.0, 124.6 (q, $J_{C\text{-}F}$ = 268.8 Hz), 124.1, 123.4 (q, $J_{C\text{-}F}$ = 32.6 Hz), 118.9, 21.2 ppm; ^{19}F NMR (376 MHz, $CDCl_3$) δ -61.6 ppm; IR (thin film): 3527, 2990, 1612, 1489, 1223, 1113, 1068, 907, 754, 669 cm^{-1}; HRMS calculated for $C_{20}H_{17}F_3NS^+$ 360.1028, found 360.1031 [M+H]$^+$.

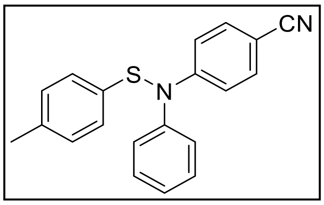

4-(Phenyl(*p*-tolylthio)amino)benzonitrile (3bg): The reaction was performed following the General Procedure with **1b** (32.3 mg, 0.15 mmol), (4-cyanophenyl)boronic acid (44.1 mg, 0.3 mmol), Cy_2NMe (48.2 µL, 0.23 mmol), $Cu(TFA)_2•H_2O$ (4.5 mg, 10 mol %) and **L3** (7.4 mg, 20 mol %). The crude product was purified by flash chromatography on silica gel (eluted with EtOAc:hexane = 1:20) to give the product **3bg** (27.0 mg, 57% yield) as a brown oil. R_f = 0.3 (hexane); ^{1}H NMR (400 MHz, $CDCl_3$) δ 7.50 – 7.44 (m, 2H), 7.39 – 7.33 (m, 2H), 7.31 – 7.20 (m, 5H), 7.17 – 7.09 (m, 4H), 2.31 (s, 3H) ppm; ^{13}C NMR (100 MHz, $CDCl_3$) δ 153.4, 147.1, 137.2, 135.2, 133.4, 130.1, 130.0, 126.6, 126.1, 124.7, 119.6, 118.1, 103.5, 21.2 ppm; IR (thin

film): 2970, 2922, 2245, 1593, 1494, 1265, 1197, 914, 798, 691 cm^{-1}; HRMS calculated for $C_{20}H_{17}N_2S^+$ 317.1107, found 317.1111 $[M+H]^+$.

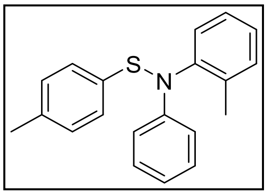

***N*-Phenyl-*N*-(*o*-tolyl)-*S*-(*p*-tolyl)thiohydroxylamine (3bh):** The reaction was performed following the General Procedure with **1b** (32.3 mg, 0.15 mmol), *o*-tolylboronic acid (40.8 mg, 0.3 mmol), Cy_2NMe (48.2 μL, 0.23 mmol), $Cu(TFA)_2•H_2O$ (4.5 mg, 10 mol %) and **L3** (7.4 mg, 20 mol %). The crude product was purified by flash chromatography on silica gel (eluted with hexane) to give the product **3bh** (36.6 mg, 80% yield) as a colorless oil. R_f = 0.5 (hexane); ^{1}H NMR (400 MHz, $CDCl_3$) δ 7.31 – 7.14 (m, 8H), 7.10 (d, *J* = 7.9 Hz, 2H), 7.06 – 7.00 (m, 2H), 6.89 – 6.82 (m, 1H), 2.31 (s, 3H), 2.14 (s, 3H) ppm; ^{13}C NMR (100 MHz, $CDCl_3$) δ 149.7, 146.3, 136.9, 136.5, 136.3, 131.7, 129.8, 129.1, 128.3, 127.5, 127.3, 125.4, 120.0, 115.7, 21.2, 18.3 ppm; IR (thin film): 3527, 2990, 1612, 1489, 1323, 1266, 1163, 1009, 831, 754 cm^{-1}; HRMS calculated for $C_{20}H_{20}NS^+$ 306.1311, found 306.1317 $[M+H]^+$.

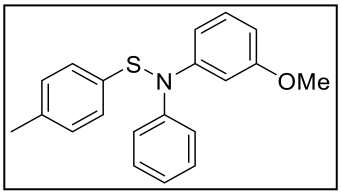

***N*-(3-Methoxyphenyl)-*N*-phenyl-*S*-(*p*-tolyl)thiohydroxylamine (3bi):** The reaction was performed following the General Procedure with **1b** (32.3 mg, 0.15 mmol), (3-methoxyphenyl)boronic acid (45.6 mg, 0.3 mmol), Cy_2NMe (48.2 μL, 0.23 mmol), $Cu(TFA)_2•H_2O$ (4.5 mg, 10 mol %) and **L3** (7.4 mg, 20 mol %). The crude product was purified by flash chromatography on silica gel (eluted with EtOAc:hexane = 1:200) to give the product **3bi** (41.4 mg, 86% yield) as a colorless oil. R_f = 0.5 (EtOAc:hexane = 1:200); ^{1}H NMR (400 MHz, $CDCl_3$) δ 7.33 – 7.22 (m, 4H), 7.19 – 7.11 (m, 3H), 7.08 (d, *J* = 8.1 Hz, 2H), 7.03 (t, *J* = 7.0 Hz, 1H), 6.86 (dd, *J* = 5.1, 2.6 Hz, 2H), 6.57 (dd, *J* = 9.1, 1.5 Hz, 1H), 3.72 (s, 3H), 2.28 (s, 3H) ppm; ^{13}C NMR (100 MHz, $CDCl_3$) δ 160.5, 150.4, 148.9, 137.3, 136.0, 129.9, 129.3, 123.7, 123.6, 122.8, 114.1, 108.3, 107.7, 55.4, 21.1 ppm; IR (thin film): 2920, 2359, 1585, 1485, 1284, 1232, 1046, 972, 799, 690 cm^{-1}; HRMS calculated for $C_{20}H_{20}NOS^+$ 322.1260, found 322.1263 $[M+H]^+$.

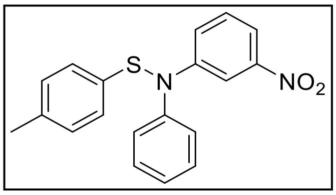

***N*-(3-Nitrophenyl)-*N*-phenyl-*S*-(*p*-tolyl)thiohydroxylamine (3bj):** The reaction was performed following the General Procedure with **1b** (32.3 mg, 0.15 mmol), (3-nitrophenyl)boronic acid (50.1 mg, 0.3 mmol), Cy_2NMe (48.2 μL, 0.23 mmol), $Cu(TFA)_2•H_2O$ (4.5 mg, 10 mol %) and **L3** (7.4 mg, 20 mol %). The crude product was purified by flash chromatography on silica gel (eluted with EtOAc:hexane = 1:50) to give the product **3bj** (36.3 mg, 72% yield) as a yellow oil. R_f = 0.2 (EtOAc:hexane = 1:50); ^{1}H NMR (400 MHz, $CDCl_3$) δ 8.12 (t, *J* = 2.3 Hz, 1H), 7.80 – 7.75 (m, 1H), 7.59 – 7.53 (m, 1H), 7.39 – 7.28 (m, 5H), 7.22 – 7.15 (m, 3H), 7.13 (d, *J* = 8.2 Hz, 2H), 2.32 (s, 3H) ppm; ^{13}C NMR (100 MHz, $CDCl_3$) δ 150.8, 149.2, 147.6, 137.1, 135.7, 130.1,

129.9, 129.8, 125.8, 125.1, 124.7, 124.6, 116.4, 114.1, 21.2 ppm; IR (thin film): 2965, 1525, 1488, 1345, 1263, 1198, 1078, 966, 844, 693 cm^{-1}; HRMS calculated for $C_{19}H_{17}N_2O_2S^+$ 337.1005, found 337.1006 $[M+H]^+$.

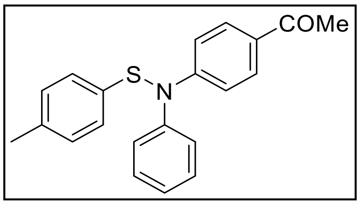

1-(4-(Phenyl(*p*-tolylthio)amino)phenyl)ethan-1-one (3bk): The reaction was performed following the General Procedure with **1b** (32.3 mg, 0.15 mmol), (4-acetylphenyl)boronic acid (49.2 mg, 0.3 mmol), Cy_2NMe (48.2 μL, 0.23 mmol), $Cu(TFA)_2•H_2O$ (4.5 mg, 10 mol %) and **L3** (7.4 mg, 20 mol %). The crude product was purified by flash chromatography on silica gel (eluted with EtOAc:hexane = 1:20) to give the product **3bk** (27.0 mg, 54% yield) as a colorless oil. R_f = 0.2 (hexane); ^1H NMR (400 MHz, $CDCl_3$) δ 7.89 – 7.79 (m, 2H), 7.38 – 7.24 (m, 6H), 7.24 – 7.06 (m, 5H), 2.53 (s, 3H), 2.30 (s, 3H) ppm; ^{13}C NMR (100 MHz, $CDCl_3$) δ 196.7, 153.7, 147.6, 136.8, 136.0, 130.5, 130.0, 129.8, 126.0, 125.6, 124.4, 117.9, 26.5, 21.2 ppm; IR (thin film): 2974, 2918, 1541, 1489, 1308, 1264, 1177, 1083, 1015, 747 cm^{-1}; HRMS calculated for $C_{21}H_{20}NOS^+$ 334.1260, found 334.1263 $[M+H]^+$.

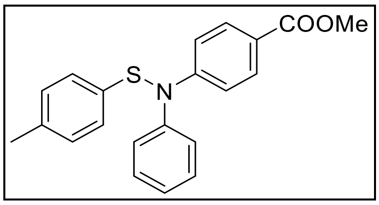

Methyl 4-(phenyl(*p*-tolylthio)amino)benzoate (3bl): The reaction was performed following the General Procedure with **1b** (32.3 mg, 0.15 mmol), (4-(methoxycarbonyl)phenyl)boronic acid (54.0 mg, 0.3 mmol), Cy_2NMe (48.2 μL, 0.23 mmol), $Cu(TFA)_2•H_2O$ (4.5 mg, 10 mol %) and **L3** (7.4 mg, 20 mol %). The crude product was purified by flash chromatography on silica gel (eluted with EtOAc:hexane = 1:50) to give the product **3bl** (37.7 mg, 72% yield) as a colorless oil. R_f = 0.3 (EtOAc:hexane = 1:50); ^1H NMR (400 MHz, $CDCl_3$) δ 7.93 – 7.85 (m, 2H), 7.36 – 7.23 (m, 6H), 7.20 – 7.07 (m, 5H), 3.86 (s, 3H), 2.29 (s, 3H) ppm; ^{13}C NMR (100 MHz, $CDCl_3$) δ 166.9, 153.5, 147.8, 136.6, 136.1, 131.0, 130.0, 129.7, 125.8, 125.4, 124.2, 122.9, 118.1, 51.9, 21.1 ppm; IR (thin film): 3359, 2974, 1583, 1489, 1308, 1264, 1015, 966, 802, 693 cm^{-1}; HRMS calculated for $C_{21}H_{20}NO_2S^+$ 350.1209, found 350.1211 $[M+H]^+$.

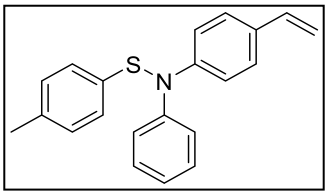

***N*-Phenyl-*S*-(*p*-tolyl)-*N*-(4-vinylphenyl)thiohydroxylamine (3bm):** The reaction was performed following the General Procedure with **1b** (32.3 mg, 0.15 mmol), (4-vinylphenyl)boronic acid (44.4 mg, 0.3 mmol), Cy_2NMe (48.2 μL, 0.23 mmol), $Cu(TFA)_2•H_2O$ (4.5 mg, 10 mol %) and **L3** (7.4 mg, 20 mol %). The crude product was purified by flash chromatography on silica gel (eluted with hexane) to give the product **3bm** (32.3 mg, 68% yield) as a colorless oil. R_f = 0.5 (hexane); ^1H NMR (400 MHz, $CDCl_3$) δ 7.33 – 7.22 (m, 8H), 7.17 – 7.13 (m, 2H), 7.09 (d, J = 8.0 Hz, 2H), 7.06 – 7.01 (m, 1H), 6.65 (dd, J = 17.6, 10.9 Hz, 1H), 5.63 (dd, J = 17.6, 0.9 Hz, 1H), 5.15 (dd, J =

10.9, 0.9 Hz, 1H), 2.29 (s, 3H) ppm; ^{13}C NMR (100 MHz, $CDCl_3$) δ 148.8, 148.7, 137.2, 136.2, 136.0, 132.6, 129.9, 129.3, 127.1, 123.6, 123.5, 122.5, 121.7, 112.6, 21.1 ppm; IR (thin film): 2974, 1592, 1263, 1201, 905, 802, 728, 648 cm^{-1}; HRMS calculated for $C_{21}H_{20}NS^+$ 318.1311, found 318.1313 $[M+H]^+$.

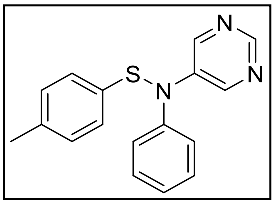

***N*-Phenyl-*N*-(pyrimidin-5-yl)-*S*-(*p*-tolyl)thiohydroxylamine (3bn):** The reaction was performed following the General Procedure with **1b** (32.3 mg, 0.15 mmol), pyrimidin-5-ylboronic acid (37.2 mg, 0.3 mmol), Cy_2NMe (48.2 μL, 0.23 mmol), $Cu(TFA)_2•H_2O$ (4.5 mg, 10 mol %) and **L3** (7.4 mg, 20 mol %). The crude product was purified by flash chromatography on silica gel (eluted with EtOAc:hexane = 1:10) to give the product **3bn** (31.2 mg, 71% yield) as a white solid. R_f = 0.2 (EtOAc:hexane = 1:10); m.p. = 97.0–98.2 °C; ^1H NMR (400 MHz, $CDCl_3$) δ 8.81 (s, 1H), 8.68 (s, 2H), 7.39 – 7.27 (m, 4H), 7.22 – 7.15 (m, 3H), 7.13 (d, *J* = 8.2 Hz, 2H), 2.31 (s, 3H) ppm; ^{13}C NMR (100 MHz, $CDCl_3$) δ 152.0, 147.9, 146.8, 143.8, 137.6, 134.9, 130.2(2), 129.9(9), 125.7, 125.2, 123.8, 21.2 ppm; IR (thin film): 3750, 3024, 2341, 1588, 1554, 1490, 1208, 924, 801, 650 cm^{-1}; HRMS calculated for $C_{17}H_{16}N_3S^+$ 294.1059, found 294.1065 $[M+H]^+$.

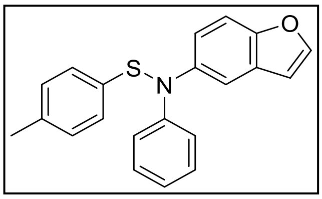

***N*-(Benzofuran-5-yl)-*N*-phenyl-*S*-(*p*-tolyl)thiohydroxylamine (3bo):** The reaction was performed following the General Procedure with **1b** (32.3 mg, 0.15 mmol), benzofuran-5-ylboronic acid (48.6 mg, 0.3 mmol), Cy_2NMe (48.2 μL, 0.23 mmol), $Cu(TFA)_2•H_2O$ (4.5 mg, 10 mol %) and **L3** (7.4 mg, 20 mol %). The crude product was purified by flash chromatography on silica gel (eluted with EtOAc:hexane = 1:100) to give the product **3bo** (40.7 mg, 82% yield) as a white solid. R_f = 0.3 (hexane); m.p. = 74.1–75.0 °C; ^1H NMR (400 MHz, $CDCl_3$) δ 7.60 (d, *J* = 2.2 Hz, 1H), 7.53 (d, *J* = 2.2 Hz, 1H), 7.41 (d, *J* = 8.8 Hz, 1H), 7.29 – 7.16 (m, 7H), 7.10 (d, *J* = 8.1 Hz, 2H), 6.97 – 6.89 (m, 1H), 6.68 (d, *J* = 1.3 Hz, 1H), 2.29 (s, 3H) ppm; ^{13}C NMR (100 MHz, $CDCl_3$) δ 152.5, 150.1, 146.0, 144.3, 137.5, 135.9, 129.9, 129.1, 128.4, 123.6, 122.2, 121.5, 119.2, 117.3, 112.0, 107.0, 21.1 ppm; IR (thin film): 3033, 2919, 1591, 1285, 1238, 1188, 1030, 963, 844, 733 cm^{-1}; HRMS calculated for $C_{21}H_{18}NOS^+$ 332.1104, found 332.1110 $[M+H]^+$.

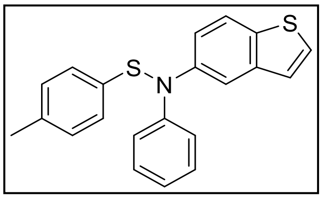

***N*-(Benzo[b]thiophen-5-yl)-*N*-phenyl-*S*-(*p*-tolyl)thiohydroxylamine (3bp):** The reaction was performed following the General Procedure with **1b** (32.3 mg, 0.15 mmol), benzo[b]thiophen-5-ylboronic acid (53.4 mg, 0.3 mmol), Cy_2NMe (48.2 μL, 0.23 mmol), $Cu(TFA)_2•H_2O$ (4.5 mg, 10 mol %) and **L3** (7.4 mg, 20 mol %). The crude product was purified by flash chromatography on silica gel (eluted with EtOAc:hexane = 1:100) to give the product **3bp** (27.1 mg, 52% yield) as a white solid. R_f = 0.2

(EtOAc:hexane = 1:100); m.p. = 86.7–89.1 °C; ^{1}H NMR (400 MHz, $CDCl_3$) δ 7.77 – 7.71 (m, 2H), 7.42 (d, J = 5.4 Hz, 1H), 7.33 (dd, J = 8.8, 2.2 Hz, 1H), 7.31 – 7.16 (m, 7H), 7.10 (d, J = 8.1 Hz, 2H), 6.99 (t, J = 7.1 Hz, 1H), 2.29 (s, 3H) ppm; ^{13}C NMR (100 MHz, $CDCl_3$) δ 149.5, 146.0, 140.7, 137.4, 135.9, 135.5, 129.9, 129.2, 127.6, 123.9, 123.5, 123.1, 122.5, 121.3, 120.8, 117.9, 21.1 ppm; IR (thin film): 2919, 2851, 1590, 1261, 1207, 1144, 1030, 930, 852, 798 cm^{-1}; HRMS calculated for $C_{21}H_{18}NS_2^+$ 348.0875, found 348.0878 [M+H]$^+$.

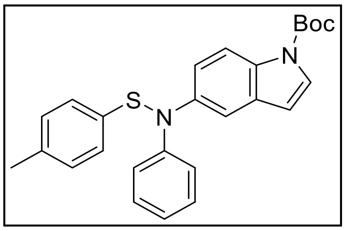

***tert*-Butyl-(phenyl(*p*-tolylthio)amino)-1*H*-indole-1-carboxylate (3bq):** The reaction was performed following the General Procedure with **1b** (32.3 mg, 0.15 mmol), (1-(*tert*-butoxycarbonyl)-1*H*-indol-5-yl)boronic acid (78.3 mg, 0.3 mmol), Cy_2NMe (48.2 μL, 0.23 mmol), $Cu(TFA)_2$•H_2O (4.5 mg, 10 mol %) and **L3** (7.4 mg, 20 mol %). The crude product was purified by flash chromatography on silica gel (eluted with EtOAc:hexane = 1:100) to give the product **3bq** (49.7 mg, 77% yield) as a colorless oil. R_f = 0.2 (EtOAc:hexane = 1:100); ^{1}H NMR (400 M Hz, $CDCl_3$) δ 8.07 (d, J = 8.6 Hz, 1H), 7.61 (d, J = 3.6 Hz, 1H), 7.54 (d, J = 2.2 Hz, 1H), 7.34 – 7.21 (m, 7H), 7.13 (d, J = 8.2 Hz, 2H), 6.97 (t, J = 6.6 Hz, 1H), 6.51 (d, J = 3.7 Hz, 1H), 2.33 (s, 3H), 1.69 (s, 9H) ppm; ^{13}C NMR (100 MHz, $CDCl_3$) δ 150.0, 149.8, 144.1, 137.6, 135.8, 131.6, 129.9, 129.1, 126.9, 123.5, 121.7, 121.6, 119.6, 116.6, 115.9, 107.5, 83.9, 28.3, 21.1 ppm; IR (thin film): 3033, 2919, 1590, 1486, 1207, 1144, 1030, 965, 852, 747 cm^{-1}; HRMS calculated for $C_{26}H_{27}N_2O_2S^+$ 431.1788, found 431.1787 [M+H]$^+$.

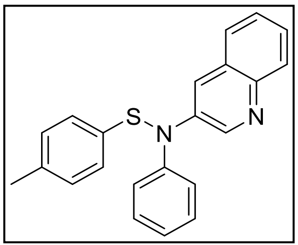

***N*-Phenyl-*N*-(quinolin-3-yl)-*S*-(*p*-tolyl)thiohydroxylamine (3br):** The reaction was performed following the General Procedure with **1b** (32.3 mg, 0.15 mmol), quinolin-6-ylboronic acid (51.9 mg, 0.3 mmol), Cy_2NMe (48.2 μL, 0.23 mmol), $Cu(TFA)_2$•H_2O (4.5 mg, 10 mol %) and **L3** (7.4 mg, 20 mol %). The crude product was purified by flash chromatography on silica gel (eluted with EtOAc:hexane = 1:20) to give the product **3br** (20.5 mg, 40% yield) as a colorless oil. R_f = 0.2 (EtOAc:hexane = 1:20); ^{1}H NMR (400 MHz, $CDCl_3$) δ 8.94 (d, J = 2.6 Hz, 1H), 8.03 (d, J = 8.4 Hz, 1H), 7.97 (d, J = 2.4 Hz, 1H), 7.66 (d, J = 8.0 Hz, 1H), 7.58 (t, J = 7.1 Hz, 1H), 7.48 (t, J = 7.4 Hz, 1H), 7.39 – 7.27 (m, 4H), 7.21 (d, J = 8.2 Hz, 2H), 7.11 (d, J = 7.7 Hz, 3H), 2.29 (s, 3H) ppm; ^{13}C NMR (100 MHz, $CDCl_3$) δ 148.2, 147.1, 144.7, 142.5, 136.8, 136.2, 130.1, 129.6, 129.2, 128.6, 128.0, 127.2(1), 127.1(8), 125.0, 124.2(3), 124.2(0), 122.2, 21.1 ppm; IR (thin film): 3033, 2919, 1590, 1487, 1241, 1201, 988, 943, 800 cm^{-1}; HRMS calculated for $C_{22}H_{19}N_2S^+$ 343.1263, found 343.1266 [M+H]$^+$.

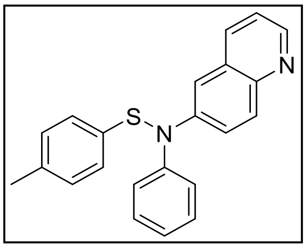

***N*-Phenyl-*N*-(quinolin-6-yl)-*S*-(*p*-tolyl)thiohydroxylamine (3bs):** The reaction was performed following the General Procedure with **1b** (32.3 mg, 0.15 mmol), quinolin-3-ylboronic acid (51.9 mg, 0.3 mmol), Cy_2NMe (48.2 µL, 0.23 mmol), $Cu(TFA)_2•H_2O$ (4.5 mg, 10 mol %) and **L3** (7.4 mg, 20 mol %). The crude product was purified by flash chromatography on silica gel (eluted with EtOAc:hexane = 1:20) to give the product **3bs** (42.1 mg, 82% yield) as a colorless oil. R_f = 0.2 (EtOAc:hexane = 1:20); ^{1}H NMR (400 MHz, $CDCl_3$) δ 8.76 (d, J = 2.9 Hz, 1H), 8.03 – 7.89 (m, 2H), 7.73 (dd, J = 9.2, 2.5 Hz, 1H), 7.61 (d, J = 2.4 Hz, 1H), 7.40 – 7.26 (m, 5H), 7.20 (d, J = 8.1 Hz, 2H), 7.11 (t, J = 8.0 Hz, 3H), 2.29 (s, 3H) ppm; ^{13}C NMR (100 MHz, $CDCl_3$) δ 148.9, 148.5, 147.2, 145.2, 136.8, 136.3, 135.2, 130.3, 130.0, 129.5, 129.1, 125.1, 124.4, 123.7, 123.4, 121.5, 116.3, 21.1 ppm; IR (thin film): 3033, 2919, 1590, 1486, 1374, 1153, 1030, 939, 800, 750 cm^{-1}; HRMS calculated for $C_{22}H_{19}N_2S^+$ 343.1263, found 343.1267 $[M+H]^+$.

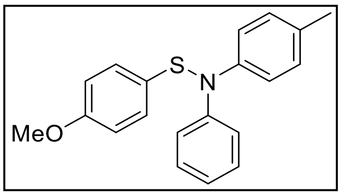

***S*-(4-Methoxyphenyl)-*N*-phenyl-*N*-(*p*-tolyl)thiohydroxylamine (3ca):** The reaction was performed following the General Procedure with *S*-(4-methoxyphenyl)-*N*-(*p*-tolyl)thiohydroxylamine (36.8 mg, 0.15 mmol), **2a** (40.8 mg, 0.3 mmol), Cy_2NMe (48.2 µL, 0.23 mmol), $Cu(TFA)_2•H_2O$ (4.5 mg, 10 mol %) and **L3** (7.4 mg, 20 mol %). The crude product was purified by flash chromatography on silica gel (eluted with hexane) to give the product **3ca** (27.0 mg, 56% yield) as a colorless oil. R_f = 0.2 (hexane); ^{1}H NMR (400 MHz, $CDCl_3$) δ 7.28 – 7.21 (m, 6H), 7.19 – 7.13 (m, 2H), 7.08 (d, J = 8.2 Hz, 2H), 6.99 – 6.93 (m, 1H), 6.86 – 6.80 (m, 2H), 3.76 (s, 3H), 2.31 (s, 3H) ppm; ^{13}C NMR (100 MHz, $CDCl_3$) δ 158.9, 149.6, 146.5, 133.6, 131.4, 130.0, 129.1, 127.2, 123.5, 122.2, 120.7, 114.8, 55.5, 20.9 ppm; IR (thin film): 2974, 1583, 1374, 1321, 1247, 1204, 1015, 966, 802, 693 cm^{-1}; HRMS calculated for $C_{20}H_{20}NOS^+$ 322.1260, found 322.1257 $[M+H]^+$.

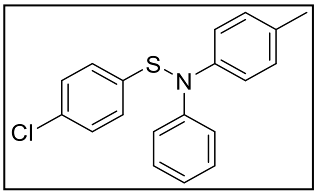

***S*-(4-Chlorophenyl)-*N*-phenyl-*N*-(*p*-tolyl)thiohydroxylamine (3da):** The reaction was performed following the General Procedure with *S*-(4-chlorophenyl)-*N*-(*p*-tolyl)thiohydroxylamine (37.4 mg, 0.15 mmol), **2a** (40.8 mg, 0.3 mmol), Cy_2NMe (48.2 µL, 0.23 mmol), $Cu(TFA)_2•H_2O$ (4.5 mg, 10 mol %) and **L3** (7.4 mg, 20 mol %). The crude product was purified by flash chromatography on silica gel (eluted with hexane) to give the product **3da** (34.1 mg, 70% yield) as a white solid. R_f = 0.5 (hexane); m.p. = 57.1–59.1 °C; ^{1}H NMR (400 MHz, $CDCl_3$) δ 7.27 – 7.20 (m, 6H), 7.17 (d, J = 7.2 Hz, 4H), 7.09 (d, J = 8.3 Hz, 2H), 7.02 – 6.96 (m, 1H), 2.31 (s, 3H) ppm; ^{13}C NMR (100 MHz, $CDCl_3$) δ 149.0, 146.0, 139.9, 134.0, 131.5, 130.1, 129.3, 129.2, 124.1, 123.2, 122.7, 120.7, 20.9 ppm; IR (thin film): 2974, 1591, 1505, 1225, 1153, 950, 692, 622 cm^{-1}; HRMS calculated for $C_{19}H_{17}ClNS^+$ 326.0765, found 326.0767 $[M+H]^+$.

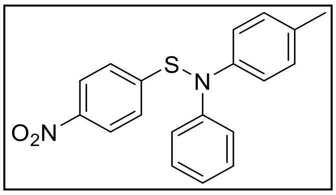

***S*-(4-Nitrophenyl)-*N*-phenyl-*N*-(*p*-tolyl)thiohydroxylamine (3ea):** The reaction was performed following the General Procedure with *S*-(4-nitrophenyl)-*N*-(*p*-tolyl)thiohydroxylamine (39.0 mg, 0.15 mmol), **2a** (40.8 mg, 0.3 mmol), Cy_2NMe (48.2 µL, 0.23 mmol), $Cu(TFA)_2•H_2O$ (4.5 mg, 10 mol %) and **L3** (7.4 mg, 20 mol %). The crude product was purified by flash chromatography on silica gel (eluted with EtOAc:hexane = 1:50) to give the product **3ea** (38.8 mg, 77% yield) as a yellow solid. R_f = 0.2 (EtOAc:hexane = 1:50); m.p. = 95.2–97.8 °C; ^1H NMR (400 MHz, $CDCl_3$) δ 8.13 (d, *J* = 8.9 Hz, 2H), 7.36 (d, *J* = 8.9 Hz, 2H), 7.30 – 7.16 (m, 6H), 7.12 (d, *J* = 8.3 Hz, 2H), 7.03 (t, *J* = 7.0 Hz, 1H), 2.32 (s, 3H) ppm; ^{13}C NMR (100 MHz, $CDCl_3$) δ 151.5, 148.2, 145.8, 145.3, 134.6, 130.2, 129.5, 124.4, 123.3, 123.1, 121.9, 120.7, 20.9 ppm; IR (thin film): 3853, 2920, 2364, 1596, 1575, 1334, 1276, 933, 813, 610 cm^{-1}; HRMS calculated for $C_{19}H_{17}N_2O_2S^+$ 337.1005, found 337.1013 $[M+H]^+$.

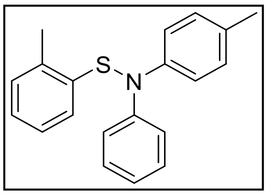

***N*-Phenyl-*S*-(*o*-tolyl)-N-(*p*-tolyl)thiohydroxylamine (3fa):** The reaction was performed following the General Procedure with *S*-(*o*-tolyl)-*N*-(*p*-tolyl)thiohydroxylamine (34.5 mg, 0.15 mmol), **2a** (40.8 mg, 0.3 mmol), Cy_2NMe (48.2 µL, 0.23 mmol), $Cu(TFA)_2•H_2O$ (4.5 mg, 10 mol %) and **L3** (7.4 mg, 20 mol %). The crude product was purified by flash chromatography on silica gel (eluted with hexane) to give the product **3fa** (41.2 mg, 90% yield) as a colorless oil. R_f = 0.5 (hexane); ^1H NMR (400 MHz, $CDCl_3$) δ 7.34 (d, *J* = 7.9 Hz, 1H), 7.29 – 7.19 (m, 6H), 7.15 – 6.94 (m, 6H), 2.30 (s, 3H), 2.22 (s, 3H) ppm; ^{13}C NMR (100 MHz, $CDCl_3$) δ 149.1, 146.1, 139.5, 133.7, 131.5, 130.4, 130.0, 129.2, 126.5, 125.1, 123.3, 122.5, 121.8, 120.8, 20.9, 18.5 ppm; IR (thin film): 2919, 1589, 1379, 1172, 1044, 1030, 948, 930, 810, 713 cm^{-1}; HRMS calculated for $C_{20}H_{20}NS^+$ 306.1311, found 306.1313 $[M+H]^+$.

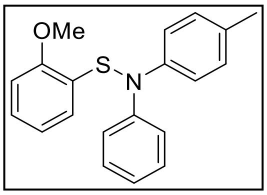

***S*-(2-Methoxyphenyl)-*N*-phenyl-*N*-(*p*-tolyl)thiohydroxylamine (3ga):** The reaction was performed following the General Procedure with *S*-(2-methoxyphenyl)-*N*-(*p*-tolyl)thiohydroxylamine (36.8 mg, 0.15 mmol), **2a** (40.8 mg, 0.3 mmol), Cy_2NMe (48.2 µL, 0.23 mmol), $Cu(TFA)_2•H_2O$ (4.5 mg, 10 mol %) and **L3** (7.4 mg, 20 mol %). The crude product was purified by flash chromatography on silica gel (eluted with hexane) to give the product **3ga** (40.0 mg, 83% yield) as a white solid. R_f = 0.3 (hexane); m.p. = 103.6–105.7 °C; ^1H NMR (400 MHz, $CDCl_3$) δ 7.32 – 7.18 (m, 7H), 7.13 – 7.04 (m, 3H), 6.96 (t, *J* = 7.1 Hz, 1H), 6.89 (t, *J* = 7.6, 1.0 Hz, 1H), 6.81 (d, *J* = 8.1 Hz, 1H), 3.84 (s, 3H), 2.30 (s, 3H) ppm; ^{13}C NMR (100 MHz, $CDCl_3$) δ 153.5, 149.0, 145.9, 133.4, 129.8, 129.0, 126.0, 123.2, 122.8, 122.3, 121.3, 120.8, 110.3, 55.7, 20.8 ppm; IR (thin film): 3853, 2921, 1595, 1575, 1235, 1198, 1037, 939, 746, 690 cm^{-1}; HRMS calculated for $C_{20}H_{20}NOS^+$ 322.1260, found 322.1267 $[M+H]^+$.

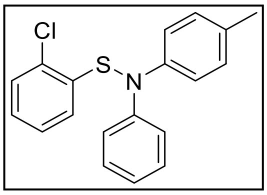

***S*-(2-Chlorophenyl)-*N*-phenyl-*N*-(*p*-tolyl)thiohydroxylamine (3ha):** The reaction was performed following the General Procedure with *S*-(2-chlorophenyl)-*N*-(*p*-tolyl)thiohydroxylamine (37.4 mg, 0.15 mmol), **2a** (40.8 mg, 0.3 mmol), Cy_2NMe (48.2 μL, 0.23 mmol), $Cu(TFA)_2•H_2O$ (4.5 mg, 10 mol %) and **L3** (7.4 mg, 20 mol %). The crude product was purified by flash chromatography on silica gel (eluted with hexane) to give the product **3ha** (41.4 mg, 85% yield) as a colorless oil. R_f = 0.5 (hexane); ^{1}H NMR (400 MHz, $CDCl_3$) δ 7.35 (dd, *J* = 7.9, 1.4 Hz, 1H), 7.29 – 7.20 (m, 7H), 7.17 (t, *J* = 7.6 Hz, 1H), 7.12 – 7.02 (m, 3H), 7.02 – 6.96 (m, 1H), 2.31 (s, 3H) ppm; ^{13}C NMR (100 MHz, $CDCl_3$) δ 148.6, 145.6, 139.7, 134.0, 130.0, 129.8, 129.3, 127.3, 126.9, 126.2, 123.4, 123.2, 122.8, 120.8, 20.9 ppm; IR (thin film): 3026, 2920, 1592, 1446, 1432, 1258, 1196, 948, 811, 743 cm^{-1}; HRMS calculated for $C_{19}H_{17}ClNS^+$ 326.0765, found 326.0767 $[M+H]^+$.

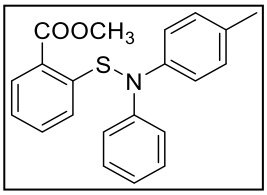

Methyl 2-((phenyl(*p*-tolyl)amino)thio)benzoate (3ia): The reaction was performed following the General Procedure with methyl 2-((*p*-tolylamino)thio)benzoate (41.0 mg, 0.15 mmol), **2a** (40.8 mg, 0.3 mmol), Cy_2NMe (48.2 μL, 0.23 mmol), $Cu(TFA)_2•H_2O$ (4.5 mg, 10 mol %) and **L3** (7.4 mg, 20 mol %). The crude product was purified by flash chromatography on silica gel (eluted with EtOAc:hexane = 1:50) to give the product **3ia** (46.1 mg, 88% yield) as a white solid. R_f = 0.2 (EtOAc:hexane = 1:50); m.p. = 97.1–98.7 °C; ^{1}H NMR (400 MHz, $CDCl_3$) δ 8.03 (dd, *J* = 7.8, 1.3 Hz, 1H), 7.66 (dd, *J* = 8.2, 0.8 Hz, 1H), 7.48 – 7.39 (m, 1H), 7.29 – 7.19 (m, 6H), 7.18 – 7.13 (m, 1H), 7.07 (d, *J* = 8.1 Hz, 2H), 6.96 (t, *J* = 7.0 Hz, 1H), 3.92 (s, 3H), 2.29 (s, 3H) ppm; ^{13}C NMR (100 MHz, $CDCl_3$) δ 167.1, 148.7, 147.8, 145.6, 133.6, 133.1, 131.5, 129.9, 129.2, 124.5, 124.1, 123.2, 122.9, 122.4, 120.7, 52.4, 20.9 ppm; IR (thin film): 3033, 2947, 1702, 1586, 1483, 1455, 1267, 1096, 972, 710 cm^{-1}; HRMS calculated for $C_{21}H_{20}NO_2S^+$ 350.1209, found 350.1210 $[M+H]^+$.

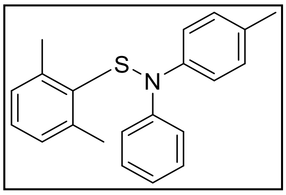

***S*-(2,6-Dimethylphenyl)-*N*-phenyl-*N*-(*p*-tolyl)thiohydroxylamine (3ja):** The reaction was performed following the General Procedure with *S*-(2,6-dimethylphenyl)-*N*-(*p*-tolyl)thiohydroxylamine (36.5 mg, 0.15 mmol), **2a** (40.8 mg, 0.3 mmol), Cy_2NMe (48.2 μL, 0.23 mmol), $Cu(TFA)_2•H_2O$ (4.5 mg, 10 mol %) and **L3** (7.4 mg, 20 mol %). The crude product was purified by flash chromatography on silica gel (eluted with hexane) to give the product **3ja** (38.3 mg, 80% yield) as white solid. R_f = 0.5 (hexane); m.p. = 84.9–86.7 °C; ^{1}H NMR (400 MHz, $CDCl_3$) δ 7.23 – 7.15 (m, 2H), 7.12 – 7.06 (m, 3H), 7.02 (d, *J* = 9.1 Hz, 4H), 6.92 (t, *J* = 7.3 Hz, 3H), 2.29 (s, 3H), 2.27 (s, 6H) ppm; ^{13}C NMR (100 MHz, $CDCl_3$) δ 150.5, 147.2, 142.3, 136.6, 133.5, 129.8, 129.2, 128.9, 128.6, 124.3, 121.8, 120.9, 22.6, 21.0 ppm; IR (thin film): 3025, 2974, 1592, 1575, 1503, 1197, 1170, 920, 704, 650 cm^{-1}; HRMS calculated for $C_{21}H_{22}NS^+$ 320.1467,

found 320.1476 [M+H]$^+$.

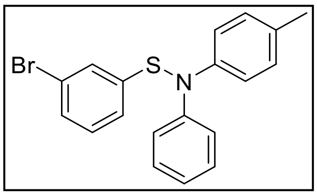

***S*-(3-Bromophenyl)-*N*-phenyl-*N*-(*p*-tolyl)thiohydroxylamine (3ka):** The reaction was performed following the General Procedure with *S*-(3-bromophenyl)-*N*-(*p*-tolyl)thiohydroxylamine (43.8 mg, 0.15 mmol), **2a** (40.8 mg, 0.3 mmol), Cy_2NMe (48.2 µL, 0.23 mmol), $Cu(TFA)_2$•H_2O (4.5 mg, 10 mol %) and **L3** (7.4 mg, 20 mol %). The crude product was purified by flash chromatography on silica gel (eluted with hexane) to give the product **3ka** (48.7 mg, 88% yield) as a colorless oil. R_f = 0.2 (hexane); ^{1}H NMR (400 MHz, $CDCl_3$) δ 7.37 (t, J = 1.7 Hz, 1H), 7.27 – 7.06 (m, 11H), 7.03 – 6.96 (m, 1H), 2.31 (s, 3H) ppm; ^{13}C NMR (100 MHz, $CDCl_3$) δ 148.9, 145.9, 144.0, 134.0, 130.5, 130.1, 129.3, 128.7, 124.9, 123.3, 123.2, 122.9, 121.0, 120.8, 20.9 ppm; IR (thin film): 3026, 1592, 1572, 1259, 1195, 1109, 948, 811, 712 cm^{-1}; HRMS calculated for $C_{19}H_{17}BrNS^+$ 370.0260, found 370.0264 [M+H]$^+$.

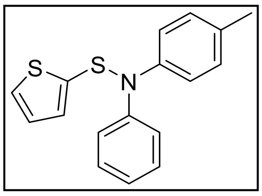

***N*-Phenyl-*S*-(thiophen-2-yl)-*N*-(*p*-tolyl)thiohydroxylamine (3la):** The reaction was performed following the General Procedure with *S*-(thiophen-2-yl)-*N*-(*p*-tolyl)thiohydroxylamine (33.2 mg, 0.15 mmol), **2a** (40.8 mg, 0.3 mmol), Cy_2NMe (48.2 µL, 0.23 mmol), $Cu(TFA)_2$•H_2O (4.5 mg, 10 mol %) and **L3** (7.4 mg, 20 mol %). The crude product was purified by flash chromatography on silica gel (eluted with hexane) to give the product **3la** (24.1 mg, 54% yield) as a colorless oil. R_f = 0.2 (hexane); ^{1}H NMR (400 MHz, $CDCl_3$) δ 7.41 (dd, J = 5.3, 1.2 Hz, 1H), 7.29 – 7.18 (m, 4H), 7.16 (dd, J = 3.6, 1.2 Hz, 1H), 7.13 – 7.05 (m, 4H), 7.00 – 6.94 (m, 2H), 2.33 (s, 3H) ppm; ^{13}C NMR (100 MHz, $CDCl_3$) δ 148.9, 145.9, 136.8, 134.0, 133.9, 131.2, 130.0, 129.1, 127.3, 124.1, 122.3, 121.0, 21.0 ppm; IR (thin film): 3673, 2981, 2921, 1592, 1487, 1261, 1083, 919, 809, 691 cm^{-1}; HRMS calculated for $C_{17}H_{16}NS_2^+$ 298.0719, found 298.0719 [M+H]$^+$.

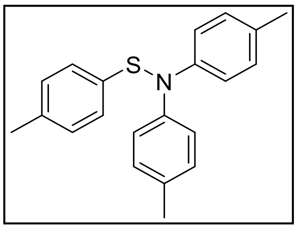

***N,N,S*-Tri-*p*-tolylthiohydroxylamine (3ma):** The reaction was performed following the General Procedure with *N,S*-di-*p*-tolylthiohydroxylamine (34.5 mg, 0.15 mmol), **2a** (40.8 mg, 0.3 mmol), Cy_2NMe (48.2 µL, 0.23 mmol), $Cu(TFA)_2$•H_2O (4.5 mg, 10 mol %) and **L3** (7.4 mg, 20 mol %). The crude product was purified by flash chromatography on silica gel (eluted with hexane) to give the product **3ma** (35.9 mg, 75% yield) as a white solid. R_f = 0.5 (hexane); m.p. = 84.1–85.6 °C; ^{1}H NMR (400 MHz, $CDCl_3$) δ 7.16 (t, J = 6.9 Hz, 6H), 7.10 – 7.02 (m, 6H), 2.29 (s, 9H) ppm; ^{13}C NMR (100 MHz, $CDCl_3$) δ 146.8, 137.8, 135.6, 132.6, 129.8, 123.2, 121.9, 21.1, 20.8 ppm; IR (thin film): 3023, 2920, 2360, 1605, 1502, 1277, 1014, 950, 804, 667 cm^{-1}; HRMS calculated for $C_{21}H_{22}NS^+$ 320.1467, found 320.1468 [M+H]$^+$.

N-(4-Methoxyphenyl)-N,S-di-p-tolylthiohydroxylamine (3na): The reaction was performed following the General Procedure with N-(4-methoxyphenyl)-S-(p-tolyl)thiohydroxylamine (36.8 mg, 0.15 mmol), **2a** (40.8 mg, 0.3 mmol), Cy_2NMe (48.2 μL, 0.23 mmol), $Cu(TFA)_2$•H_2O (4.5 mg, 10 mol %) and **L3** (7.4 mg, 20 mol %). The crude product was purified by flash chromatography on silica gel (eluted with hexane) to give the product **3na** (31.1 mg, 62% yield) as a white solid. R_f = 0.4 (hexane); m.p. = 106.4–107.1 °C; ^{1}H NMR (400 MHz, $CDCl_3$) δ 7.23 – 7.18 (m, 2H), 7.18 – 7.06 (m, 6H), 7.02 (d, J = 8.5 Hz, 2H), 6.86 – 6.76 (m, 2H), 3.77 (s, 3H), 2.29 (s, 3H), 2.27 (s, 3H) ppm; ^{13}C NMR (100 MHz, $CDCl_3$) δ 156.4, 147.4, 142.3, 137.9, 135.7, 131.3, 129.8, 129.7, 125.2, 123.4, 119.8, 114.6, 55.6, 21.1, 20.7 ppm; IR (thin film): 3386, 2975, 2361, 1648, 1502, 1241, 1043, 879, 661, 651 cm^{-1}; HRMS calculated for $C_{21}H_{22}NOS^+$ 336.1417, found 336.1420 [M+H]$^+$.

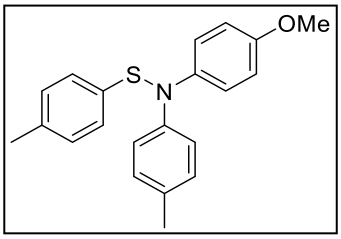

N-(4-Fluorophenyl)-N,S-di-p-tolylthiohydroxylamine (3oa): The reaction was performed following the General Procedure with N-(4-fluorophenyl)-S-(p-tolyl)thiohydroxylamine (35.0 mg, 0.15 mmol), **2a** (40.8 mg, 0.3 mmol), Cy_2NMe (48.2 μL, 0.23 mmol), $Cu(TFA)_2$•H_2O (4.5 mg, 10 mol %) and **L3** (7.4 mg, 20 mol %). The crude product was purified by flash chromatography on silica gel (eluted with hexane) to give the product **3oa** (36.3 mg, 75% yield) as a colorless oil. R_f = 0.5 (hexane); ^{1}H NMR (400 MHz, $CDCl_3$) δ 7.25 – 7.18 (m, 2H), 7.15 (d, J = 8.5 Hz, 4H), 7.12 – 7.03 (m, 4H), 6.96 – 6.89 (m, 2H), 2.29 (s, 6H) ppm; ^{13}C NMR (100 MHz, $CDCl_3$) δ 159.0 (d, $J_{C\text{-}F}$ = 242.5 Hz), 146.7, 145.4 (d, $J_{C\text{-}F}$ = 2.7 Hz), 137.4, 136.0, 132.8, 129.9, 123.6 (d, $J_{C\text{-}F}$ = 8.0 Hz), 123.5, 121.7, 115.8 (d, $J_{C\text{-}F}$ = 22.6 Hz), 21.1, 20.8 ppm; ^{19}F NMR (376 MHz, $CDCl_3$) δ -120.4 ppm; IR (thin film): 2921, 2220, 1672, 1594, 1497, 1174, 1081, 951, 800, 611 cm^{-1}; HRMS calculated for $C_{20}H_{19}FNS^+$ 324.1217, found 324.1217 [M+H]$^+$.

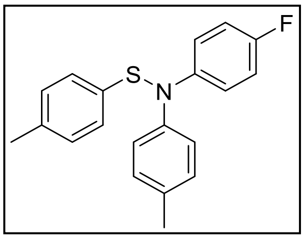

N-(4-Chlorophenyl)-N,S-di-p-tolylthiohydroxylamine (3pa): The reaction was performed following the General Procedure with N-(4-Chlorophenyl)-S-(p-tolyl)thiohydroxylamine (37.4 mg, 0.15 mmol), **2a** (40.8 mg, 0.3 mmol), Cy_2NMe (48.2 μL, 0.23 mmol), $Cu(TFA)_2$•H_2O (4.5 mg, 10 mol %) and **L3** (7.4 mg, 20 mol %). The crude product was purified by flash chromatography on silica gel (eluted with hexane) to give the product **3pa** (38.1 mg, 75% yield) as a colorless oil. R_f = 0.5 (hexane); ^{1}H NMR (400 MHz, $CDCl_3$) δ 7.20 – 7.15 (m, 6H), 7.15 – 7.11 (m, 2H), 7.09 (dd, J = 8.5, 2.3 Hz, 4H), 2.31 (s, 3H), 2.29 (s, 3H) ppm; ^{13}C NMR (100 MHz, $CDCl_3$) δ 148.2, 146.0, 137.0, 136.1, 134.2, 130.1, 129.9, 129.0, 127.1, 123.6, 121.6, 21.1, 20.9 ppm; IR (thin film): 3675, 2970, 1590, 1505, 1455, 1199, 1172, 927, 814, 694 cm^{-1}; HRMS calculated for $C_{20}H_{19}ClNS^+$ 340.0921, found 340.0916 [M+H]$^+$.

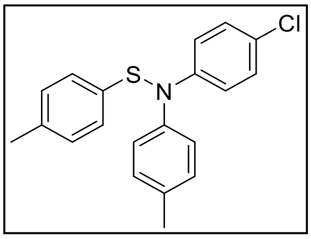

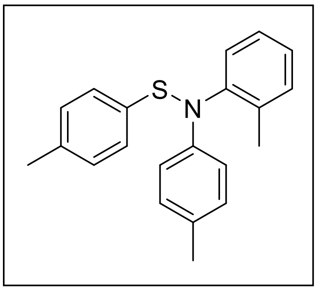

***N*-(*o*-Tolyl)-*N*,*S*-di-*p*-tolylthiohydroxylamine** (**3qa**): The reaction was performed following the General Procedure with *N*-(*o*-tolyl)-*S*-(*p*-tolyl)thiohydroxylamine (34.4 mg, 0.15 mmol), **2a** (40.8 mg, 0.3 mmol), Cy_2NMe (48.2 μL, 0.23 mmol), $Cu(TFA)_2{\bullet}H_2O$ (4.5 mg, 10 mol %) and **L3** (7.4 mg, 20 mol %) at 35 °C for 24 h. The crude product was purified by flash chromatography on silica gel (eluted with hexane) to give the product **3qa** (29.2 mg, 61% yield) as a colorless oil. R_f = 0.5 (EtOAc:hexane = 1:100); ^{1}H NMR (600 MHz, $CDCl_3$) δ 7.35 – 7.28 (m, 3H), 7.26 – 7.19 (m, 3H), 7.16 (d, *J* = 8.0 Hz, 2H), 7.06 (d, *J* = 8.4 Hz, 2H), 6.99 (d, *J* = 8.6 Hz, 2H), 2.36 (s, 3H), 2.32 (s, 3H), 2.20 (s, 3H) ppm; ^{13}C NMR (150 MHz, $CDCl_3$) δ 147.3, 146.4, 137.2, 136.2, 136.1, 131.6, 129.7, 129.6, 129.2, 128.2, 127.3, 127.1, 124.9, 115.7, 21.1, 20.4, 18.3 ppm; IR (thin film): 1610, 1501, 1264, 1176, 1115, 804, 756, 719 cm^{-1}; HRMS calculated for $C_{21}H_{22}NS^+$ 320.1467, found 320.1465 $[M+H]^+$.

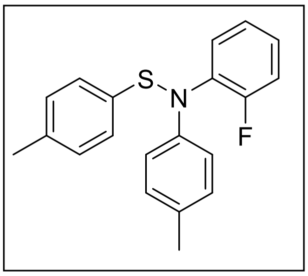

***N*-(2-Fluorophenyl)-*N*,*S*-di-*p*-tolylthiohydroxylamine** (**3ra**): The reaction was performed following the General Procedure with *N*-(2-fluorophenyl)-*S*-(*p*-tolyl)thiohydroxylamine (35.0 mg, 0.15 mmol), **2a** (40.8 mg, 0.3 mmol), Cy_2NMe (48.2 μL, 0.23 mmol), $Cu(TFA)_2{\bullet}H_2O$ (4.5 mg, 10 mol %) and **L3** (7.4 mg, 20 mol %) for 12 h. The crude product was purified by flash chromatography on silica gel (eluted with hexane) to give the product **3ra** (41.2 mg, 85% yield) as a colorless oil. R_f = 0.5 (EtOAc:hexane = 1:100); ^{1}H NMR (400 MHz, $CDCl_3$) δ 7.42 (d, *J* = 1.5 Hz, 1H), 7.21 (d, *J* = 1.5 Hz, 1H), 7.19 – 7.18 (m, 1H), 7.17 – 7.13 (m, 1H), 7.13 – 7.05 (m, 4H), 7.01 – 7.00 (m, 4H), 2.29 (s, 3H), 2.26 (s, 3H) ppm; ^{13}C NMR (100 MHz, $CDCl_3$) δ 157.9 (d, $J_{C\text{-}F}$ = 250.8 Hz), 146.9, 137.2, 135.9, 135.6, 130.6, 129.8, 129.6, 129.1, 127.4 (d, $J_{C\text{-}F}$ = 7.8 Hz), 125.0 (d, $J_{C\text{-}F}$ = 3.8 Hz), 123.4, 116.9 (d, $J_{C\text{-}F}$ = 20.1 Hz), 116.8, 21.0, 20.5 ppm; ^{19}F NMR (377 MHz, $CDCl_3$) δ -118.1 ppm; IR (thin film): 1608, 1496, 1452, 1292, 1260, 1224, 930, 804 cm^{-1}; HRMS calculated for $C_{20}H_{19}FNS^+$ 324.1217, found 324.1215 $[M+H]^+$.

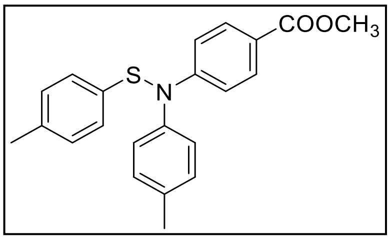

Methyl 4-(*p*-tolyl(*p*-tolylthio)amino)benzoate (3sa): The reaction was performed following the General Procedure with methyl 4-((*p*-tolylthio)amino)benzoate (41.0 mg, 0.15 mmol), **2a** (40.8 mg, 0.3 mmol), Cy_2NMe (48.2 μL, 0.23 mmol), $Cu(TFA)_2{\bullet}H_2O$ (4.5 mg, 10 mol %) and **L3** (7.4 mg, 20 mol %). The crude product was purified by flash chromatography on silica gel (eluted with EtOAc:hexane = 1:50) to give the product **3sa** (35.4 mg, 65% yield) as a colorless oil. R_f = 0.5 (EtOAc:hexane = 1:50); ^{1}H NMR (400 MHz, $CDCl_3$) δ 7.91 – 7.84 (m, 2H), 7.25 – 7.16 (m, 4H), 7.16 – 7.12 (m, 4H), 7.09 (d, *J* = 8.2 Hz, 2H), 3.86 (s, 3H), 2.34 (s, 3H), 2.29 (s, 3H) ppm; ^{13}C NMR (100 MHz, $CDCl_3$) δ 167.0, 153.8, 145.2, 136.6,

136.2, 136.0, 131.0, 130.4, 130.0, 125.9, 124.3, 122.3, 117.3, 51.9, 21.1(4), 21.1(1) ppm; IR (thin film): 3670, 2987, 1713, 1598, 1307, 1261, 1167, 928, 801, 695 cm^{-1}; HRMS calculated for $C_{22}H_{22}NO_2S^+$ 364.1366, found 364.1359 $[M+H]^+$.

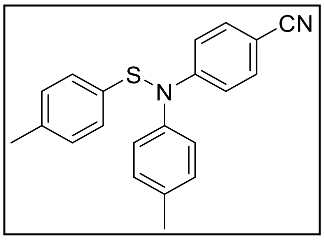

4-(*p*-Tolyl(*p*-tolylthio)amino)benzonitrile (3ta): The reaction was performed following the General Procedure with 4-((*p*-tolylthio)amino)benzonitrile (36.0 mg, 0.15 mmol), **2a** (40.8 mg, 0.3 mmol), Cy_2NMe (48.2 μL, 0.23 mmol), $Cu(TFA)_2$•H_2O (4.5 mg, 10 mol %) and **L3** (7.4 mg, 20 mol %). The crude product was purified by flash chromatography on silica gel (eluted with EtOAc:hexane = 1:20) to give the product **3ta** (25.7 mg, 52% yield) as a colorless oil. R_f = 0.4 (EtOAc:hexane = 1:20); ^{1}H NMR (400 MHz, $CDCl_3$) δ 7.48 – 7.43 (m, 2H), 7.23 (d, J = 9.0 Hz, 2H), 7.19 – 7.09 (m, 8H), 2.35 (s, 3H), 2.31 (s, 3H) ppm; ^{13}C NMR (100 MHz, $CDCl_3$) δ 153.6, 144.5, 137.1, 136.8, 135.4, 133.3, 130.6, 130.1, 126.3, 124.7, 119.7, 117.6, 103.0, 21.1(7), 21.1(5) ppm; IR (thin film): 2972, 2920, 1671, 1594, 1498, 1283, 1042, 951, 801, 611 cm^{-1}; HRMS calculated for $C_{21}H_{19}N_2S^+$ 331.1263, found 331.1260 $[M+H]^+$.

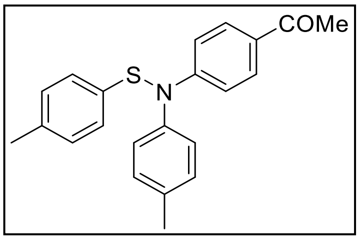

1-(4-(*p*-Tolyl(*p*-tolylthio)amino)phenyl)ethan-1-one (3ua): The reaction was performed following the General Procedure with 1-(4-((*p*-tolylthio)amino)phenyl)ethan-1-one (38.6 mg, 0.15 mmol), **2a** (40.8 mg, 0.3 mmol), Cy_2NMe (48.2 μL, 0.23 mmol), $Cu(TFA)_2$•H_2O (4.5 mg, 10 mol %) and **L3** (7.4 mg, 20 mol %). The crude product was purified by flash chromatography on silica gel (eluted with EtOAc:hexane = 1:20) to give the product **3ua** (32.3 mg, 62% yield) as a colorless oil. R_f = 0.4 (EtOAc:hexane = 1:20); ^{1}H NMR (400 MHz, $CDCl_3$) δ 7.86 – 7.78 (m, 2H), 7.26 – 7.21 (m, 2H), 7.21 – 7.12 (m, 6H), 7.10 (d, J = 8.2 Hz, 2H), 2.52 (s, 3H), 2.34 (s, 3H), 2.30 (s, 3H) ppm; ^{13}C NMR (100 MHz, $CDCl_3$) δ 196.7, 154.0, 145.0, 136.7, 136.2, 136.0, 130.4, 130.0(2), 129.9(9), 126.0, 124.4, 117.2, 26.4, 21.1(4), 21.1(2) ppm; IR (thin film): 3750, 2972, 2360, 1671, 1590, 1419, 1356, 955, 801, 647 cm^{-1}; HRMS calculated for $C_{22}H_{22}NOS^+$ 348.1417, found 348.1411 $[M+H]^+$.

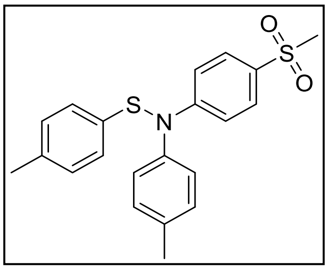

***N*-(4-(Methylsulfonyl)phenyl)-*N*,*S*-di-*p*-tolylthiohydroxylamine (3va):** The reaction was performed following the General Procedure with methyl *N*-(4-(methylsulfonyl)phenyl)-*S*-(*p*-tolyl)thiohydroxylamine (44.0 mg, 0.15 mmol), **2a** (40.8 mg, 0.3 mmol), Cy_2NMe (48.2 μL, 0.23 mmol), $Cu(TFA)_2$•H_2O (4.5 mg, 10 mol %) and **L3** (7.4 mg, 20 mol %). The crude product was purified by flash chromatography on silica gel (eluted with EtOAc:hexane = 1:20) to give the product **3va** (35.6 mg, 62% yield) as a white solid. R_f = 0.2 (EtOAc:hexane = 1:20); m.p. = 107.3–109.1 °C; ^{1}H NMR (400 MHz, $CDCl_3$) δ 7.77 – 7.70 (m, 2H), 7.31 (d, J = 9.0 Hz, 2H), 7.20 – 7.09 (m, 8H), 3.02 (s, 3H), 2.36 (s, 3H), 2.31 (s, 3H)

ppm; ^{13}C NMR (100 MHz, $CDCl_3$) δ 154.5, 144.6, 137.1, 136.8, 135.4, 131.5, 130.6, 130.1, 128.9, 126.2, 124.8, 117.4, 45.0, 21.1(7), 21.1(6) ppm; IR (thin film): 3673, 2987, 1583, 1506, 1287, 1263, 954, 803, 769 cm^{-1}; HRMS calculated for $C_{21}H_{22}NO_2S_2^+$ 384.1086, found 384.1080 $[M+H]^+$.

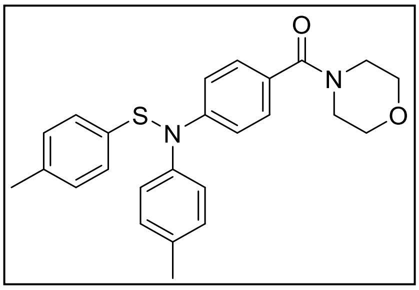

Morpholino(4-(*p*-tolyl(*p*-tolylthio)amino)phenyl)methanone (3wa): The reaction was performed following the General Procedure with morpholino(4-((*p*-tolylthio)amino)phenyl)methanone (49.2 mg, 0.15 mmol), **2a** (40.8 mg, 0.3 mmol), Cy_2NMe (48.2 µL, 0.23 mmol), $Cu(TFA)_2{\bullet}H_2O$ (4.5 mg, 10 mol %) and **L3** (7.4 mg, 20 mol %). The crude product was purified by flash chromatography on silica gel (eluted with EtOAc:hexane = 1:2) to give the product **3wa** (40.8 mg, 65% yield) as a colorless solid. R_f = 0.3 (EtOAc:hexane = 1:2); m.p. = 75.7–77.8 °C; ^1H NMR (400 MHz, $CDCl_3$) δ 7.28 – 7.31 (m, 2H), 7.27 – 7.22 (m, 2H), 7.21 – 7.18 (m, 2H), 7.16 – 7.07 (m, 6H), 3.77 – 3.54 (m, 8H), 2.32 (s, 3H), 2.30 (s, 3H) ppm; ^{13}C NMR (100 MHz, $CDCl_3$) δ 170.5, 151.2, 145.5, 136.6, 136.3, 135.3, 130.2, 129.9, 128.7, 127.6, 125.1, 123.8, 118.5, 67.1, 21.1, 21.0 ppm; IR (thin film): 2360, 1626, 1506, 1421, 1254, 1112, 1006, 837, 803, 760 cm^{-1}; HRMS calculated for $C_{25}H_{27}N_2O_2S^+$ 419.1788, found 419.1782 $[M+H]^+$.

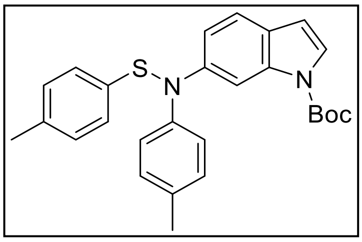

***tert*-Butyl 5-(*p*-tolyl(*p*-tolylthio)amino)-1*H*-indole-1-carboxylate (3xa):** The reaction was performed following the General Procedure with *tert*-butyl 5-((*p*-tolylthio)amino)-1*H*-indole-1-carboxylate (53.1 mg, 0.15 mmol), **2a** (40.8 mg, 0.3 mmol), Cy_2NMe (48.2 µL, 0.23 mmol), $Cu(TFA)_2{\bullet}H_2O$ (6.8 mg, 15 mol %) and **L3** (11.1 mg, 30 mol %). The crude product was purified by flash chromatography on silica gel (eluted with EtOAc:hexane = 1:100) to give the product **3xa** (38.0 mg, 57% yield) as a yellow oil. R_f = 0.3 (EtOAc:hexane = 1:100); ^1H NMR (400 MHz, $CDCl_3$) δ 8.00 (s, 1H), 7.56 (s, 1H), 7.47 (s, 1H), 7.30 – 7.23 (m, 1H), 7.20 – 7.15 (m, 4H), 7.11 – 7.00 (m, 4H), 6.50 – 6.41 (m, 1H), 2.29 (s, 6H), 1.65 (s, 9H) ppm; ^{13}C NMR (100 MHz, $CDCl_3$) δ 149.8, 147.4, 144.6, 137.9, 135.6, 131.8, 131.5, 129.8, 129.7, 126.8, 123.3, 120.7, 120.6, 115.7, 115.3, 107.4, 83.8, 28.3, 21.1, 20.8 ppm; IR (thin film): 2974, 1677, 1493, 1393, 1327, 1134, 1018, 814, 760 cm^{-1}; HRMS calculated for $C_{27}H_{29}N_2O_2S^+$ 445.1944, found 445.1937 $[M+H]^+$.

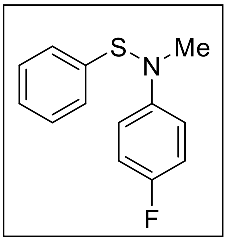

***N*-(4-Fluorophenyl)-*N*-methyl-*S*-phenylthiohydroxylamine (3yd):** The reaction was performed following the General Procedure with *N*-methyl-*S*-phenylthiohydroxylamine (21.9 mg, 0.15 mmol), **2d** (42.0 mg, 0.3 mmol), Cy_2NMe (48.2 Ml, 0.23 mmol), $Cu(TFA)_2$•H_2O (4.5 mg, 10 mol %), and **L3** (7.4 mg, 20 mol %) at 35 °C for 2 h. The crude product was purified by flash chromatography on silica gel (eluted with hexane) to give the product **3yd** (18.5 mg, 53% yield) as a colorless oil. R_f = 0.6 (hexane); ^{1}H NMR (400 MHz, $CDCl_3$) δ 7.33 – 7.29 (m, 2H), 7.19 – 7.10 (m, 5H), 7.00 – 6.95 (m, 2H), 3.49 (s, 3H) ppm; ^{13}C NMR (100 MHz, $CDCl_3$) δ 158.3 (d, J_{C-F} = 257.0 Hz), 146.1 (d, J_{C-F} = 2.0 Hz), 140.3, 129.1, 125.7, 122.8, 116.2 (d, J_{C-F} = 8.0 Hz), 115.5 (d, J_{C-F} = 23.0 Hz), 44.8 ppm; ^{19}F NMR (377 MHz, $CDCl_3$) δ -125.9 ppm. The spectroscopic data match the previously reported data.[4]

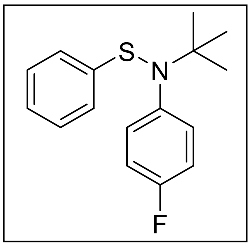

***N*-(*tert*-Butyl)-*N*-(4-fluorophenyl)-*S*-phenylthiohydroxylamine (3zd):** The reaction was performed following the General Procedure with *N*-(*tert*-butyl)-*S*-phenylthiohydroxylamine (27.2 mg, 0.15 mmol), **2d** (42.0 mg, 0.3 mmol), Cy_2NMe (48.2 µL, 0.23 mmol), $Cu(TFA)_2$•H_2O (4.5 mg, 10 mol %), and **L3** (7.4 mg, 20 mol %) for 12 h. The crude product was purified by flash chromatography on silica gel (eluted with hexane) to give the product **3zd** (30.9 mg, 75% yield) as a colorless oil. R_f = 0.8 (hexane); ^{1}H NMR (400 MHz, $CDCl_3$) δ 7.47 – 7.43 (m, 2H), 7.35 – 7.32 (m, 2H), 7.27 – 7.23 (m, 2H), 7.11 – 7.04 (m, 3H), 1.17 (s, 9H) ppm; ^{13}C NMR (150 MHz, $CDCl_3$) δ 163.3 (d, J_{C-F} = 246.0 Hz), 144.5, 136.5 (d, J_{C-F} = 3.0 Hz), 128.7 (d, J_{C-F} = 9.0 Hz), 128.4, 124.5, 122.5, 115.8 (d, J_{C-F} = 21.0 Hz), 54.8, 29.2 ppm; ^{19}F NMR (377 MHz, $CDCl_3$) δ -115.6 ppm; IR (thin film): 2348, 1502, 1225, 1074, 822, 803, 738, 691 cm^{-1}; HRMS calculated for $C_{16}H_{19}NFS^+$ 276.1217, found 276.1218 $[M+H]^+$.

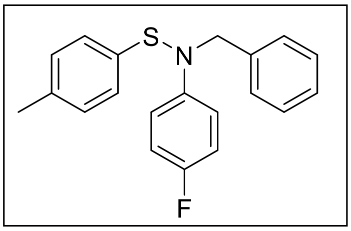

***N*-Benzyl-*N*-(4-fluorophenyl)-*S*-(*p*-tolyl)thiohydroxylamine (3aad):** The reaction was performed following the General Procedure with *N*-benzyl-*S*-(*p*-tolyl)thiohydroxylamine (34.4 mg, 0.15 mmol), **2d** (42.0 mg, 0.3 mmol), Cy_2NMe (48.2 µL, 0.23 mmol), $Cu(TFA)_2$•H_2O (4.5 mg, 10 mol %), and **L3** (7.4 mg, 20 mol %) at 35 °C for 12 h. The crude product was purified by flash chromatography on silica gel (eluted with hexane) to give the product **3aad** (31.0 mg, 64% yield) as a colorless oil. R_f = 0.7 (hexane); ^{1}H NMR (600 MHz, $CDCl_3$) δ 7.39 – 7.36 (m, 2H), 7.31 – 7.29 (m, 3H), 7.17 – 7.13 (m, 4H), 7.11 – 7.09 (m, 2H), 6.93 – 6.90 (m, 2H), 4.98 (s, 2H), 2.37 (s, 3H) ppm; ^{13}C NMR (100 MHz, $CDCl_3$) δ 158.4 (d, J_{C-F} = 237.0 Hz), 145.8 (d, J_{C-F} = 3.0 Hz), 137.9, 136.2, 136.0, 129.9, 128.7, 127.3, 126.6, 123.8, 117.1 (d, J_{C-F} = 8.0 Hz), 115.5 (d, J_{C-F} = 22.0 Hz), 60.7, 21.0 ppm; ^{19}F NMR (377 MHz, $CDCl_3$) δ -125.3 ppm; IR (thin

film): 1499, 1452, 1218, 1068, 802, 730, 696 cm^{-1}; HRMS calculated for $C_{20}H_{19}NFS^+$ 324.1217, found 324.1215 $[M+H]^+$.

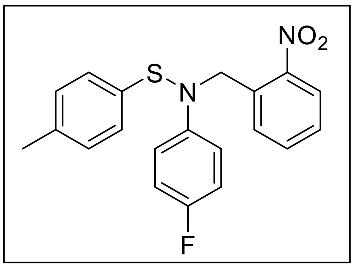

***N*-(4-Fluorophenyl)-*N*-(2-nitrobenzyl)-*S*-(*p*-tolyl)thiohydroxylamine (3abd):** The reaction was performed following the General Procedure with *N*-(2-nitrobenzyl)-*S*-(*p*-tolyl)thiohydroxylamine (41.1 mg, 0.15 mmol), **2d** (42.0 mg, 0.3 mmol), Cy_2NMe (48.2 μL, 0.23 mmol), $Cu(TFA)_2•H_2O$ (4.5 mg, 10 mol %) and **L3** (7.4 mg, 20 mol %) for 12 h. The crude product was purified by flash chromatography on silica gel (eluted with EtOAc:hexane = 1:20) to give the product **3abd** (39.7 mg, 72% yield) as a yellow oil. R_f = 0.5 (EtOAc:hexane = 1:10); ^{1}H NMR (600 MHz, $CDCl_3$) δ 8.14 – 8.13 (m, 1H), 7.60 – 7.57 (m, 1H), 7.47 – 7.39 (m, 2H), 7.14 (d, *J* = 8.0 Hz, 2H), 7.07 (d, *J* = 8.0 Hz, 2H) 7.04 – 7.02 (m, 2H), 6.91 – 6.88 (m, 2H), 5.32 (s, 2H), 2.32 (s, 3H) ppm; ^{13}C NMR (100 MHz, $CDCl_3$) δ 158.6 (d, J_{C-F} = 239.0 Hz), 147.9, 145.2 (d, J_{C-F} = 2.0 Hz), 136.4, 135.2, 134.0, 133.9, 130.0, 128.5, 128.2, 125.6, 123.9, 116.7 (d, J_{C-F} = 8.0 Hz), 115.8 (d, J_{C-F} = 2.0 Hz), 58.7, 21.0 ppm; ^{19}F NMR (377 MHz, $CDCl_3$) δ -124.7 ppm; IR (thin film): 1500, 1341, 1218, 1116, 908, 857, 803, 727 cm^{-1}; HRMS calculated for $C_{20}H_{18}N_2FO_2S^+$ 369.1068, found 369.1064 $[M+H]^+$.

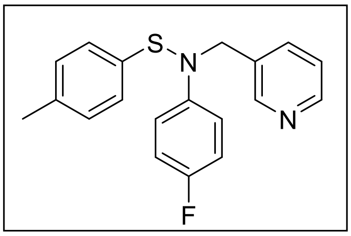

***N*-(4-Fluorophenyl)-*N*-(pyridin-3-ylmethyl)-*S*-(*p*-tolyl)thiohydroxylamine (3acd):** The reaction was performed following the General Procedure with *N*-(pyridin-3-ylmethyl)-*S*-(*p*-tolyl)thiohydroxylamine (34.5 mg, 0.15 mmol), **2d** (42.0 mg, 0.3 mmol), Cy_2NMe (48.2 μL, 0.23 mmol), $Cu(TFA)_2•H_2O$ (4.5 mg, 10 mol %), and **L3** (7.4 mg, 20 mol %) for 12 h. The crude product was purified by flash chromatography on silica gel (eluted with EtOAc:hexane = 1:10) to give the product **3acd** (30.6 mg, 63% yield) as a colorless oil. R_f = 0.4 (EtOAc:hexane = 1:10); ^{1}H NMR (400 MHz, $CDCl_3$) δ 8.57 – 8.49 (m, 2H), 7.60 – 7.56 (m, 1H), 7.26 – 7.22 (m, 1H), 7.13 – 7.09 (m, 4H), 7.06 – 7.04 (m, 2H), 6.88 – 6.92 (m, 2H), 4.89 (s, 2H), 2.32 (s, 3H) ppm; ^{13}C NMR (100 MHz, $CDCl_3$) δ 158.6 (d, J_{C-F} = 238.0 Hz), 148.7(1), 148.6(9), 145.6 (d, J_{C-F} = 2.0 Hz), 136.6, 135.3, 134.9, 133.5, 129.9, 124.6, 123.6, 117.6 (d, J_{C-F} = 8.0 Hz), 115.7 (d, J_{C-F} = 22.0 Hz), 58.0, 21.0 ppm; ^{19}F NMR (377 MHz, $CDCl_3$) δ -124.4 ppm; IR (thin film): 1501, 1426, 1220, 1078, 908, 803, 732 cm^{-1}; HRMS calculated for $C_{19}H_{18}N_2FS^+$ 325.1169, found 325.1167 $[M+H]^+$.

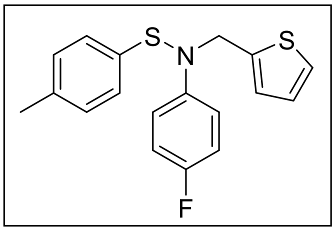

***N*-(4-Fluorophenyl)-*N*-(thiophen-2-ylmethyl)-*S*-(*p*-tolyl)thiohydroxylamine (3add):** The reaction was performed following the General Procedure with *N*-(thiophen-2-ylmethyl)-*S*-(*p*-tolyl)thiohydroxylamine (35.3 mg, 0.15 mmol), **2d** (42.0 mg, 0.3 mmol), Cy_2NMe (48.2 µL, 0.23 mmol), $Cu(TFA)_2$•H_2O (4.5 mg, 10 mol %), and **L3** (7.4 mg, 20 mol %) for 12 h. The crude product was purified by flash chromatography on silica gel (eluted with hexane) to give the product **3add** (30.1 mg, 61% yield) as a colorless oil. R_f = 0.4 (hexane); ^1H NMR (400 MHz, $CDCl_3$) δ 7.22 – 7.20 (m, 1H), 7.18 – 7.14 (m, 2H), 7.12 – 7.10 (m, 2H), 7.06 – 7.03 (m, 2H), 6.99 – 6.87 (m, 4H), 5.00 (s, 2H), 2.31 (s, 3H) ppm; ^{13}C NMR (100 MHz, $CDCl_3$) δ 158.6 (d, $J_{C\text{-}F}$ = 238.0 Hz), 145.4 (d, $J_{C\text{-}F}$ = 3.0 Hz), 141.5, 136.2, 135.8, 129.8, 126.8, 125.5, 124.9, 124.0, 117.6 (d, $J_{C\text{-}F}$ = 8.0 Hz), 115.6 (d, $J_{C\text{-}F}$ = 22.0 Hz), 55.8, 21.0 ppm; ^{19}F NMR (377 MHz, $CDCl_3$) δ -124.7 ppm; IR (thin film): 1499, 1444, 1400, 1222, 1111, 1064, 801, 756 cm^{-1}; HRMS calculated for $C_{18}H_{17}NFS_2^+$ 330.0781, found 330.0778 $[M+H]^+$.

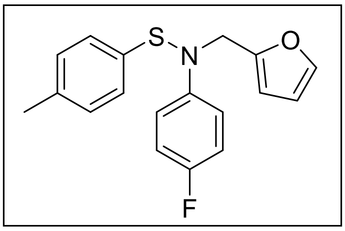

***N*-(4-Fluorophenyl)-*N*-(furan-2-ylmethyl)-*S*-(*p*-tolyl)thiohydroxylamine (3aed):** The reaction was performed following the General Procedure with *N*-(furan-2-ylmethyl)-*S*-(*p*-tolyl)thiohydroxylamine (32.9 mg, 0.15 mmol), **2d** (42.0 mg, 0.3 mmol), Cy_2NMe (48.2 µL, 0.23 mmol), $Cu(TFA)_2$•H_2O (4.5 mg, 10 mol %), and **L3** (7.4 mg, 20 mol %) for 12 h. The crude product was purified by flash chromatography on silica gel (eluted with hexane) to give the product **3aed** (28.7 mg, 61% yield) as a colorless oil. R_f = 0.4 (hexane); ^1H NMR (600 MHz, $CDCl_3$) δ 7.41 – 7.40 (m, 1H), 7.25 – 7.23 (m, 2H), 7.14 – 7.13 (m, 2H), 7.06 – 7.04 (m, 2H), 6.96 – 6.94 (m, 2H), 6.35 – 6.34 (m, 1H), 6.29 – 6.28 (m, 1H), 4.83 (s, 2H), 2.34 (s, 3H) ppm; ^{13}C NMR (150 MHz, $CDCl_3$) δ 158.2 (d, $J_{C\text{-}F}$ = 232.5 Hz), 151.7, 145.6 (d, $J_{C\text{-}F}$ = 9.0 Hz), 142.2, 136.2, 135.9, 129.8, 123.6, 117.5 (d, $J_{C\text{-}F}$ = 7.5 Hz), 115.5 (d, $J_{C\text{-}F}$ = 2.3 Hz), 110.4, 108.4, 53.5, 21.0 ppm; ^{19}F NMR (377 MHz, $CDCl_3$) δ -124.9 ppm; IR (thin film): 1502, 1441,1311, 1224, 1148, 922, 803, 739 cm^{-1}; HRMS calculated for $C_{18}H_{16}NFOS^+$ 313.0937, found 313.0936 $[M]^+$.

2.4 X-ray Structure of 3bn (CCDC 2142998)

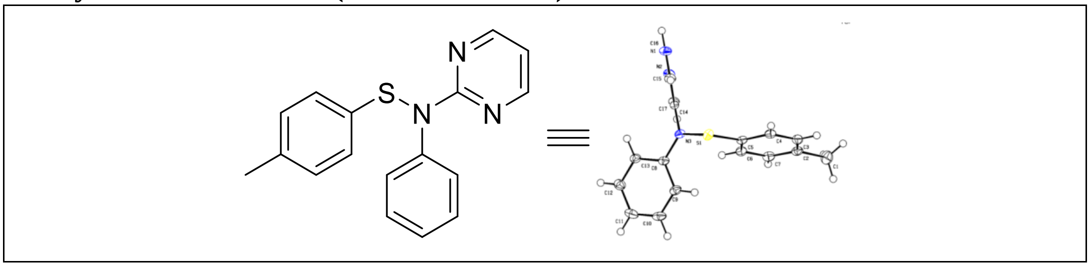

Single crystals of **3bn** were cultured by slow evaporation of suturated dichloromethane solution of **3bn** at room temperature. A suitable crystal of **3bn** was selected and placed on a **'Bruker APEX-II CCD'** diffractometer. The crystal was

kept at 100.0 K during data collection. Using Olex2[5], the structure was solved with the ShelXT[6] structure solution program using Intrinsic Phasing and refined with the ShelXL[7] refinement package using Least Squares minimisation.

Crystal Data for $C_{17}H_{15}N_3S$ (M=293.38 g/mol): monoclinic, space group P2$_1$/n (no. 14), a = 6.1285(2) Å, b = 20.1865(8) Å, c = 12.0240(4) Å, β = 97.994(2)°, V = 1473.07(9) Å^3, Z = 4, T = 100.00 K, μ(CuKα) = 1.908 mm^{-1}, $Dcalc$ = 1.323 g/cm^3, 14236 reflections measured (8.622° ≤ 2Θ ≤ 136.372°), 2690 unique (R_{int} = 0.0574, R_{sigma} = 0.0341) which were used in all calculations. The final R_1 was 0.0376 (I > 2σ(I)) and wR_2 was 0.1015 (all data).

Rotation frames were integrated using **SAINT**, producing a listing of unaveraged F^2 and σ(F^2) values. A total of 14236 reflections were measured over the ranges 8.622° ≤ 2Θ ≤ 136.372°, -7≤ h ≤ 7, -24≤ k ≤ 24, -14 ≤ l ≤ 11 yielding 2690 unique reflections (R_{int} = 0.0574). The intensity data were corrected for Lorentz and polarization effects and for absorption using SADABS (minimum and maximum transmission 0.504, 0.753). The structure was solved by direct methods – ShelXT. Refinement was by full-matrix least squares based on F^2 using ShelXL-2014. All reflections were used during refinement. The weighting scheme used was $w = 1/[\sigma^2(F_o^2) + (0.0401P)^2 + 0.3189P]$ where $P = (F_o^2 + 2F_c^2)/3$. Non-hydrogen atoms were refined anisotropically and hydrogen atoms were refined using a riding model. Refinement converged to R_1=0.0376 and $wR_2 = 0.1003$ for 14236 observed reflections for which $F > 4\sigma(F)$ and R_1=0.0394 and $wR_2 = 0.1015$ and GOF =1.047 for all 2690 unique, non-zero reflections and 191 variables. The maximum Δ/σ in the final cycle of least squares was 0.000 and the two most prominent peaks in the final difference Fourier were +0.42 and -0.41 e/Å3.

Supplementary Table 6 lists the cell information, data collection parameters, and refinement data. Final positional and equivalent isotropic thermal parameters are given in Supplementary Table 7 and 8. Anisotropic thermal parameters are in Supplementary Table 9. Supplementary Table 10 and 11 list the bond distances and bond angles. Supplementary Figure 1 is an ORTEP representation of the molecule with 50% probability thermal ellipsoids displayed.

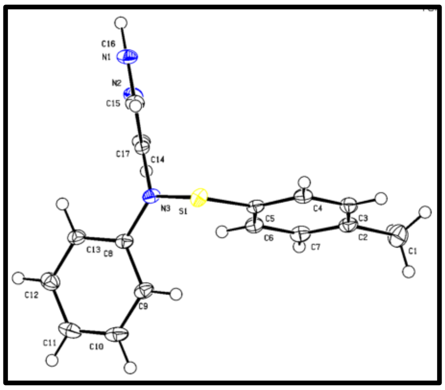

Supplementary Figure 1. ORTEP Drawing of **3bn** with 50% Thermal Ellipsoids

Supplementary Table 6. Summary of Structure Determination of Compound **3bn**.

Empirical formula	$C_{17}H_{15}N_3S$
Formula weight	293.38
Temperature/K	100.00
Crystal system	monoclinic
Space group	$P2_1/n$
a/Å	6.1285(2)
b/Å	20.1865(8)
c/Å	12.0240(4)
α/°	90
β/°	97.994(2)
γ/°	90
Volume/Å^3	1473.07(9)
Z	4
ρ_{calc}g/cm^3	1.323
μ/mm^{-1}	1.908
F(000)	616.0
Crystal size/mm^3	0.3 × 0.28 × 0.04
Radiation	CuKα (λ = 1.54178)
2Θ range for data collection/°	8.622 to 136.372
Index ranges	$-7 \leq h \leq 7$, $-24 \leq k \leq 24$, $-14 \leq l \leq 11$
Reflections collected	14236
Independent reflections	2690 [R_{int} = 0.0574, R_{sigma} = 0.0341]
Data/restraints/parameters	2690/0/191
Goodness-of-fit on F^2	1.047
Final R indexes [I>=2σ (I)]	R_1 = 0.0376, wR_2 = 0.1003
Final R indexes [all data]	R_1 = 0.0394, wR_2 = 0.1015
Largest diff. peak/hole / e Å^{-3}	0.42/-0.41

Supplementary Table 7. Refined Positional Parameters for Compound **3bn.**

Atom	x	y	z	U(eq)
S1	7517.6(6)	7430.6(2)	5153.5(3)	18.75(14)
N1	9702(2)	8615.1(7)	2460.3(11)	22.8(3)
N2	5930(2)	8681.7(7)	1655.6(10)	20.4(3)
N3	6279(2)	8129.0(6)	4605.8(10)	17.1(3)
C1	1918(3)	5159.6(9)	2981.4(15)	30.4(4)
C2	3307(3)	5731.8(8)	3479.8(12)	20.9(3)
C3	5463(3)	5635.2(8)	4005.6(12)	20.6(3)
C4	6739(2)	6157.8(8)	4480.9(12)	18.7(3)
C5	5850(2)	6794.7(7)	4446.8(11)	17.0(3)

C6	3720(2)	6904.1(8)	3911.7(12)	19.0(3)
C7	2470(2)	6373.8(8)	3432.4(12)	20.6(3)
C8	4622(2)	8456.0(7)	5114.8(12)	16.5(3)
C9	3531(3)	8133.1(8)	5908.8(12)	20.8(3)
C10	1914(3)	8463.4(9)	6394.9(13)	25.4(4)
C11	1344(3)	9111.0(9)	6102.6(13)	26.4(4)
C12	2436(3)	9433.3(8)	5324.0(13)	23.6(3)
C13	4075(2)	9113.7(8)	4837.5(12)	19.4(3)
C14	6871(2)	8349.4(7)	3563.2(12)	15.3(3)
C15	9074(2)	8421.8(8)	3430.9(12)	19.1(3)
C16	8082(3)	8737.1(8)	1625.9(12)	19.8(3)
C17	5326(2)	8484.8(7)	2633.5(12)	18.4(3)

Supplementary Table 8. Positional Parameters for Hydrogens in Compound **3bn**

Atom	x	y	z	U(eq)
H1A	1091.95	4974.01	3549.99	46
H1B	2874.29	4817.2	2731.43	46
H1C	885.8	5315.86	2339.05	46
H3	6070.96	5201.55	4038.58	25
H4	8207.87	6082.23	4827.08	22
H6	3118.82	7338.63	3873.13	23
H7	1017.85	6452.06	3065.83	25
H9	3897.44	7688.3	6115.02	25
H10	1187.46	8241.93	6936.25	30
H11	220.96	9330.99	6431.06	32
H12	2059.49	9877.79	5120.67	28
H13	4826.95	9342.97	4314.58	23
H15	10171.82	8330.79	4050.95	23
H16	8511.87	8878.99	935.01	24
H17	3803.84	8436.87	2691.19	22

Supplementary Table 9. Refined Thermal Parameters (U's) for Compound **3bn**.

Atom	U_{11}	U_{22}	U_{33}	U_{23}	U_{13}	U_{12}
S1	14.5(2)	18.8(2)	21.5(2)	3.26(13)	-2.56(15)	-1.55(12)
N1	15.2(6)	30.9(7)	23.5(6)	-1.0(5)	7.3(5)	-1.6(5)
N2	17.3(6)	25.9(7)	18.4(6)	-0.1(5)	3.3(5)	3.0(5)
N3	13.9(6)	19.5(6)	18.3(6)	4.4(5)	3.6(5)	0.5(5)
C1	28.1(9)	29.9(9)	31.8(9)	-4.7(7)	-0.8(7)	-6.9(7)
C2	21.9(8)	25.9(8)	15.6(7)	-0.2(6)	5.1(6)	-5.2(6)
C3	21.5(7)	21.8(7)	19.3(7)	1.0(6)	5.8(6)	0.1(6)
C4	14.3(7)	24.1(8)	17.9(7)	2.5(6)	3.3(5)	0.7(6)

C5	16.1(7)	21.5(7)	14.0(7)	2.0(5)	3.9(5)	-2.7(6)
C6	15.9(7)	22.3(7)	18.7(7)	1.5(6)	2.4(6)	-0.4(6)
C7	14.6(7)	28.8(8)	17.9(7)	1.7(6)	0.3(6)	-2.1(6)
C8	10.8(7)	23.7(7)	14.6(7)	-1.6(5)	0.2(5)	-3.4(6)
C9	20.4(7)	26.3(8)	15.7(7)	1.4(6)	2.0(6)	-5.7(6)
C10	21.0(8)	40.5(9)	15.4(7)	-1.3(6)	5.6(6)	-7.2(7)
C11	19.1(8)	39.7(9)	21.5(7)	-7.1(7)	6.7(6)	0.6(7)
C12	19.5(8)	26.7(8)	24.7(8)	-3.6(6)	3.5(6)	0.6(6)
C13	15.4(7)	23.3(7)	19.8(7)	-0.5(6)	4.1(6)	-3.1(6)
C14	13.7(7)	14.8(7)	18.2(7)	-0.6(5)	4.6(5)	-1.1(5)
C15	12.4(7)	25.0(8)	19.3(7)	-0.8(6)	0.6(6)	-0.6(6)
C16	19.5(7)	21.6(7)	19.8(7)	-1.5(6)	7.9(6)	0.3(6)
C17	11.7(6)	23.1(7)	21.1(7)	-0.3(6)	4.3(6)	0.2(6)

Supplementary Table 10. Bond Distances in Compound **3bn** (Å)

Atom	Atom	Length/Å	Atom	Atom	Length/Å
S1	N3	1.6906(12)	C4	C5	1.395(2)
S1	C5	1.7811(15)	C5	C6	1.390(2)
N1	C15	1.337(2)	C6	C7	1.393(2)
N1	C16	1.333(2)	C8	C9	1.400(2)
N2	C16	1.329(2)	C8	C13	1.398(2)
N2	C17	1.3411(19)	C9	C10	1.389(2)
N3	C8	1.4196(19)	C10	C11	1.386(3)
N3	C14	1.4238(18)	C11	C12	1.386(2)
C1	C2	1.509(2)	C12	C13	1.389(2)
C2	C3	1.397(2)	C14	C15	1.389(2)
C2	C7	1.392(2)	C14	C17	1.388(2)
C3	C4	1.388(2)			

Supplementary Table 11. Bond Angles in Compound **3bn** (°)

Atom	Atom	Atom	Angle/˚	Atom	Atom	Atom	Angle/˚
N3	S1	C5	102.62(7)	C2	C7	C6	121.34(14)
C16	N1	C15	115.86(13)	C9	C8	N3	121.02(14)
C16	N2	C17	116.39(13)	C13	C8	N3	120.06(12)
C8	N3	S1	121.95(10)	C13	C8	C9	118.91(13)
C8	N3	C14	121.28(12)	C10	C9	C8	119.97(15)
C14	N3	S1	116.63(9)	C11	C10	C9	121.03(14)
C3	C2	C1	121.26(15)	C10	C11	C12	119.05(15)
C7	C2	C1	120.81(15)	C11	C12	C13	120.77(15)
C7	C2	C3	117.93(14)	C12	C13	C8	120.25(14)
C4	C3	C2	121.56(15)	C15	C14	N3	120.28(13)
C3	C4	C5	119.54(14)	C17	C14	N3	122.83(13)

C4	C5	S1	117.02(11)	C17	C14	C15	116.87(13)
C6	C5	S1	123.08(12)	N1	C15	C14	122.21(14)
C6	C5	C4	119.86(14)	N2	C16	N1	127.05(14)
C5	C6	C7	119.74(14)	N2	C17	C14	121.60(13

2.5 Synthetic Applications

2.5.1 Further transformations of 3ba

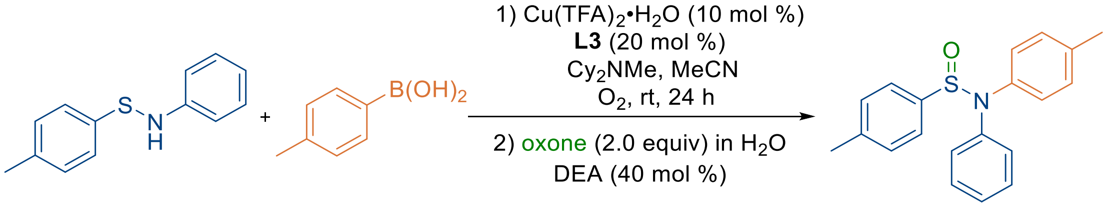

1b **2a** **4**, 48%

Procedure: To an oven-dried microwave vial equipped with a stir bar was added $Cu(TFA)_2$•H_2O (4.5 mg, 10 mol %), **L3** (7.4 mg, 20 mol %), 3-((*p*-tolylthio)amino)benzene-1-ylium (**1b**) (32.3 mg, 0.15 mmol), and *p*-tolylboronic acid (**2a**) (40.8 mg, 0.3 mmol). Then, Cy_2NMe (48.2 μL, 0.23 mmol) and MeCN (0.5 mL) was added via syringe under an air atmosphere. The vial was sealed with a septum, and refilled by a dioxygen balloon for 3 min. The solution was stirred at room temperature for 24 h under an O_2 atmosphere. Then, the vial was opened to air, and DEA (40 mol %, 6.2 μL) and oxone (2.0 equiv, 103.9 mg) in H_2O (0.5 mL) was added via syringe. The reaction was stirred at room temperature for additional 2 h. Upon completion, the reaction was quenched by saturated $Na_2S_2O_3$ solution (10 mL), and extracted by EtOAc (10 mL×3). The combined organic layers were extracted by brine (10 mL), dried over anhydrous Na_2SO_4, filtered, and concentrated under reduced pressure. The crude product was purified by column chromatography on silica gel (eluted with EtOAc:hexane = 1:2) to give the product **4** (23.1 mg, 48% yield for two steps) as a colorless solid. R_f = 0.2 (EtOAc:hexane = 1:2); m.p. = 144.6–146.3 °C; ^1H NMR (600 MHz, DMSO-d_6) δ 8.50 – 8.46 (m, 1H), 7.50 (d, J = 8.4 Hz, 2H), 7.43 (d, J = 8.6 Hz, 2H), 7.33 (d, J = 7.8 Hz, 2H), 7.09 (d, J = 7.8 Hz, 2H), 7.00 – 7.04 (m, 4H), 2.32 (s, 3H), 2.24 (s, 3H) ppm; ^{13}C NMR (151 MHz, DMSO-d_6) δ 147.5, 143.3, 140.5, 138.9, 133.6, 130.8, 129.8, 129.7, 126.7, 124.1, 119.6, 114.7, 20.8, 20.4 ppm; IR (thin film): 1588, 1513, 1325, 1176, 1079, 1005, 998, 811, 713, 641 cm^{-1}; HRMS calculated for $C_{20}H_{20}NOS^+$ 322.1260, found 322.1257 $[M+H]^+$.

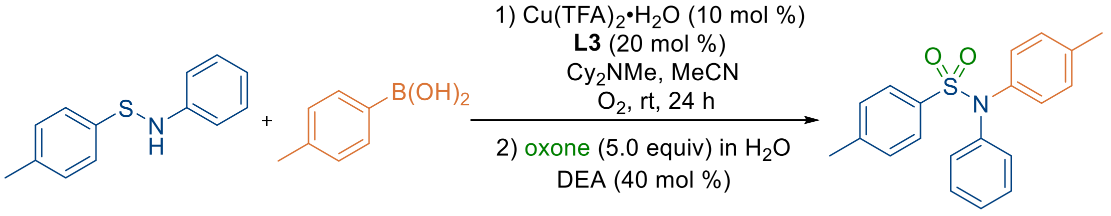

1b **2a** **5**, 45%

Procedure: To an oven-dried microwave vial equipped with a stir bar was added $Cu(TFA)_2 \cdot H_2O$ (4.5 mg, 10 mol %), **L3** (7.4 mg, 20 mol %), 3-((*p*-tolylthio)amino)benzene-1-ylium (**1b**) (32.3 mg, 0.15 mmol), and *p*-tolylboronic acid (**2a**) (40.8 mg, 0.3 mmol). Then, Cy_2NMe (48.2 μL, 0.23 mmol) and MeCN (0.5 mL) was added via syringe under an air atmosphere. The vial was sealed with a septum, and refilled by a dioxygen balloon for 3 min. The solution was stirred at room temperature for 24 h under an O_2 atmosphere. Then, the vial was opened to air, and DEA (40 mol %, 6.2 μL) and oxone (5.0 equiv, 259.7 mg) in H_2O (0.5 mL) was added via syringe. The reaction was stirred at room temperature for additional 2 h. Upon completion, the reaction was quenched by saturated $Na_2S_2O_3$ solution (10 mL), and extracted by EtOAc (10.0 mL×3). The combined organic layers were extracted by brine (10 mL), dried over anhydrous Na_2SO_4, filtered, and concentrated under reduced pressure. The crude product was purified by column chromatography on silica gel (eluted with EtOAc:hexane = 1:5) to give the product **5** (22.8 mg, 45% yield for two steps) as a colorless solid. R_f = 0.3 (EtOAc:hexane = 1:5); ^{1}H NMR (400 MHz, $CDCl_3$) δ 7.62 – 7.55 (m, 2H), 7.31 – 7.19 (m, 7H), 7.18 – 7.13 (m, 2H), 7.12 – 7.07 (m, 2H), 2.40 (s, 3H), 2.30 (s, 3H) ppm; ^{13}C NMR (100 MHz, $CDCl_3$) δ 143.6, 141.9, 139.0, 137.8, 137.7, 130.0, 129.6, 129.3, 128.5, 128.1, 127.9, 127.3, 21.7, 21.1 ppm; IR (thin film): 1348, 1156, 1089, 947, 903, 812, 755, 720, 710, 683 cm^{-1}. The spectroscopic data match the previously reported data.[8]

2.5.2 Late-stage modification of sulfenamide-analogues of drugs

Synthesis of 7: To an oven-dried Schlenk flask equipped with a stir bar was added sulfacetamide **6** (214.2 mg, 1.0 mmol), TEA (0.14 mL, 1.0 equiv) and anhydrous THF (5.0 mL) under an argon atmosphere. After the reaction mixture was stirred for 30 min at 0 °C, *p*-tolyl hypochlorothioite (190.3 mg, 1.2 equiv) was added dropwise into the solution. After stirring for additional 2 h at room temperature, the reaction was filtered. The organic layer was separated and evaporated under reduced pressure. The crude compound was purified by flash chromatography on silica gel (eluted with EtOAc:hexane = 1:2) to give the product **7** (252.3 mg, 75% yield) as a light yellow solid. R_f = 0.4 (EtOAc:hexane = 1:1); m.p. = 137.3–138.6 °C; ^{1}H NMR (400 MHz, DMSO-d_6) δ 11.77 (s, 1H), 8.60 (s, 1H), 7.66 (d, J = 9.0 Hz, 2H), 7.22 – 6.96 (m, 6H), 2.17 (s, 3H), 1.82 (s, 3H) ppm; ^{13}C NMR (100 MHz, DMSO-d_6) δ 169.0, 152.9, 136.8, 135.9, 130.3, 130.1, 129.4, 123.7, 114.3, 23.7, 21.0 ppm; IR (thin film): 3318, 3196, 2971, 1708, 1593, 1449, 1148, 1091, 860 cm^{-1}; HRMS calculated for $C_{15}H_{17}N_2O_3S_2^+$ 337.0675, found 337.0677 $[M+H]^+$.

Synthesis of 8: To an oven-dried microwave vial equipped with a stir bar was added $Cu(TFA)_2 \cdot H_2O$ (4.5 mg, 10 mol %), **L3** (7.4 mg, 20 mol %), *N*-((4-((*p*-tolylthio)amino)phenyl)sulfonyl)acetamide (**7**) (50.4 mg, 0.15 mmol), and *p*-tolylboronic acid (**2a**) (40.8 mg, 0.3 mmol). Then, Cy_2NMe (48.2 μL, 0.23 mmol) and MeCN:EtOH (*v*/*v* 4:1, 0.5 mL in total) was added via syringe. The vial was sealed with a septum, and refilled by an O_2 balloon for 3 min. The solution was stirred at

room temperature for 24 h under an O_2 atmosphere. The crude product was purified by flash chromatography on silica gel (eluted with EtOAc:hexane = 1:2) to give the product **8** (33.2 mg, 52% yield) as a colorless solid. R_f = 0.3 (EtOAc:hexane = 1:2); m.p. = 126.1–128.3 °C; ^{1}H NMR (400 MHz, DMSO-d_6) δ 12.43 (s, 1H), 8.43 (d, J = 9.0 Hz, 2H), 8.16 (d, J = 9.0 Hz, 2H), 7.54 – 7.30 (m, 4H), 7.17 (dd, J = 17.8, 8.0 Hz, 4H), 2.27 (s, 3H), 2.26 (s, 3H), 1.95 (s, 3H) ppm; ^{13}C NMR (100 MHz, DMSO-d_6) δ 169.6, 150.8, 145.0, 138.0, 137.4, 133.1, 131.7, 131.5, 130.5, 129.7, 128.6, 125.0, 118.8, 23.8, 21.0, 20.9 ppm; IR (thin film): 3359, 1720, 1591, 1493, 1427, 1143, 1084, 856, 821, 802 cm^{-1}; HRMS calculated for $C_{22}H_{23}N_2O_3S_2^+$ 427.1145, found 427.1140 $[M+H]^+$.

Synthesis of 10: To an oven-dried Schlenk flask equipped with a stir bar was added methyl 3-hydroxy-4-nitrobenzoate **9** (591.3 mg, 3.0 mmol), K_2CO_3 (648.9 mg, 6.0 mmol), and DMF (10.0 mL), and then 1-bromobutane (0.97 mL, 9.0 mmol) was added into the mixture. After the reaction mixture was heated at 90 ^{o}C for 12 h, it was cooled to room temperature. And 100 mL H_2O was added, and the mixture was extracted with EtOAc (60.0 mL×3). The combined organic layers were dried over anhydrous Na_2SO_4, and evaporated under reduced pressure. The crude residue was purified by flash chromatography on silica gel (eluted with EtOAc:hexane = 1:4) to give the product **10** (661.0 mg, 87% yield) as a yellow oil. R_f = 0.4 (EtOAc:hexane = 1:30); ^{1}H NMR (400 MHz, $CDCl_3$) δ 7.79 (d, J = 8.4 Hz, 1H), 7.73 (s, 1H), 7.65 (dd, J = 8.4, 1.6 Hz, 1H), 4.17 (t, J = 6.4 Hz, 2H), 3.96 (s, 3H), 1.90 – 1.71 (m, 2H), 1.52 (q, J = 7.4 Hz, 2H), 0.98 (t, J = 7.4 Hz, 3H) ppm; ^{13}C NMR (100 MHz, $CDCl_3$) δ 165.2, 151.9, 142.5, 134.7, 125.1, 121.0, 115.4, 69.6, 52.7, 30.8, 19.0, 13.7 ppm; IR (thin film): 2960, 1727, 1608, 1529, 1287, 1235, 1107, 839, 744 cm^{-1}; HRMS calculated for $C_{12}H_{16}NO_5^+$ 254.1023, found 254.1027 $[M+H]^+$.

Synthesis of 11: To an oven-dried round-bottom flask equipped with a stir bar was added compound **10** (633.1 mg, 2.5 mmol), MeOH (5.0 mL), THF (5.0 mL), and 10% NaOH aqueous solution (10.0 mL), and the reaction mixture was stirred for 12 h at room temperature. After the reaction was completed, the mixture was concentrated, and the resulting residue was acidified with 1.0 M HCl (30.0 mL), and then extracted with EtOAc (30.0 mL×3). The organic layers were washed with 1.0 M HCl and brine, dried over anhydrous Na_2SO_4, and evaporated under reduced pressure to give the product **11** (549.7 mg, 92% yield) as a yellow solid; R_f = 0.2 (DCM:MeOH = 10:1); m.p. = 171.5–173.3 °C; ^{1}H NMR (400 MHz, Methanol-d_4) δ 7.81 (d, J = 8.4 Hz, 2H), 7.67 (d, J = 8.4 Hz, 1H), 4.19 (t, J = 6.4 Hz, 2H), 1.94 – 1.68 (m, 2H), 1.53 (q, J = 7.4 Hz, 2H), 1.00 (t, J = 7.4 Hz, 3H) ppm; ^{13}C NMR (100 MHz, methanol-d_4) δ 166.3, 151.4, 142.7, 135.3, 124.5, 121.1, 115.2, 69.2, 30.7, 18.7, 12.7 ppm; IR (thin film): 2960, 1665, 1607, 1526, 1349, 1254, 1058, 979, 747 cm^{-1}; HRMS calculated for $C_{11}H_{12}NO_5^-$ 238.0721, found 238.0715 $[M-H]^-$.

Synthesis of 12: To an oven-dried round-bottom flask equipped with a stir bar was added compound **11** (478.5 mg, 2.0 mmol) and 3 drops of DMF in anhydrous DCM

(15.0 mL) at 0 °C, and $(COCl)_2$ (0.2 mL, 2.4 mmol) was slowly added by drops. The reaction mixture was stirred at room temperature for 1 h. The solvent was then evaporated under reduced pressure, and the resulting acyl chloride was obtained and used without further purification. To another an oven-dried Schlenk flask equipped with a stir bar was added DEAE (0.3 mL, 2.2 mmol), TEA (0.42 mL, 3.0 mmol), and anhydrous DCM (10.0 mL) under an argon atmosphere, and then the acyl chloride and DMAP (48.9 mg, 0.4 mmol) were added. The reaction mixture was stirred at room temperature for 2 h. Upon completion of the reaction, the reaction was quenched with water (20.0 mL). The organic layer was separated, and the aqueous layer was extracted with DCM (15.0 mL×3). The combined organic layers were dried over anhydrous $MgSO_4$, and evaporated under reduced pressure. The crude residue was purified by flash chromatography on silica gel (eluted with DCM:MeOH = 50:1) to give the product **12** (514.4 mg, 76% yield) as a yellow oil; R_f = 0.2 (EtOAc:hexane = 1:2); ^{1}H NMR (600 MHz, $CDCl_3$) δ 7.70 (dd, *J* = 8.3, 1.2 Hz, 1H), 7.65 (d, *J* = 1.6 Hz, 1H), 7.57 (dt, *J* = 8.3, 1.6 Hz, 1H), 4.33 (t, *J* = 6.2 Hz, 2H), 4.08 (t, *J* = 6.4 Hz, 2H), 2.77 (t, *J* = 6.2 Hz, 2H), 2.54 (q, *J* = 7.2 Hz, 4H), 1.77 – 1.69 (m, 2H), 1.44 (q, *J* = 7.6 Hz, 2H), 0.98 (dd, *J* = 7.8, 6.4 Hz, 6H), 0.89 (t, *J* = 7.4 Hz, 3H) ppm; ^{13}C NMR (150 MHz, $CDCl_3$) δ 164.6, 151.8, 142.5, 134.9, 125.0, 121.0, 115.4, 69.5, 64.3, 51.0, 47.8, 30.8, 19.0, 13.6, 12.1 ppm; IR (thin film): 2966, 1724, 1609, 1529, 1287, 1232, 1015, 735 cm^{-1}; HRMS calculated for $C_{17}H_{27}N_2O_5^+$ 339.1914, found 339.1915 $[M+H]^+$.

Synthesis of 13: To a flame-dried round-bottom flask, **12** (507.6 mg, 1.5 mmol) dissolved in anhydrous EtOAc (10.0 mL) and 10 wt% wet Pd/C (53.2 mg, 0.5 mmol) were added. The flask was equipped with a H_2 balloon (1 atm) and the reaction mixture was stirred at room temperature for 12 h. Upon completion of the reaction, the reaction mixture was filtered over Celite and the solvent was evaporated under reduced pressure. Product **13** (411.7 mg, 89% yield) was obtained after concentrated as a yellow oil. R_f = 0.2 (DCM:MeOH = 10:1); ^{1}H NMR (600 MHz, $CDCl_3$) δ 7.51 (dd, *J* = 8.2, 1.8 Hz, 1H), 7.43 (d, *J* = 1.8 Hz, 1H), 6.63 (d, *J* = 8.2 Hz, 1H), 4.32 (t, *J* = 6.2 Hz, 2H), 4.25 (s, 2H), 4.02 (t, *J* = 6.4 Hz, 2H), 2.82 (t, *J* = 6.4 Hz, 2H), 2.61 (q, *J* = 7.2 Hz, 4H), 1.82 – 1.74 (m, 2H), 1.51 – 1.45 (m, 2H), 1.05 (t, *J* = 7.2 Hz, 6H), 0.96 (t, *J* = 7.4 Hz, 3H) ppm; ^{13}C NMR (100 MHz, $CDCl_3$) δ 166.9, 145.5, 141.3, 123.9, 119.4, 113.0, 112.1, 68.1, 62.8, 51.0, 47.8, 31.3, 19.3, 13.9, 12.0 ppm; IR (thin film): 2924, 1716, 1609, 1502, 1284, 1222, 805, 763 cm^{-1}; HRMS calculated for $C_{17}H_{29}N_2O_3^+$ 309.2173, found 309.2175 $[M+H]^+$.

Synthesis of 14: To an oven-dried Schlenk flask equipped with a stir bar was added sulfacetamide **13** (308.4 mg, 1.0 mmol), TEA (0.14 mL, 1.0 equiv), and anhydrous THF (5.0 mL) under an argon atmosphere. After the reaction mixture was stirred for 30 min at 0 °C, *p*-tolyl hypochlorothioite (190.3 mg, 1.2 equiv) was added dropwise into the solution. After stirring for additional 2 h at room temperature, the reaction was filtered. The organic layer was separated and evaporated under reduced pressure. The crude reside was purified by flash chromatography on silica gel (eluted with

EtOAc:hexane = 1:2) to give the product **14** (305.7 mg, 71% yield) as a light yellow oil; R_f = 0.2 (EtOAc:hexane = 1:2); ^{1}H NMR (600 MHz, $CDCl_3$) δ 7.58 (dd, *J* = 8.4, 1.8 Hz, 1H), 7.47 (d, *J* = 1.8 Hz, 1H), 7.23 (d, *J* = 8.4 Hz, 1H), 7.13 – 7.09 (m, 4H), 6.07 (s, 1H), 4.35 (t, *J* = 6.2 Hz, 2H), 4.09 (t, *J* = 6.4 Hz, 2H), 2.85 (t, *J* = 6.2 Hz, 2H), 2.64 (q, *J* = 7.2 Hz, 4H), 2.29 (s, 3H), 1.87 – 1.77 (m, 2H), 1.56 – 1.47 (m, 2H), 1.07 (t, *J* = 7.2 Hz, 6H), 1.00 (t, *J* = 7.4 Hz, 3H); ^{13}C NMR (150 MHz, $CDCl_3$) δ 166.7, 146.6, 141.2, 136.8, 135.9, 129.8, 123.8, 123.4, 121.5, 112.3, 111.6, 68.5, 63.0, 51.0, 47.8, 31.3, 21.0, 19.3, 13.9, 12.0 ppm; IR (thin film): 2971, 1703, 1593, 1287, 1085, 1046, 803, 683 cm^{-1}; HRMS calculated for $C_{24}H_{35}N_2O_3S^+$ 431.2363, found 431.2367 $[M+H]^+$.

Synthesis of 15: To an oven-dried microwave vial equipped with a stir bar was added $Cu(TFA)_2$•H_2O (4.5 mg, 10 mol %), **L3** (7.4 mg, 20 mol %), 2-(diethylamino)ethyl 3-butoxy-4-((*p*-tolylthio)amino)benzoate (**14**) (50.4 mg, 0.15 mmol), and *p*-tolylboronic acid (**2a**) (40.8 mg, 0.3 mmol). Then, Cy_2NMe (48.2 μL, 0.23 mmol) and MeCN (0.5 mL) was added via syringe under an air atmosphere. The vial was sealed with a septum, and refilled by an O_2 balloon for 3 min. The solution was stirred at room temperature for 24 h under an O_2 atmosphere. The crude product was purified by flash chromatography on silica gel (eluted with EtOAc:hexane = 1:2) to give the product **15** (47.6 mg, 61% yield) as a colorless oil. R_f = 0.2 (EtOAc:hexane = 1:2); ^{1}H NMR (400 MHz, $CDCl_3$) *δ* 7.62 – 7.58 (m, 1H), 7.57 (d, *J* = 1.8 Hz, 1H), 7.45 (d, *J* = 8.2 Hz, 1H), 7.22 (d, *J* = 8.2 Hz, 2H), 7.09 (d, *J* = 8.2 Hz, 2H), 6.99 – 6.90 (m, 4H), 4.49 – 4.35 (m, 2H), 3.93 (t, *J* = 6.4 Hz, 2H), 2.98 – 2.83 (m, 2H), 2.79 – 2.58 (m, 4H), 2.30 (s, 3H), 2.26 (s, 3H), 1.53 – 1.44 (m, 2H), 1.35 – 1.21 (m, 2H), 1.20 – 1.09 (m, 6H), 0.80 (t, *J* = 7.4 Hz, 3H) ppm; ^{13}C NMR (100 MHz, $CDCl_3$) *δ* 166.4, 153.9, 147.4, 141.1, 137.9, 135.6, 130.5, 129.7, 129.3, 128.5, 128.0, 123.2, 122.7, 117.5, 114.0, 68.1, 63.6, 51.1, 47.8, 31.2, 29.8, 21.1, 20.6, 19.0, 13.8, 12.2 ppm; IR (thin film): 2963, 2349, 1703, 1598, 1515, 1268, 1219, 1105, 733, 705 cm^{-1}; HRMS calculated for $C_{31}H_{41}N_2O_3S^+$ 521.2832, found 521.2827 $[M+H]^+$.

3.Supplementary Discussion

3.1 Mechanistic Studies

3.1.1 UV/Vis-Absorption Spectra of the Reaction Components.

All the samples were used freshly for spectra measurement. The absorption spectrum of the solution was recorded by Perkin-Elmer model Lambda 365 UV-Vis spectrophotometer using a 1.0 cm quartz cell to add 1.0 mL of the sample.

L3 in MeCN (8 mM): In a dry box, **L3** (9.9 mg, 0.04 mmol) was dissolved in dry MeCN (5.0 mL), and the UV-Vis spectrum was measured immediately.

1a in MeCN (8 mM): In a dry box, **1a** (8.8 mg, 0.04 mmol) was dissolved in dry MeCN (5.0 mL), and the UV-Vis spectrum was measured immediately.

2a in MeCN (8 mM): In a dry box, **2a** (5.4 mg, 0.04 mmol) was dissolved in dry MeCN (5.0 mL), and the UV-Vis spectrum was measured immediately.

$Cu(TFA)_2$•H_2O and L3 in MeCN (4 mM): In a dry box, **L3** (9.9 mg, 0.04 mmol), and $Cu(TFA)_2$•H_2O (5.8 mg, 0.02 mmol) were dissolved in dry MeCN (5.0 mL). The UV-Vis spectrum was measured immediately.

Reaction mixture (0.4 mM): To an oven-dried microwave vial equipped with a stir bar was added $Cu(TFA)_2$•H_2O (4.5 mg, 10 mol %), **L3** (7.4 mg, 20 mol %), *S*-(4-fluorophenyl)-*N*-(*p*-tolyl)thiohydroxylamine (**1a**) (35.0 mg, 0.15 mmol), and *p*-tolylboronic acid (**2a**) (40.8 mg, 0.3 mmol). Then, Cy_2NMe (48.2 μL, 0.23 mmol) and MeCN (0.5 mL) was added via syringe. The vial was sealed with a septum, and refilled by an O_2 balloon for 3 min. The solution was then stirred at room temperature for 24 h under an O_2 atmosphere. Upon completion of the reaction, the concentration of copper was dilute to 0.4 mM in a dry box, and the UV-Vis spectrum was measured immediately.

3.1.2 Kinetic Studies

Procedure by using ligand L1: To an oven-dried microwave vial equipped with a stir bar was added $Cu(TFA)_2$•H_2O (4.5 mg, 10 mol %), **L1** (7.6 mg, 20 mol %), *S*-(4-fluorophenyl)-*N*-(*p*-tolyl)thiohydroxylamine (**1a**) (35.0 mg, 0.15 mmol), and *p*-tolylboronic acid (**2a**) (40.8 mg, 0.3 mmol). Then, Cy_2NMe (48.2 μL, 0.23 mmol) and MeCN (0.5 mL) was added via syringe. The vial was sealed with a septum, and refilled by an O_2 balloon for 3 min. The solution was then stirred at room temperature under an O_2 atmosphere. The consumption of **1a** and formation of **3aa** was monitored by ^{19}F NMR using 0.1 mmol $PhCF_3$ (12.0 μL) as internal standard.

Procedure by using ligand L2: To an oven-dried microwave vial equipped with a stir bar was added $Cu(TFA)_2$•H_2O (4.5 mg, 10 mol %), **L2** (6.5 mg, 20 mol %), *S*-(4-fluorophenyl)-*N*-(*p*-tolyl)thiohydroxylamine (**1a**) (35.0 mg, 0.15 mmol) and *p*-tolylboronic acid (**2a**) (40.8 mg, 0.3 mmol). Then, Cy_2NMe (48.2 μL, 0.23 mmol) and MeCN (0.5 mL) was added via syringe. The vial was sealed with a septum, and refilled by an O_2 balloon for 3 min. The solution was then stirred at room temperature under an O_2 atmosphere. The consumption of **1a** and formation of **3aa** was monitored by ^{19}F NMR using 0.1 mmol $PhCF_3$ (12.0 μL) as internal standard.

Procedure by using ligand L3: To an oven-dried microwave vial equipped with a stir bar was added $Cu(TFA)_2$•H_2O (4.5 mg, 10 mol %), **L3** (7.4 mg, 20 mol %), *S*-(4-fluorophenyl)-*N*-(*p*-tolyl)thiohydroxylamine (**1a**) (35.0 mg, 0.15 mmol) and *p*-tolylboronic acid (**2a**) (40.8 mg, 0.3 mmol). Then, Cy_2NMe (48.2 μL, 0.23 mmol) and MeCN (0.5 mL) was added via syringe. The vial was sealed with a septum, and refilled by an O_2 balloon for 3 min. The solution was then stirred at room temperature under an O_2 atmosphere. The consumption of **1a** and formation of **3aa** was monitored by ^{19}F NMR using 0.1 mmol $PhCF_3$ (12.0 μL) as internal standard.

3.1.3 EPR Spectroscopy

X-band (9.36 GHz) CW EPR spectra were recorded on a Bruker EleXsys E500

spectrometer equipped with a super-high Q resonator (ER4122SHQE). Cryogenic temperatures were achieved and controlled using an ESR900 liquid helium cryostat in conjunction with a temperature controller (Oxford Instruments MercuryiTC) and a gas flow controller. CW EPR spectra of frozen solution samples were recorded at 50 K by using 0.02 mW power (no saturation) under slow-passage conditions. The spectrometer settings were as follows: conversion time of 40 ms, modulation amplitude of 0.5 mT and modulation frequency of 100 kHz. Simulations of the CW spectra were performed using EasySpin 5.2.35 toolbox[9,10] within the Matlab 2014a software suite (The Mathworks Inc., Natick, MA).

EPR Sample preparations

CuTc+L3+Cy$_2$NMe+toluene+2a (N$_2$, 2 h): To an oven-dried microwave vial equipped with a stir bar was added CuTc (4.8 mg, 0.025 mmol), **L3** (12.4 mg, 0.05 mmol) and *p*-tolylboronic acid (**2a**) (68.0 mg, 0.5 mmol). Then, Cy$_2$NMe (80.3 μL, 0.38 mmol) and toluene (0.5 mL) was added via syringe. The vial was sealed with a septum in the glove box. The solution was then stirred at room temperature for 2 h under an N$_2$ atmosphere. After that, 200.0 μL of the reaction mixture was directly transferred into the X-band EPR tube and frozen in liquid nitrogen for recording EPR spectrum (Fig. 4c, trace a).

CuTc+L3+Cy$_2$NMe+toluene+2a (O$_2$, 2 h): To an oven-dried microwave vial equipped with a stir bar was added CuTc (4.8 mg, 0.025 mmol), **L3** (12.4 mg, 0.05 mmol) and *p*-tolylboronic acid (**2a**) (68.0 mg, 0.5 mmol). Then, Cy$_2$NMe (80.3 μL, 0.38 mmol) and toluene (0.5 mL) was added via syringe. The vial was sealed with a septum, and refilled by an O$_2$ balloon for 3 min. The solution was then stirred at room temperature for 2 h under the O$_2$ atmosphere. After that, 200.0 μL of the reaction mixture was directly transferred into the X-band EPR tube and frozen in liquid nitrogen for recording EPR spectrum (Fig. 4c, trace b).

CuTc+L3+Cy$_2$NMe+toluene+2a (O$_2$, 2 h) + 1a (O$_2$, 20 min): To an oven-dried microwave vial equipped with a stir bar was added CuTc (4.8 mg, 0.025 mmol), **L3** (12.4 mg, 0.05 mmol) and *p*-tolylboronic acid (**2a**) (68.0 mg, 0.5 mmol). Then, Cy$_2$NMe (80.3 μL, 0.38 mmol) and toluene (0.5 mL) was added via syringe. The vial was sealed with a septum, and refilled by an O$_2$ balloon for 3 min. The solution was then stirred at room temperature for 2 h under an O$_2$ atmosphere. Then, *S*-(4-fluorophenyl)-*N*-(*p*-tolyl)thiohydroxylamine (**1a**) (54.8 mg, 0.25 mmol) was added. The reaction mixture was stirred for additional 20 min. After that, 200.0 μL of the reaction mixture was directly transferred into the X-band EPR tube and frozen in liquid nitrogen for recording EPR spectrum (Fig. 4c, trace c).

CuTc+L3+Cy$_2$NMe+toluene+2a+1a (N$_2$, 2 h), then O$_2$, 5 min: To an oven-dried microwave vial equipped with a stir bar was added CuTc (4.8 mg, 0.025 mmol), **L3** (12.4 mg, 0.05 mmol), *p*-tolylboronic acid (**2a**) (68.0 mg, 0.5 mmol) and *S*-(4-fluorophenyl)-*N*-(*p*-tolyl)thiohydroxylamine (**1a**) (54.8 mg, 0.25 mmol). Then, Cy$_2$NMe (80.3 μL, 0.38 mmol) and toluene (0.5 mL) was added via syringe. The vial

was sealed with a septum in the glovebox. The solution was stirred at room temperature for 2 h under an N_2 atmosphere. Then O_2 was injected and the reaction mixture was stirred for another 5 minutes. After that, 200.0 μL of the reaction mixture was directly transferred into the X-band EPR tube and frozen in liquid nitrogen for recording EPR spectrum (Fig. 4c, trace d).

CuTc+L3+Cy$_2$NMe+toluene+2a+^{15}N-1a (N_2, 2 h), then O_2, 5 min: To an oven-dried microwave vial equipped with a stir bar was added CuTc (4.8 mg, 0.025 mmol), **L3** (12.4 mg, 0.05 mmol), *p*-tolylboronic acid (**2a**) (68.0 mg, 0.5 mmol) and *S*-(4-fluorophenyl)-*N*-phenylthiohydroxylamine-*N*-^{15}N (^{15}N-**1a**) (55.1 mg, 0.25 mmol). Then, Cy$_2$NMe (80.3 μL, 0.38 mmol) and toluene (0.5 mL) was added via syringe. The vial was sealed with a septum in the glovebox. The solution was stirred at room temperature for 2 h under an N_2 atmosphere. Then O_2 was injected and the reaction mixture was stirred for another 5 minutes. After that, 200.0 μL of the reaction mixture was directly transferred into the X-band EPR tube and frozen in liquid nitrogen for recording EPR spectrum (Fig. 4c, trace e).

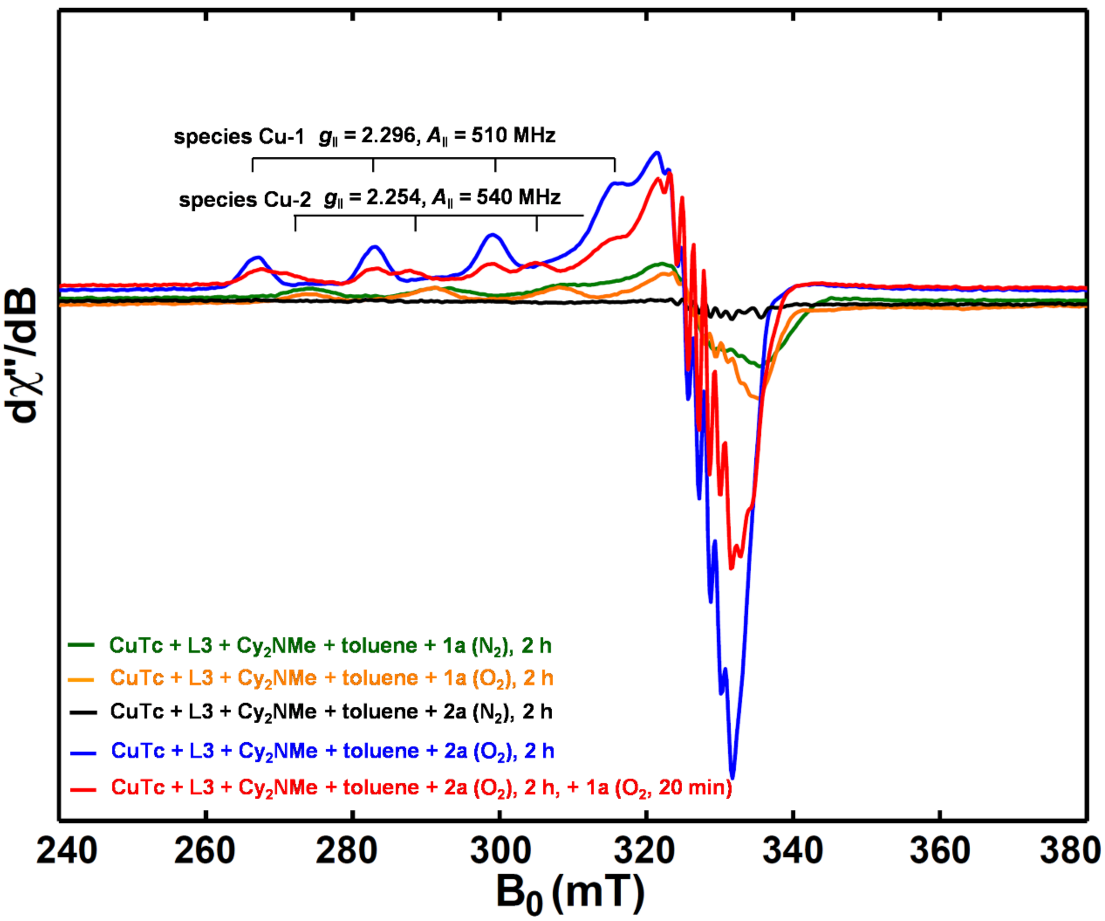

Supplementary Figure 2. X-band (9.35 GHz, 50 K) CW EPR spectra of varying control samples. Since Cu(I)Tc was used as the precursor, no obvious EPR signal was observed for those samples without O_2 exposure, except for the green trace which shows a small amount Cu(II) signal that is most likely from the impurity of Cu(I)Tc. For those samples exposed to O_2, well-defined Cu EPR signal was observed only in the presence of boronic acid **2a**. One single species, assigned as species **Cu-1**, was formed via adding **2a** to the system with CuTc, **L3**, base (Cy_2NMe) and O_2 (blue spectrum). *see* Supplementary Figure 4 for detailed analysis of species **Cu-1**.

Once further introducing sulfenamide **1a** to the system, a second species, assigned as species **Cu-2**, was observed (red spectrum). *see* Supplementary Figure 5 and 6 for detailed analysis of species **Cu-2**.

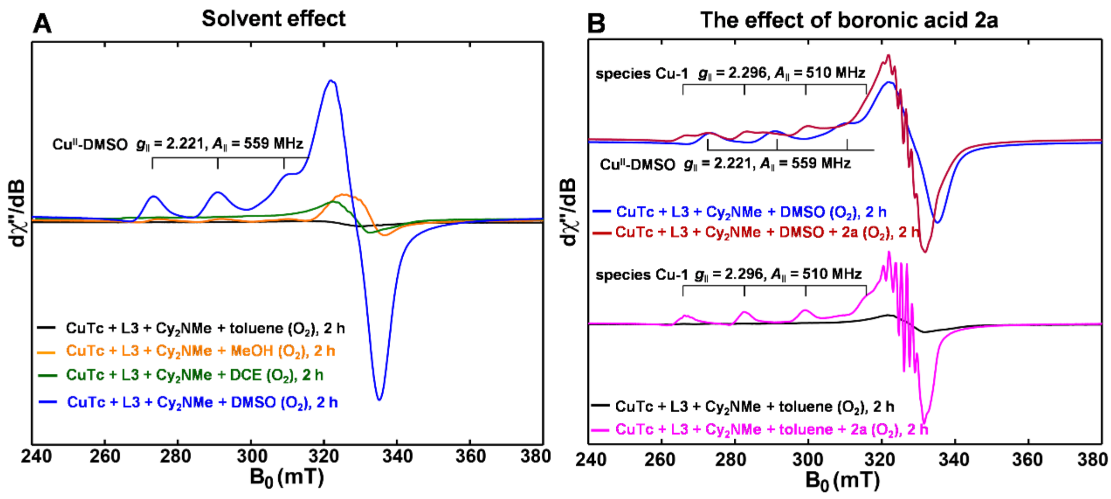

Supplementary Figure 3. X-band (9.35 GHz, 50 K) CW EPR spectra of varying control samples. (A) For solvents including toluene, MeOH and DCE, well-defined Cu EPR signal was observed only in the presence of boronic acid **2a**. For DMSO, although Cu EPR signal was observed (blue spectrum), no suprehyperfine splittings were detected, suggesting that Cu(II) could binds with DMSO. (B) once further introducing boronic acid **2a** to the system, species **Cu-1** was formed with some residual species of **Cu^{II}-DMSO**.

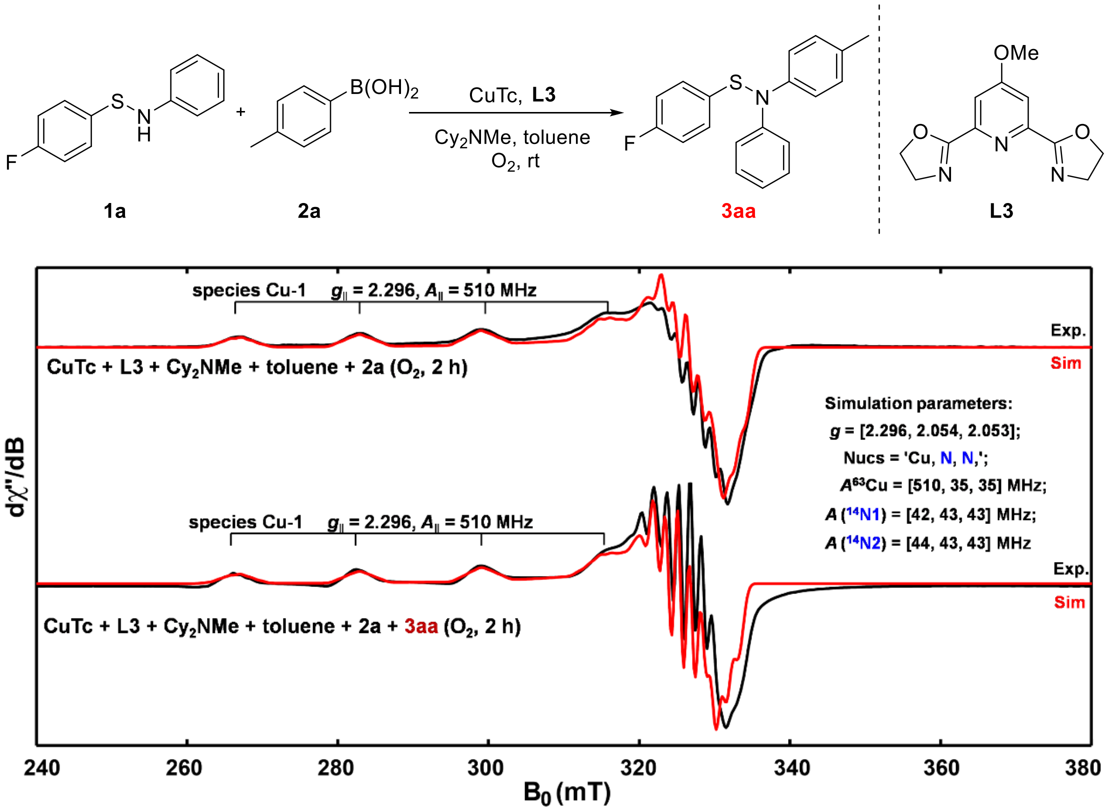

Supplementary Figure 4. Species **Cu-1** was well simulated by using a **g** tensor of [2.296, 2.054, 2.053] and a hyperfine tensor of $A(^{63}Cu)$ = [510, 35, 35] MHz. For fitting the superhyperfine coupling signals at the $g_{\perp} \sim 2.054$ region that is arising from the hyperfine interactions of the ^{14}N ($I = 1$) from the ligand **L3** with the electron spin, we noticed that only two ^{14}N nuclei were needed for a good fittting ($A(^{14}N1)$ = [42, 43, 43] MHz; $A(^{14}N2)$ = [44, 43, 43] MHz). This suggests that with the introduction of boronic acid **2a**, only two nitrogen of the ligand **L3** binds Cu(II). DFT analysis supports this assignment.

The bottom experimental and simulated spectra suggest that the product **3aa** does not binds to Cu(II).

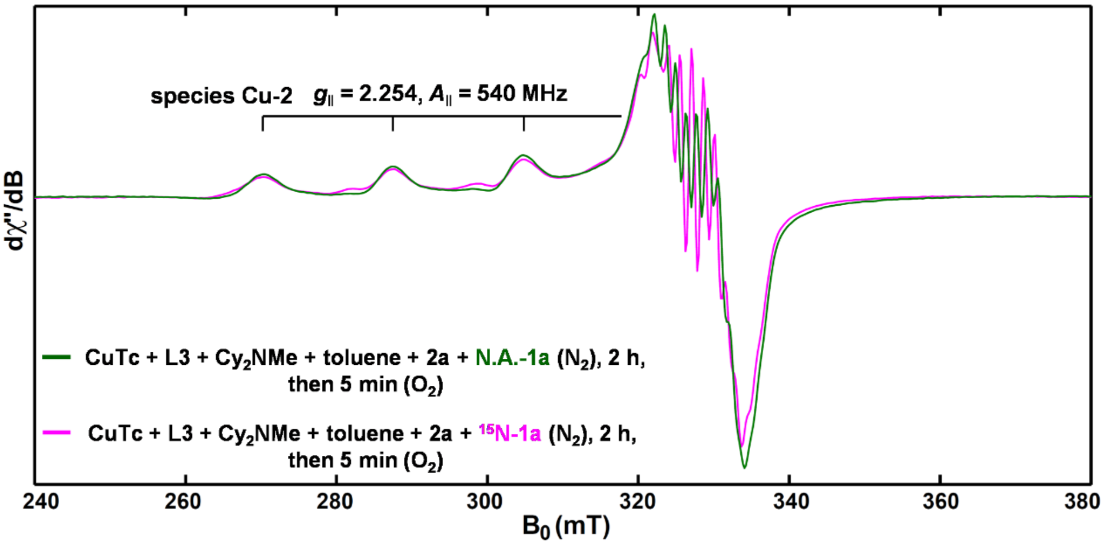

Supplementary Figure 5. Comparison of the spectrum of species **Cu-2** generated by using isotoplogs of sulfenamide **1a**. Corresponding isotope response was observed at the $g_{\perp} \sim 2.054$ region. *see* Supplementary Figure 6 for detailed analysis of species **Cu-2**.

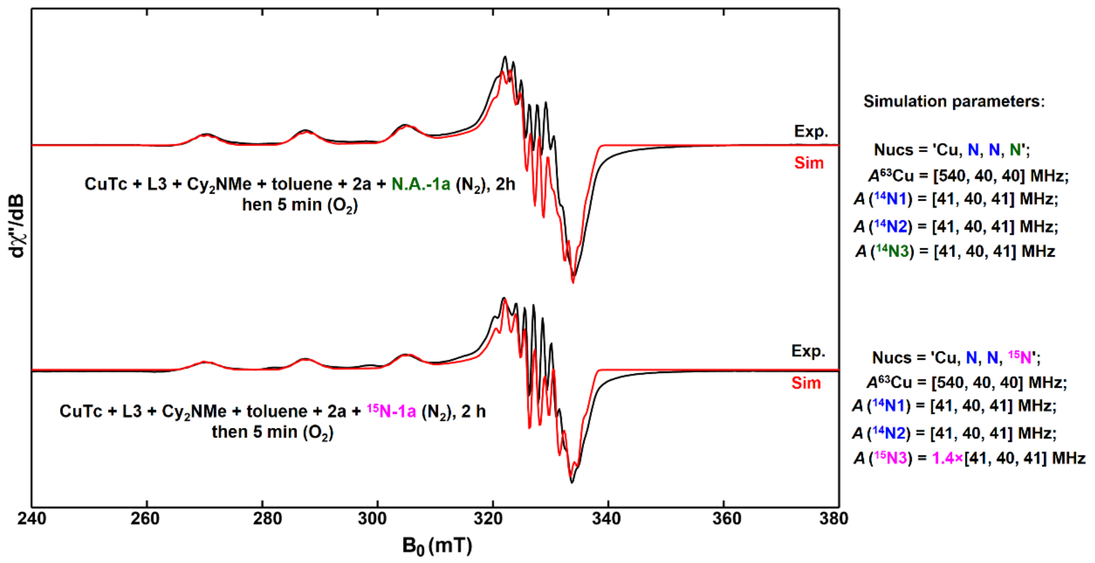

Supplementary Figure 6. Species **Cu-2** was well simulated by using a **g** tensor of [2.254, 2.046, 2.046] and a hyperfine tensor of $A(^{63}Cu)$ = [540, 40, 40] MHz. For fitting the superhyperfine coupling signals at the $g_{\perp} \sim 2.046$ region that is arising from the hyperfine interactions of the ^{14}N ($I = 1$) with the electron spin, three ^{14}N nuclei were needed for a good fittting ($A(^{14}N1)$ = [41, 40, 41] MHz; $A(^{14}N2)$ = [41, 40, 41] MHz; $A(^{14}N3)$ = [41, 40, 41] MHz). By comparing with species **Cu-1**, this suggests that sulfenamide **1a** binds to Cu(II) via nitrogen rather than sulfur. This was confirmed by using ^{15}N-labeled sulfenamide, the spectrum is well simulated by only adjusting the hyperfine value of the third nitrogen by using a scaling factor of $\gamma(^{15}N)/\gamma(^{14}N) = 1.403$, where the $\gamma(^{15}N)$ or $\gamma(^{14}N)$ is the gyromagnetic ratios of each nuclear spin.

3.1.4 Computational Studies

General Remarks

All optimizations of intermediates and transition states were calculated using unrestricted B3LYP[11,12]-D3[13]/6-31G(d)[14] -SDD(Cu)[15] using an ultrafine (99,590) grid with the “opt=noeigen” keyword as implemented in Gaussian16. Frequency calculations, using the same method, were used to obtain thermal corrections (at 298.15K; enthalpy and free energy) and to characterize the obtained stationary points as transition states (only one single imaginary frequency) or intermediate (zero imaginary frequencies). Conformational searches were performed manually for all intermediates and transition states, and only the lowest energy species were shown and discussed. Intrinsic reaction coordinate (IRC) calculations were undertaken to ensure transition states connected to the corresponding intermediates. Single point energy calculations using UM06[16]/6-311++G(d,p)[17,18] -SDD(Cu) with solvent corrections calculated in implicit solvent (dimethoxyethane) using CPCM[19] were also performed on all structures. All 3-D structures were generated using CYLview.[20] Noncovalent interaction (NCI) analysis, also known as reduce density gradient (RDG) method, was performed on Multiwfn to study the possible effect of noncovalent interaction in the stereodetermining transition states.[21] Extension distance of 0 Bohr, medium quality grid (totally about 512000 points) were set by default. Further visualization of the color-filled RDG isosurface was realized by VMD, where RDG isosurface and color range were set as 0.5, and -0.035 to 0.2, respectively.[22]

Full Reference of Gaussian 16 Software

Gaussian 16, Revision B.01, M. J. Frisch, G. W. Trucks, H. B. Schlegel, G. E. Scuseria, M. A. Robb, J. R. Cheeseman, G. Scalmani, V. Barone, G. A. Petersson, H. Nakatsuji, X. Li, M. Caricato, A. V. Marenich, J. Bloino, B. G. Janesko, R. Gomperts, B. Mennucci, H. P. Hratchian, J. V. Ortiz, A. F. Izmaylov, J. L. Sonnenberg, D. Williams-Young, F. Ding, F. Lipparini, F. Egidi, J. Goings, B. Peng, A. Petrone, T. Henderson, D. Ranasinghe, V. G. Zakrzewski, J. Gao, N. Rega, G. Zheng, W. Liang, M. Hada, M. Ehara, K. Toyota, R. Fukuda, J. Hasegawa, M. Ishida, T. Nakajima, Y. Honda, O. Kitao, H. Nakai, T. Vreven, K. Throssell, J. A. Montgomery, Jr., J. E. Peralta, F. Ogliaro, M. J. Bearpark, J. J. Heyd, E. N. Brothers, K. N. Kudin, V. N. Staroverov, T. A. Keith, R. Kobayashi, J. Normand, K. Raghavachari, A. P. Rendell, J. C. Burant, S. S. Iyengar, J. Tomasi, M. Cossi, J. M. Millam, M. Klene, C. Adamo, R. Cammi, J. W. Ochterski, R. L. Martin, K. Morokuma, O. Farkas, J. B. Foresman, and D. J. Fox, Gaussian, Inc., Wallingford CT, 2016.

Supporting Figures

Entry	Method<br>opt: UB3LYP-D3/6-31G(d)-SDD(Cu)	18a	18
1	UM06/6-311++G(d,p)-SDD(Cu)//opt	0.0	0.8
2	UM06/def2tzvp//opt	0.0	3.1
3	UB3LYP-D3/def2tzvp//opt	0.0	1.8
4	UB3LYP-D3/6-311++G(d,p)-SDD(Cu)//opt	0.0	0.0
5	UWB97XD/def2tzvp//opt	0.0	3.1
6	UWB97XD/6-311++G(d,p)-SDD(Cu)//opt	0.0	1.1
7	opt	0.0	-0.9
8	UM06/6-311++G(d,p)-SDD(Cu)-CPCM(DME)//opt	0.0	2.3

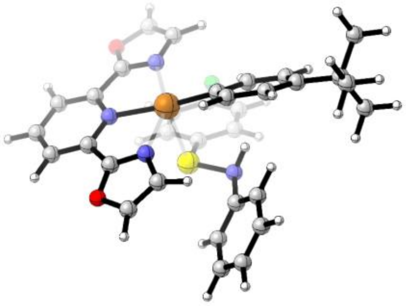

18a

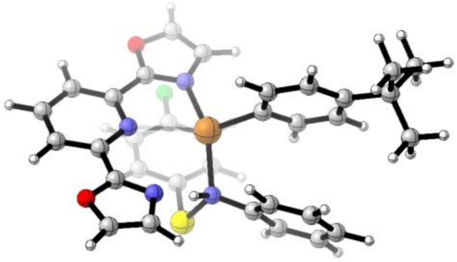

18

Supplementary Figure 7. Comparison of single point methods (with UB3LYP-D3/6-31G(d)-SDD(Cu) optimization) for the difference in free energies between **18a** and experimentally observed **18**.

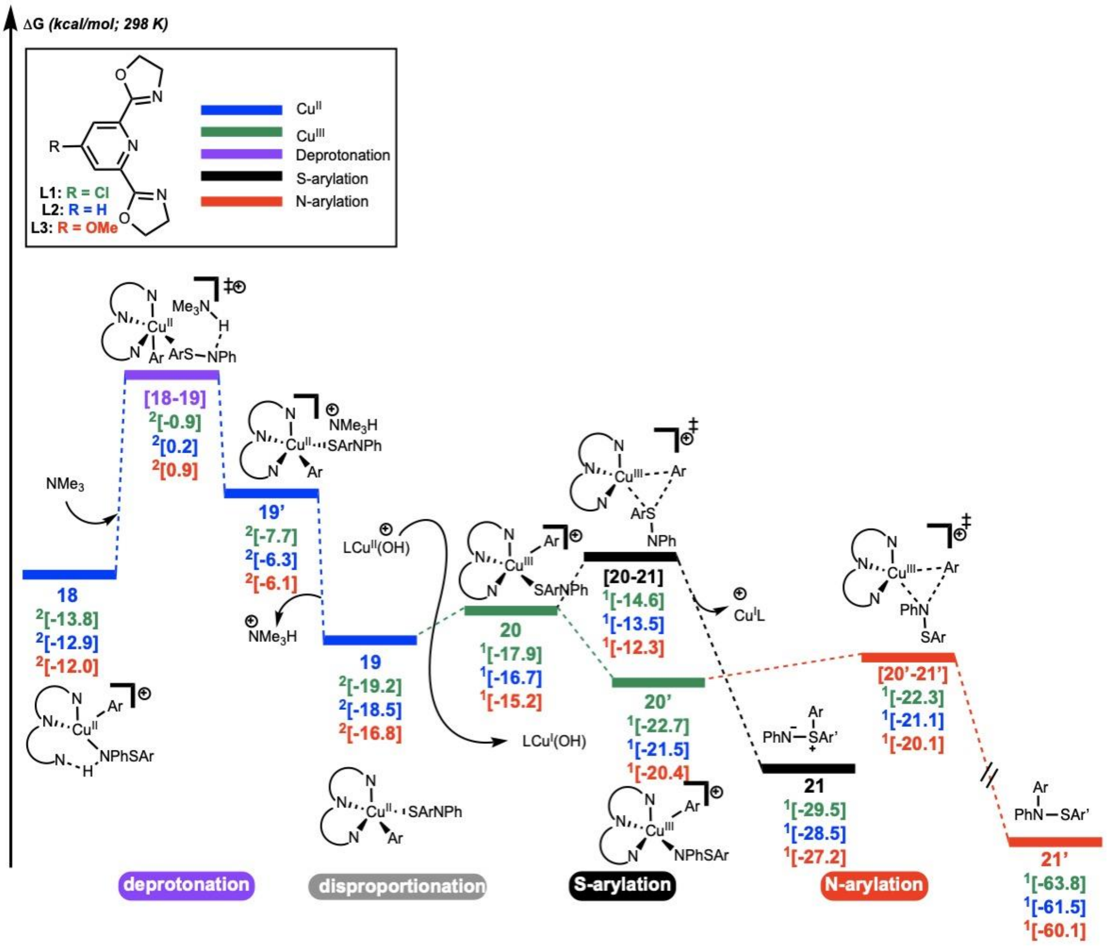

Supplementary Figure 8. Formation of *N*-arylation product **21'** with **L1** (green), **L2** (blue) and **L3** (red). All free energies were computed using UM06/6-311++G(d,p)-SDD(Cu)-CPCM(DME)//UB3LYP-D3/6-31G(d)-SDD(Cu).

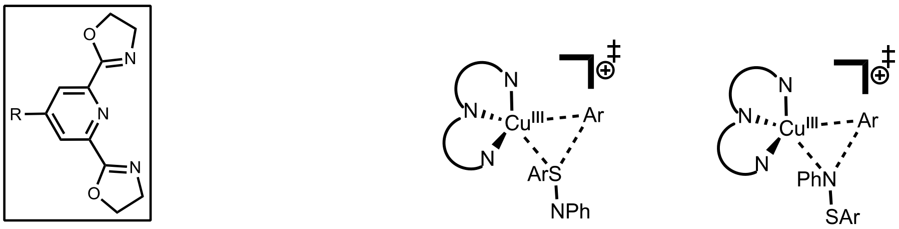

	Experimental N:S	Span for S-Arylation	Span for N-Arylation	Difference
R = Cl	56: 11	6.2 [4.6]	1.5 [0.4]	4.7 [4.2]
R = H	75: 7	7.1 [5.0]	1.5 [0.4]	5.5 [4.6]
R = OMe	87: 5	9.7 [4.5]	1.6 [0.3]	8.1 [4.2]

Supplementary Figure 9. Difference between barrier for *S*-arylation and *N*-arylation, computed using UB3LYP-D3/6-31G(d)-SDD(Cu) and UM06/6-311++G(d,p)-SDD(Cu)-CPCM(DME)//UB3LYP-D3/6-31G(d)-SDD(Cu) (brackets). Optimization method UB3LYP-D3/6-31G(d)-SDD(Cu) predicts that **L3** will be the most selective ligand, as observed experimentally.

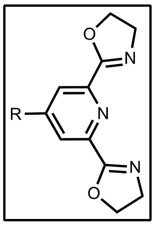

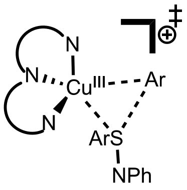

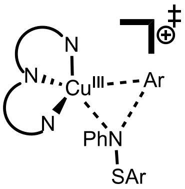

	Experimental N:S	Span for S-Arylation	Span for N-Arylation	Difference
R = Cl	56: 11	0.9 (4.4) [7.1]	0.6 (2.8) [1.7]	0.3 (1.6) [5.4]
R = H	75: 7	1.7 (5.3) [8.0]	2.0 (2.8) [1.8]	0.3 (2.5) [6.2]
R = OMe	87: 5	2.5 (7.2) [9.5]	1.9 (2.9) [1.7]	0.6 (4.3) [7.8]

Supplementary Figure 10. Difference between barrier for *S*-arylation and *N*-arylation, computed using UM06/6-311++G(d,p)-SDD(Cu)//UB3LYP-D3/6-31G(d)-SDD(Cu), UM06L/6-311++G(d,p)-SDD(Cu)//UB3LYP-D3/6-31G(d)-SDD(Cu) (parenthesis), and UB3LYP-D3/6-311++G(d,p)-SDD(Cu)//UB3LYP-D3/6-31G(d)-SDD(Cu) (brackets). Single point energies predict **L3** to be the most selective ligand.

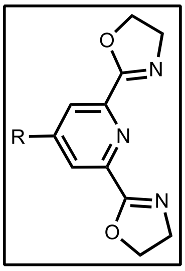

	ΔG	Interaction E	Distortion E	Int+Dist
S-arylation				
L1, R = Cl	-0.8	-25.4	4.5	-20.9
L2, R = H	0.5	-25.0	4.6	-20.5
L3, R = OMe	2.9	-23.9	4.0	-19.9
N-arylation				
L1, R = Cl	-13.8	-52.5	4.9	-47.7
L2, R = H	-12.5	-52.3	5.0	-47.3
L3, R = OMe	-10.4	-52.3	5.3	-47.0

Supplementary Figure 11. Interaction-distortion analysis of *S*-arylation and *N*-arylation with **L1, L2,** and **L3** computed using UM06/6-311++G(d,p)-SDD(Cu)-CPCM(DME)//UB3LYP-D3/6-31G(d)-SDD(Cu).

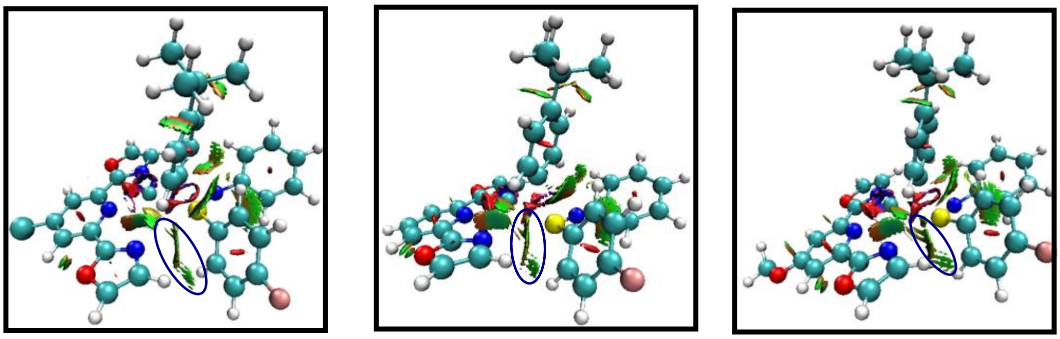

[20-21]-Cl
L1
-14.6

[20-21]
L2
-13.5

[20-21]-OMe
L3
-12.3

Supplementary Figure 12. A comparison of NCI plots for *S*-arylation transition states **[20-21]** for **L1**, **L2**, and **L3**. There is a slightly larger favorable interaction

between the aryl of the sulfenamide and the ligand in **L1**. All free energies were computed using UM06/6-311++G(d,p)-SDD(Cu)-CPCM(DME)//UB3LYP-D3/6-31G(d)-SDD(Cu).

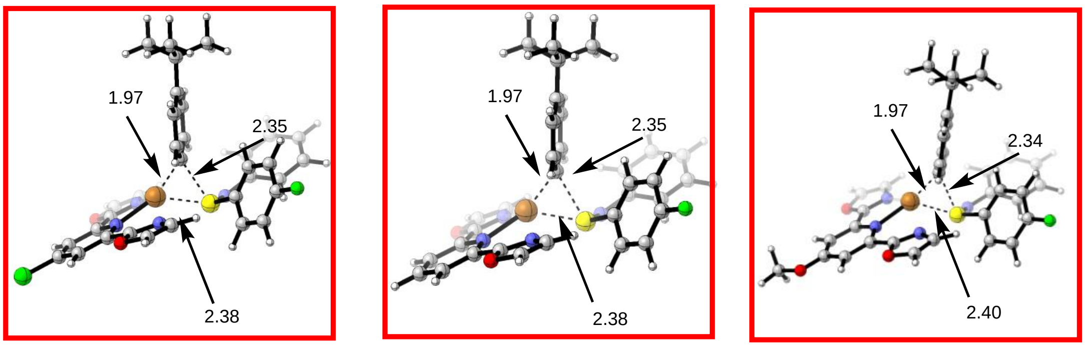

[20’-21’]-Cl
L1
-22.3

[20’-21’]
L2
-21.1

[20’-21’]-OMe
L3
-20.1

Supplementary Figure 13. A comparison of *N*-arylation transition states **[20’-21’]** for **L1**, **L2**, and **L3** with a slightly shorter C-S bond for **L3**, indicating it is a later transition state with more distortion. All free energies were computed using UM06/6-311++G(d,p)-SDD(Cu)-CPCM(DME)//UB3LYP-D3/6-31G(d)-SDD(Cu).

Calculated Structures and Energies

16

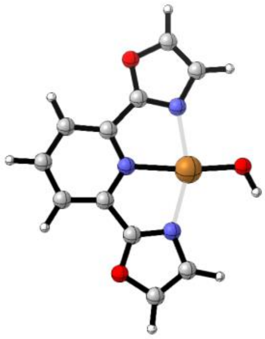

Supplementary Figure 14.
UB3LYP-D3/6-31G(d)-SDD(Cu)
Zero-point correction= 0.183308 (Hartree/Particle)
Thermal correction to Energy= 0.198183
Thermal correction to Enthalpy= 0.199127
Thermal correction to Gibbs Free Energy= 0.140074
Sum of electronic and zero-point Energies= -1010.896026
Sum of electronic and thermal Energies= -1010.881151
Sum of electronic and thermal Enthalpies= -1010.880207
Sum of electronic and thermal Free Energies= -1010.939260

UM06/6-311++G(d,p)-SDD(Cu)//UB3LYP-D3/6-31G(d)-SDD(Cu)
HF = -1010.8366105

16-Cl

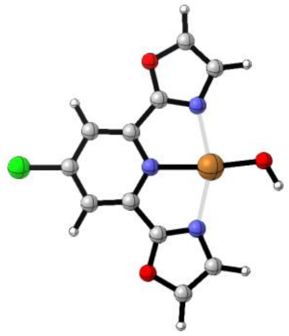

Supplementary Figure 15.
UB3LYP-D3/6-31G(d)-SDD(Cu)
Zero-point correction= 0.173402 (Hartree/Particle)
Thermal correction to Energy= 0.189566
Thermal correction to Enthalpy= 0.190510
Thermal correction to Gibbs Free Energy= 0.128076
Sum of electronic and zero-point Energies= -1470.493387
Sum of electronic and thermal Energies= -1470.477223
Sum of electronic and thermal Enthalpies= -1470.476279
Sum of electronic and thermal Free Energies= -1470.538713

UM06/6-311++G(d,p)-SDD(Cu)//UB3LYP-D3/6-31G(d)-SDD(Cu)
HF = -1470.4252474

16-OMe

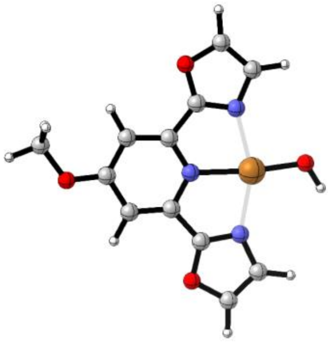

Supplementary Figure 16.
UB3LYP-D3/6-31G(d)-SDD(Cu)
Zero-point correction= 0.216166 (Hartree/Particle)
Thermal correction to Energy= 0.233706
Thermal correction to Enthalpy= 0.234650
Thermal correction to Gibbs Free Energy= 0.169673
Sum of electronic and zero-point Energies= -1125.398346
Sum of electronic and thermal Energies= -1125.380807
Sum of electronic and thermal Enthalpies= -1125.379862

Sum of electronic and thermal Free Energies= -1125.444840

UM06/6-311++G(d,p)-SDD(Cu)//UB3LYP-D3/6-31G(d)-SDD(Cu)
HF = -1125.3374162

16'

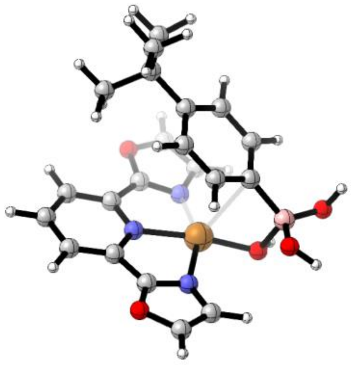

Supplementary Figure 17.
UB3LYP-D3/6-31G(d)-SDD(Cu)
Zero-point correction= 0.424130 (Hartree/Particle)
Thermal correction to Energy= 0.452939
Thermal correction to Enthalpy= 0.453883
Thermal correction to Gibbs Free Energy= 0.365279
Sum of electronic and zero-point Energies= -1576.224248
Sum of electronic and thermal Energies= -1576.195439
Sum of electronic and thermal Enthalpies= -1576.194495
Sum of electronic and thermal Free Energies= -1576.283099

UM06/6-311++G(d,p)-SDD(Cu)//UB3LYP-D3/6-31G(d)-SDD(Cu)
HF = -1576.1492471

[16-17]

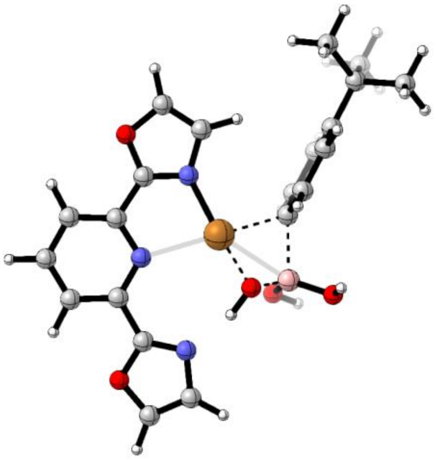

Supplementary Figure 18.

UB3LYP-D3/6-31G(d)-SDD(Cu)
Imaginary frequency = -194.26 cm^{-1}
Zero-point correction= 0.422326 (Hartree/Particle)
Thermal correction to Energy= 0.451145
Thermal correction to Enthalpy= 0.452089
Thermal correction to Gibbs Free Energy= 0.360748
Sum of electronic and zero-point Energies= -1576.217043
Sum of electronic and thermal Energies= -1576.188224
Sum of electronic and thermal Enthalpies= -1576.187280
Sum of electronic and thermal Free Energies= -1576.278620

UM06/6-311++G(d,p)-SDD(Cu)//UB3LYP-D3/6-31G(d)-SDD(Cu)
HF = -1576.1346146

17

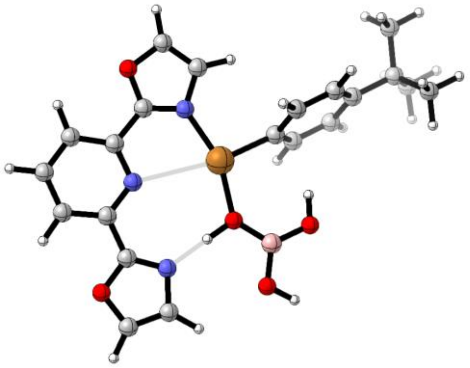

Supplementary Figure 19.
UB3LYP-D3/6-31G(d)-SDD(Cu)
Zero-point correction= 0.423373 (Hartree/Particle)
Thermal correction to Energy= 0.453023
Thermal correction to Enthalpy= 0.453967
Thermal correction to Gibbs Free Energy= 0.358143
Sum of electronic and zero-point Energies= -1576.250986
Sum of electronic and thermal Energies= -1576.221336
Sum of electronic and thermal Enthalpies= -1576.220392
Sum of electronic and thermal Free Energies= -1576.316216

UM06/6-311++G(d,p)-SDD(Cu)//UB3LYP-D3/6-31G(d)-SDD(Cu)
HF = -1576.1641008

18

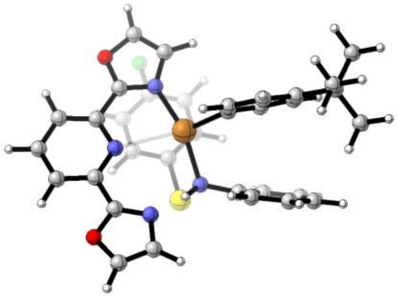

Supplementary Figure 20.
UB3LYP-D3/6-31G(d)-SDD(Cu)
Zero-point correction= 0.566098 (Hartree/Particle)
Thermal correction to Energy= 0.604591
Thermal correction to Enthalpy= 0.605535
Thermal correction to Gibbs Free Energy= 0.489793
Sum of electronic and zero-point Energies= -2339.706457
Sum of electronic and thermal Energies= -2339.667964
Sum of electronic and thermal Enthalpies= -2339.667020
Sum of electronic and thermal Free Energies= -2339.782762

UM06/6-311++G(d,p)-SDD(Cu)//UB3LYP-D3/6-31G(d)-SDD(Cu)
HF = -2339.4946616

18a

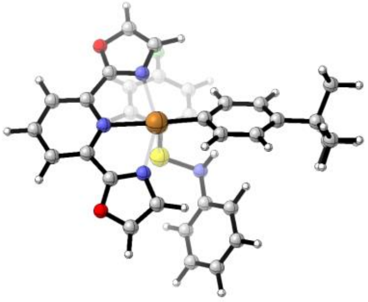

Supplementary Figure 21.
UB3LYP-D3/6-31G(d)-SDD(Cu)
Zero-point correction= 0.566700 (Hartree/Particle)
Thermal correction to Energy= 0.605116
Thermal correction to Enthalpy= 0.606061
Thermal correction to Gibbs Free Energy= 0.491530
Sum of electronic and zero-point Energies= -2339.706100
Sum of electronic and thermal Energies= -2339.667684
Sum of electronic and thermal Enthalpies= -2339.666739
Sum of electronic and thermal Free Energies= -2339.781270

UM06/6-311++G(d,p)-SDD(Cu)//UB3LYP-D3/6-31G(d)-SDD(Cu)

HF = -2339.5000397

[18-19]

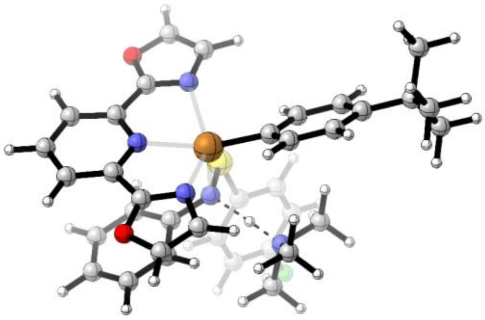

Supplementary Figure 22.
UB3LYP-D3/6-31G(d)-SDD(Cu)
Imaginary frequency = -441.09 cm^{-1}
Zero-point correction= 0.687392 (Hartree/Particle)
Thermal correction to Energy= 0.731806
Thermal correction to Enthalpy= 0.732750
Thermal correction to Gibbs Free Energy= 0.606719
Sum of electronic and zero-point Energies= -2514.086365
Sum of electronic and thermal Energies= -2514.041951
Sum of electronic and thermal Enthalpies= -2514.041007
Sum of electronic and thermal Free Energies= -2514.167038

UM06/6-311++G(d,p)-SDD(Cu)//UB3LYP-D3/6-31G(d)-SDD(Cu)
HF = -2513.8915345

19

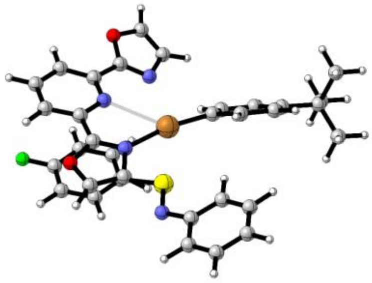

Supplementary Figure 23.
UB3LYP-D3/6-31G(d)-SDD(Cu)
Zero-point correction= 0.551311 (Hartree/Particle)
Thermal correction to Energy= 0.590275
Thermal correction to Enthalpy= 0.591219
Thermal correction to Gibbs Free Energy= 0.472277
Sum of electronic and zero-point Energies= -2339.278665

Sum of electronic and thermal Energies= -2339.239701
Sum of electronic and thermal Enthalpies= -2339.238757
Sum of electronic and thermal Free Energies= -2339.357699

UM06/6-311++G(d,p)-SDD(Cu)//UB3LYP-D3/6-31G(d)-SDD(Cu)
HF = -2339.0389963

20

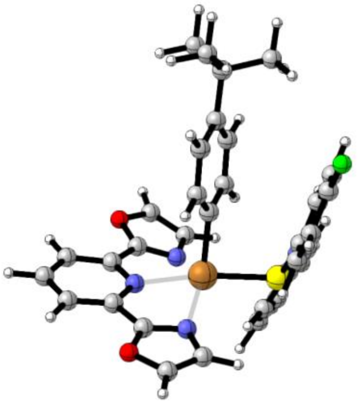

Supplementary Figure 24.
UB3LYP-D3/6-31G(d)-SDD(Cu)
Zero-point correction= 0.553167 (Hartree/Particle)
Thermal correction to Energy= 0.591583
Thermal correction to Enthalpy= 0.592527
Thermal correction to Gibbs Free Energy= 0.478122
Sum of electronic and zero-point Energies= -2339.075209
Sum of electronic and thermal Energies= -2339.036793
Sum of electronic and thermal Enthalpies= -2339.035849
Sum of electronic and thermal Free Energies= -2339.150253

UM06/6-311++G(d,p)-SDD(Cu)//UB3LYP-D3/6-31G(d)-SDD(Cu)
HF = -2338.8554299

[20-21]

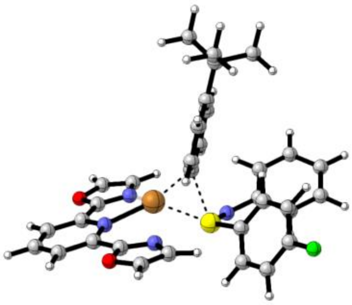

Supplementary Figure 25.

UB3LYP-D3/6-31G(d)-SDD(Cu)
Imaginary frequency = -133.82 cm^{-1}
Zero-point correction= 0.553183 (Hartree/Particle)
Thermal correction to Energy= 0.591141
Thermal correction to Enthalpy= 0.592085
Thermal correction to Gibbs Free Energy= 0.476579
Sum of electronic and zero-point Energies= -2339.064138
Sum of electronic and thermal Energies= -2339.026180
Sum of electronic and thermal Enthalpies= -2339.025236
Sum of electronic and thermal Free Energies= -2339.140742
UM06/6-311++G(d,p)-SDD(Cu)//UB3LYP-D3/6-31G(d)-SDD(Cu)
HF = -2338.8487864

21

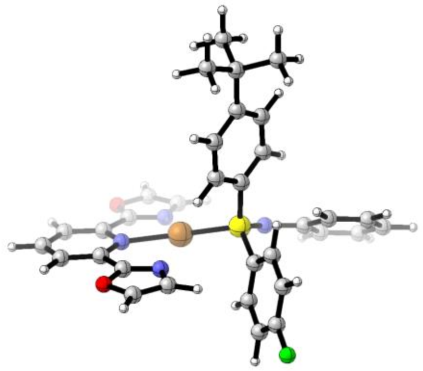

Supplementary Figure 26.
UB3LYP-D3/6-31G(d)-SDD(Cu)
Zero-point correction= 0.555019 (Hartree/Particle)
Thermal correction to Energy= 0.593177
Thermal correction to Enthalpy= 0.594121
Thermal correction to Gibbs Free Energy= 0.478574
Sum of electronic and zero-point Energies= -2339.088624
Sum of electronic and thermal Energies= -2339.050466
Sum of electronic and thermal Enthalpies= -2339.049522
Sum of electronic and thermal Free Energies= -2339.165069

UM06/6-311++G(d,p)-SDD(Cu)//UB3LYP-D3/6-31G(d)-SDD(Cu)
HF = -2338.8746716

20’

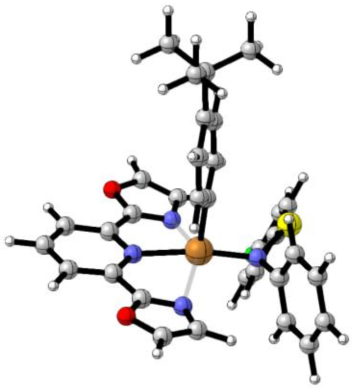

Supplementary Figure 27.
UB3LYP-D3/6-31G(d)-SDD(Cu)
Zero-point correction= 0.554037 (Hartree/Particle)
Thermal correction to Energy= 0.592078
Thermal correction to Enthalpy= 0.593022
Thermal correction to Gibbs Free Energy= 0.479795
Sum of electronic and zero-point Energies= -2339.089665
Sum of electronic and thermal Energies= -2339.051624
Sum of electronic and thermal Enthalpies= -2339.050680
Sum of electronic and thermal Free Energies= -2339.163907

UM06/6-311++G(d,p)-SDD(Cu)//UB3LYP-D3/6-31G(d)-SDD(Cu)
HF = -2338.8648096
[20'-21']

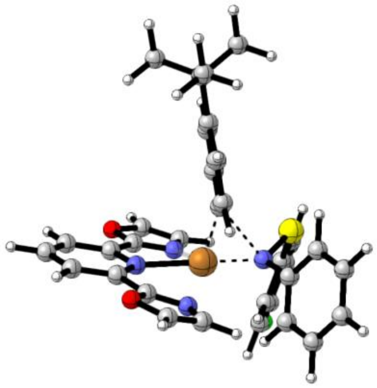

Supplementary Figure 28.
UB3LYP-D3/6-31G(d)-SDD(Cu)
Imaginary frequency = -137.08 cm^{-1}
Zero-point correction= 0.553518 (Hartree/Particle)
Thermal correction to Energy= 0.590967
Thermal correction to Enthalpy= 0.591911
Thermal correction to Gibbs Free Energy= 0.480332
Sum of electronic and zero-point Energies= -2339.088202
Sum of electronic and thermal Energies= -2339.050753

Sum of electronic and thermal Enthalpies= -2339.049808
Sum of electronic and thermal Free Energies= -2339.161387

UM06/6-311++G(d,p)-SDD(Cu)//UB3LYP-D3/6-31G(d)-SDD(Cu)
HF = -2338.8647541

21'

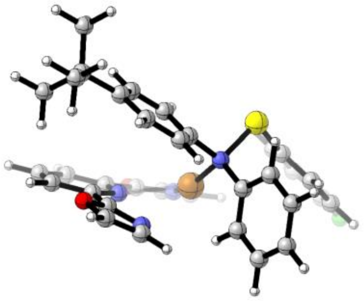

Supplementary Figure 29.
UB3LYP-D3/6-31G(d)-SDD(Cu)
Zero-point correction= 0.556264 (Hartree/Particle)
Thermal correction to Energy= 0.593810
Thermal correction to Enthalpy= 0.594754
Thermal correction to Gibbs Free Energy= 0.483526
Sum of electronic and zero-point Energies= -2339.147757
Sum of electronic and thermal Energies= -2339.110211
Sum of electronic and thermal Enthalpies= -2339.109267
Sum of electronic and thermal Free Energies= -2339.220495

UM06/6-311++G(d,p)-SDD(Cu)//UB3LYP-D3/6-31G(d)-SDD(Cu)
HF = -2338.9322314

18-Cl

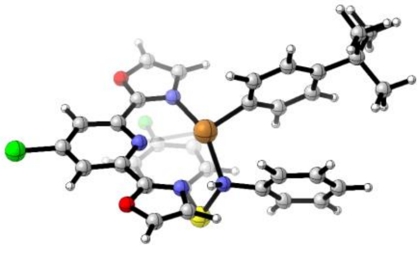

Supplementary Figure 30.
UB3LYP-D3/6-31G(d)-SDD(Cu)
Zero-point correction= 0.556224 (Hartree/Particle)
Thermal correction to Energy= 0.596013
Thermal correction to Enthalpy= 0.596957
Thermal correction to Gibbs Free Energy= 0.477872

Sum of electronic and zero-point Energies= -2799.305438
Sum of electronic and thermal Energies= -2799.265648
Sum of electronic and thermal Enthalpies= -2799.264704
Sum of electronic and thermal Free Energies= -2799.383789

UM06/6-311++G(d,p)-SDD(Cu)//UB3LYP-D3/6-31G(d)-SDD(Cu)
HF = -2799.0848949

[18-19]-Cl

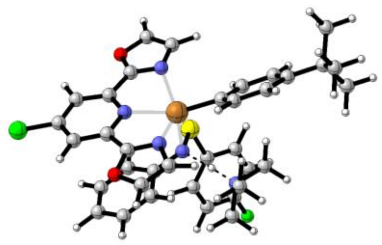

Supplementary Figure 31.
UB3LYP-D3/6-31G(d)-SDD(Cu)
Imaginary frequency = -567.82 cm^{-1}
Zero-point correction= 0.677363 (Hartree/Particle)
Thermal correction to Energy= 0.723114
Thermal correction to Enthalpy= 0.724058
Thermal correction to Gibbs Free Energy= 0.594652
Sum of electronic and zero-point Energies= -2973.686304
Sum of electronic and thermal Energies= -2973.640553
Sum of electronic and thermal Enthalpies= -2973.639608
Sum of electronic and thermal Free Energies= -2973.769015

UM06/6-311++G(d,p)-SDD(Cu)//UB3LYP-D3/6-31G(d)-SDD(Cu)
HF = -2973.4824116

19’-Cl

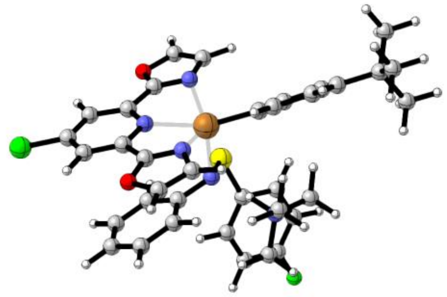

Supplementary Figure 32.
UB3LYP-D3/6-31G(d)-SDD(Cu)
Zero-point correction= 0.680414 (Hartree/Particle)
Thermal correction to Energy= 0.726593

Thermal correction to Enthalpy= 0.727538
Thermal correction to Gibbs Free Energy= 0.596643
Sum of electronic and zero-point Energies= -2973.683572
Sum of electronic and thermal Energies= -2973.637392
Sum of electronic and thermal Enthalpies= -2973.636448
Sum of electronic and thermal Free Energies= -2973.767342

UM06/6-311++G(d,p)-SDD(Cu)//UB3LYP-D3/6-31G(d)-SDD(Cu)
HF = -2973.483845

19-Cl

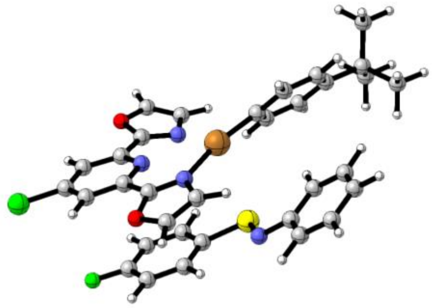

Supplementary Figure 33.
UB3LYP-D3/6-31G(d)-SDD(Cu)
Zero-point correction= 0.541490 (Hartree/Particle)
Thermal correction to Energy= 0.581741
Thermal correction to Enthalpy= 0.582685
Thermal correction to Gibbs Free Energy= 0.460359
Sum of electronic and zero-point Energies= -2798.880828
Sum of electronic and thermal Energies= -2798.840578
Sum of electronic and thermal Enthalpies= -2798.839633
Sum of electronic and thermal Free Energies= -2798.961959

UM06/6-311++G(d,p)-SDD(Cu)//UB3LYP-D3/6-31G(d)-SDD(Cu)
HF = -2798.6308293

20-Cl

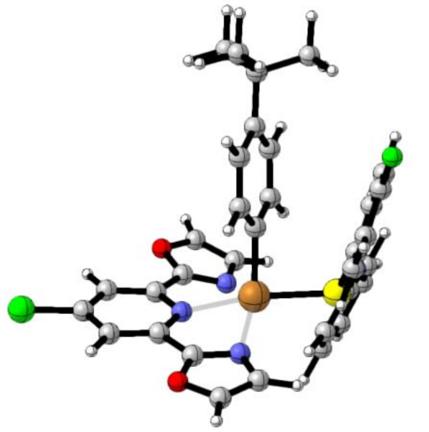

Supplementary Figure 34.
UB3LYP-D3/6-31G(d)-SDD(Cu)
Zero-point correction= 0.543255 (Hartree/Particle)
Thermal correction to Energy= 0.582991
Thermal correction to Enthalpy= 0.583935
Thermal correction to Gibbs Free Energy= 0.466018
Sum of electronic and zero-point Energies= -2798.674050
Sum of electronic and thermal Energies= -2798.634315
Sum of electronic and thermal Enthalpies= -2798.633371
Sum of electronic and thermal Free Energies= -2798.751287

UM06/6-311++G(d,p)-SDD(Cu)//UB3LYP-D3/6-31G(d)-SDD(Cu)
HF = -2798.4453736

[20-21]-Cl

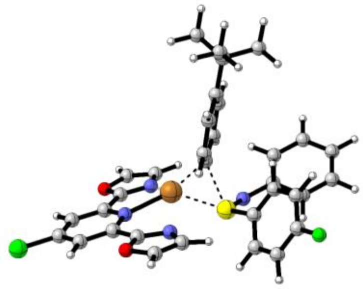

Supplementary Figure 35.
UB3LYP-D3/6-31G(d)-SDD(Cu)
Imaginary frequency = -134.93 cm^{-1}
Zero-point correction= 0.543268 (Hartree/Particle)
Thermal correction to Energy= 0.582540
Thermal correction to Enthalpy= 0.583484
Thermal correction to Gibbs Free Energy= 0.464540
Sum of electronic and zero-point Energies= -2798.662993
Sum of electronic and thermal Energies= -2798.623721
Sum of electronic and thermal Enthalpies= -2798.622777

Sum of electronic and thermal Free Energies= -2798.741721

UM06/6-311++G(d,p)-SDD(Cu)//UB3LYP-D3/6-31G(d)-SDD(Cu)
HF = -2798.4386383
21-Cl

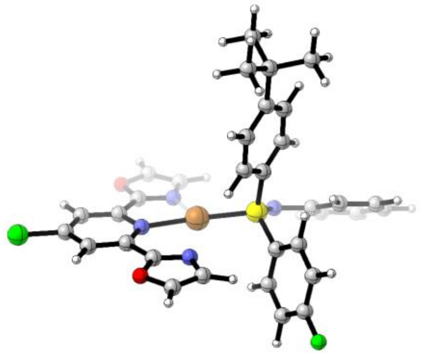

Supplementary Figure 36.
UB3LYP-D3/6-31G(d)-SDD(Cu)
Zero-point correction= 0.545144 (Hartree/Particle)
Thermal correction to Energy= 0.584594
Thermal correction to Enthalpy= 0.585539
Thermal correction to Gibbs Free Energy= 0.466685
Sum of electronic and zero-point Energies= -2798.687318
Sum of electronic and thermal Energies= -2798.647868
Sum of electronic and thermal Enthalpies= -2798.646924
Sum of electronic and thermal Free Energies= -2798.765777

UM06/6-311++G(d,p)-SDD(Cu)//UB3LYP-D3/6-31G(d)-SDD(Cu)
HF = -2798.4646008
20’-Cl

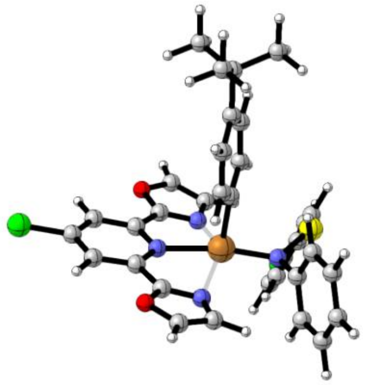

Supplementary Figure 37.
UB3LYP-D3/6-31G(d)-SDD(Cu)
Zero-point correction= 0.544132 (Hartree/Particle)
Thermal correction to Energy= 0.583485

Thermal correction to Enthalpy= 0.584429
Thermal correction to Gibbs Free Energy= 0.467830
Sum of electronic and zero-point Energies= -2798.688620
Sum of electronic and thermal Energies= -2798.649267
Sum of electronic and thermal Enthalpies= -2798.648323
Sum of electronic and thermal Free Energies= -2798.764922

UM06/6-311++G(d,p)-SDD(Cu)//UB3LYP-D3/6-31G(d)-SDD(Cu)
HF = -2798.454791

[20'-21']-Cl

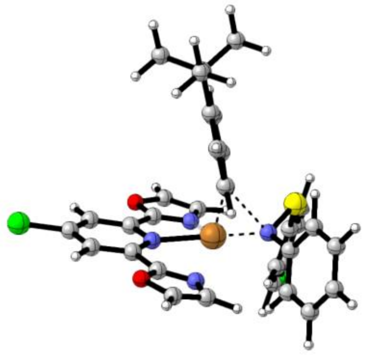

Supplementary Figure 38.
UB3LYP-D3/6-31G(d)-SDD(Cu)
Imaginary frequency = -136.32 cm^{-1}
Zero-point correction= 0.543611 (Hartree/Particle)
Thermal correction to Energy= 0.582372
Thermal correction to Enthalpy= 0.583317
Thermal correction to Gibbs Free Energy= 0.468310
Sum of electronic and zero-point Energies= -2798.687163
Sum of electronic and thermal Energies= -2798.648403
Sum of electronic and thermal Enthalpies= -2798.647458
Sum of electronic and thermal Free Energies= -2798.762464

UM06/6-311++G(d,p)-SDD(Cu)//UB3LYP-D3/6-31G(d)-SDD(Cu)
HF = -2798.4547057

21'-Cl

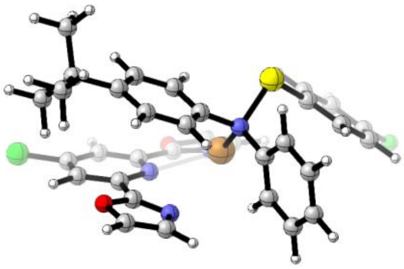

Supplementary Figure 39.

UB3LYP-D3/6-31G(d)-SDD(Cu)

Zero-point correction= 0.546380 (Hartree/Particle)
Thermal correction to Energy= 0.585242
Thermal correction to Enthalpy= 0.586186
Thermal correction to Gibbs Free Energy= 0.471193
Sum of electronic and zero-point Energies= -2798.747428
Sum of electronic and thermal Energies= -2798.708566
Sum of electronic and thermal Enthalpies= -2798.707621
Sum of electronic and thermal Free Energies= -2798.822615

UM06/6-311++G(d,p)-SDD(Cu)//UB3LYP-D3/6-31G(d)-SDD(Cu)
HF = -2798.5236247

18-OMe

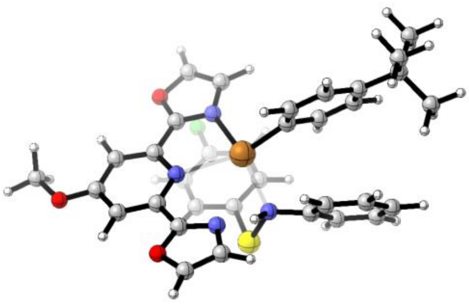

Supplementary Figure 40.

UB3LYP-D3/6-31G(d)-SDD(Cu)

Zero-point correction= 0.599127 (Hartree/Particle)
Thermal correction to Energy= 0.640199
Thermal correction to Enthalpy= 0.641143
Thermal correction to Gibbs Free Energy= 0.519992
Sum of electronic and zero-point Energies= -2454.206140
Sum of electronic and thermal Energies= -2454.165069
Sum of electronic and thermal Enthalpies= -2454.164124
Sum of electronic and thermal Free Energies= -2454.285276

UM06/6-311++G(d,p)-SDD(Cu)//UB3LYP-D3/6-31G(d)-SDD(Cu)
HF = -2453.9947646

[18-19]-OMe

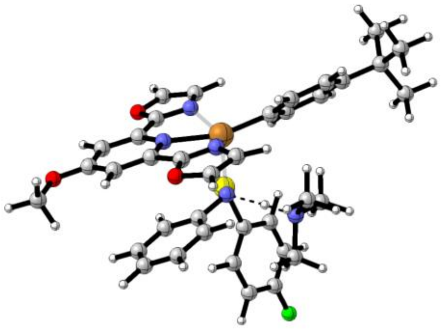

Supplementary Figure 41.
UB3LYP-D3/6-31G(d)-SDD(Cu)
Imaginary frequency = -382.58 cm^{-1}
Zero-point correction= 0.720416 (Hartree/Particle)
Thermal correction to Energy= 0.767401
Thermal correction to Enthalpy= 0.768345
Thermal correction to Gibbs Free Energy= 0.636778
Sum of electronic and zero-point Energies= -2628.586689
Sum of electronic and thermal Energies= -2628.539705
Sum of electronic and thermal Enthalpies= -2628.538760
Sum of electronic and thermal Free Energies= -2628.670328

UM06/6-311++G(d,p)-SDD(Cu)//UB3LYP-D3/6-31G(d)-SDD(Cu)
HF = -2628.3922031

19'-OMe

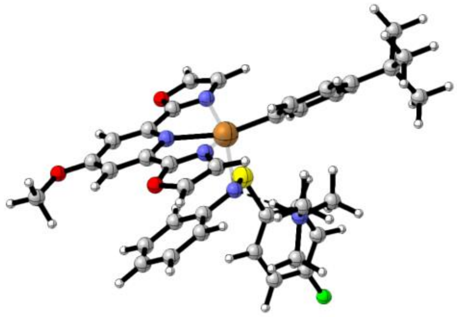

Supplementary Figure 42.
UB3LYP-D3/6-31G(d)-SDD(Cu)
Zero-point correction= 0.722890 (Hartree/Particle)
Thermal correction to Energy= 0.770390
Thermal correction to Enthalpy= 0.771334
Thermal correction to Gibbs Free Energy= 0.638085
Sum of electronic and zero-point Energies= -2628.584348
Sum of electronic and thermal Energies= -2628.536848
Sum of electronic and thermal Enthalpies= -2628.535904
Sum of electronic and thermal Free Energies= -2628.669153

UM06/6-311++G(d,p)-SDD(Cu)//UB3LYP-D3/6-31G(d)-SDD(Cu)
HF = -2628.3932649

19-OMe

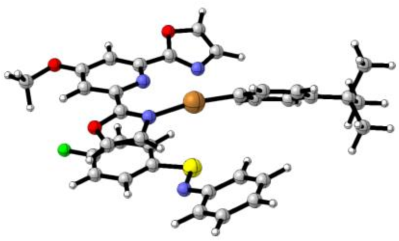

Supplementary Figure 43.
UB3LYP-D3/6-31G(d)-SDD(Cu)
Zero-point correction= 0.584381 (Hartree/Particle)
Thermal correction to Energy= 0.625855
Thermal correction to Enthalpy= 0.626799
Thermal correction to Gibbs Free Energy= 0.502754
Sum of electronic and zero-point Energies= -2453.777342
Sum of electronic and thermal Energies= -2453.735869
Sum of electronic and thermal Enthalpies= -2453.734925
Sum of electronic and thermal Free Energies= -2453.858970

UM06/6-311++G(d,p)-SDD(Cu)//UB3LYP-D3/6-31G(d)-SDD(Cu)
HF = -2453.5369602

20-OMe

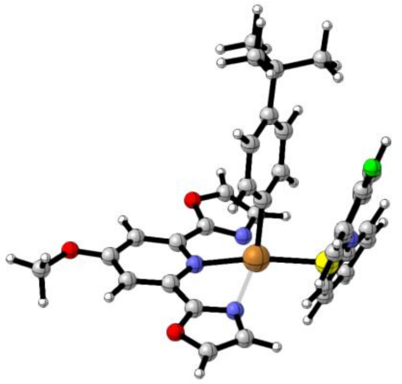

Supplementary Figure 44.
UB3LYP-D3/6-31G(d)-SDD(Cu)
Zero-point correction= 0.586009 (Hartree/Particle)
Thermal correction to Energy= 0.627097
Thermal correction to Enthalpy= 0.628041
Thermal correction to Gibbs Free Energy= 0.507649
Sum of electronic and zero-point Energies= -2453.574757
Sum of electronic and thermal Energies= -2453.533669

Sum of electronic and thermal Enthalpies= -2453.532725
Sum of electronic and thermal Free Energies= -2453.653117

UM06/6-311++G(d,p)-SDD(Cu)//UB3LYP-D3/6-31G(d)-SDD(Cu)
HF = -2453.3551679

[20-21]-OMe

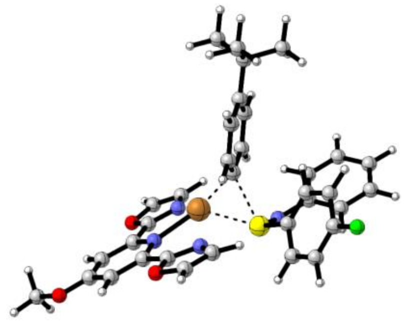

Supplementary Figure 45.
UB3LYP-D3/6-31G(d)-SDD(Cu)
Imaginary frequency = -133.33 cm^{-1}
Zero-point correction= 0.586087 (Hartree/Particle)
Thermal correction to Energy= 0.626700
Thermal correction to Enthalpy= 0.627644
Thermal correction to Gibbs Free Energy= 0.506181
Sum of electronic and zero-point Energies= -2453.564043
Sum of electronic and thermal Energies= -2453.523430
Sum of electronic and thermal Enthalpies= -2453.522486
Sum of electronic and thermal Free Energies= -2453.643950

UM06/6-311++G(d,p)-SDD(Cu)//UB3LYP-D3/6-31G(d)-SDD(Cu)
HF = -2453.3491699

21-OMe

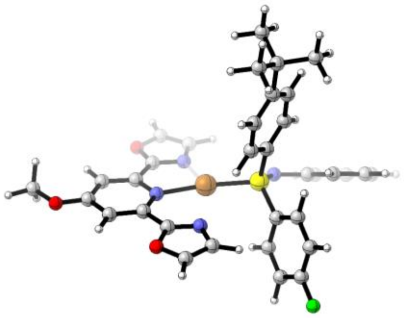

Supplementary Figure 46.
UB3LYP-D3/6-31G(d)-SDD(Cu)
Zero-point correction= 0.587876 (Hartree/Particle)

Thermal correction to Energy= 0.628693
Thermal correction to Enthalpy= 0.629637
Thermal correction to Gibbs Free Energy= 0.508171
Sum of electronic and zero-point Energies= -2453.588820
Sum of electronic and thermal Energies= -2453.548003
Sum of electronic and thermal Enthalpies= -2453.547059
Sum of electronic and thermal Free Energies= -2453.668525

UM06/6-311++G(d,p)-SDD(Cu)//UB3LYP-D3/6-31G(d)-SDD(Cu)
HF = -2453.3747678

20'-OMe

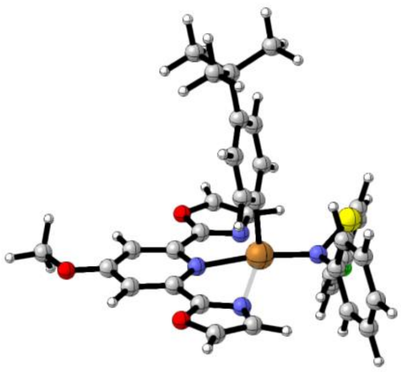

Supplementary Figure 47.
UB3LYP-D3/6-31G(d)-SDD(Cu)
Zero-point correction= 0.586950 (Hartree/Particle)
Thermal correction to Energy= 0.627631
Thermal correction to Enthalpy= 0.628576
Thermal correction to Gibbs Free Energy= 0.509658
Sum of electronic and zero-point Energies= -2453.590269
Sum of electronic and thermal Energies= -2453.549587
Sum of electronic and thermal Enthalpies= -2453.548642
Sum of electronic and thermal Free Energies= -2453.667560

UM06/6-311++G(d,p)-SDD(Cu)//UB3LYP-D3/6-31G(d)-SDD(Cu)
HF = -2453.3654693

[20'-21']-OMe

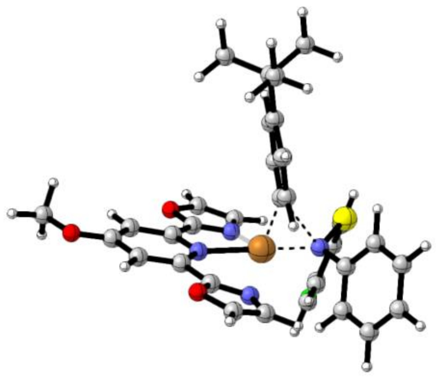

Supplementary Figure 48.
UB3LYP-D3/6-31G(d)-SDD(Cu)
Imaginary frequency = -140.71 cm^{-1}
Zero-point correction= 0.586401 (Hartree/Particle)
Thermal correction to Energy= 0.626506
Thermal correction to Enthalpy= 0.627450
Thermal correction to Gibbs Free Energy= 0.510002
Sum of electronic and zero-point Energies= -2453.588685
Sum of electronic and thermal Energies= -2453.548580
Sum of electronic and thermal Enthalpies= -2453.547636
Sum of electronic and thermal Free Energies= -2453.665083

UM06/6-311++G(d,p)-SDD(Cu)//UB3LYP-D3/6-31G(d)-SDD(Cu)
HF = -2453.3653084

21'-OMe

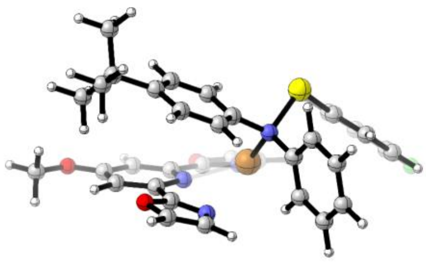

Supplementary Figure 49.
UB3LYP-D3/6-31G(d)-SDD(Cu)
Zero-point correction= 0.589300 (Hartree/Particle)
Thermal correction to Energy= 0.629447
Thermal correction to Enthalpy= 0.630391
Thermal correction to Gibbs Free Energy= 0.513558
Sum of electronic and zero-point Energies= -2453.647869
Sum of electronic and thermal Energies= -2453.607721
Sum of electronic and thermal Enthalpies= -2453.606777
Sum of electronic and thermal Free Energies= -2453.723611

UM06/6-311++G(d,p)-SDD(Cu)//UB3LYP-D3/6-31G(d)-SDD(Cu)
HF = -2453.4326028

4. Supplementary Figures

4.1 NMR Spectra

^{1}H NMR (400 MHz, $CDCl_3$) of *S*-(4-Fluorophenyl)-*N*-Phenyl-*N*-(*p*-tolyl)thiohydroxylamine (3aa)

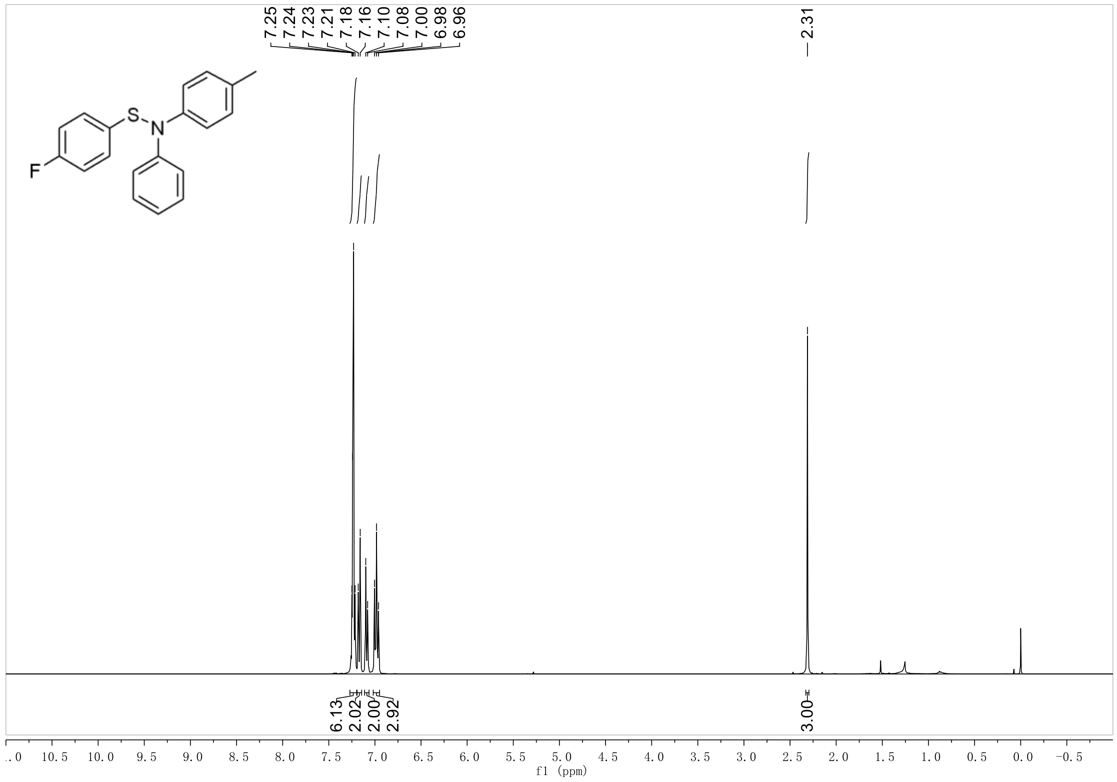

^{13}C NMR (100 MHz, $CDCl_3$) of *S*-(4-Fluorophenyl)-*N*-Phenyl-*N*-(*p*-tolyl)thiohydroxylamine (3aa)

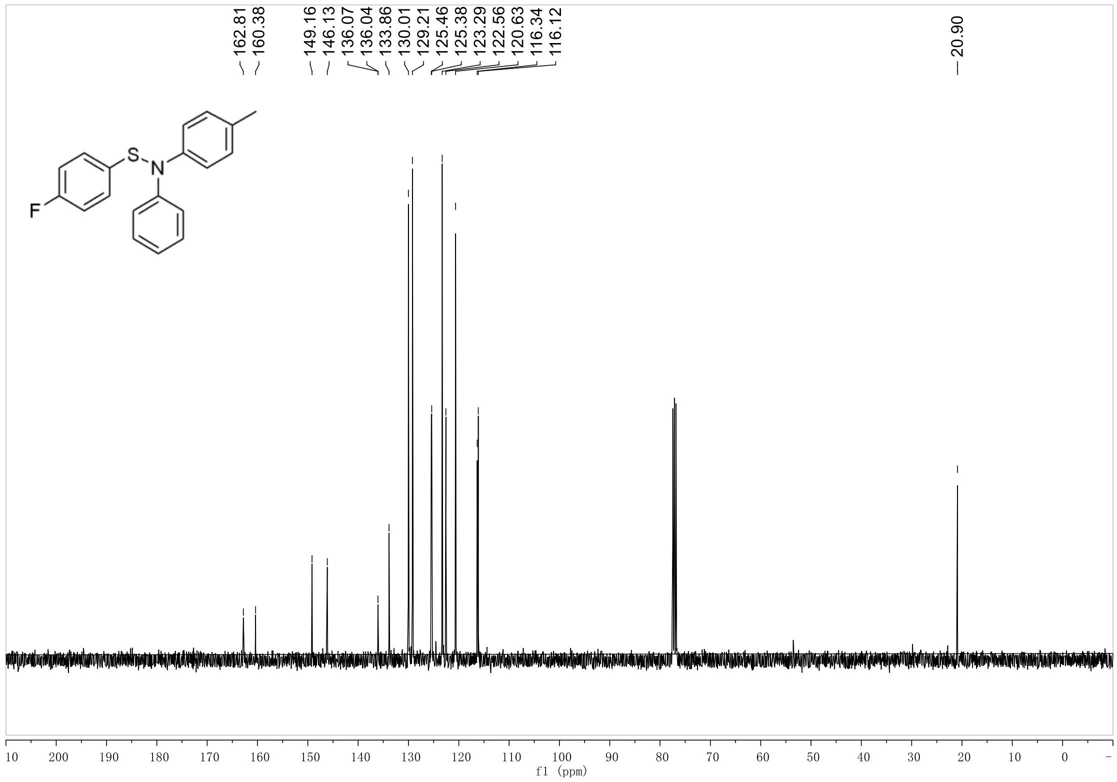

^{19}F NMR (376 MHz, $CDCl_3$) of *S*-(4-Fluorophenyl)-*N*-Phenyl-*N*-(*p*-tolyl)thiohydroxylamine (3aa)

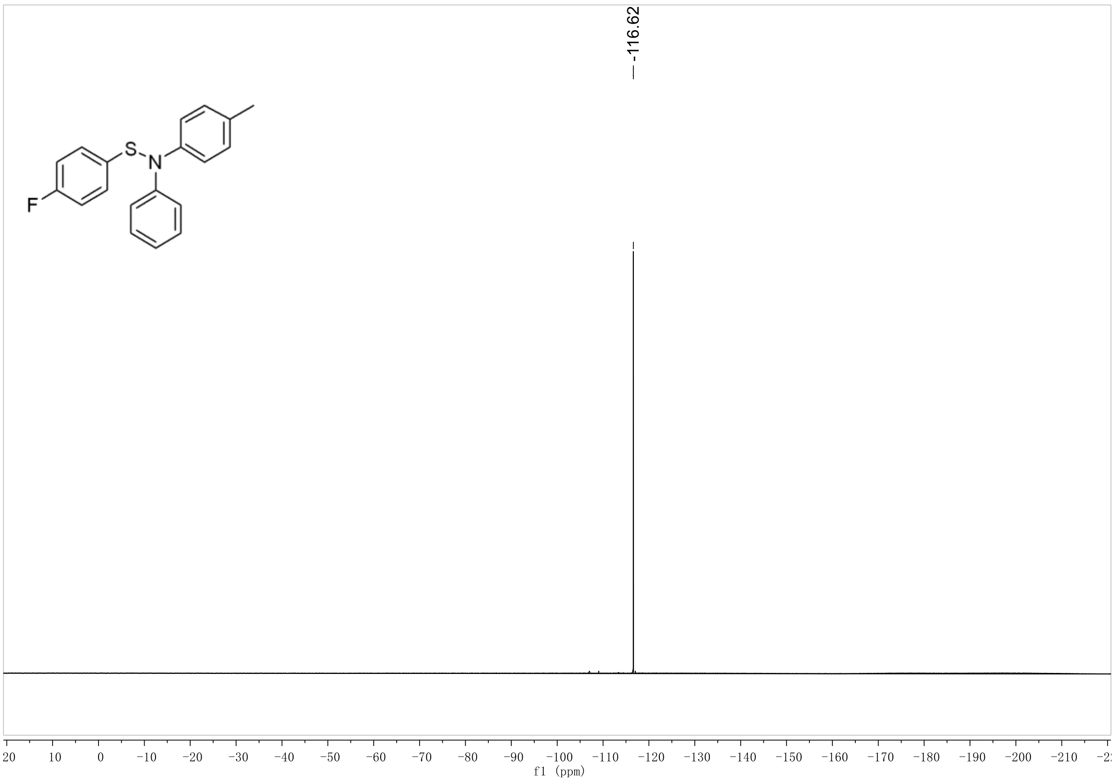

^{1}H NMR (400 MHz, CDCl$_3$) of *N*-Phenyl-*N,S*-di-*p*-tolylthiohydroxylamine (3ba)

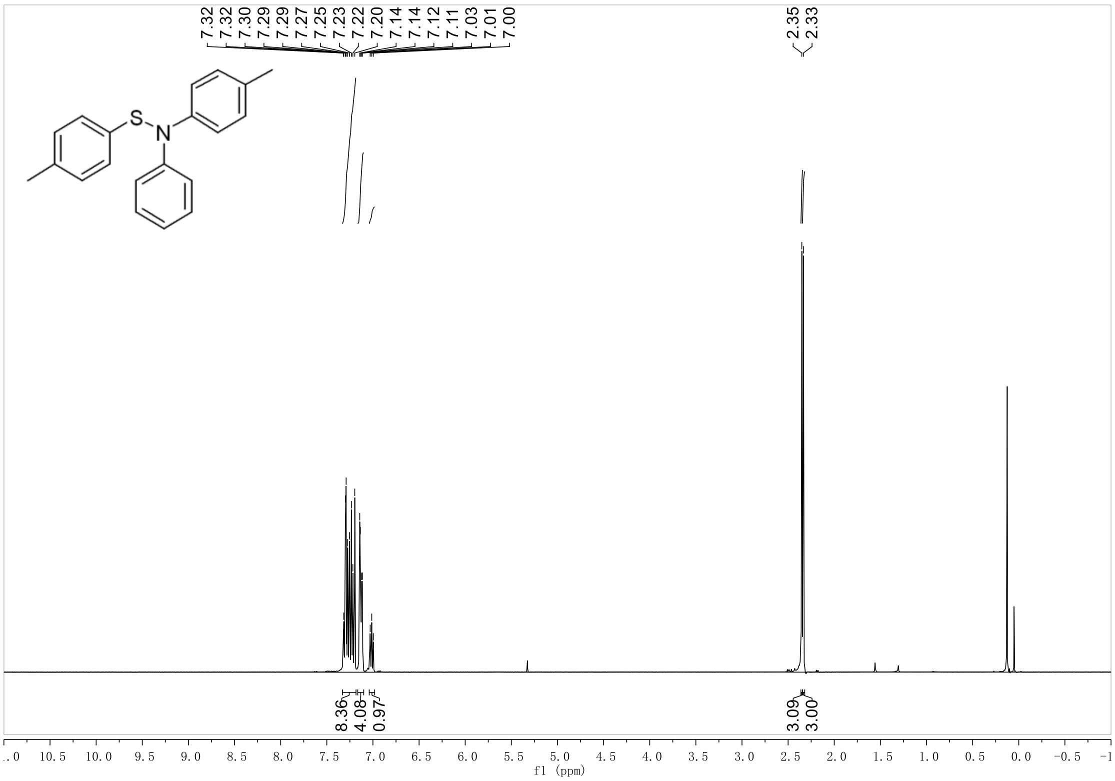

^{13}C NMR (100 MHz, CDCl$_3$) of *N*-Phenyl-*N,S*-di-*p*-tolylthiohydroxylamine (3ba)

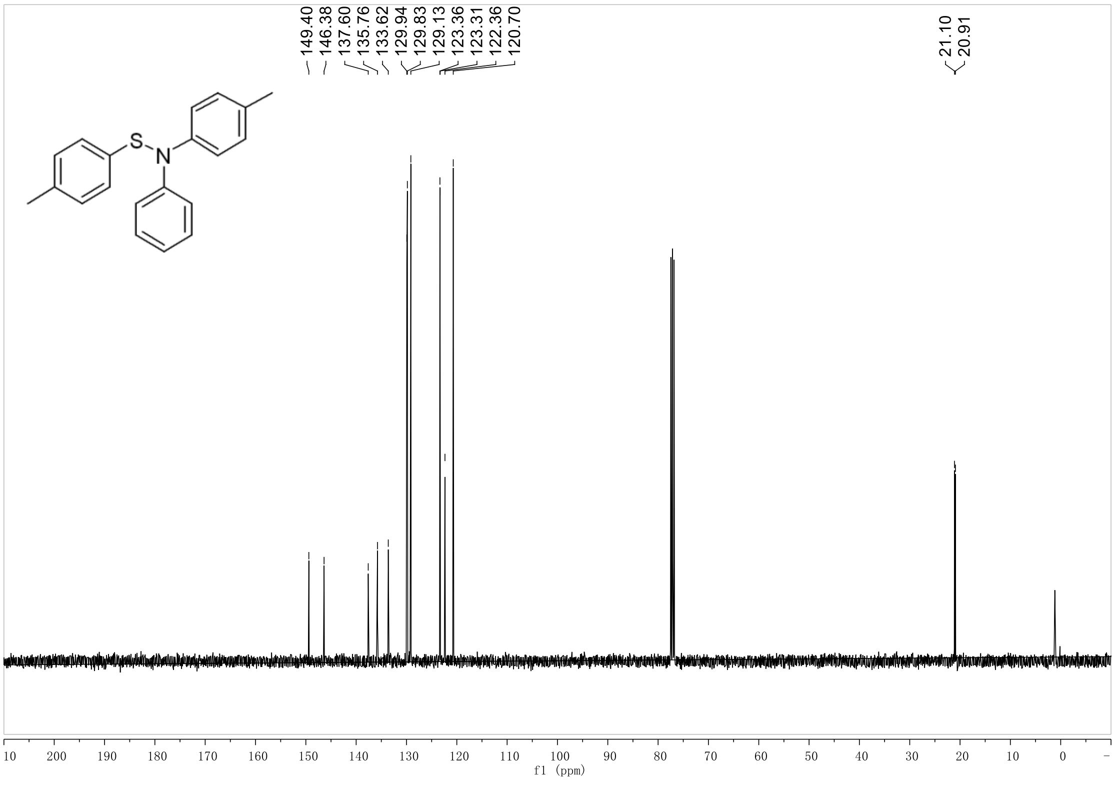

^{1}H NMR (400 MHz, $CDCl_3$) of *N*-(4-Methoxyphenyl)-*N*-phenyl-*S*-(*p*-tolyl)thiohydroxylamine (3bb)

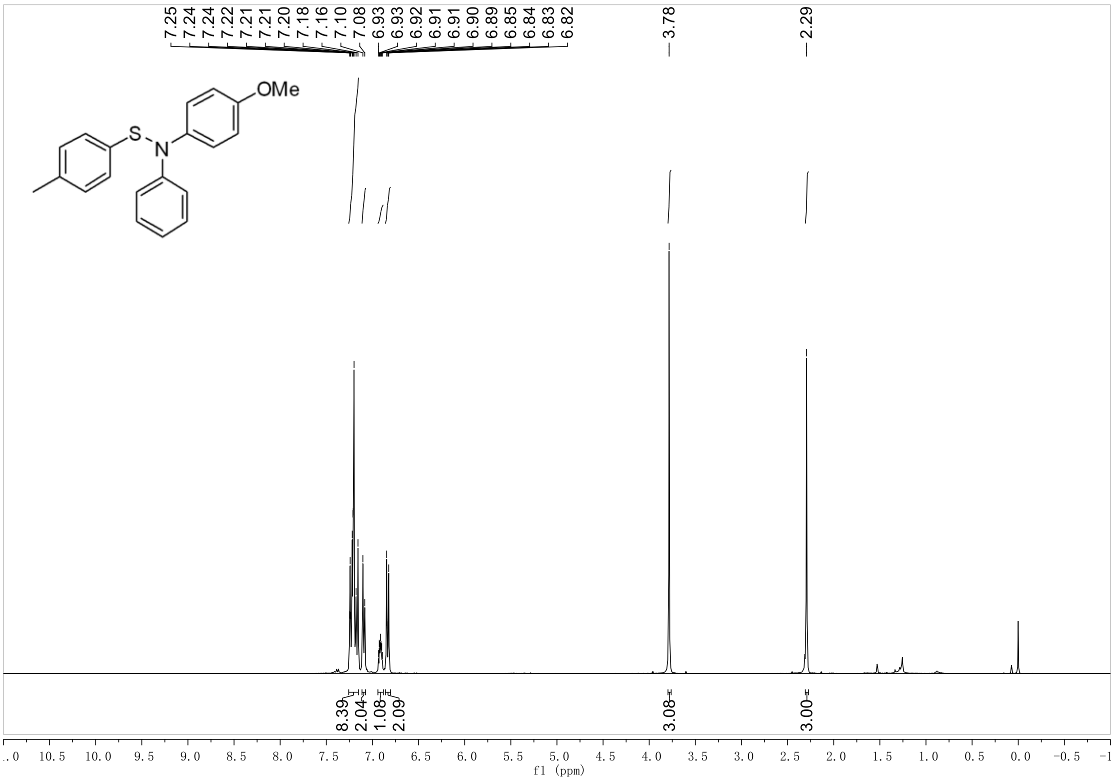

^{13}C NMR (100 MHz, $CDCl_3$) of *N*-(4-Methoxyphenyl)-*N*-phenyl-*S*-(*p*-tolyl)thiohydroxylamine (3bb)

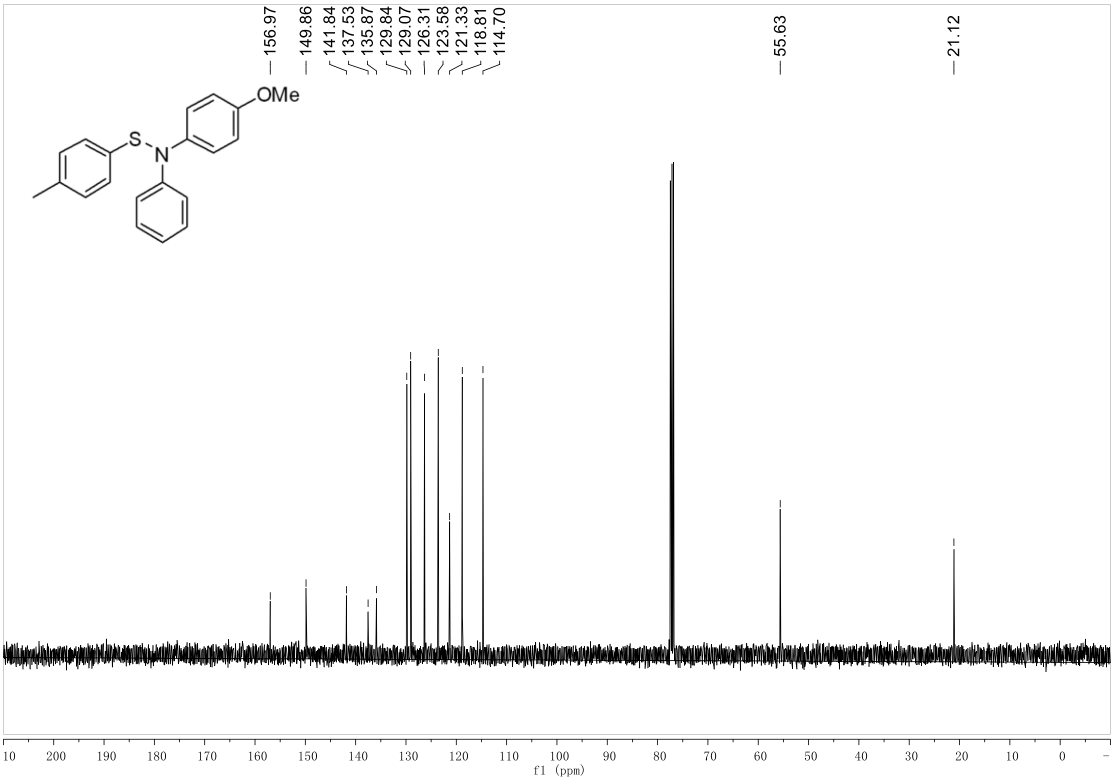

^{1}H NMR (400 MHz, $CDCl_3$) of *N*-(4-(Methylthio)phenyl)-*N*-phenyl-*S*-(*p*-tolyl)thiohydroxylamine (3bc)

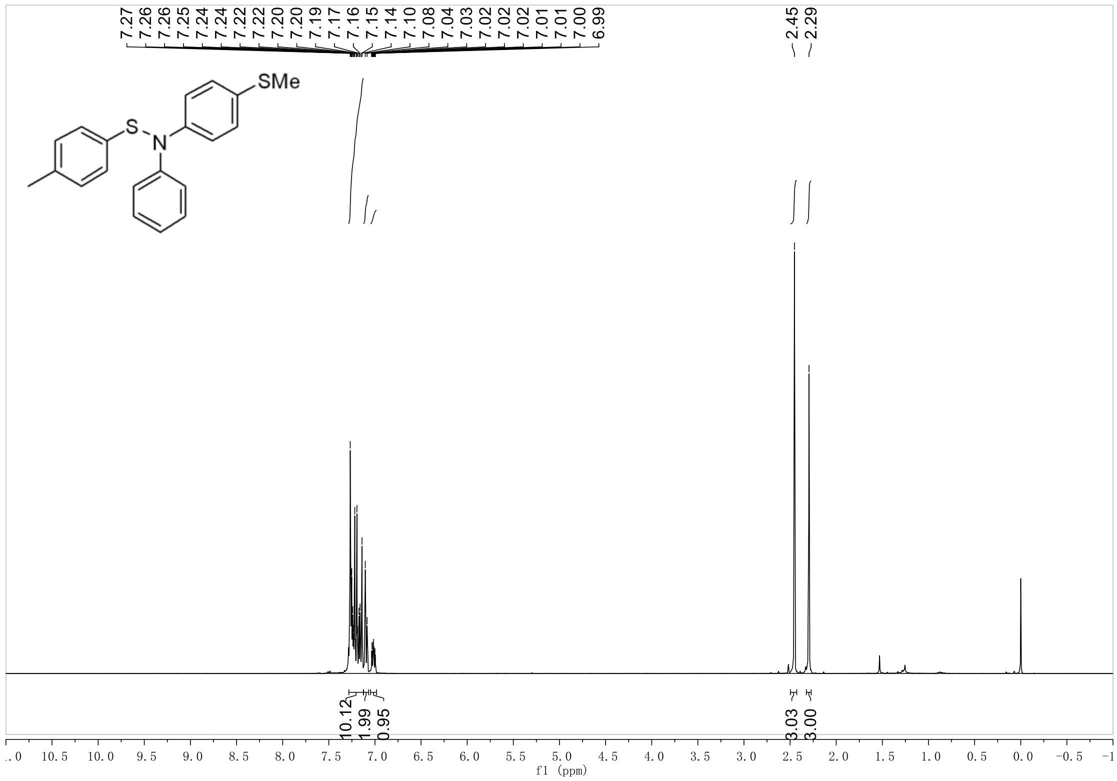

^{13}C NMR (100 MHz, $CDCl_3$) of *N*-(4-(Methylthio)phenyl)-*N*-phenyl-*S*-(*p*-tolyl)thiohydroxylamine (3bc)

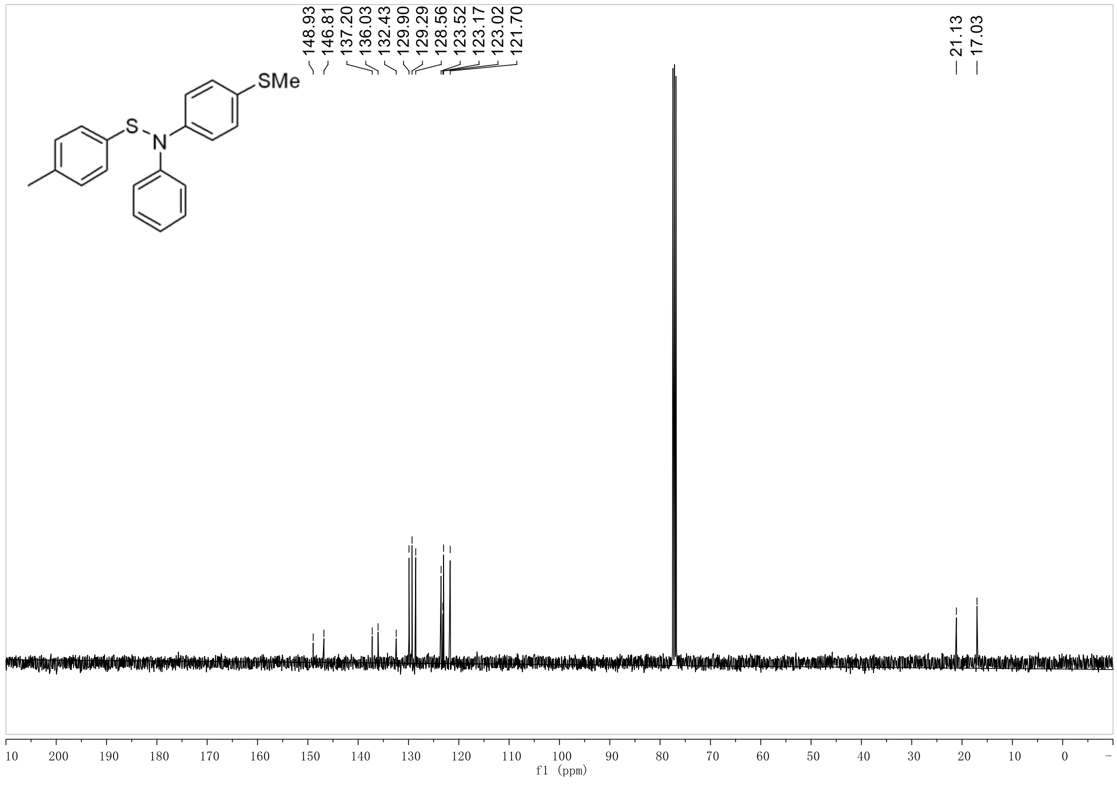

^{1}H NMR (400 MHz, $CDCl_3$) of *N*-(4-Fluorophenyl)-*N*-phenyl-*S*-(*p*-tolyl)thiohydroxylamine (3bd)

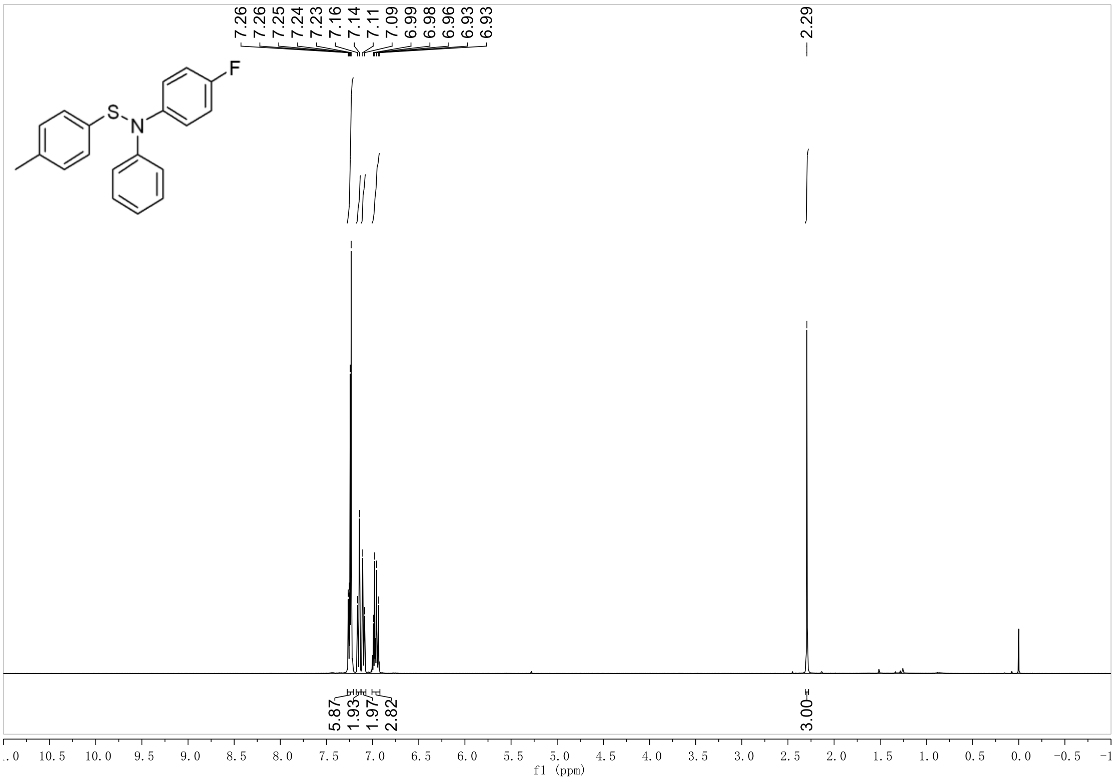

^{13}C NMR (100 MHz, $CDCl_3$) of *N*-(4-Fluorophenyl)-*N*-phenyl-*S*-(*p*-tolyl)thiohydroxylamine (3bd)

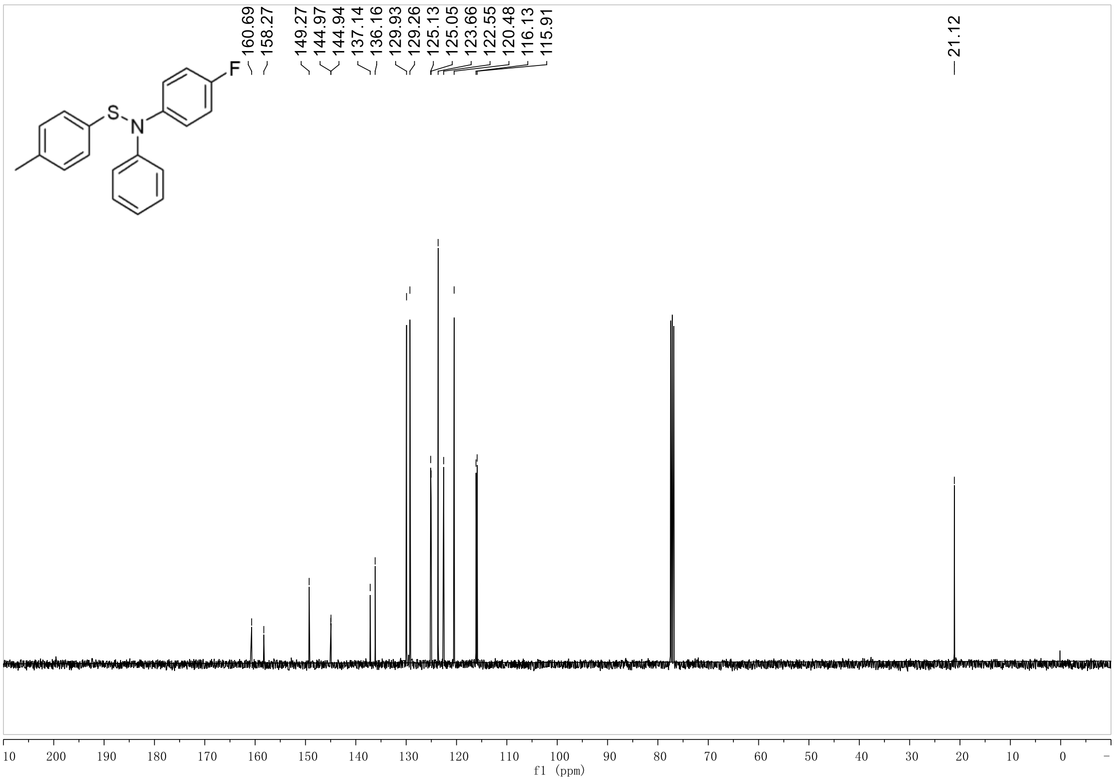

19F NMR (376 MHz, CDCl3) of *N*-(4-Fluorophenyl)-*N*-phenyl-*S*-(*p*-tolyl)thiohydroxylamine (3bd)

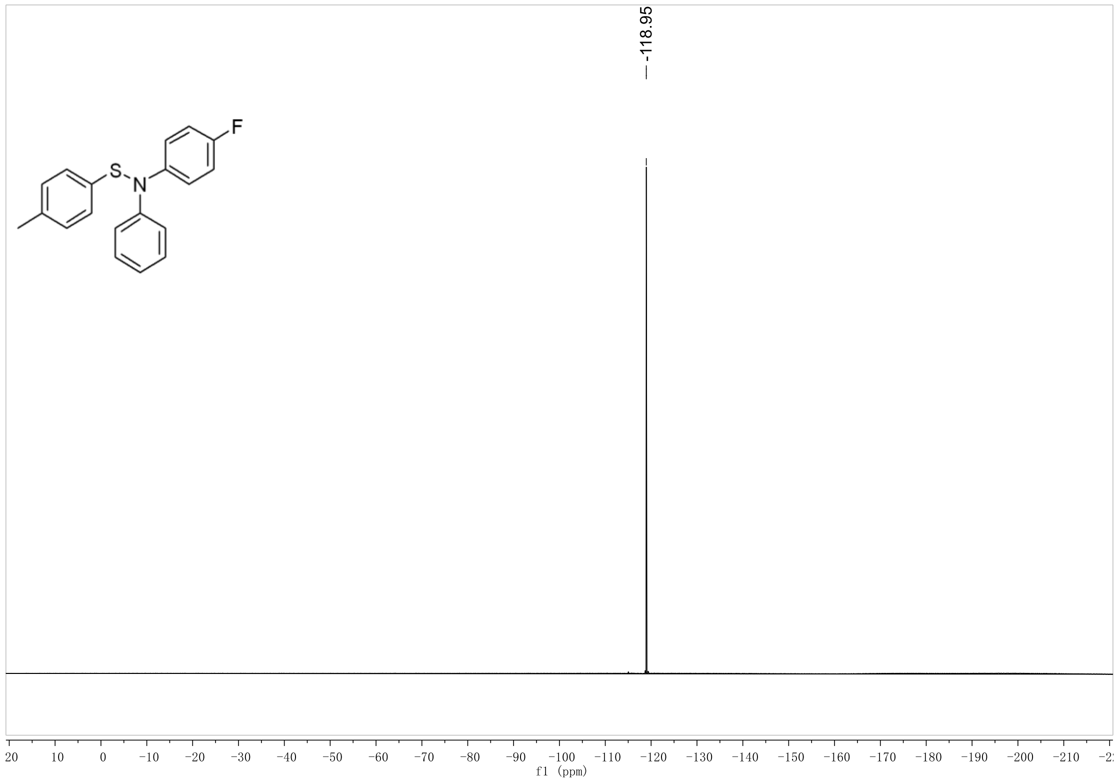

^{1}H NMR (400 MHz, $CDCl_3$) of *N*-(4-Chlorophenyl)-*N*-phenyl-*S*-(*p*-tolyl)thiohydroxylamine (3be)

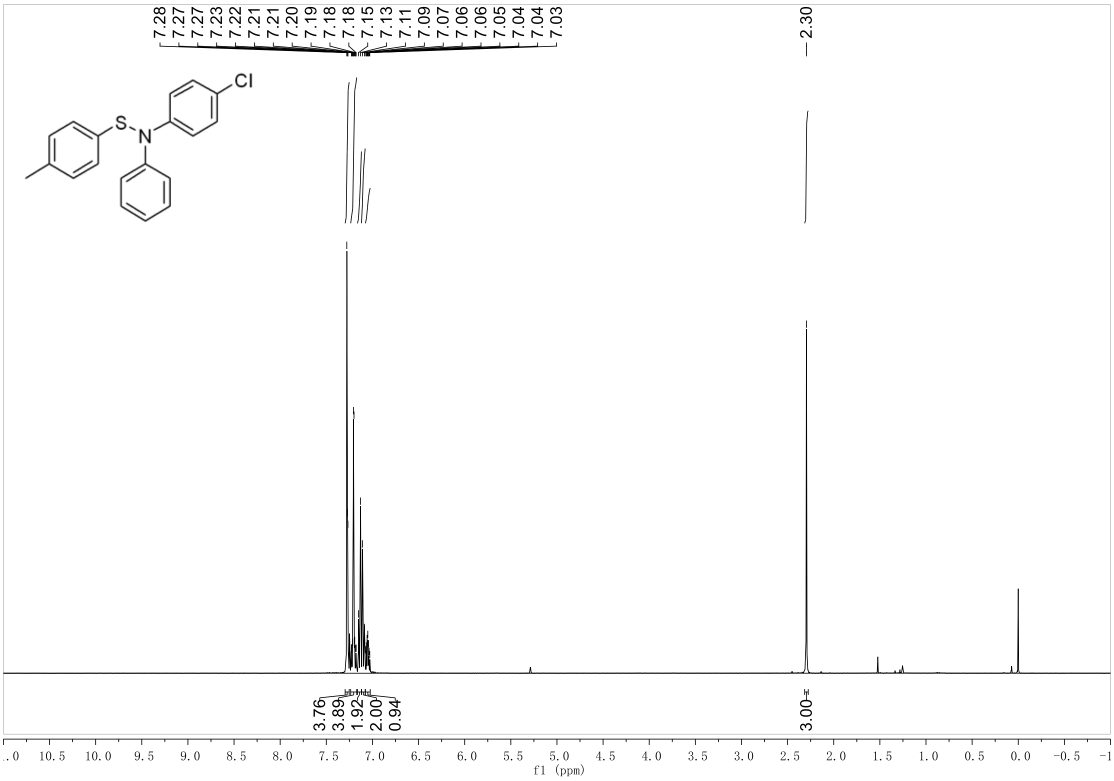

^{13}C NMR (100 MHz, $CDCl_3$) of *N*-(4-Chlorophenyl)-*N*-phenyl-*S*-(*p*-tolyl)thiohydroxylamine (3be)

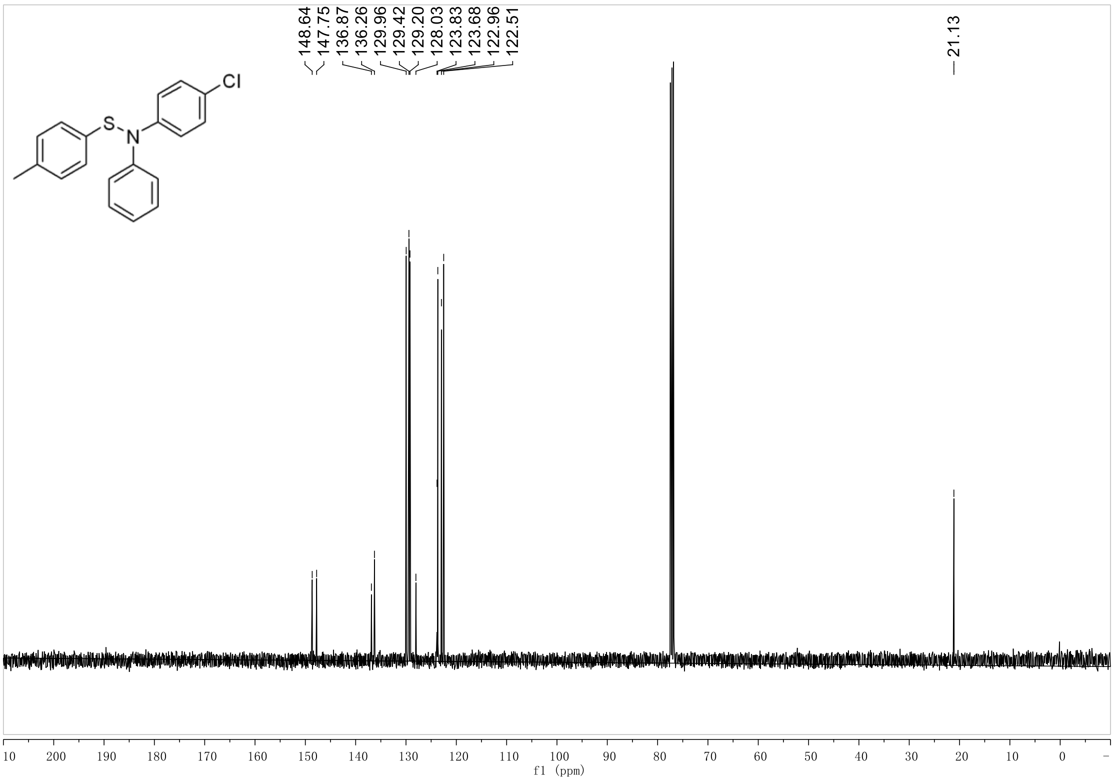

^{1}H NMR (400 MHz, $CDCl_3$) of *N*-Phenyl-*S*-(*p*-tolyl)-*N*-(4-(trifluoromethyl)phenyl)thiohydroxylamine (3bf)

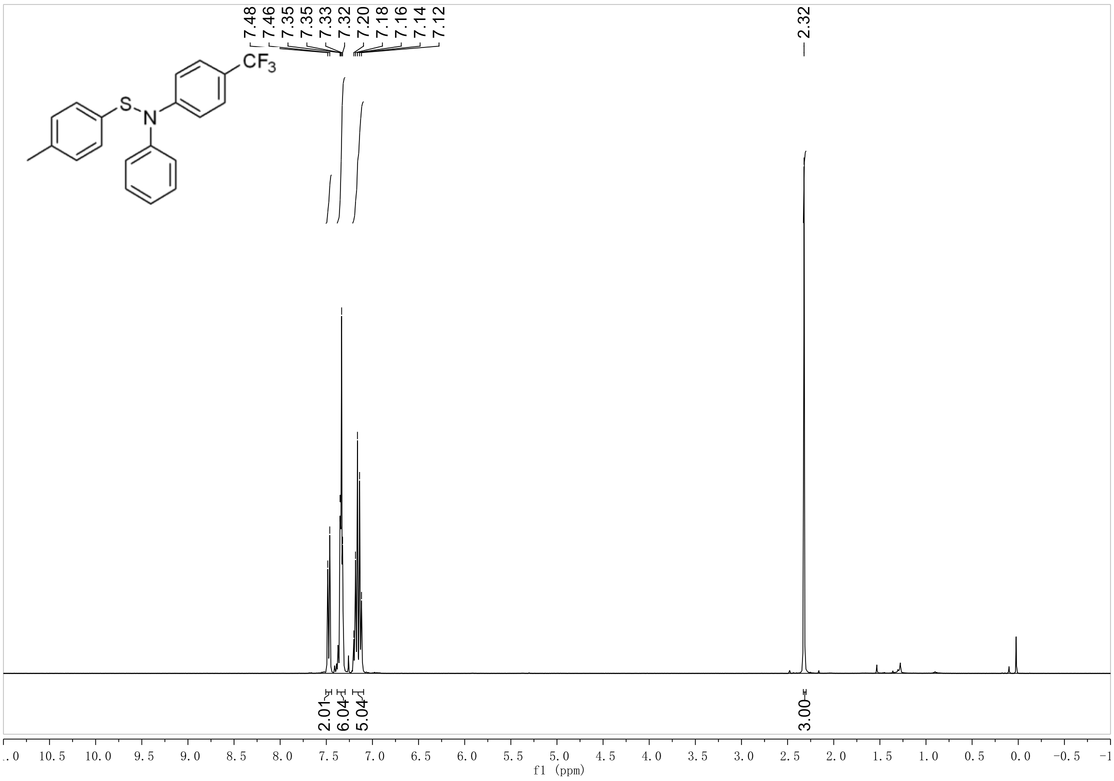

^{13}C NMR (100 MHz, $CDCl_3$) of *N*-Phenyl-*S*-(*p*-tolyl)-*N*-(4-(trifluoromethyl)phenyl)thiohydroxylamine (3bf)

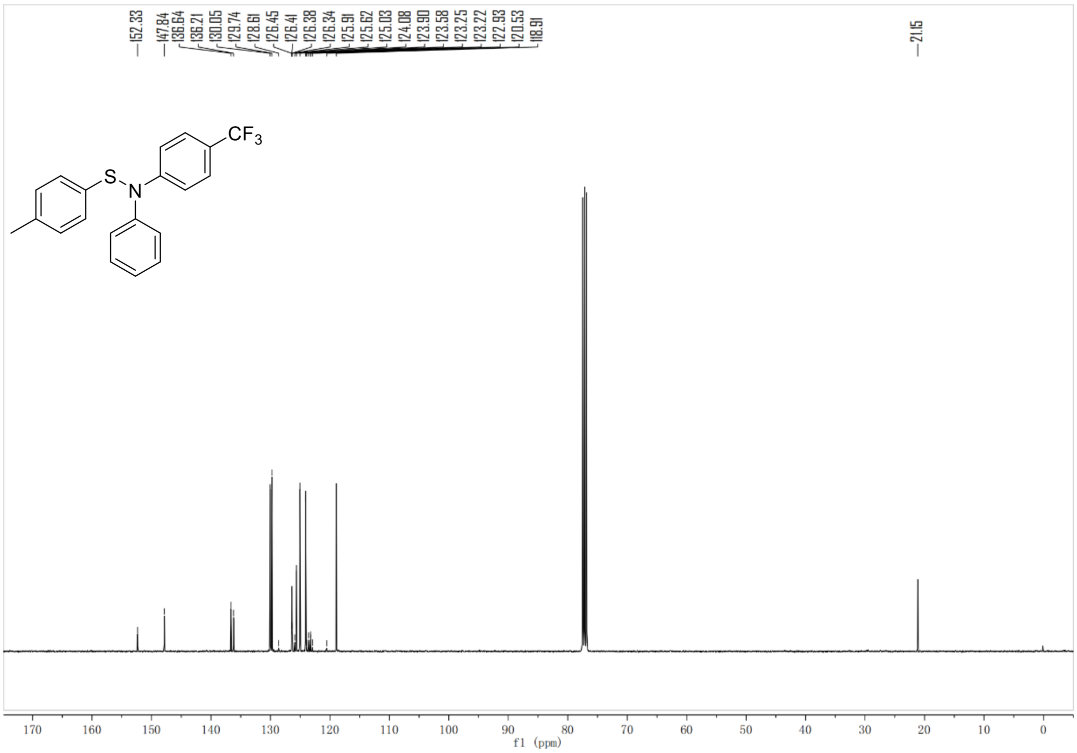

^{19}F NMR (376 MHz, $CDCl_3$) of *N*-Phenyl-*S*-(*p*-tolyl)-*N*-(4-(trifluoromethyl)phenyl)thiohydroxylamine (3bf)

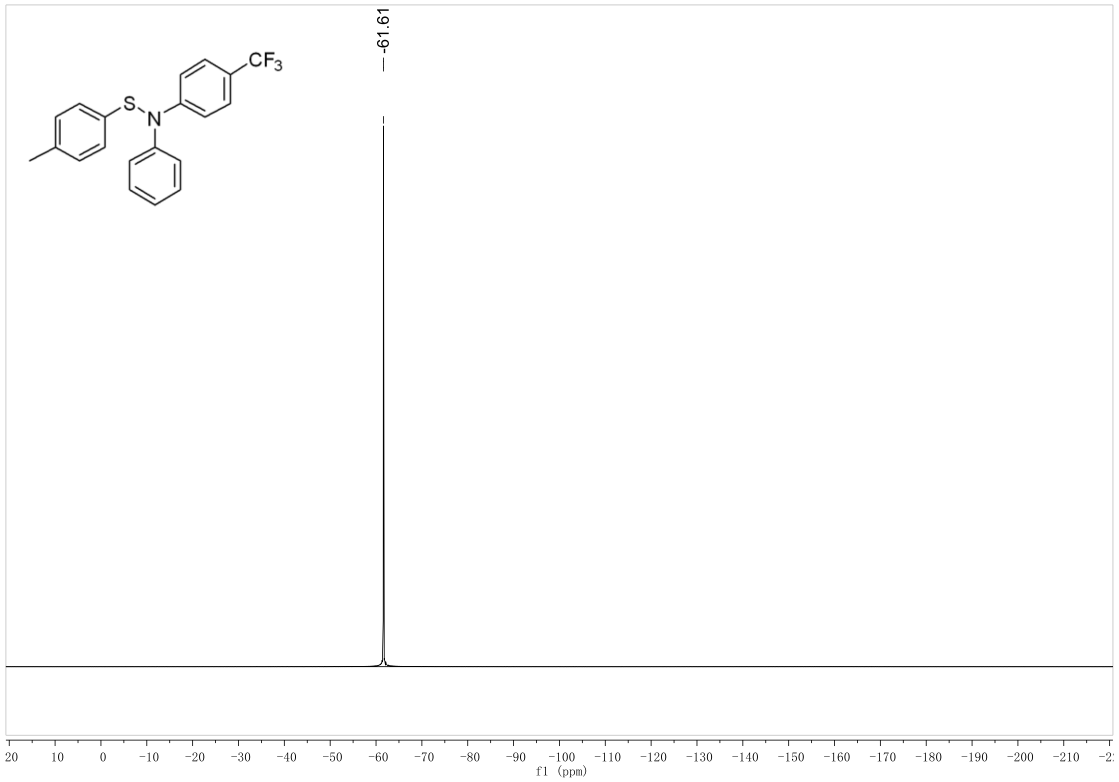

^{1}H NMR (400 MHz, $CDCl_3$) of 4-(Phenyl(p-tolylthio)amino)benzonitrile (3bg)

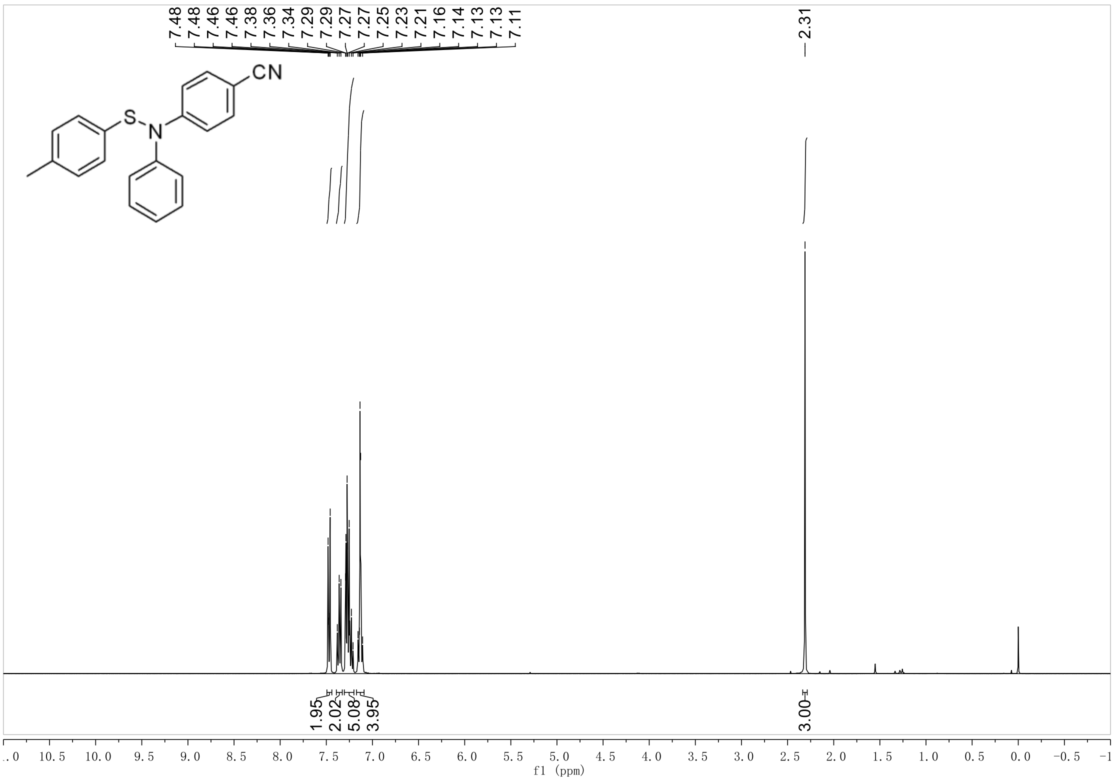

^{13}C NMR (100 MHz, $CDCl_3$) of 4-(Phenyl(p-tolylthio)amino)benzonitrile (3bg)

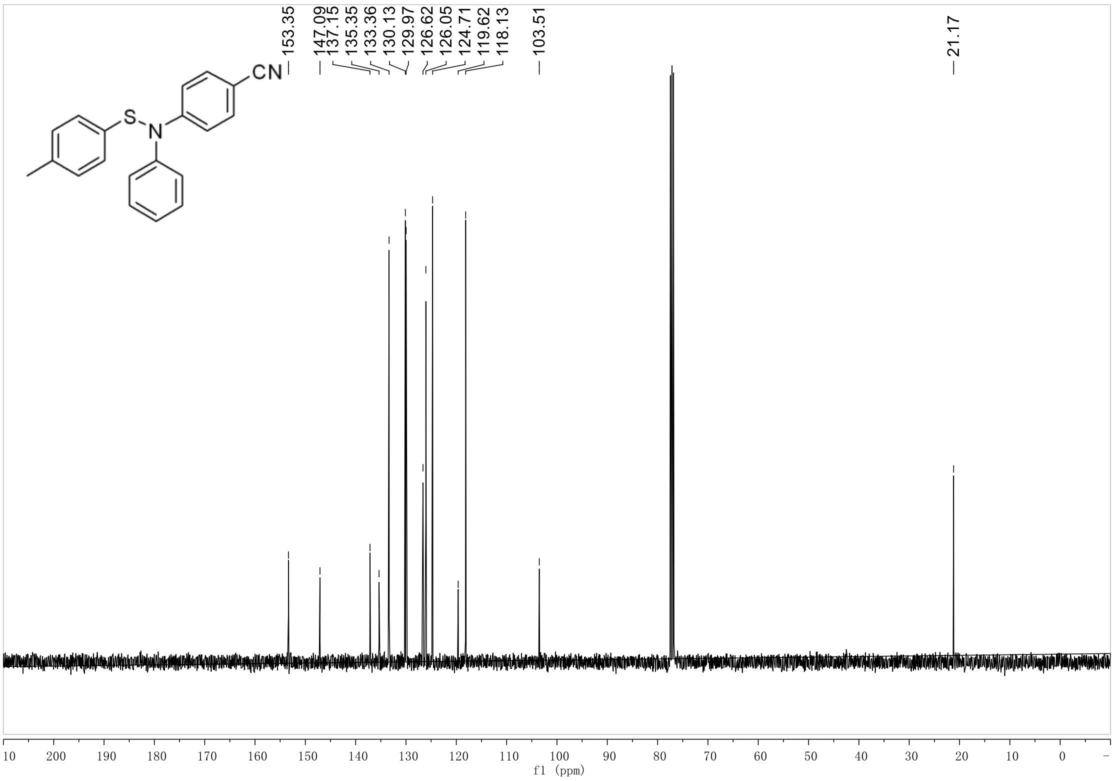

^{1}H NMR (400 MHz, $CDCl_3$) of *N*-Phenyl-*N*-(*o*-tolyl)-*S*-(*p*-tolyl)thiohydroxylamine (3bh)

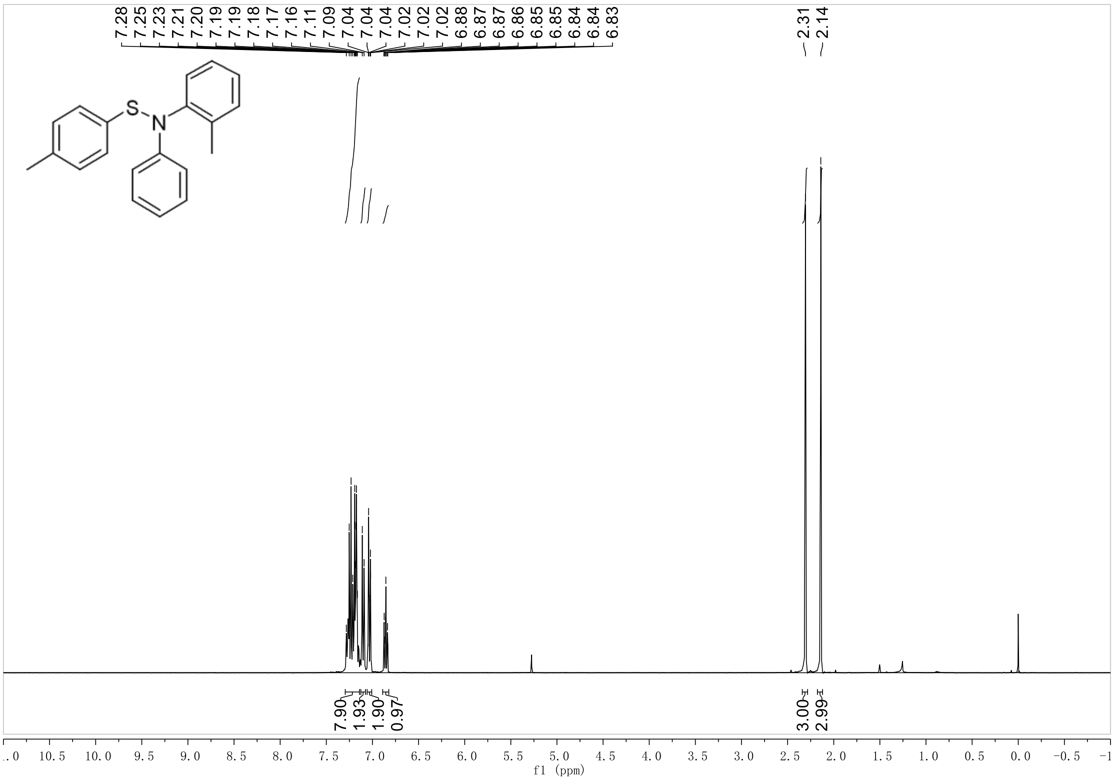

^{13}C NMR (100 MHz, $CDCl_3$) of *N*-Phenyl-*N*-(*o*-tolyl)-*S*-(*p*-tolyl)thiohydroxylamine (3bh)

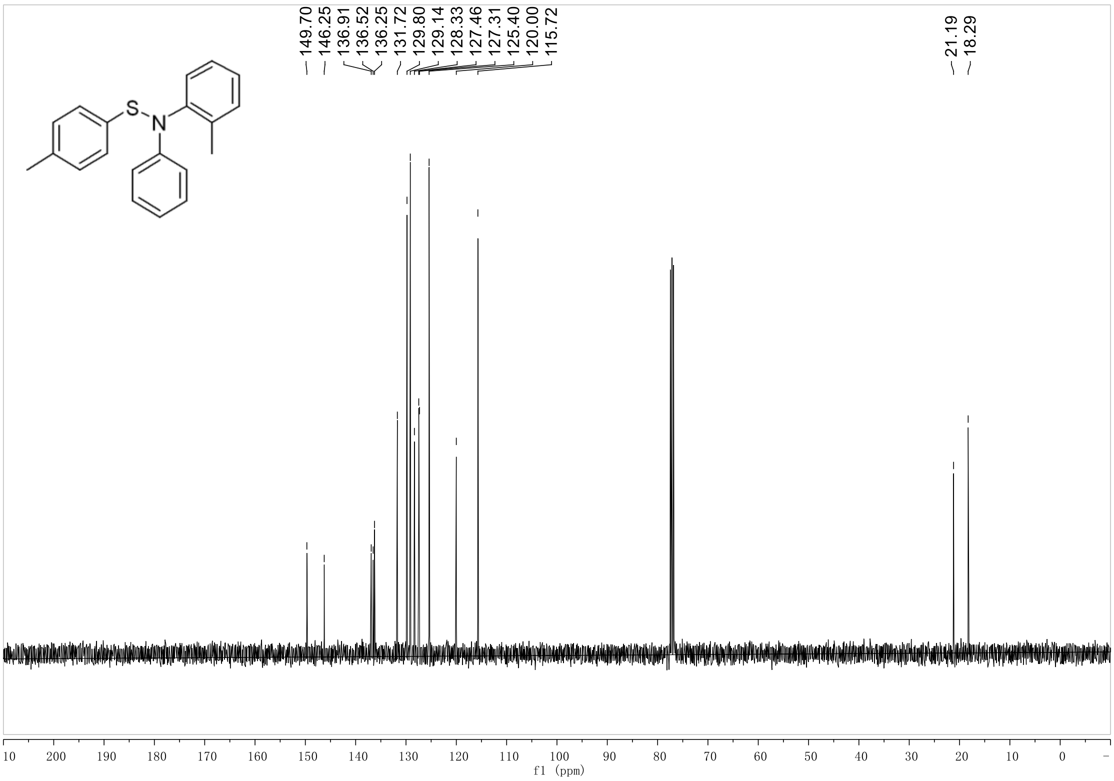

^{1}H NMR (400 MHz, $CDCl_3$) of *N*-(3-Methoxyphenyl)-*N*-phenyl-*S*-(*p*-tolyl)thiohydroxylamine (3bi)

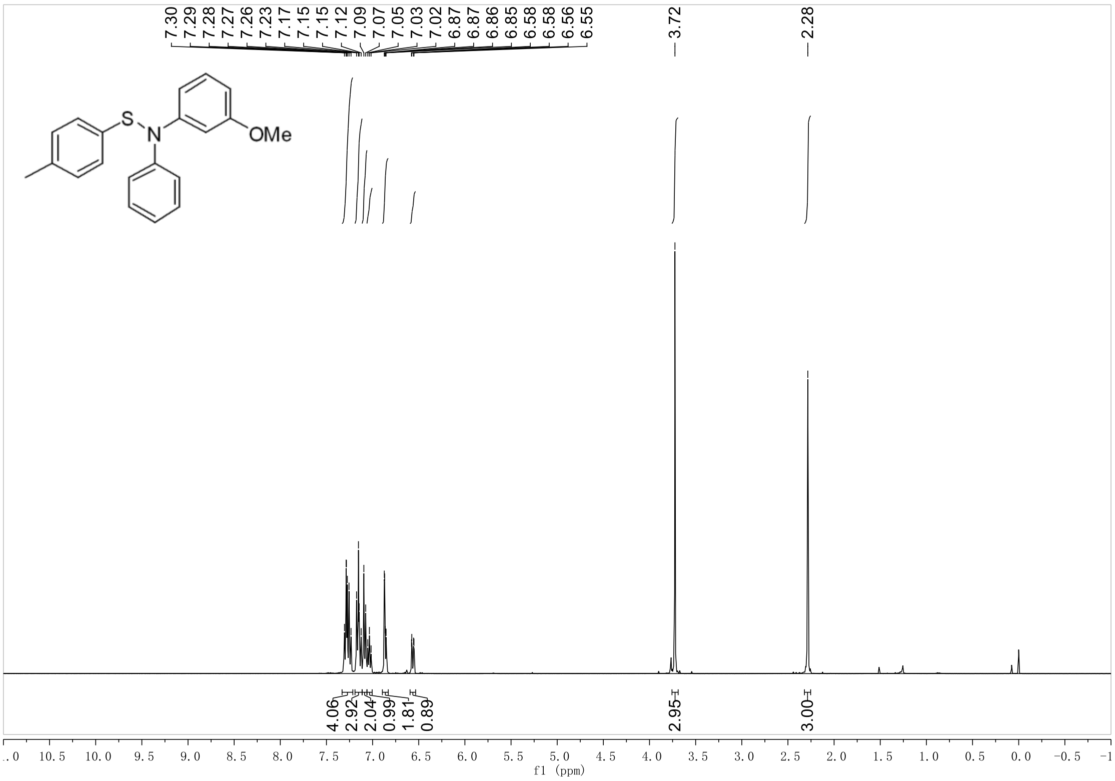

^{13}C NMR (100 MHz, $CDCl_3$) of *N*-(3-Methoxyphenyl)-*N*-phenyl-*S*-(*p*-tolyl)thiohydroxylamine (3bi)

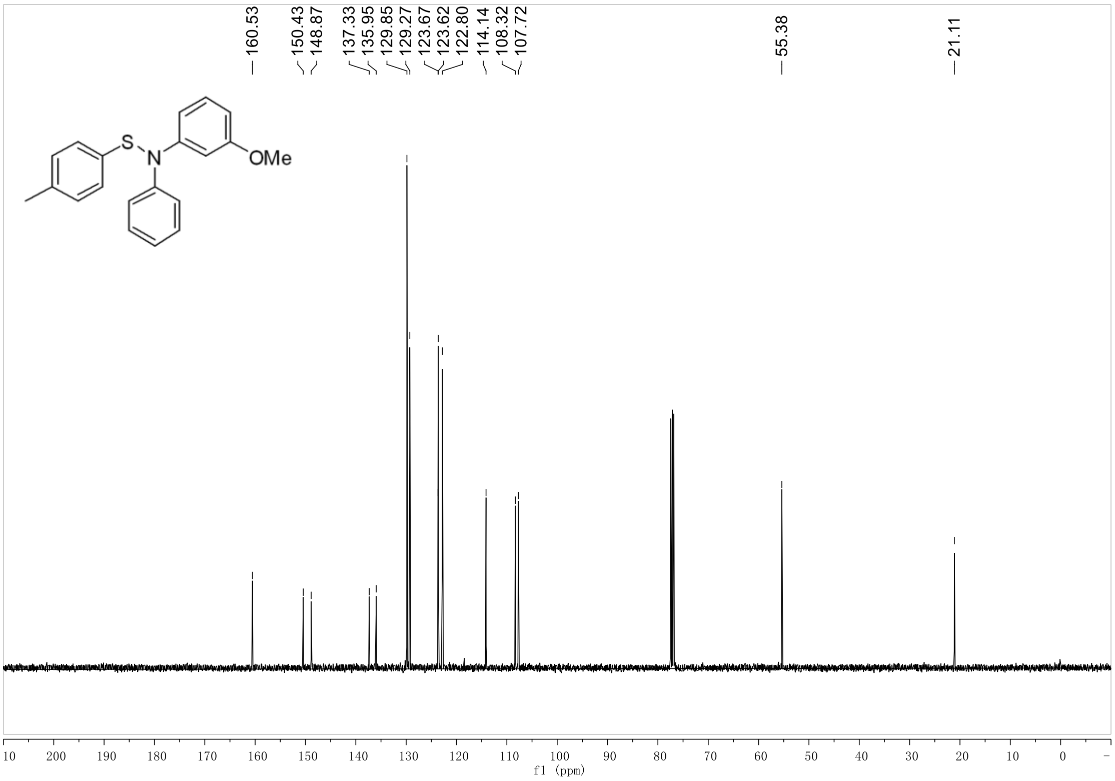

^{1}H NMR (400 MHz, $CDCl_3$) of *N*-(3-Nitrophenyl)-*N*-phenyl-*S*-(*p*-tolyl)thiohydroxylamine (3bj)

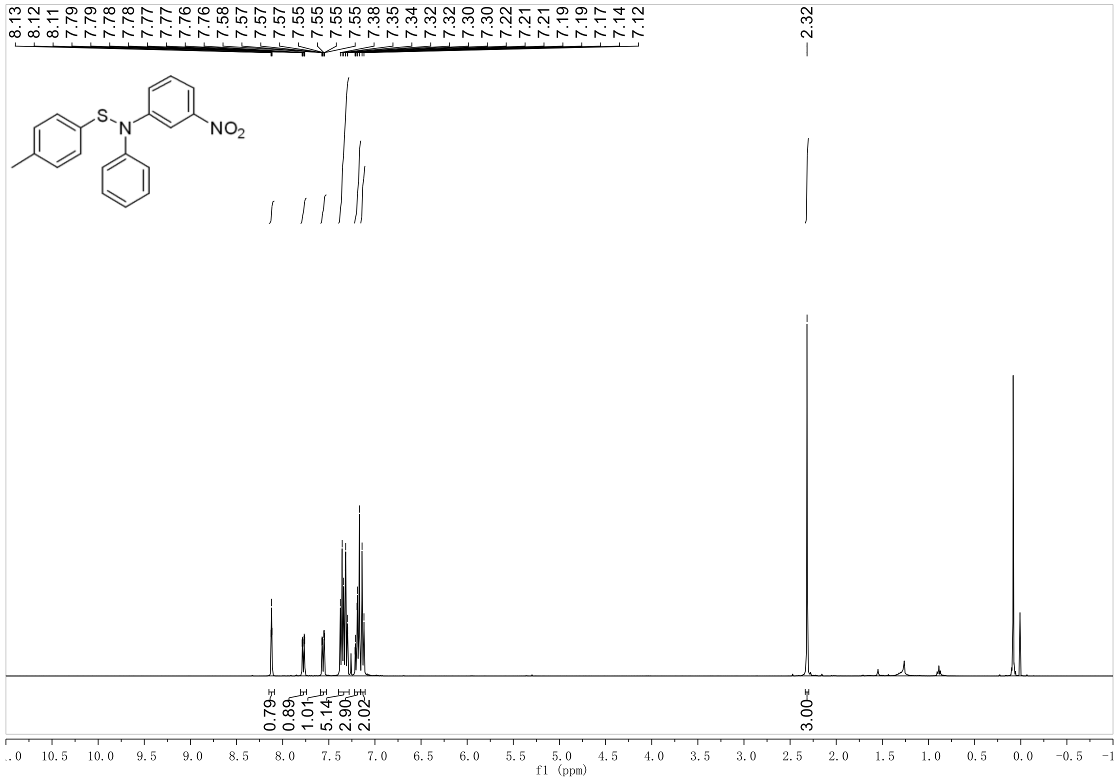

^{13}C NMR (100 MHz, $CDCl_3$) of *N*-(3-Nitrophenyl)-*N*-phenyl-*S*-(*p*-tolyl)thiohydroxylamine (3bj)

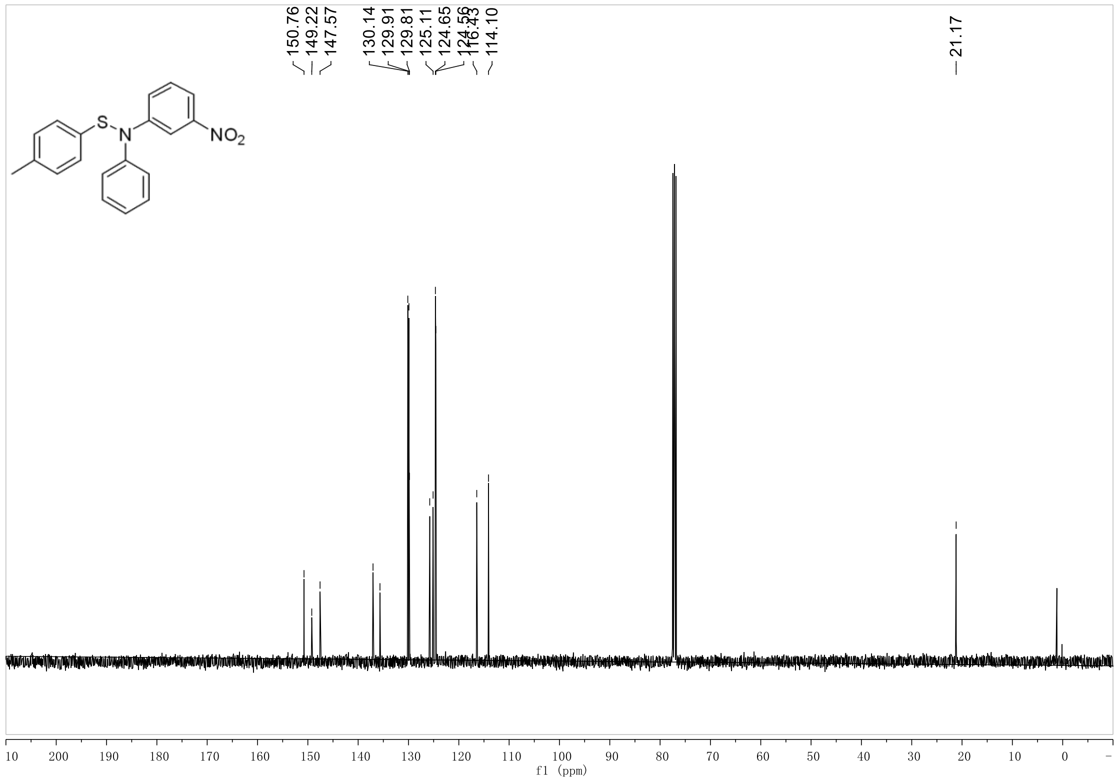

^{1}H NMR (400 MHz, $CDCl_3$) of 1-(4-(Phenyl(*p*-tolylthio)amino)phenyl)ethan-1-one (3bk)

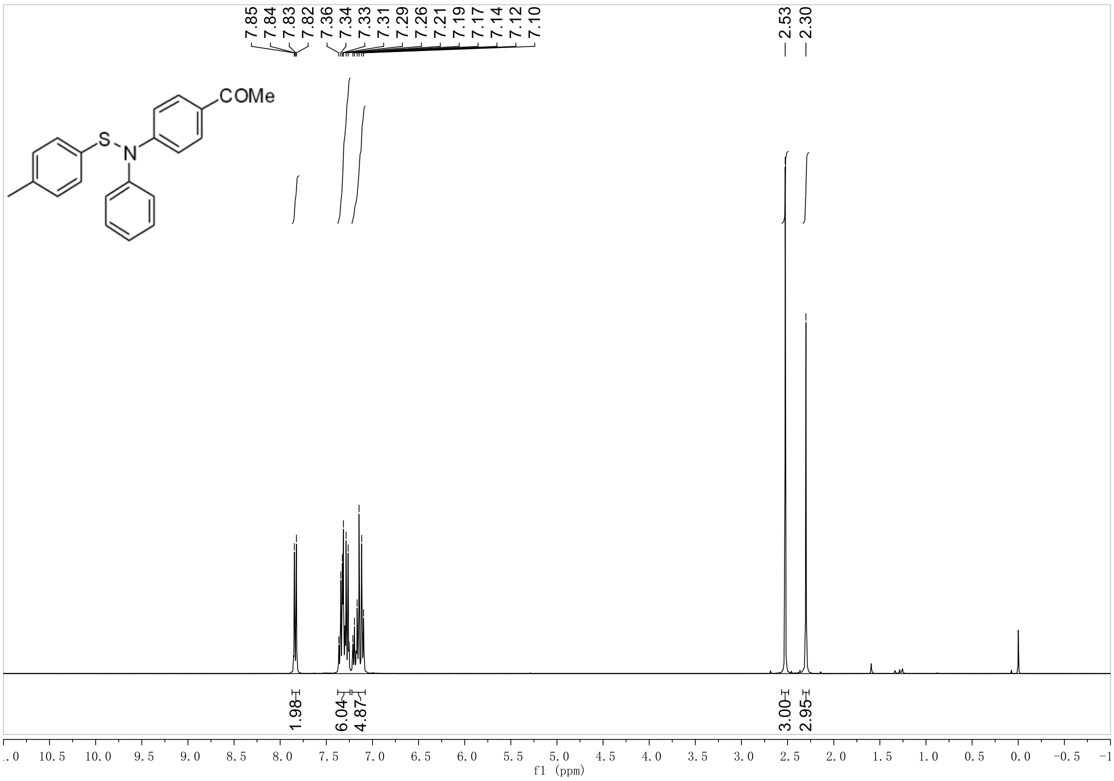

^{13}C NMR (100 MHz, $CDCl_3$) of 1-(4-(Phenyl(*p*-tolylthio)amino)phenyl)ethan-1-one (3bk)

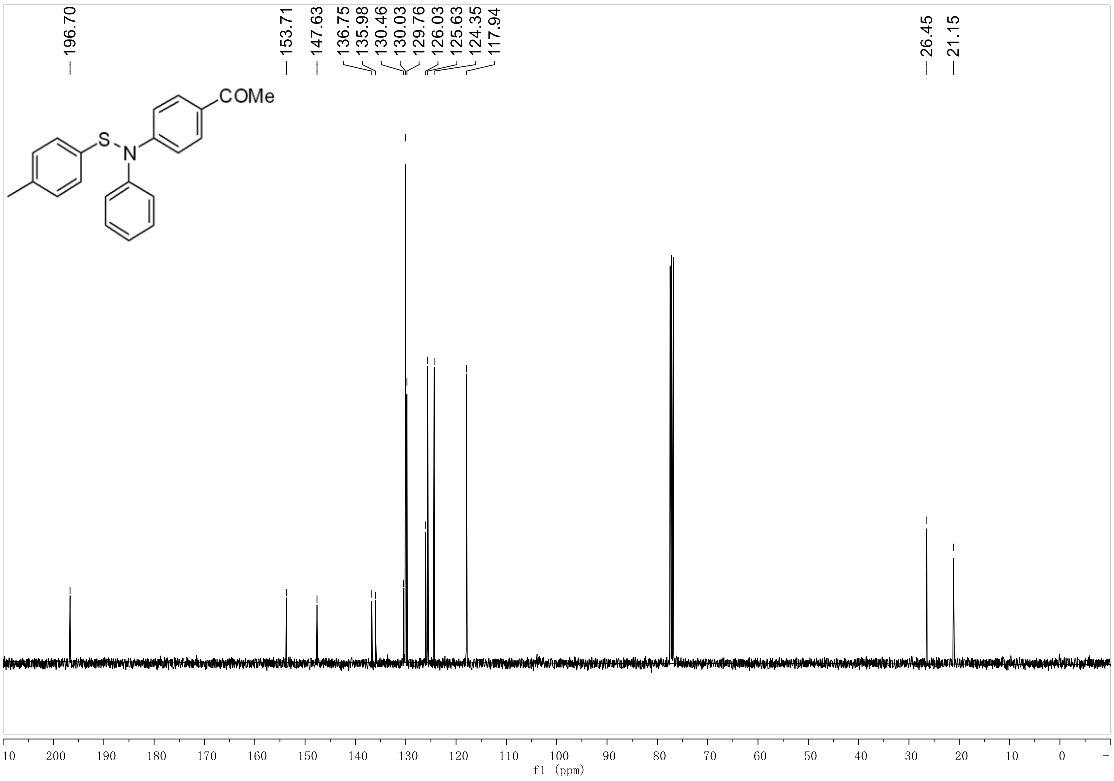

^{1}H NMR (400 MHz, $CDCl_3$) of Methyl 4-(phenyl(*p*-tolylthio)amino)benzoate (3bl)

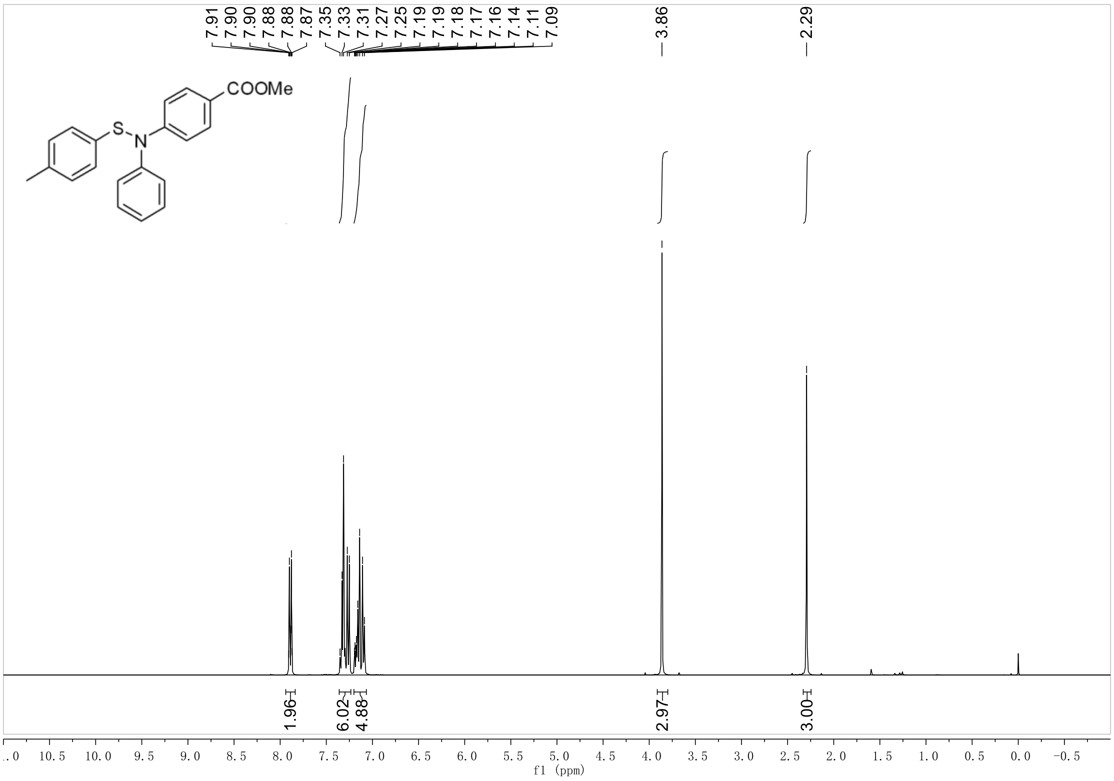

^{13}C NMR (100 MHz, $CDCl_3$) of Methyl 4-(phenyl(*p*-tolylthio)amino)benzoate (3bl)

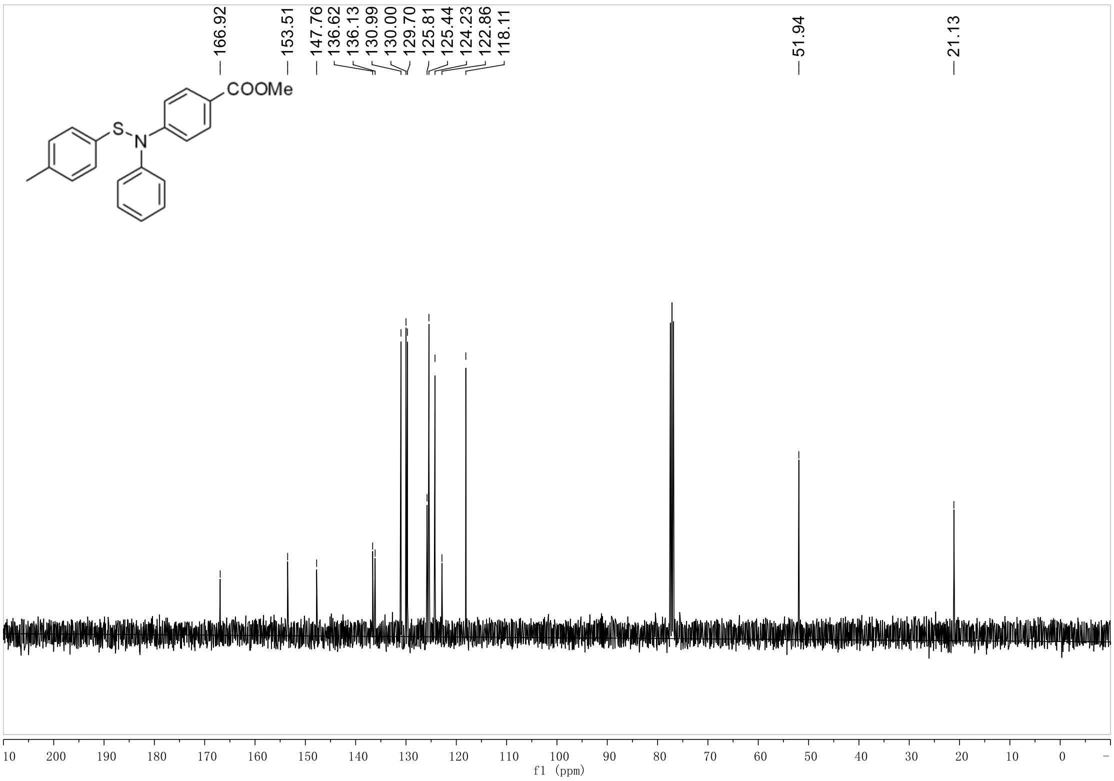

^{1}H NMR (400 MHz, $CDCl_3$) of *N*-Phenyl-*S*-(*p*-tolyl)-*N*-(4-vinylphenyl)thiohydroxylamine (3bm)

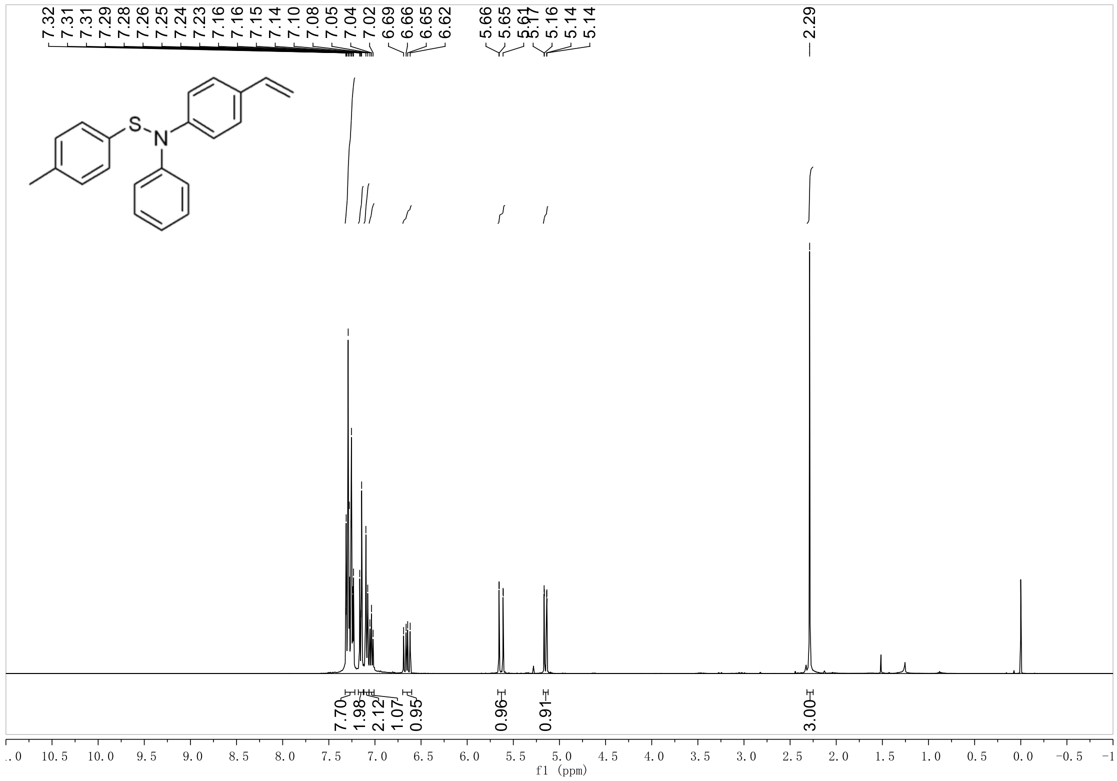

^{13}C NMR (100 MHz, $CDCl_3$) of *N*-Phenyl-*S*-(*p*-tolyl)-*N*-(4-(trifluoromethyl)phenyl)thiohydroxylamine (3bm)

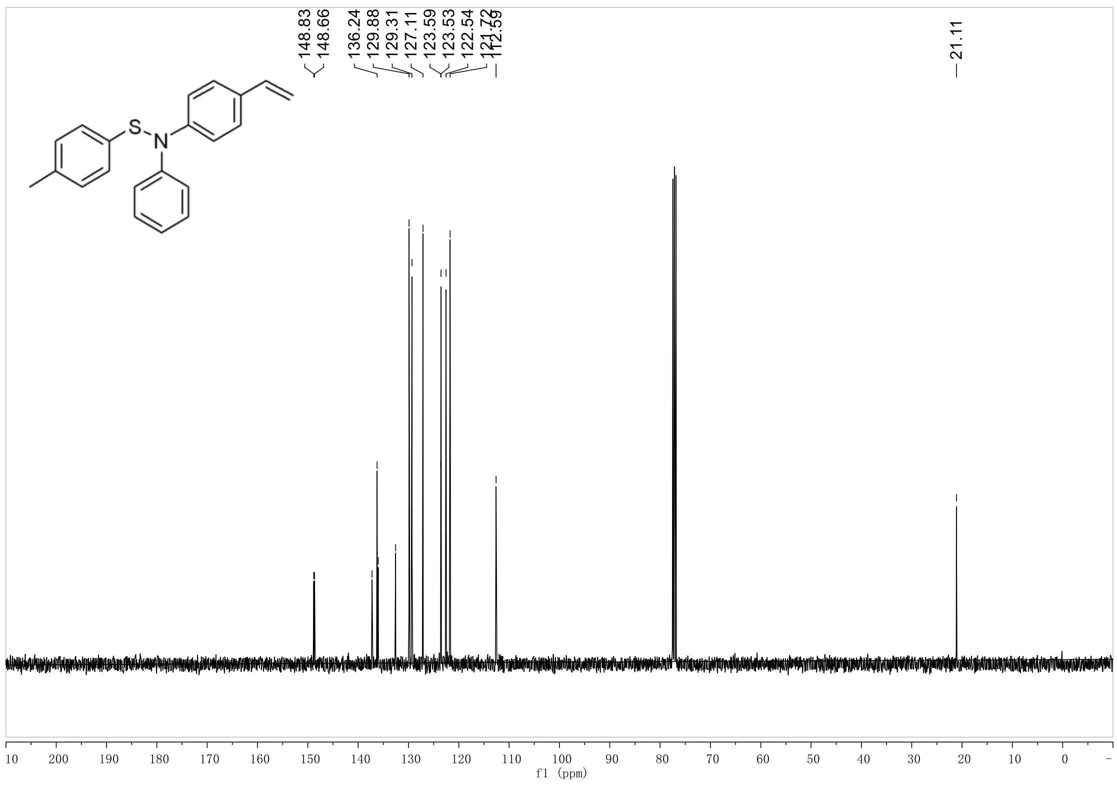

^{1}H NMR (400 MHz, $CDCl_3$) of *N*-Phenyl-*N*-(pyrimidin-5-yl)-S-(*p*-tolyl)thiohydroxylamine (3bn)

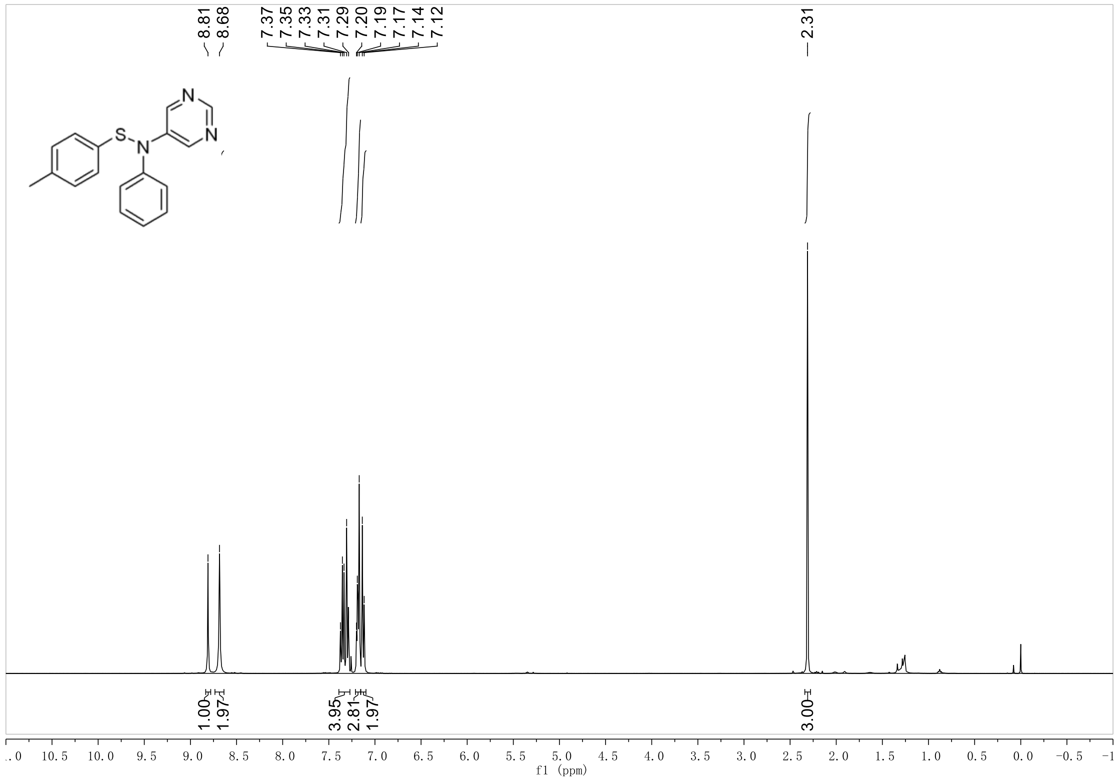

^{13}C NMR (100 MHz, $CDCl_3$) of *N*-Phenyl-*N*-(pyrimidin-5-yl)-S-(*p*-tolyl)thiohydroxylamine (3bn)

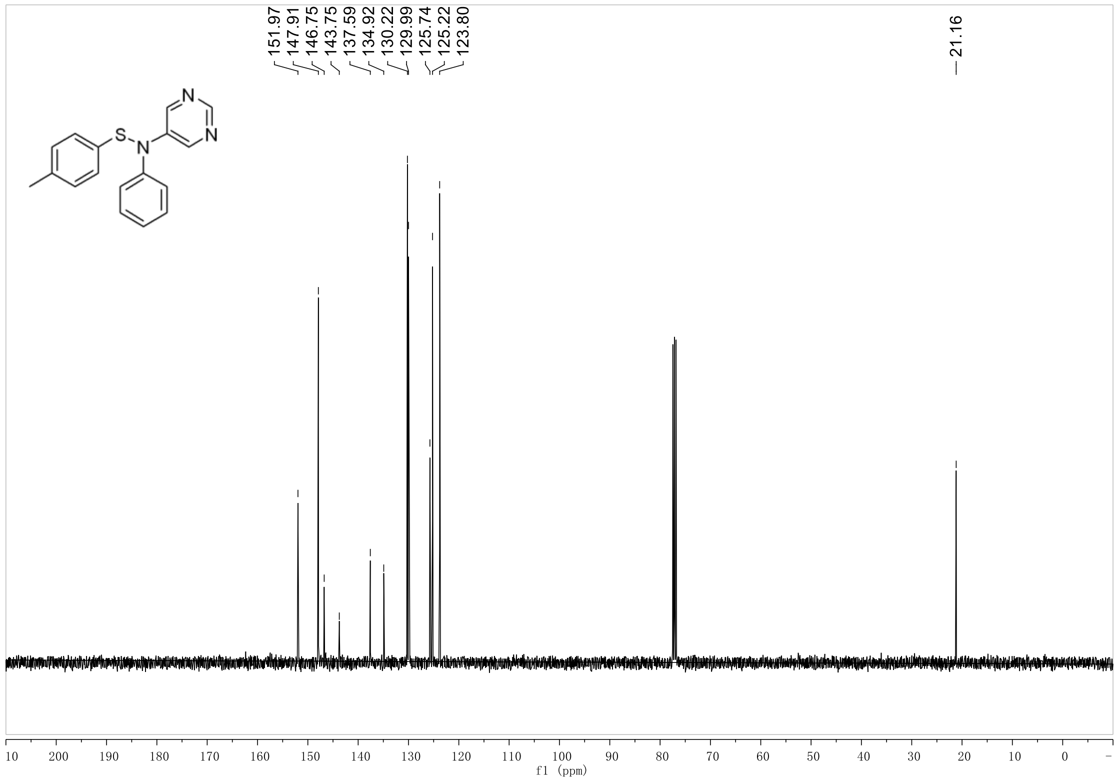

^{1}H NMR (400 MHz, CDCl$_3$) of *N*-(Benzofuran-5-yl)-*N*-phenyl-*S*-(*p*-tolyl)thiohydroxylamine (3bo)

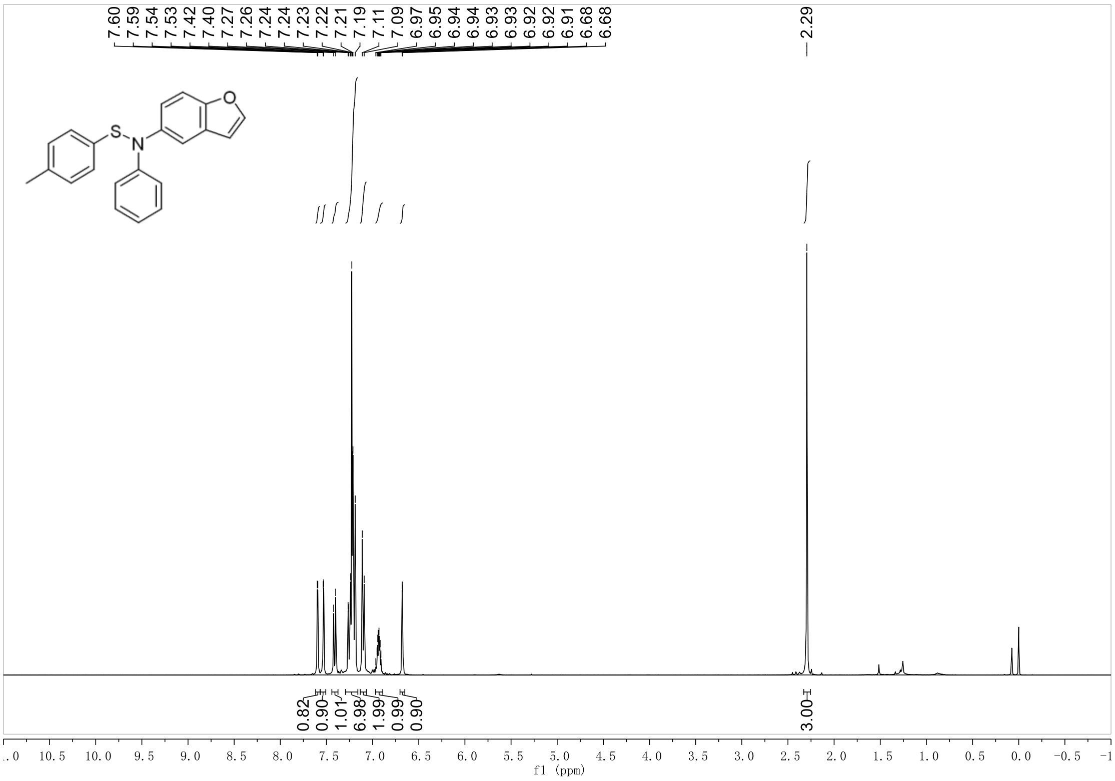

^{13}C NMR (100 MHz, CDCl$_3$) of *N*-(Benzofuran-5-yl)-*N*-phenyl-*S*-(*p*-tolyl)thiohydroxylamine (3bo)

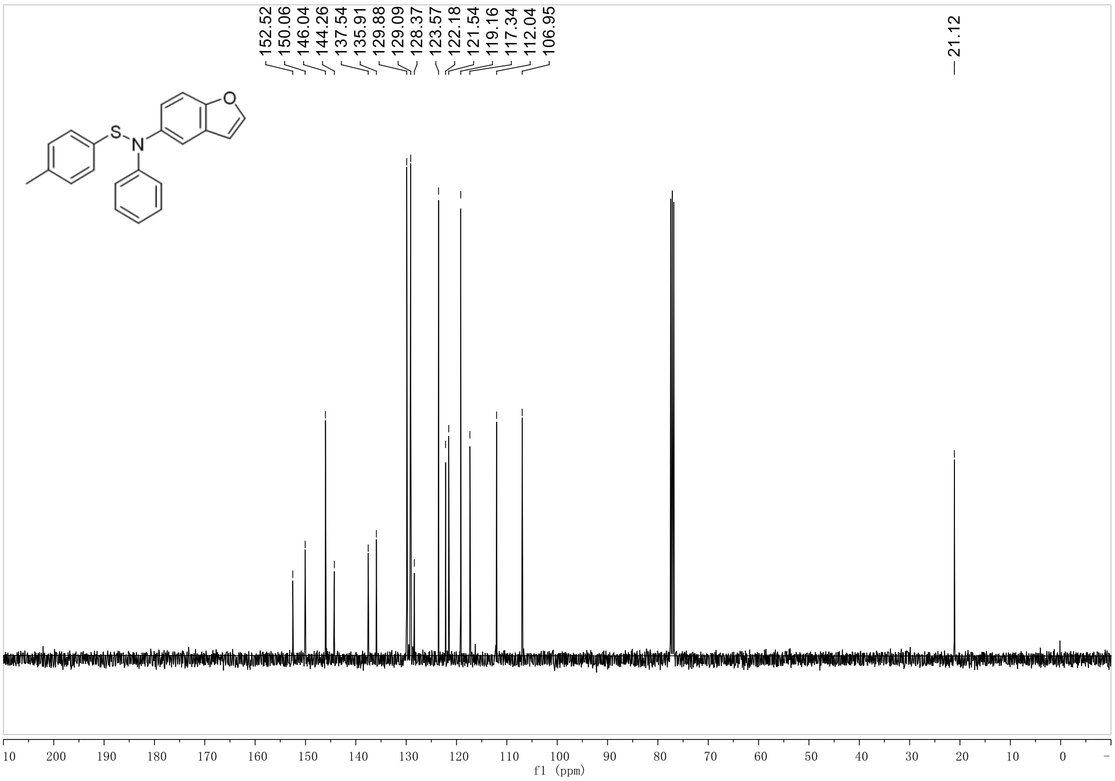

^{1}H NMR (400 MHz, $CDCl_3$) of *N*-(Benzo[b]thiophen-5-yl)-*N*-phenyl-*S*-(*p*-tolyl)thiohydroxylamine (3bp)

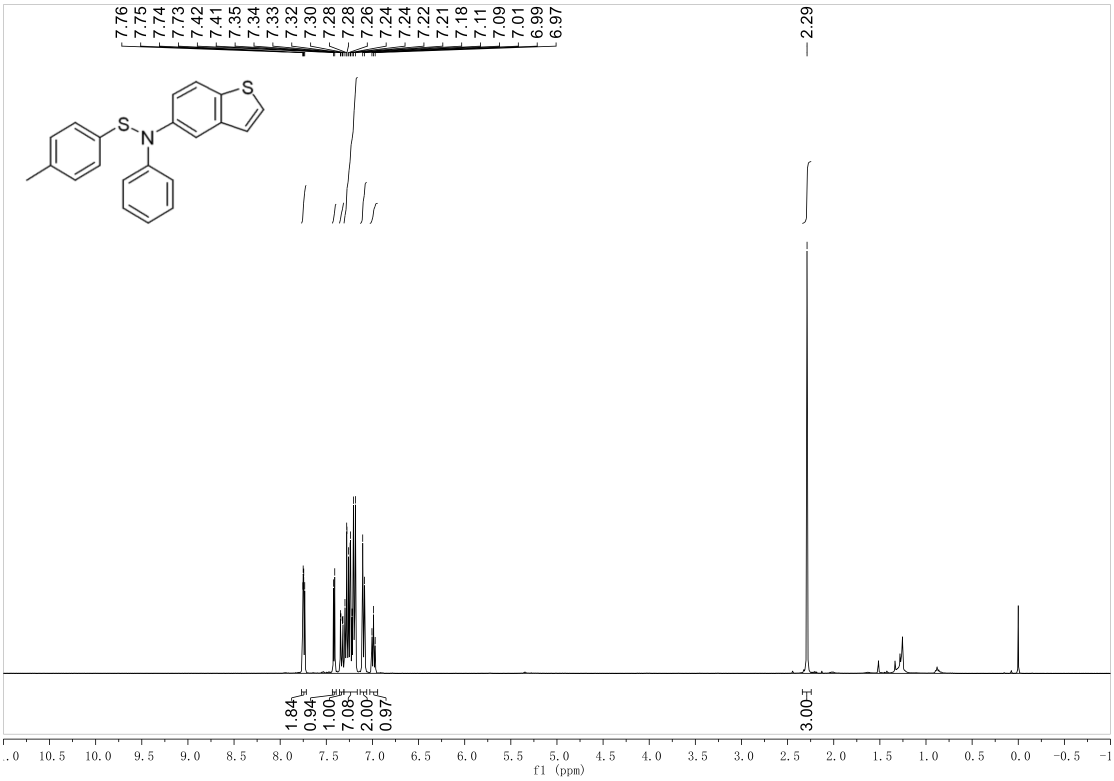

^{13}C NMR (100 MHz, $CDCl_3$) of *N*-(Benzo[b]thiophen-5-yl)-*N*-phenyl-*S*-(*p*-tolyl)thiohydroxylamine (3bp)

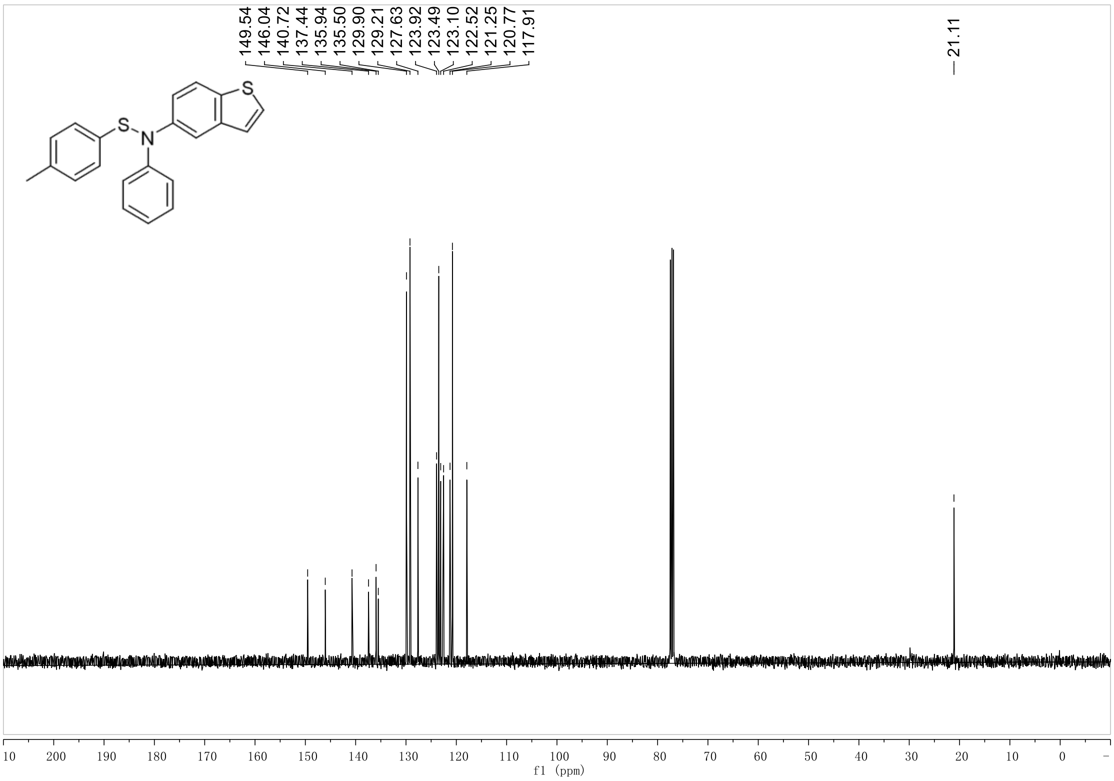

^{1}H NMR (400 MHz, $CDCl_3$) of *tert*-Butyl-(phenyl(*p*-tolylthio)amino)-1*H*-indole-1-carboxylate (3bq)

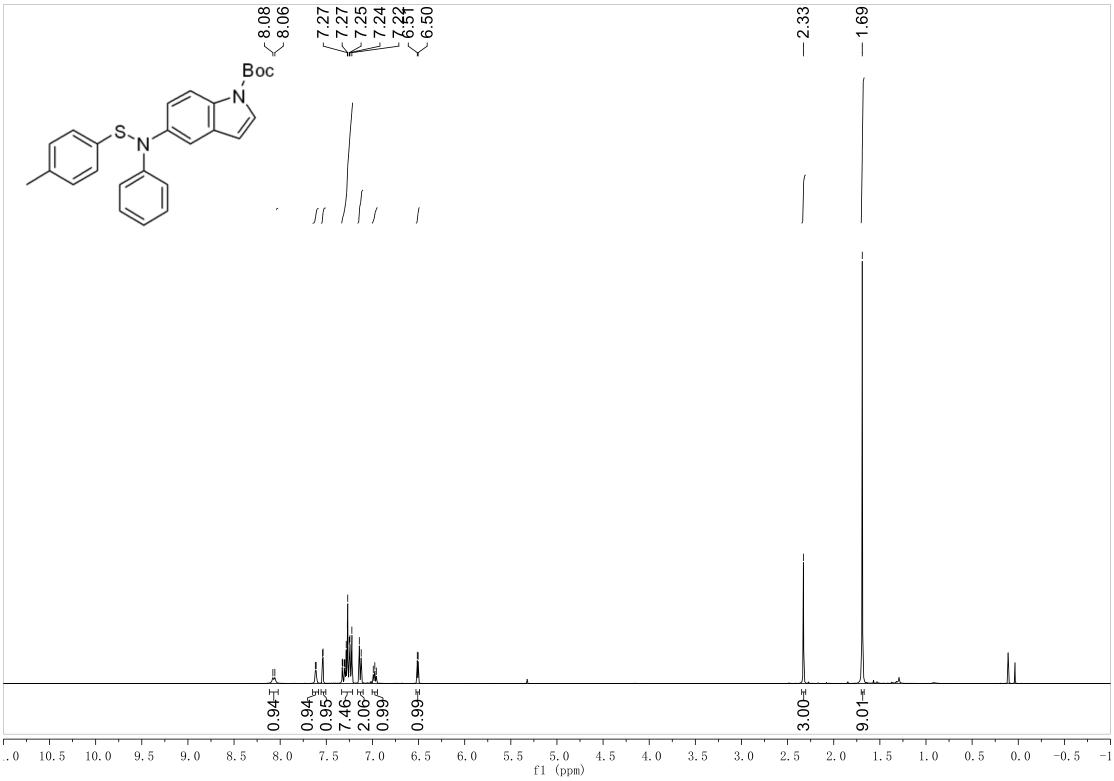

^{13}C NMR (100 MHz, $CDCl_3$) of *tert*-Butyl-(phenyl(*p*-tolylthio)amino)-1*H*-indole-1-carboxylate (3bq)

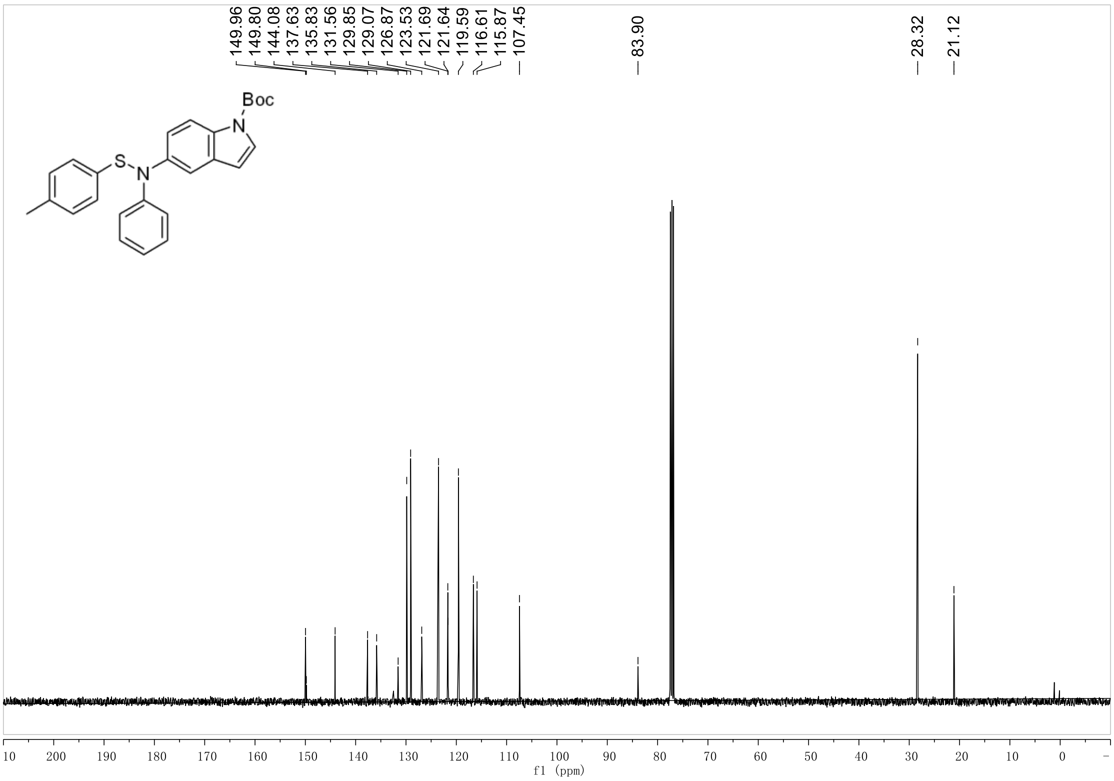

^{1}H NMR (400 MHz, $CDCl_3$) of *N*-Phenyl-*N*-(quinolin-3-yl)-*S*-(*p*-tolyl)thiohydroxylamine (3br)

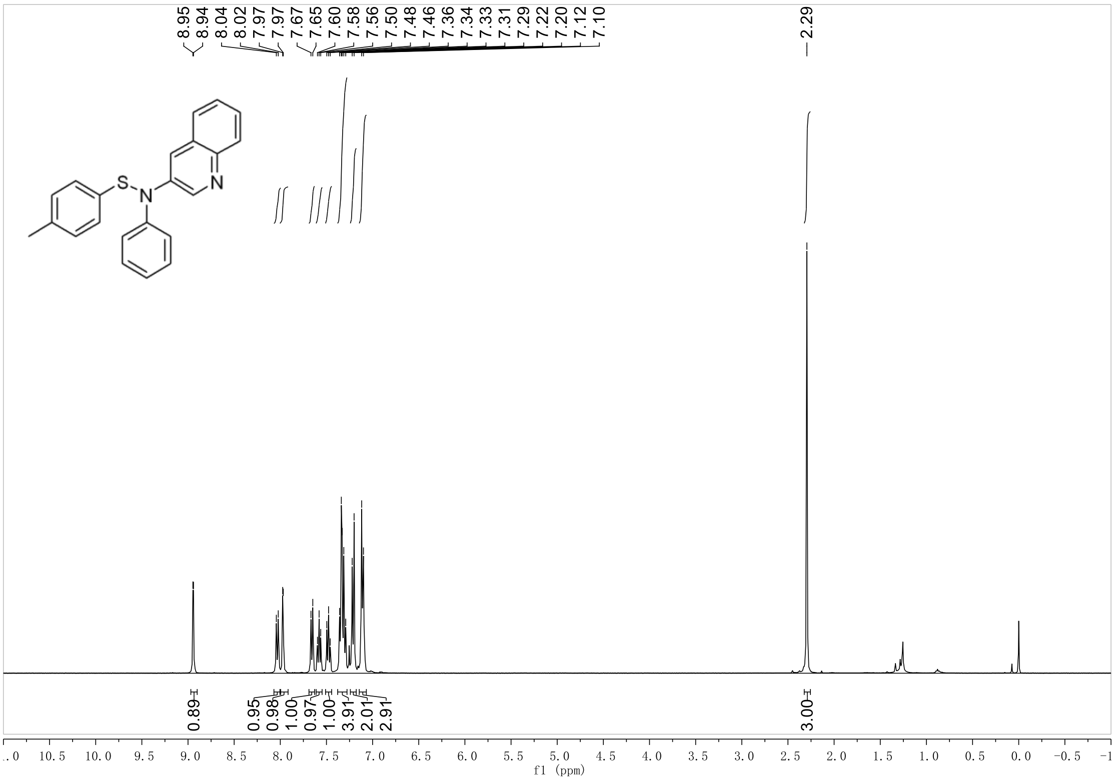

^{13}C NMR (100 MHz, $CDCl_3$) of *N*-Phenyl-*N*-(quinolin-3-yl)-*S*-(*p*-tolyl)thiohydroxylamine (3br)

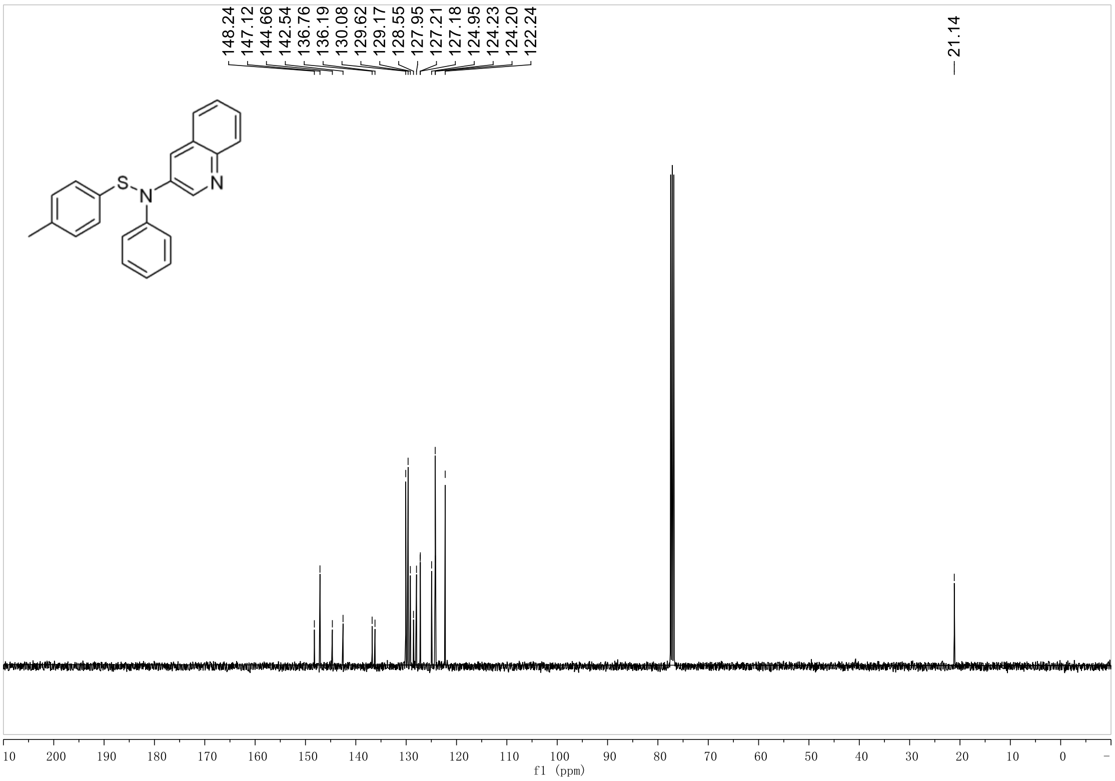

^{1}H NMR (400 MHz, $CDCl_3$) of *N*-Phenyl-*N*-(quinolin-6-yl)-*S*-(*p*-tolyl)thiohydroxylamine (3bs)

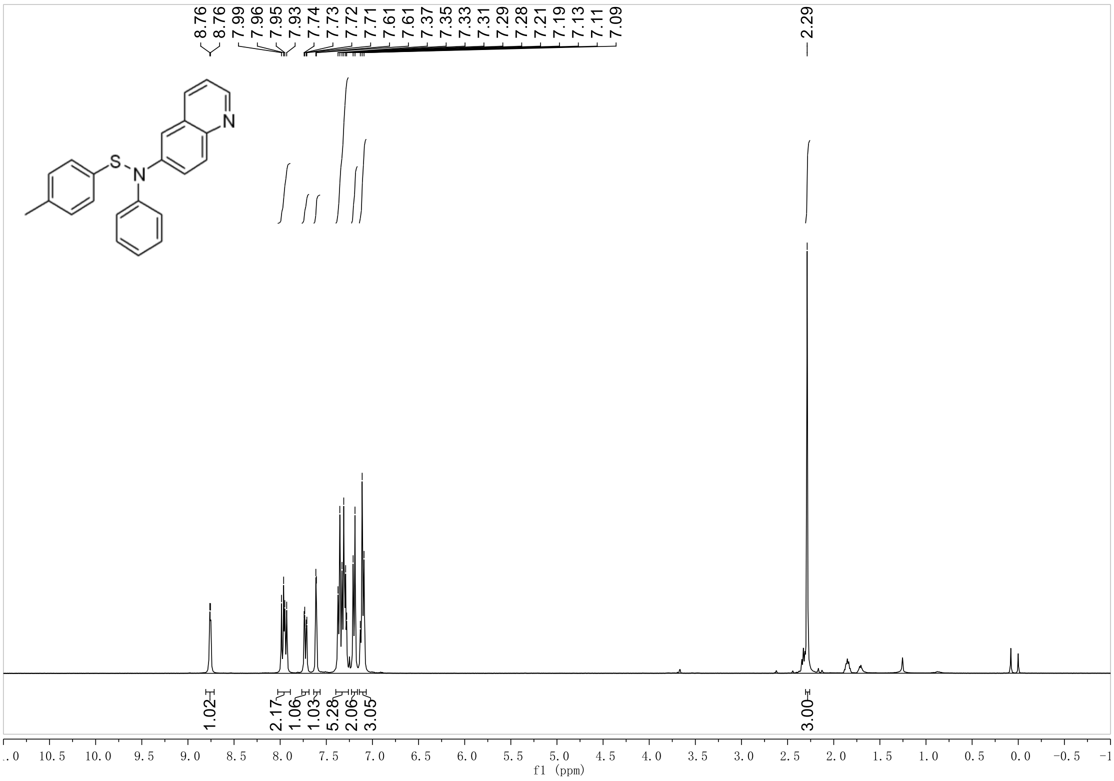

^{13}C NMR (100 MHz, $CDCl_3$) of *N*-Phenyl-*N*-(quinolin-6-yl)-*S*-(*p*-tolyl)thiohydroxylamine (3bs)

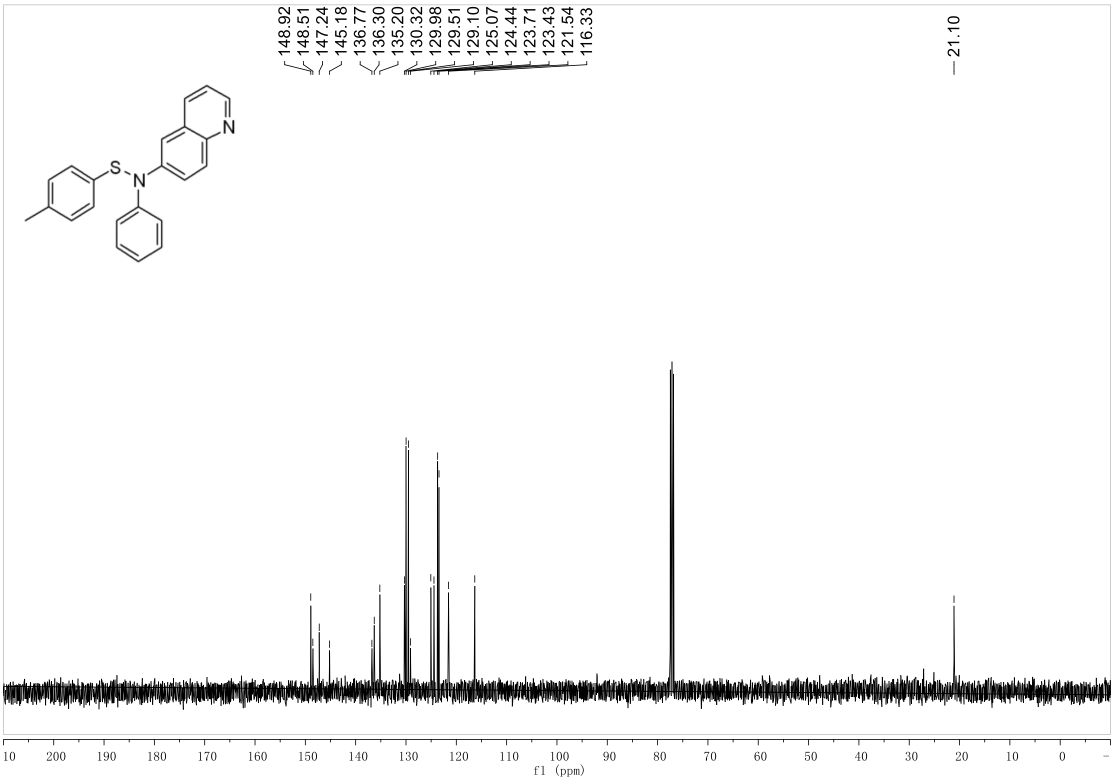

^{1}H NMR (400 MHz, $CDCl_3$) of *S*-(4-Methoxyphenyl)-*N*-phenyl-*N*-(*p*-tolyl)thiohydroxylamine (3ca)

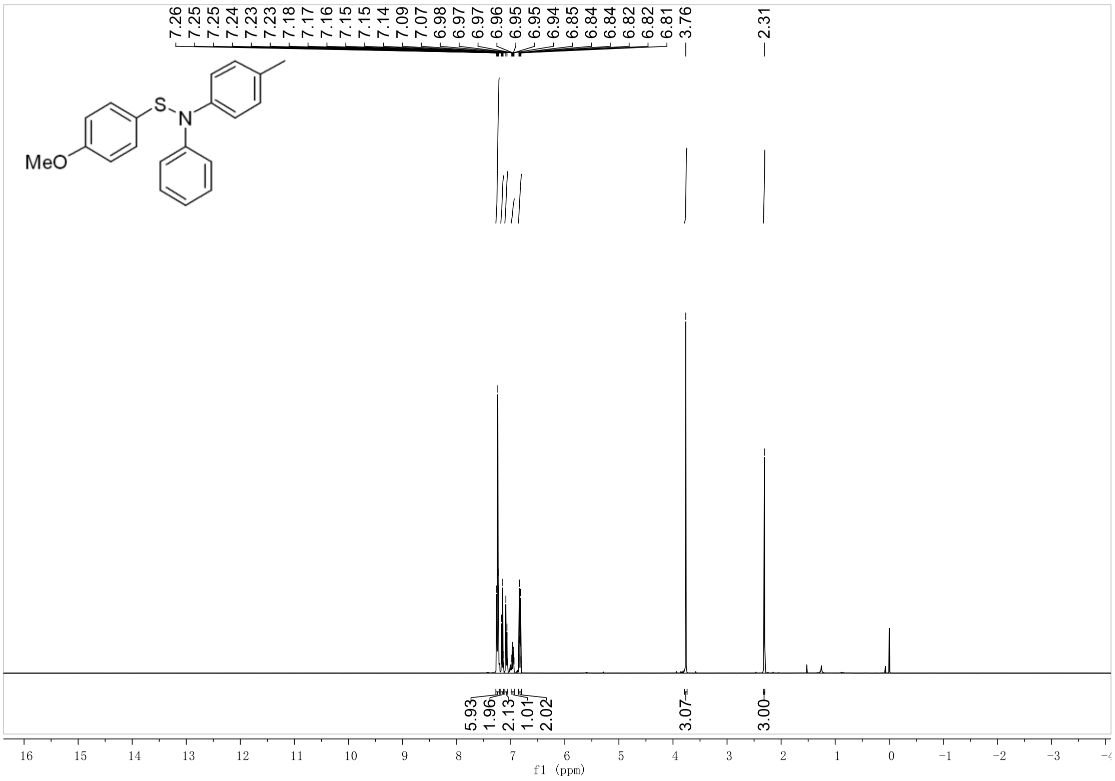

^{13}C NMR (100 MHz, $CDCl_3$) of *S*-(4-Methoxyphenyl)-*N*-phenyl-*N*-(*p*-tolyl)thiohydroxylamine (3ca)

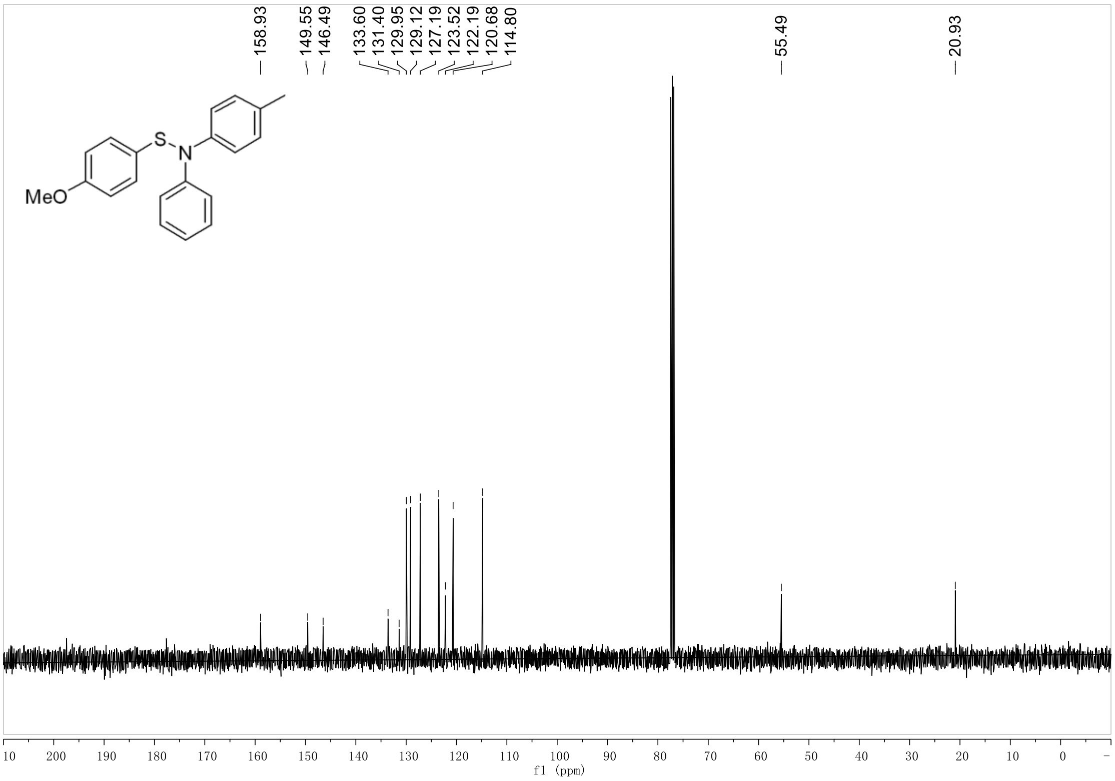

^{1}H NMR (400 MHz, $CDCl_3$) of *S*-(4-Chlorophenyl)-*N*-phenyl-*N*-(*p*-tolyl)thiohydroxylamine (3da)

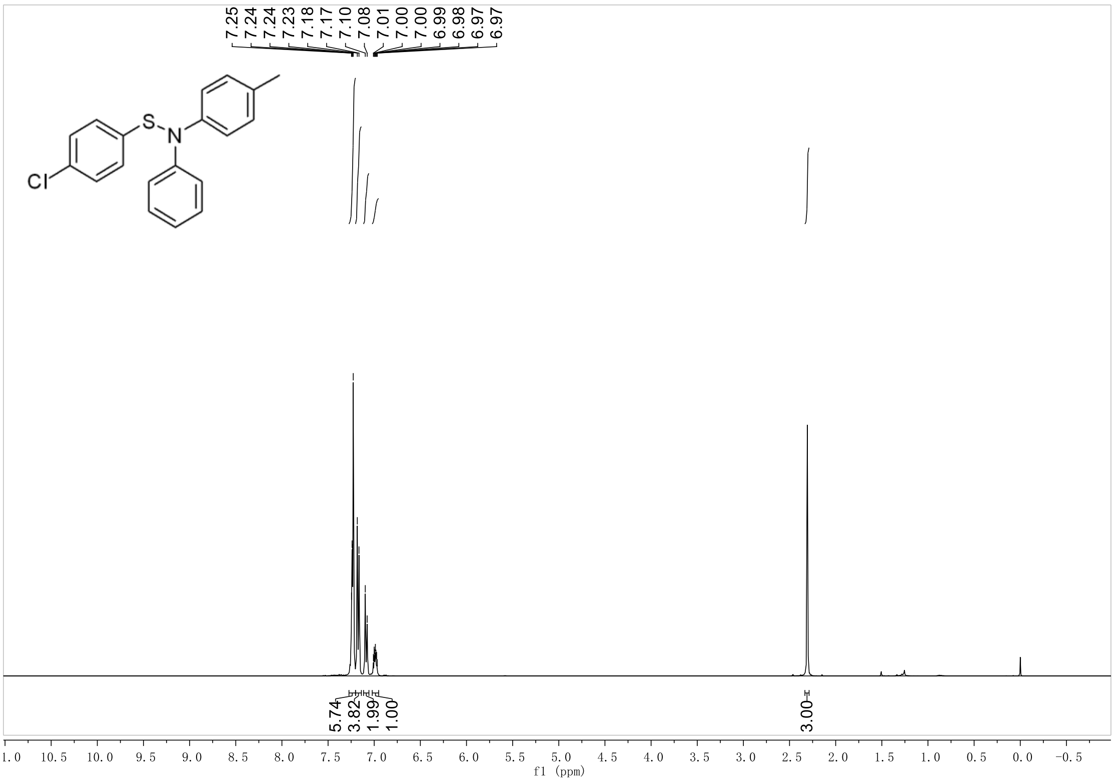

^{13}C NMR (100 MHz, $CDCl_3$) of *S*-(4-Chlorophenyl)-*N*-phenyl-*N*-(*p*-tolyl)thiohydroxylamine (3da)

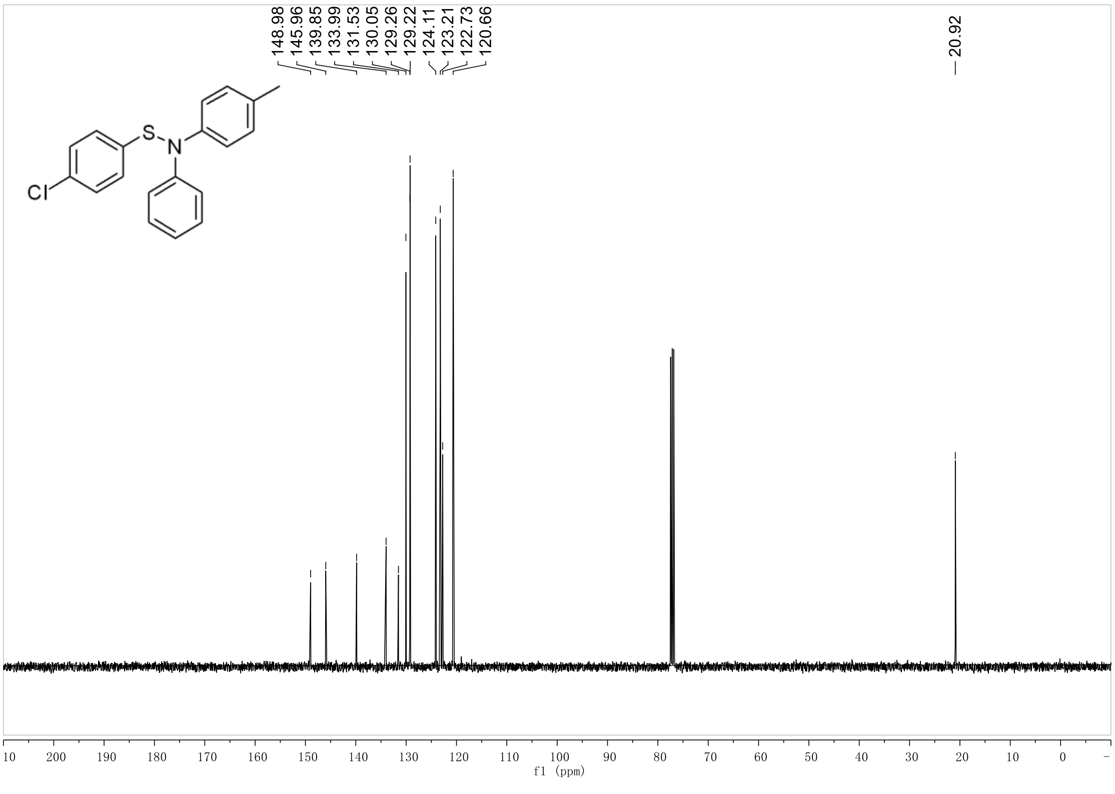

^{1}H NMR (400 MHz, $CDCl_3$) of *S*-(4-Nitrophenyl)-*N*-phenyl-*N*-(*p*-tolyl)thiohydroxylamine (3ea)

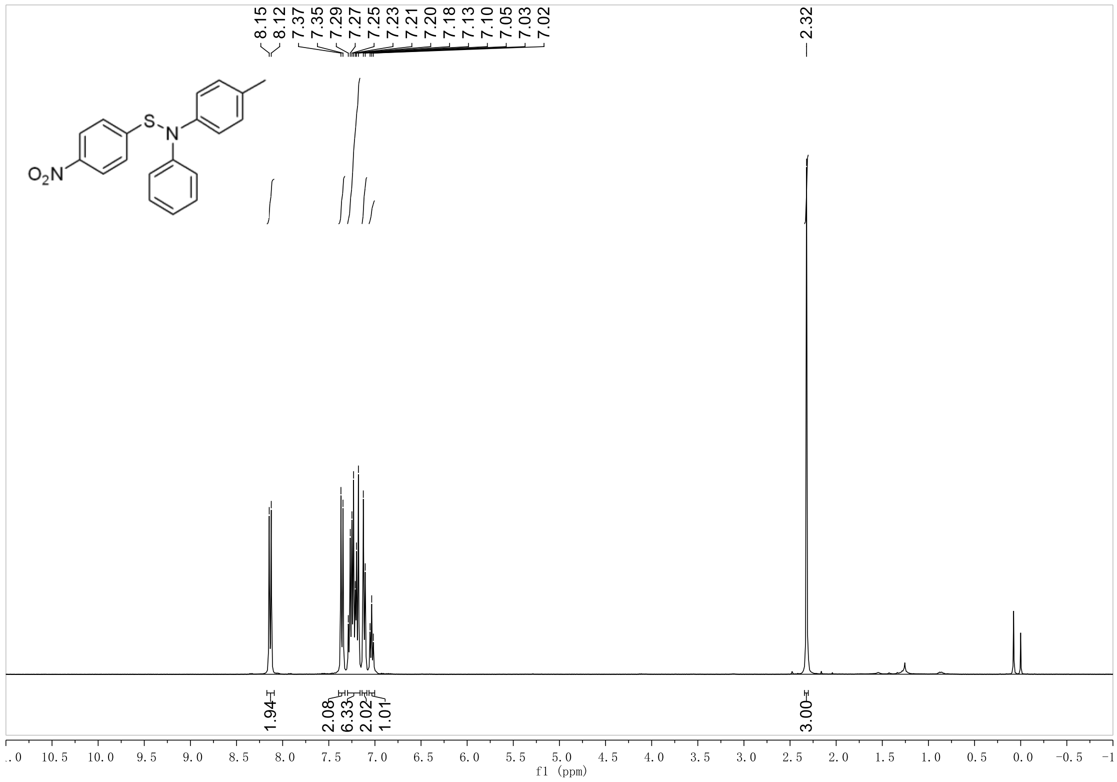

^{13}C NMR (100 MHz, $CDCl_3$) of *S*-(4-Nitrophenyl)-*N*-phenyl-*N*-(*p*-tolyl)thiohydroxylamine (3ea)

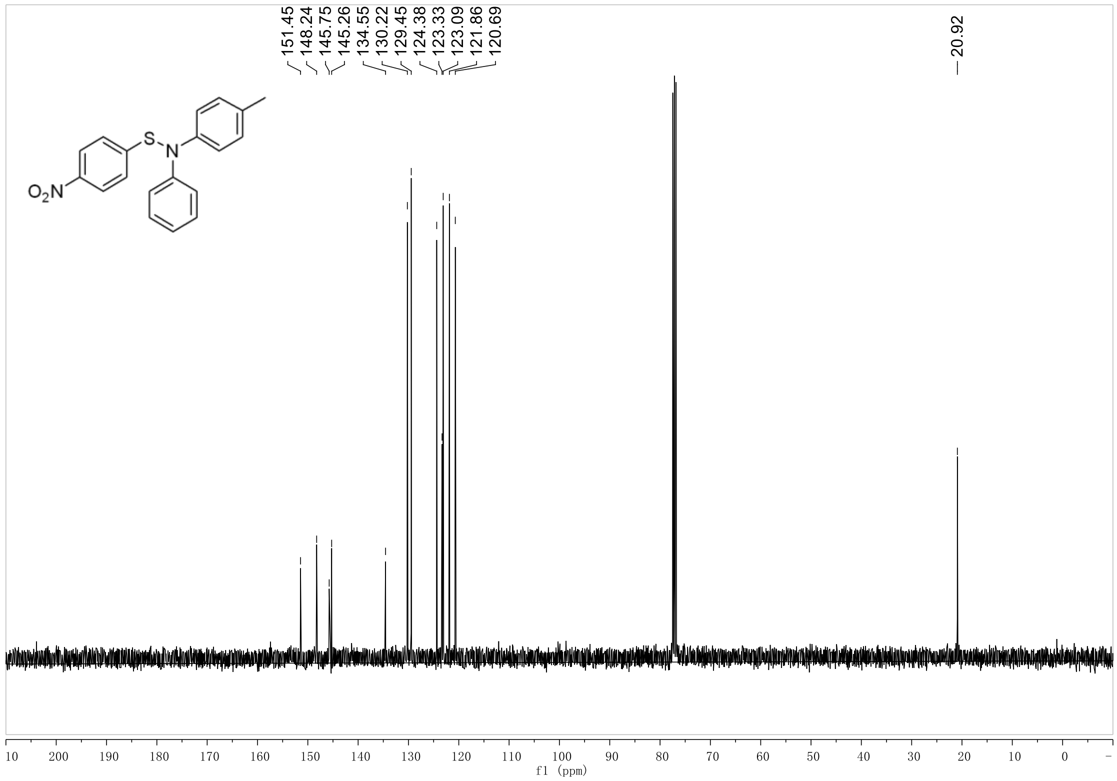

^{1}H NMR (400 MHz, $CDCl_3$) of *N*-Phenyl-*S*-(*o*-tolyl)-N-(*p*-tolyl)thiohydroxylamine (3fa)

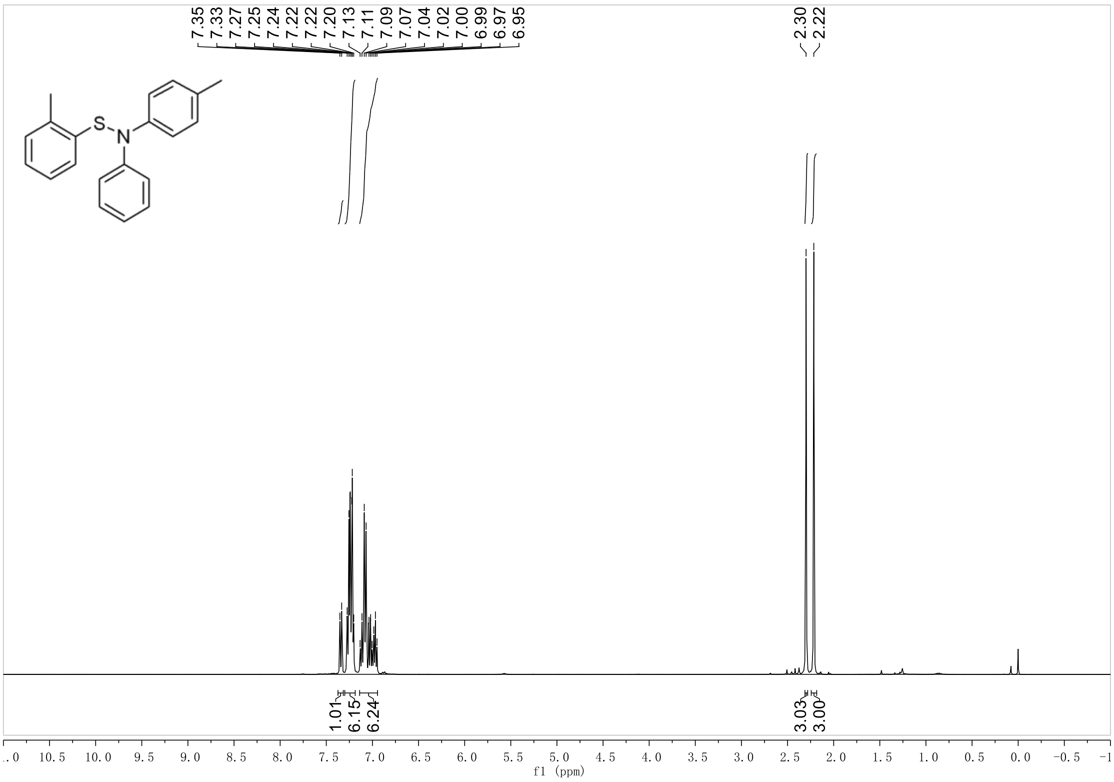

^{13}C NMR (100 MHz, $CDCl_3$) of *N*-Phenyl-*S*-(*o*-tolyl)-N-(*p*-tolyl)thiohydroxylamine (3fa)

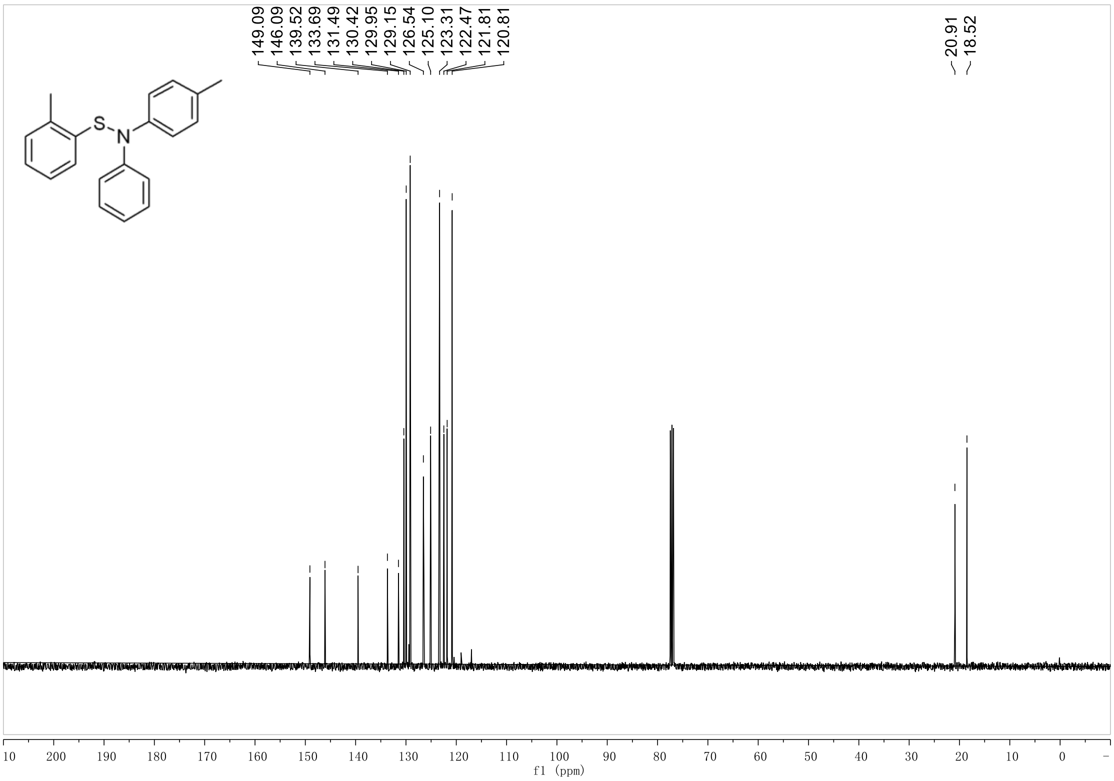

^{1}H NMR (400 MHz, $CDCl_3$) of *S*-(2-Methoxyphenyl)-*N*-phenyl-*N*-(*p*-tolyl)thiohydroxylamine (3ga)

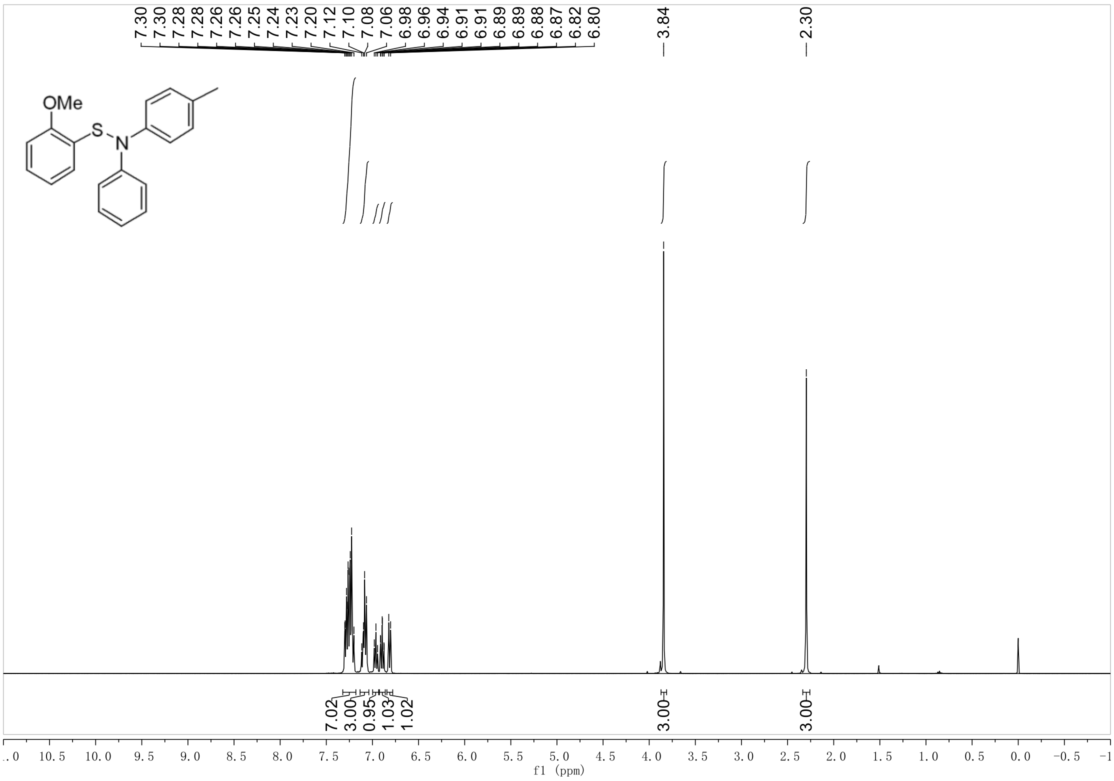

^{13}C NMR (100 MHz, $CDCl_3$) of *S*-(2-Methoxyphenyl)-*N*-phenyl-*N*-(*p*-tolyl)thiohydroxylamine (3ga)

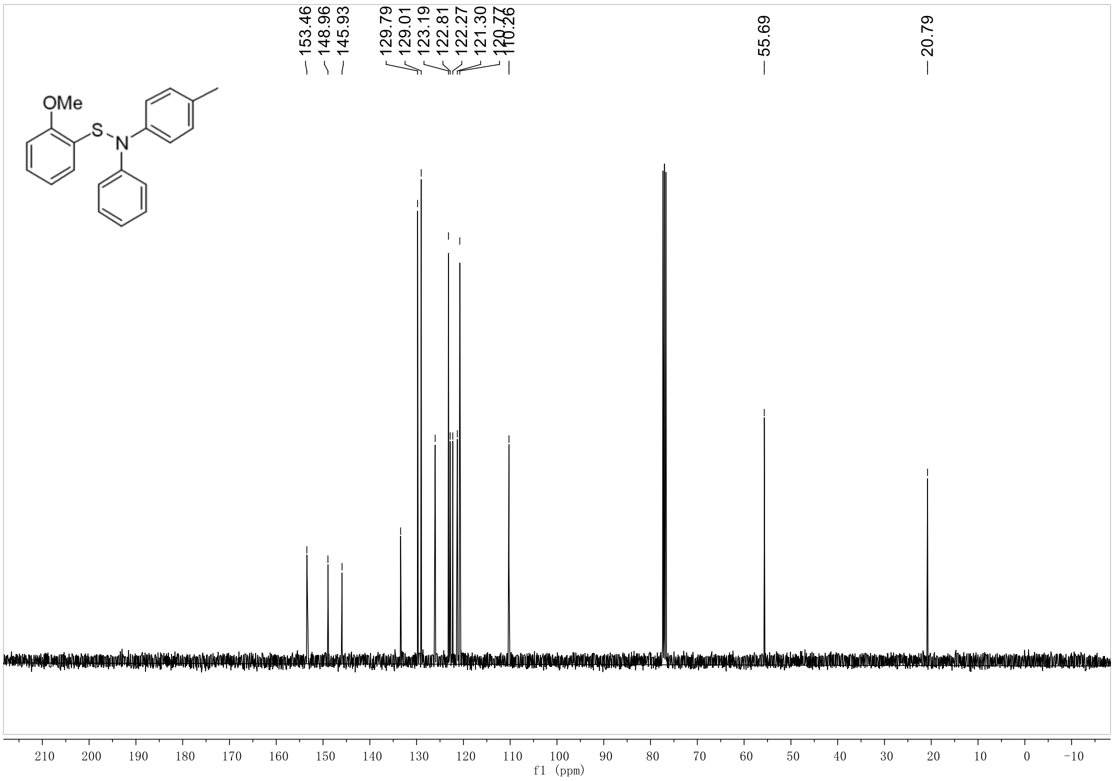

^{1}H NMR (400 MHz, $CDCl_3$) of ***S*-(2-Chlorophenyl)-*N*-phenyl-*N*-(*p*-tolyl)thiohydroxylamine (3ha)**

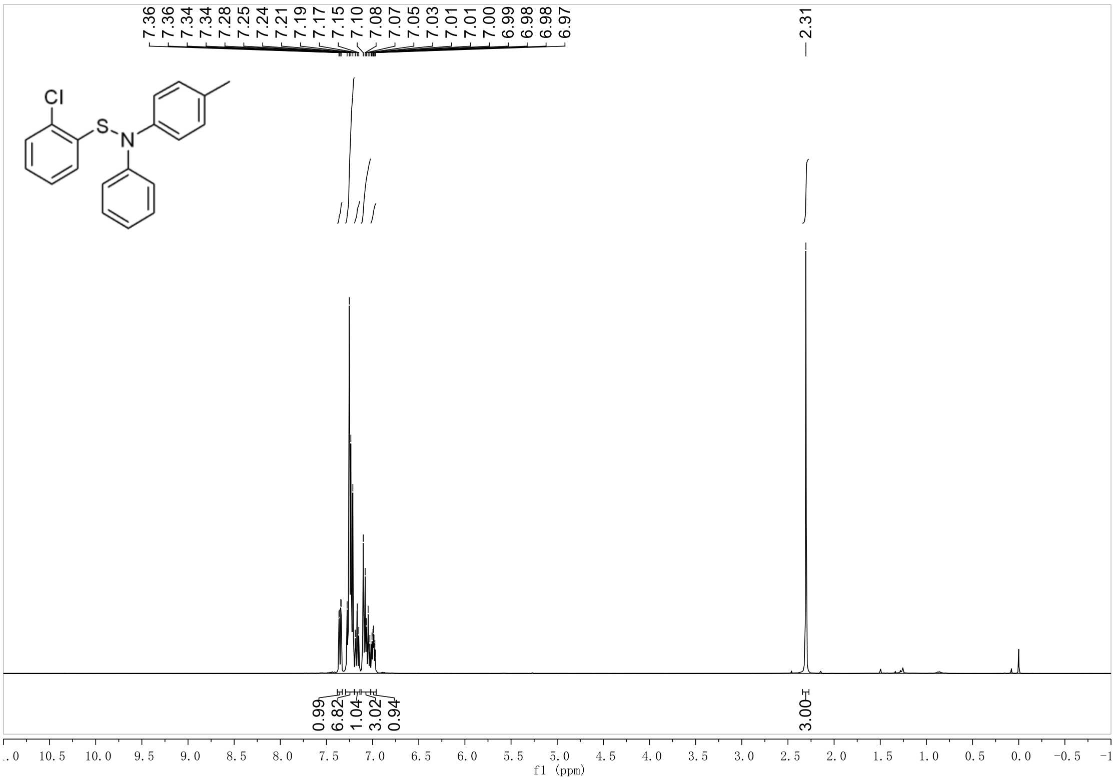

^{13}C NMR (100 MHz, $CDCl_3$) of ***S*-(2-Chlorophenyl)-*N*-phenyl-*N*-(*p*-tolyl)thiohydroxylamine (3ha)**

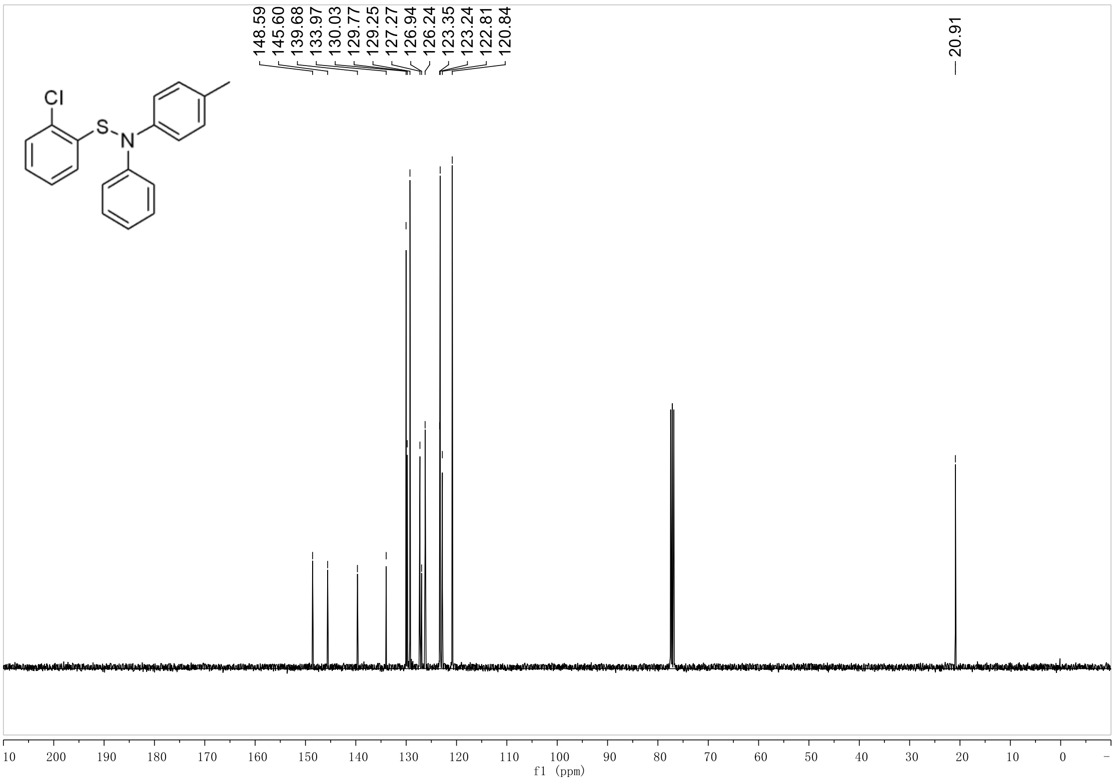

^{1}H NMR (400 MHz, $CDCl_3$) of Methyl 2-((phenyl(*p*-tolyl)amino)thio)benzoate (3ia)

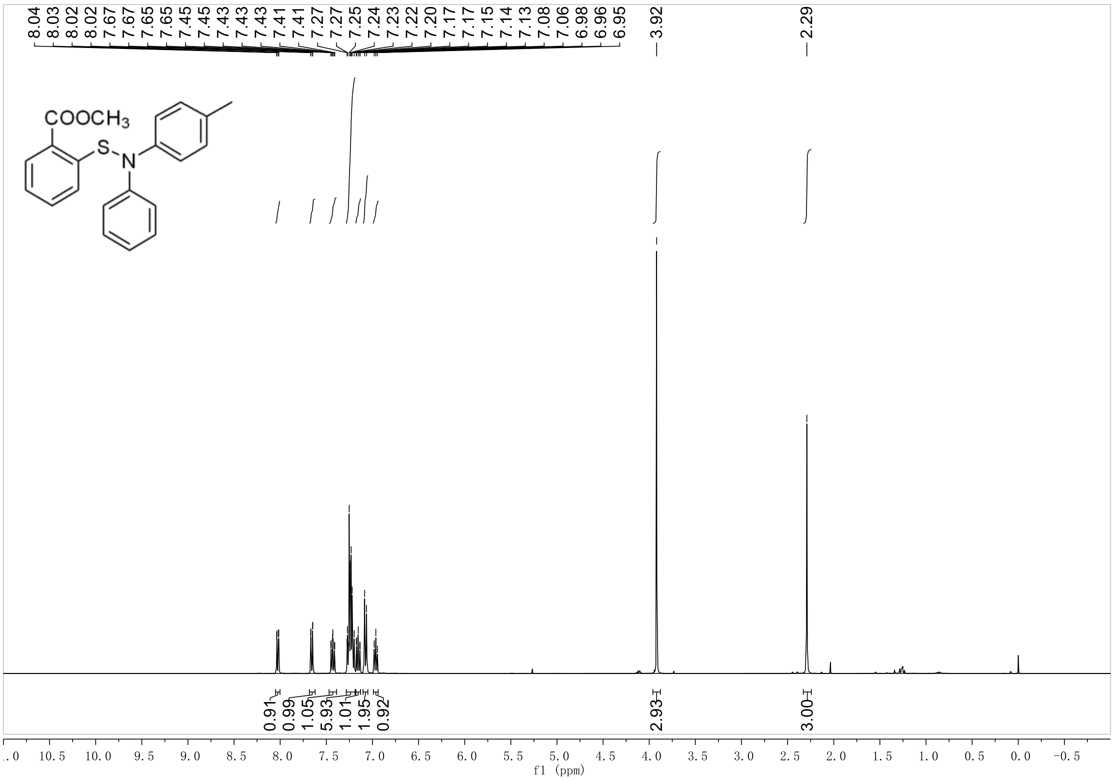

^{13}C NMR (100 MHz, $CDCl_3$) of Methyl 2-((phenyl(*p*-tolyl)amino)thio)benzoate (3ia)

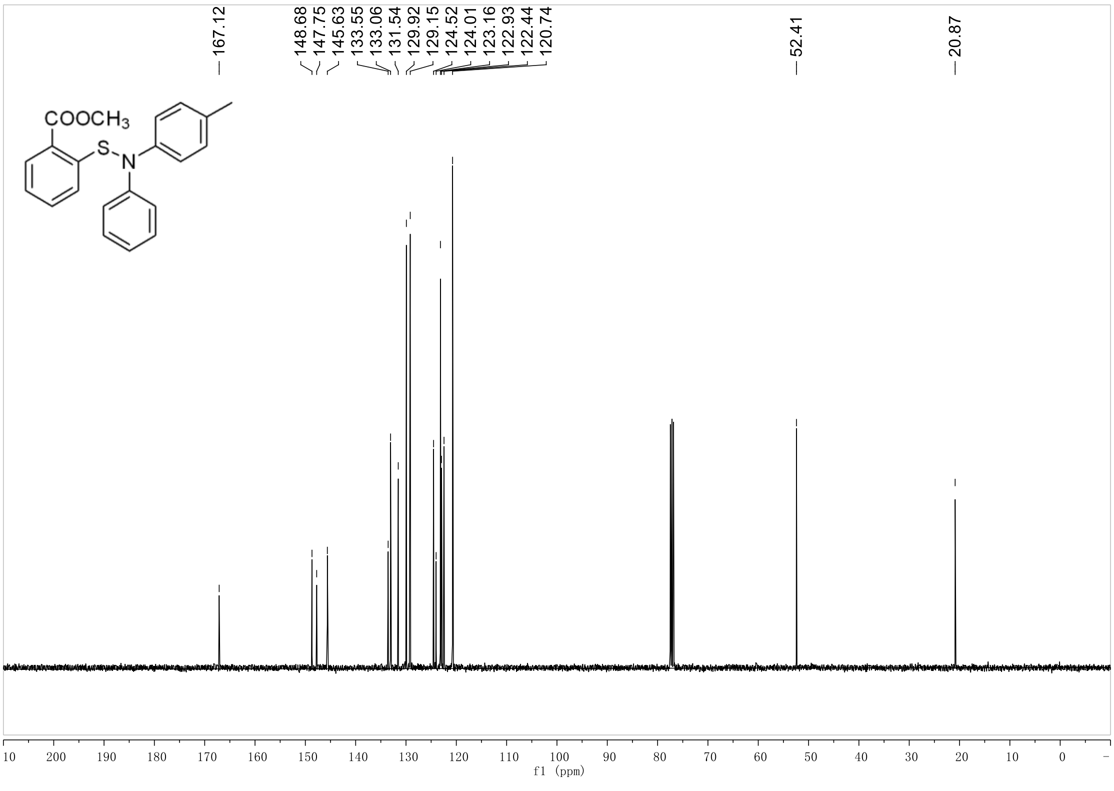

^{1}H NMR (400 MHz, $CDCl_3$) of *S*-(2,6-Dimethylphenyl)-*N*-phenyl-*N*-(*p*-tolyl)thiohydroxylamine (3ja)

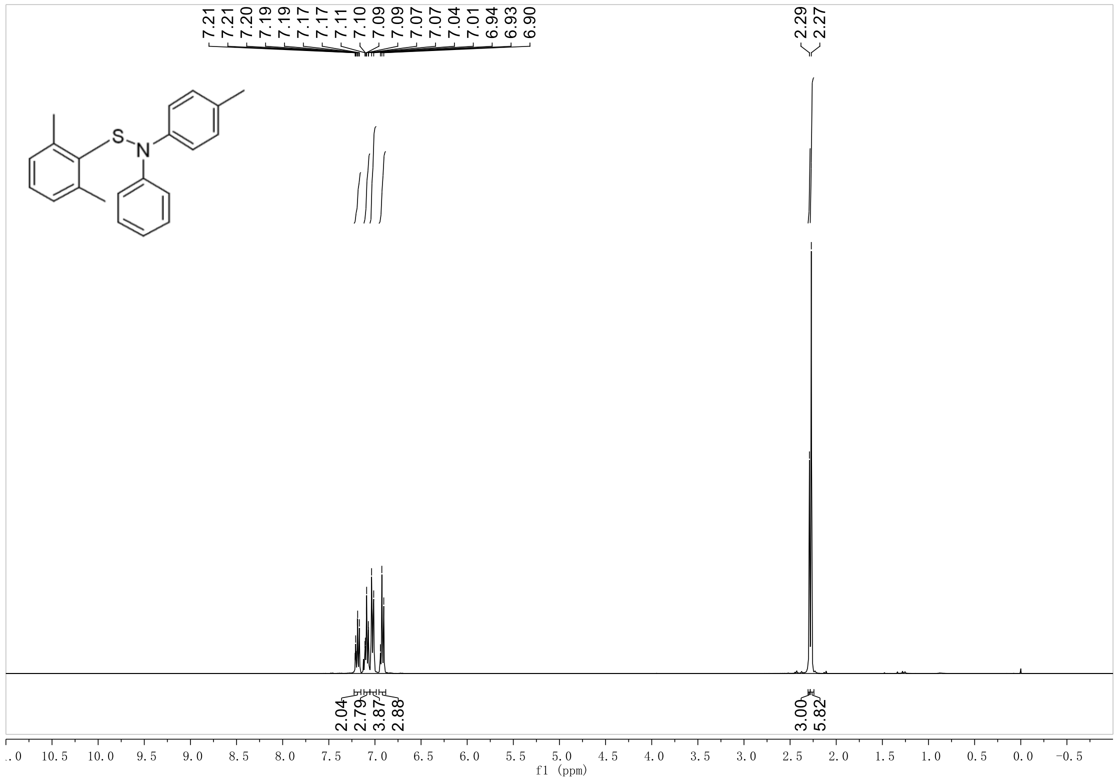

^{13}C NMR (100 MHz, $CDCl_3$) of *S*-(2,6-Dimethylphenyl)-*N*-phenyl-*N*-(*p*-tolyl)thiohydroxylamine (3ja)

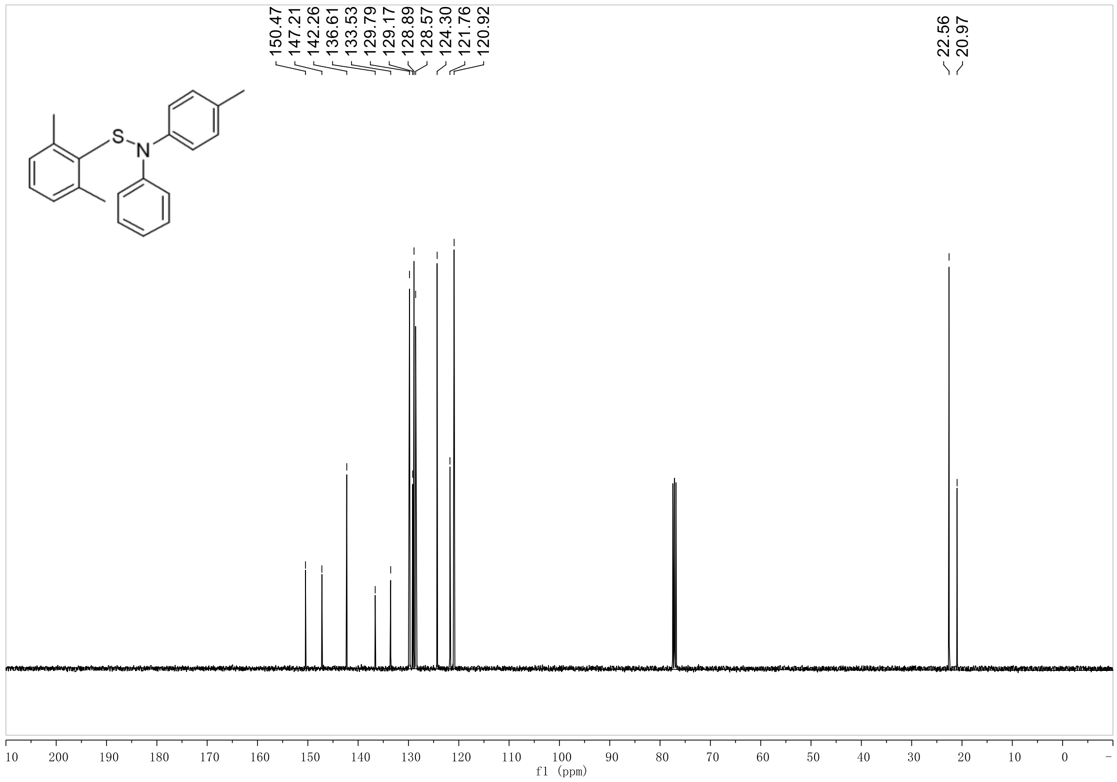

^{1}H NMR (400 MHz, $CDCl_3$) of *S*-(3-Bromophenyl)-*N*-phenyl-*N*-(*p*-tolyl)thiohydroxylamine (3ka)

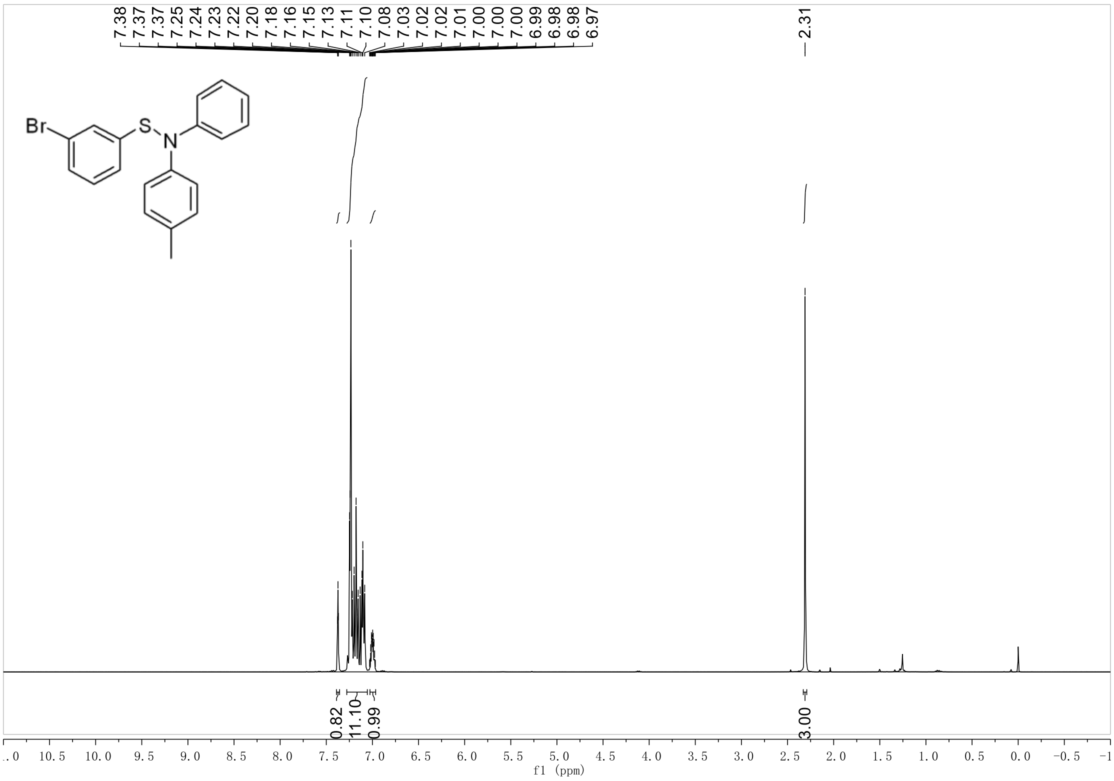

^{13}C NMR (100 MHz, $CDCl_3$) of *S*-(3-Bromophenyl)-*N*-phenyl-*N*-(*p*-tolyl)thiohydroxylamine (3ka)

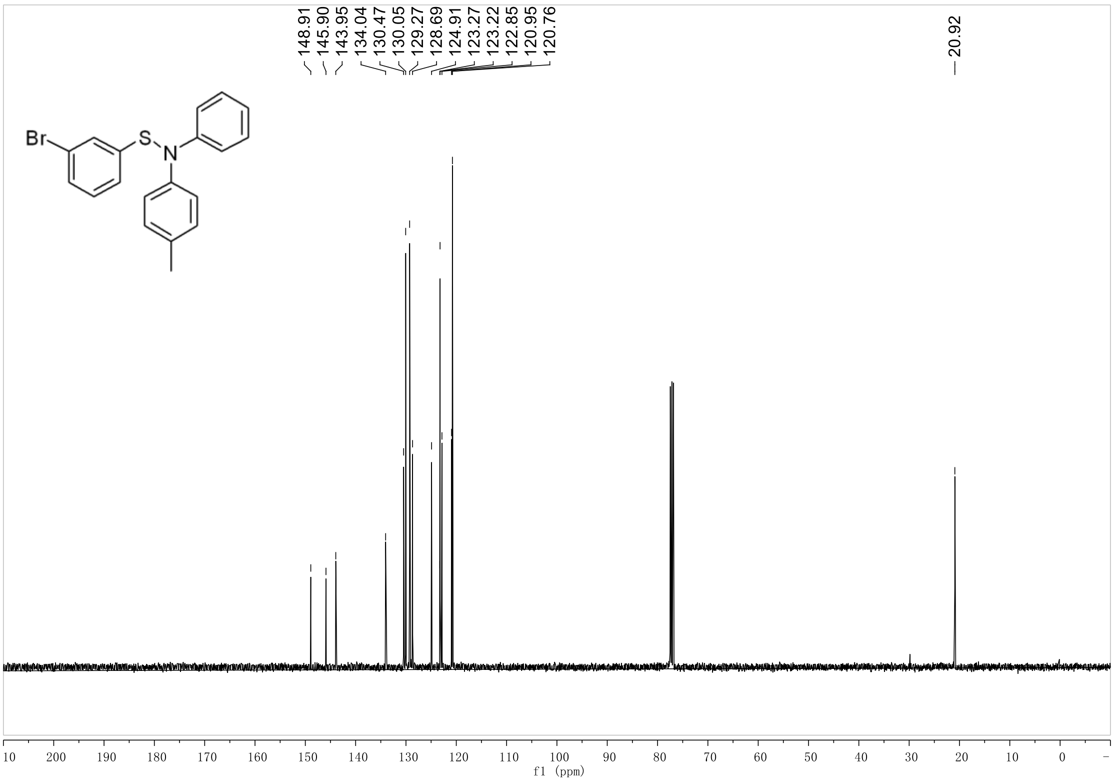

^{1}H NMR (400 MHz, $CDCl_3$) of *S*-(3-Bromophenyl)-*N*-phenyl-*N*-(*p*-tolyl)thiohydroxylamine (3la)

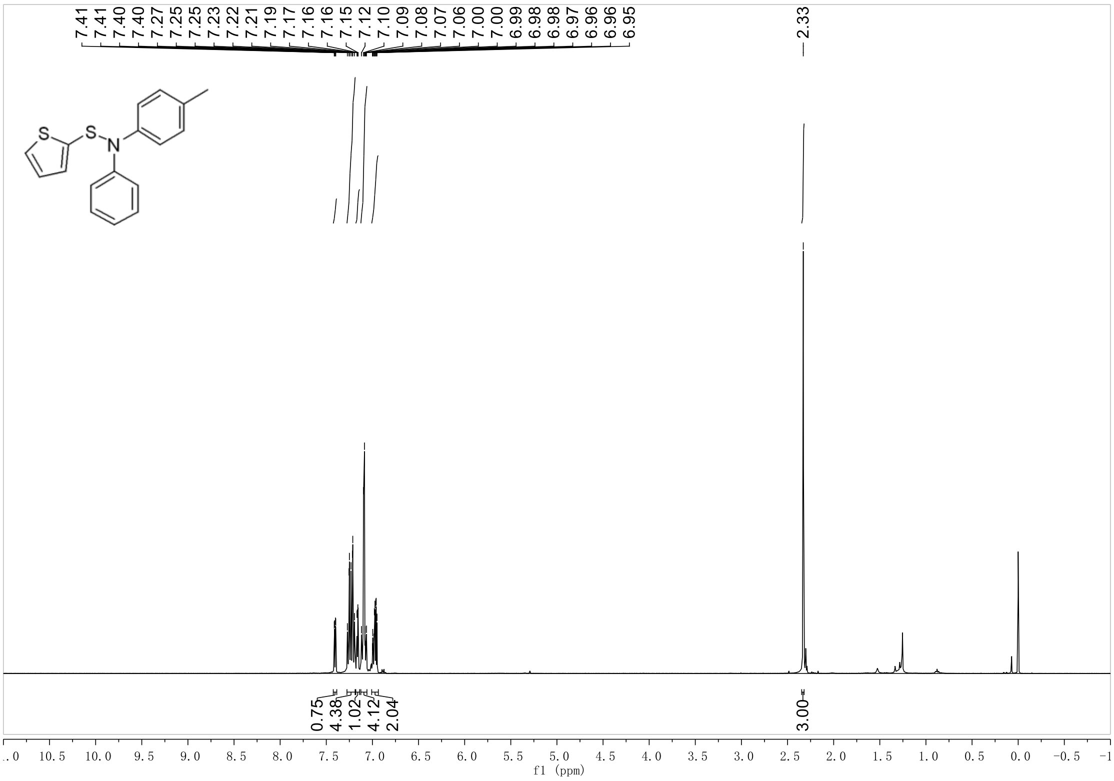

^{13}C NMR (100 MHz, $CDCl_3$) of *S*-(3-Bromophenyl)-*N*-phenyl-*N*-(*p*-tolyl)thiohydroxylamine (3la)

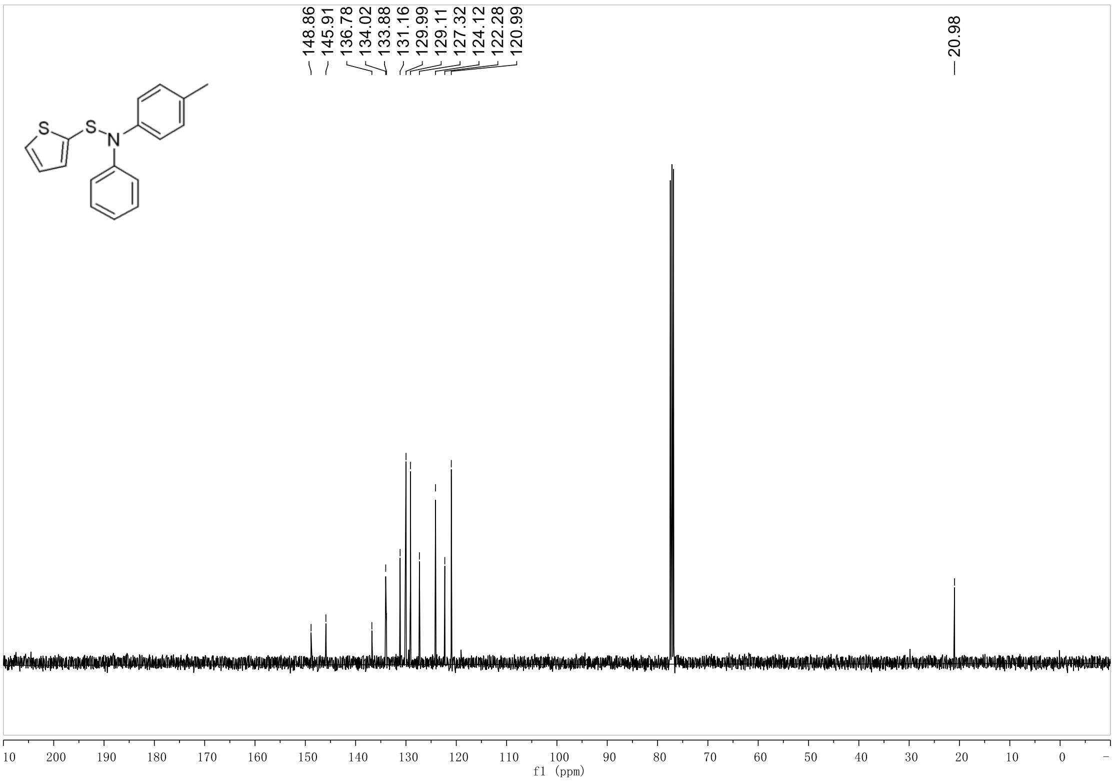

^{1}H NMR (400 MHz, $CDCl_3$) of *N,N,S*-tri-*p*-tolylthiohydroxylamine (3ma)

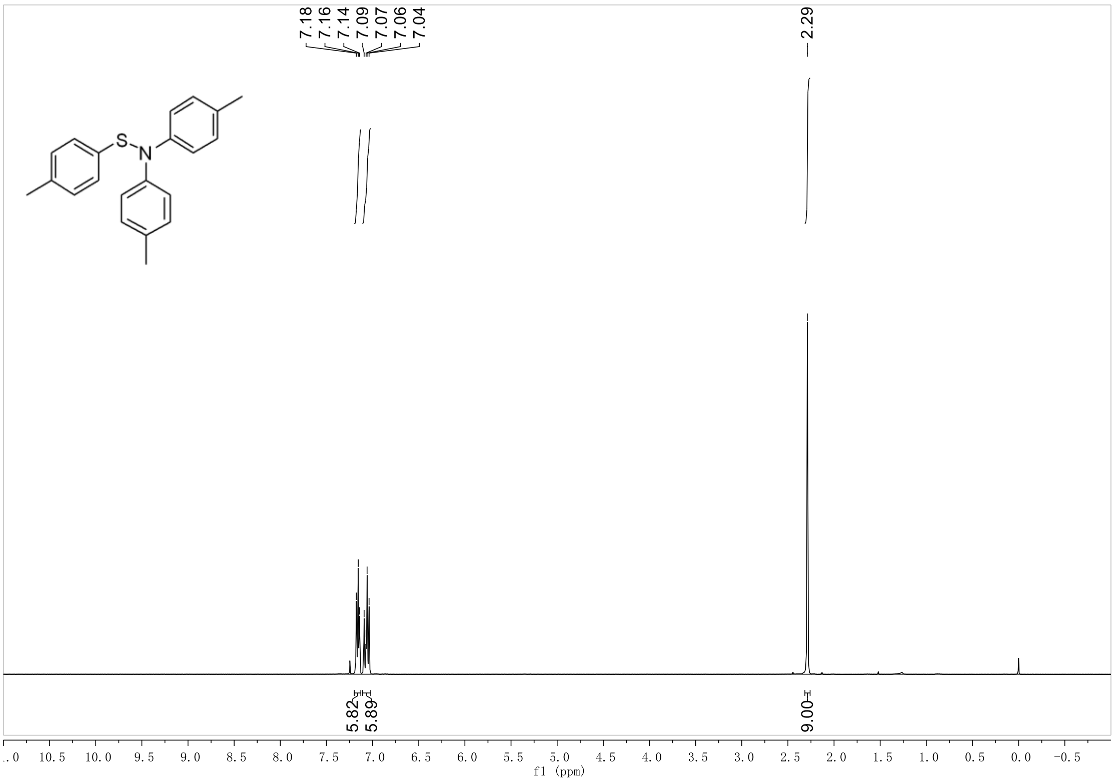

^{13}C NMR (100 MHz, $CDCl_3$) of *N,N,S*-tri-*p*-tolylthiohydroxylamine (3ma)

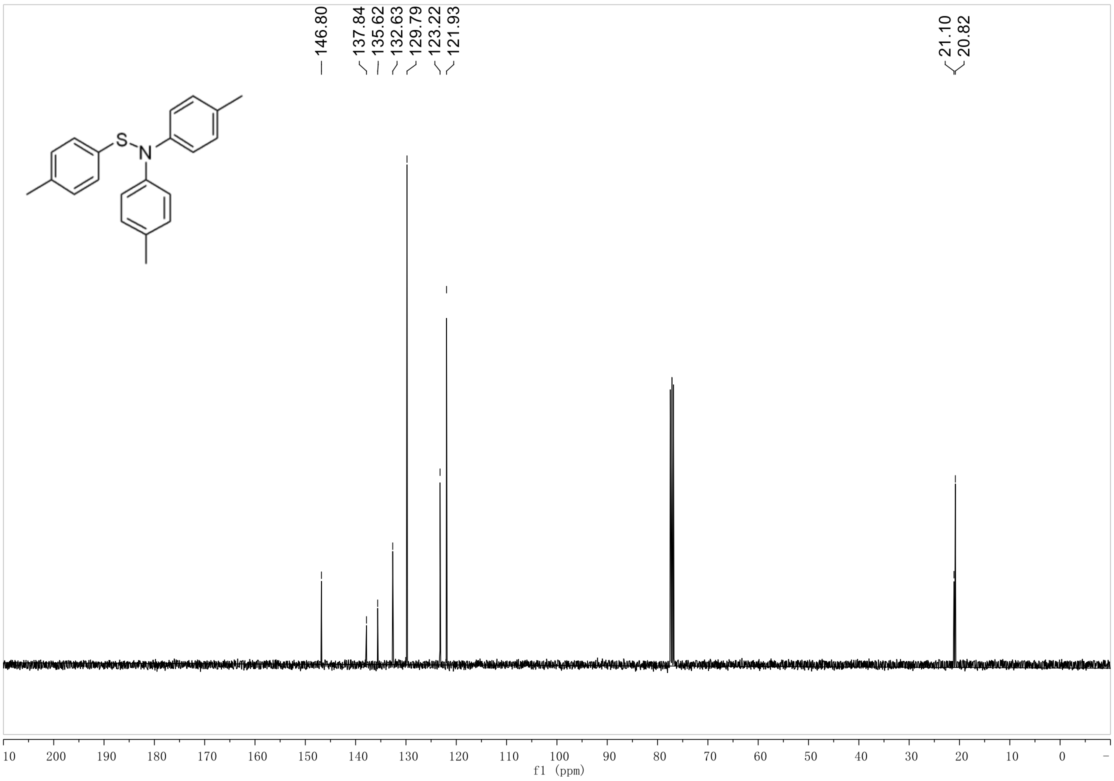

^{1}H NMR (400 MHz, $CDCl_3$) of *N,N,S*-tri-*p*-Tolylthiohydroxylamine (3na)

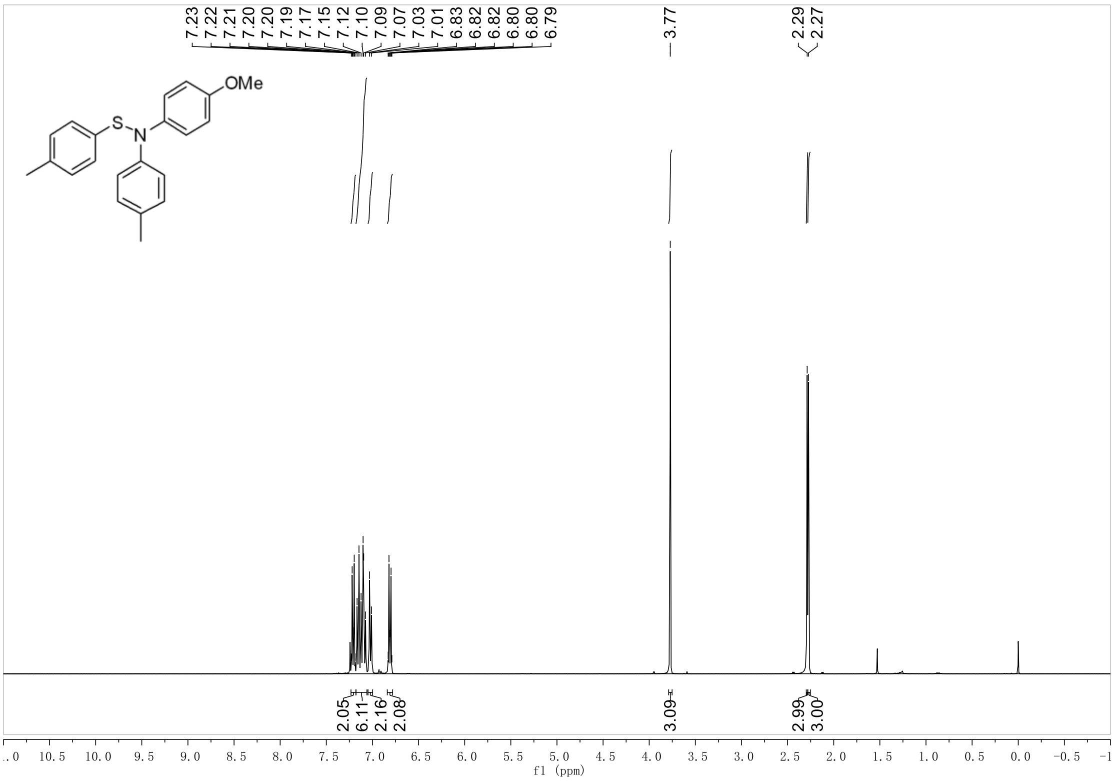

^{13}C NMR (100 MHz, $CDCl_3$) of *N,N,S*-tri-*p*-Tolylthiohydroxylamine (3na)

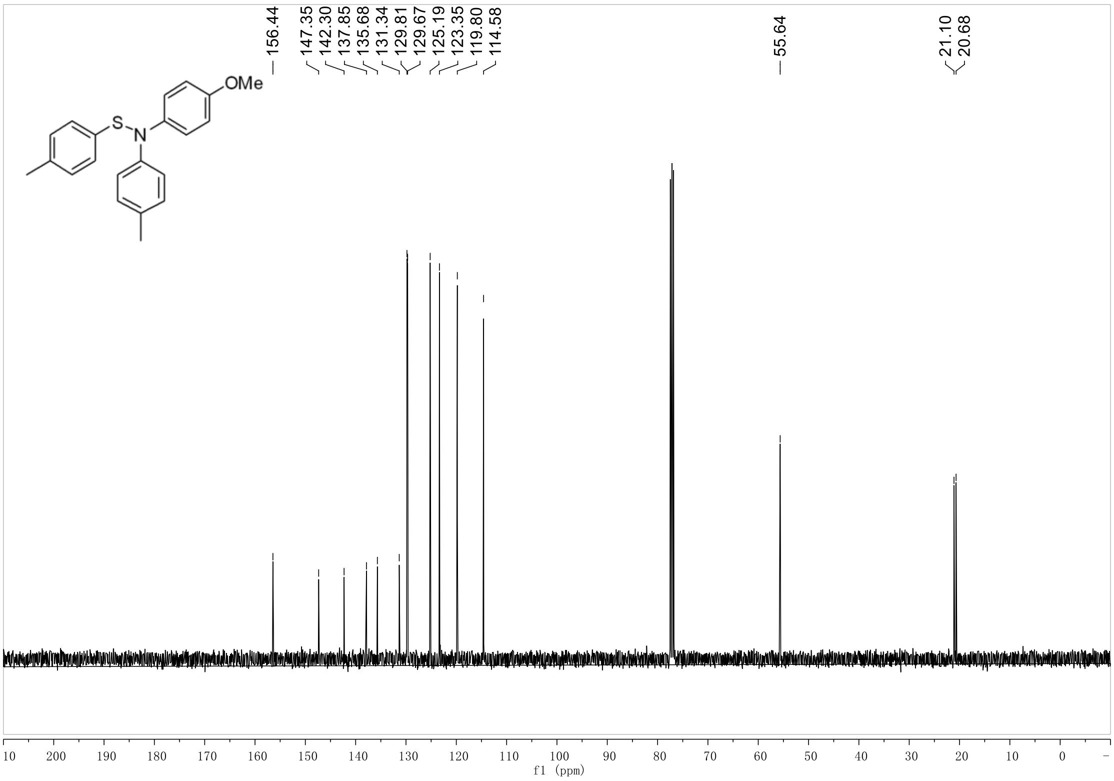

^{1}H NMR (400 MHz, $CDCl_3$) of *N*-(4-Fluorophenyl)-*N*,*S*-di-*p*-tolylthiohydroxylamine (3oa)

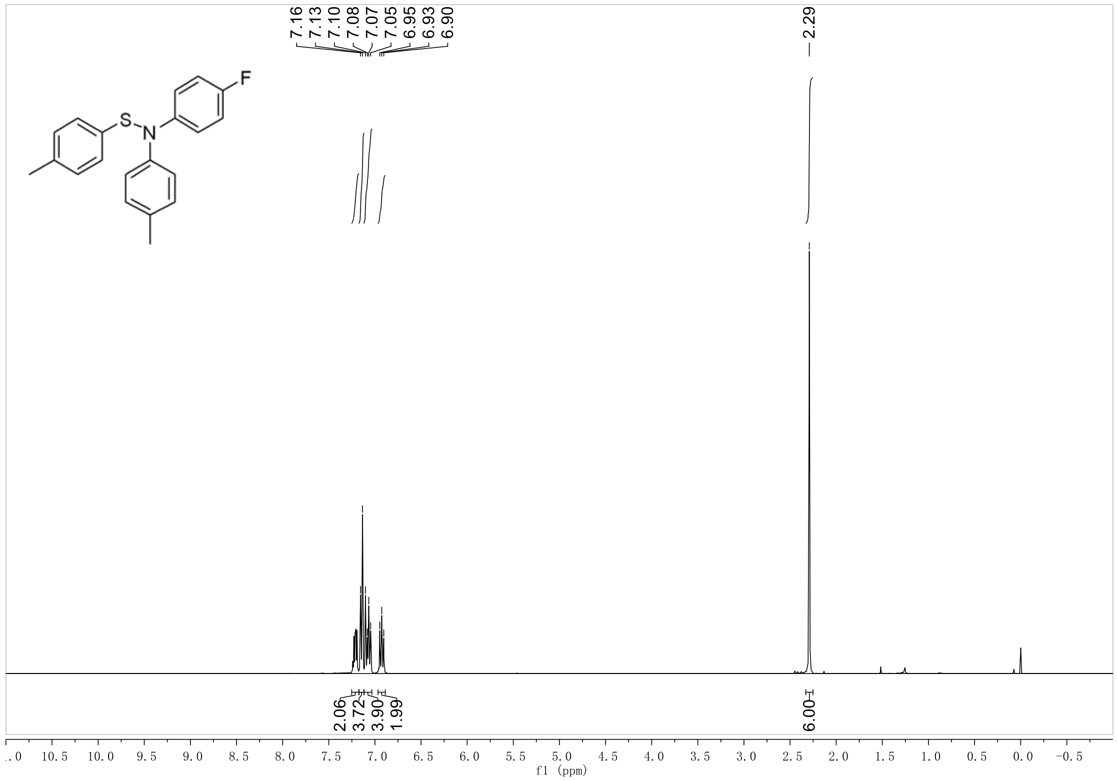

^{13}C NMR (100 MHz, $CDCl_3$) of *N*-(4-Fluorophenyl)-*N*,*S*-di-*p*-tolylthiohydroxylamine (3oa)

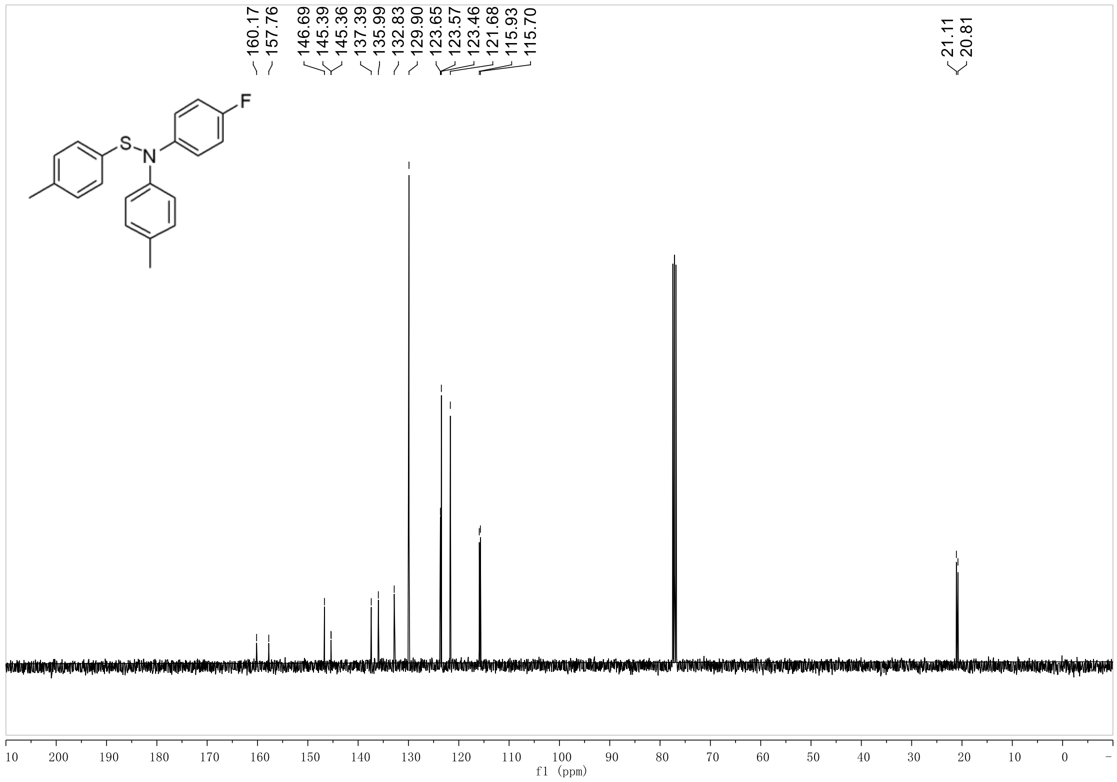

^{19}F NMR (377 MHz, $CDCl_3$) of *N*-(4-Fluorophenyl)-*N*,*S*-di-*p*-tolylthiohydroxylamine (3oa)

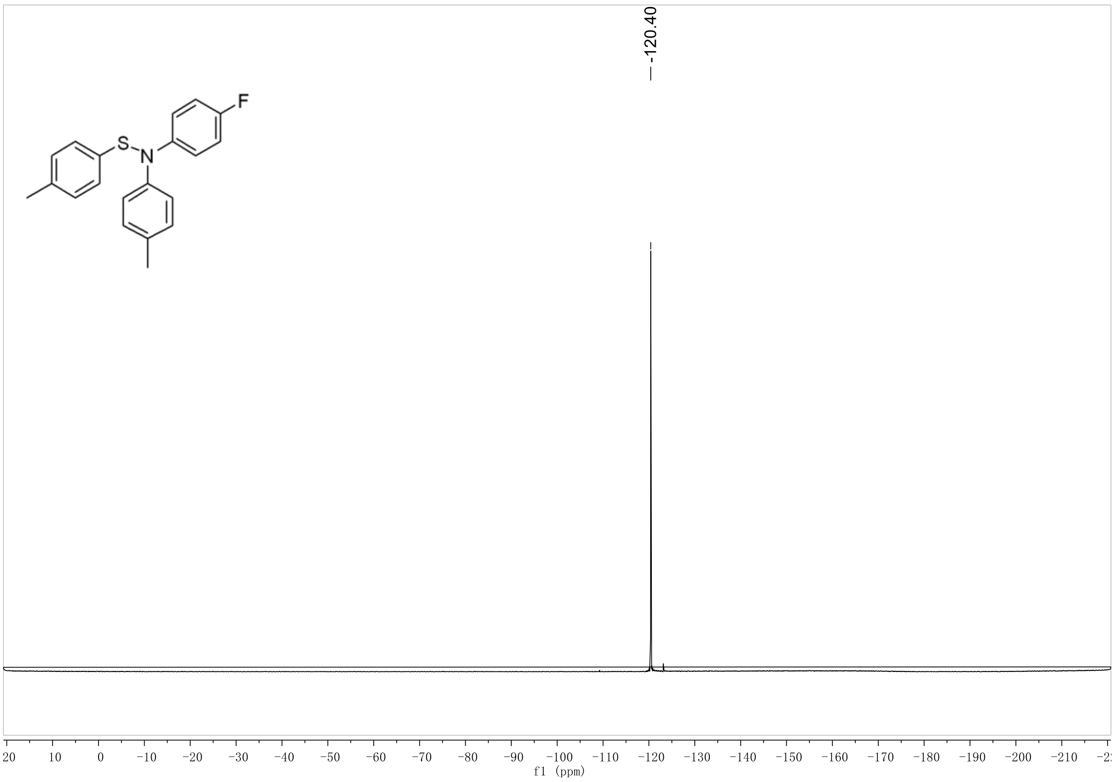

^{1}H NMR (400 MHz, $CDCl_3$) of *N*-(4-Chlorophenyl)-*N*,*S*-di-*p*-tolylthiohydroxylamine (3pa)

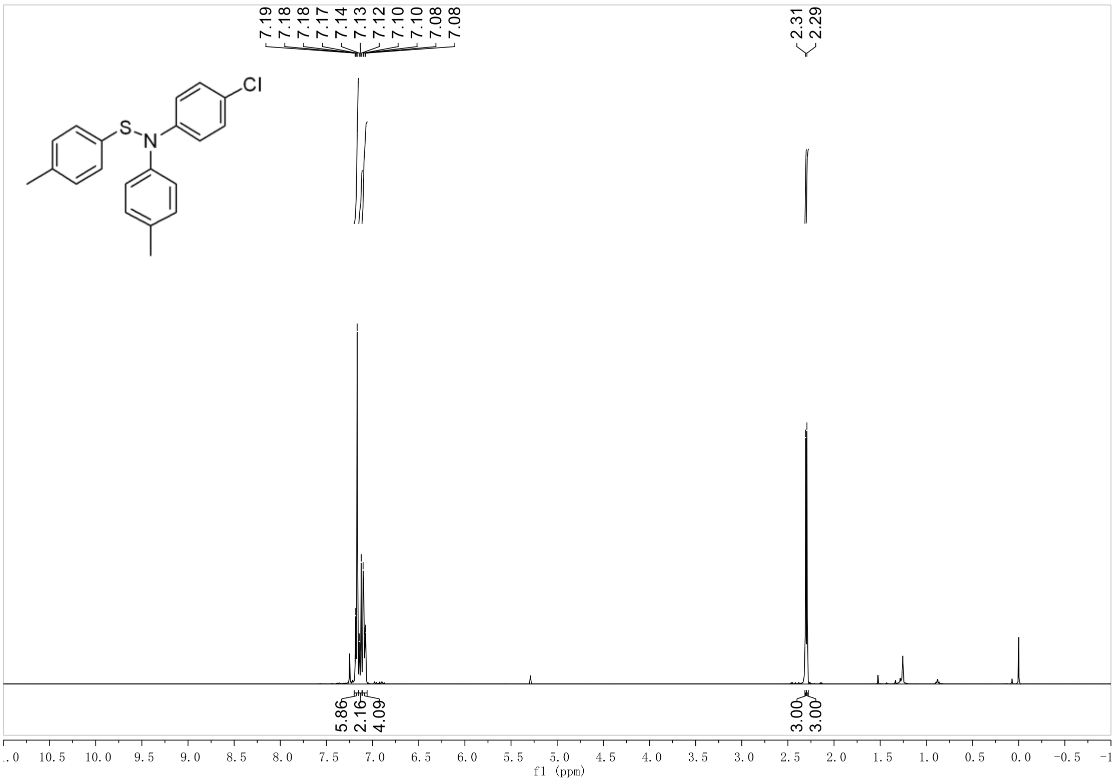

^{13}C NMR (100 MHz, $CDCl_3$) of *N*-(4-Chlorophenyl)-*N*,*S*-di-*p*-tolylthiohydroxylamine (3pa)

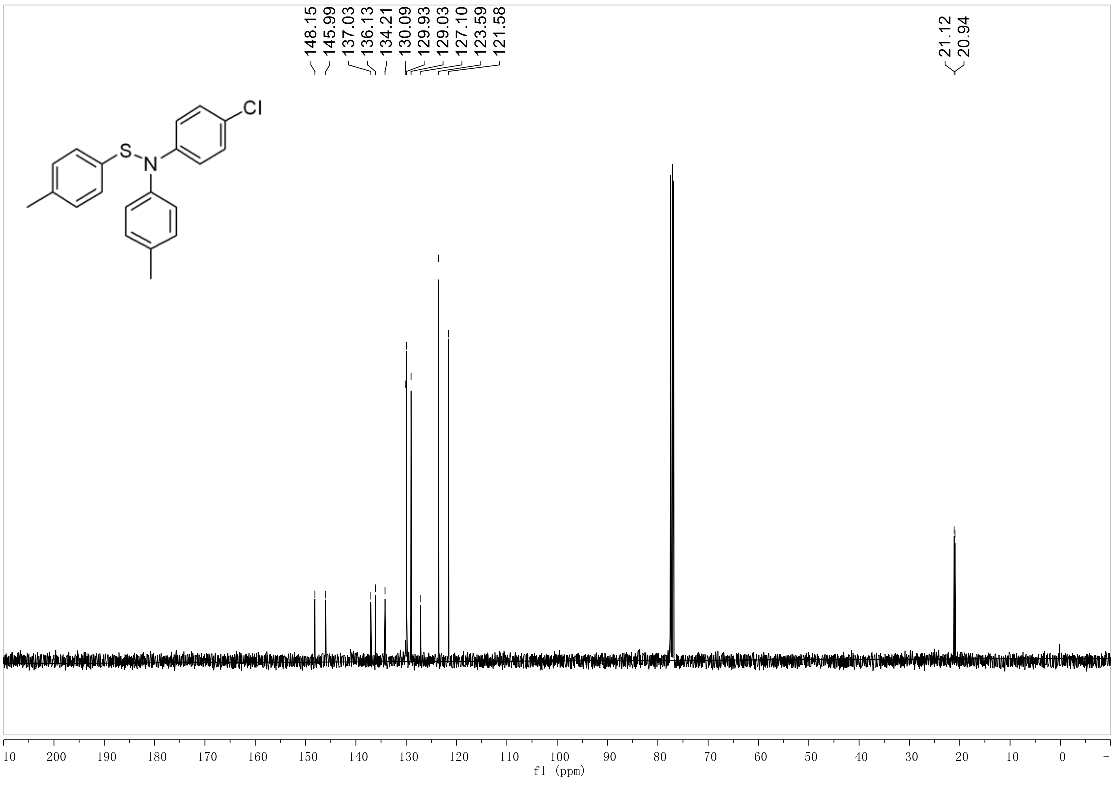

^{1}H NMR (600 MHz, $CDCl_3$) of *N*-(*o*-Tolyl)-*N*,*S*-di-*p*-tolylthiohydroxylamine (3qa)

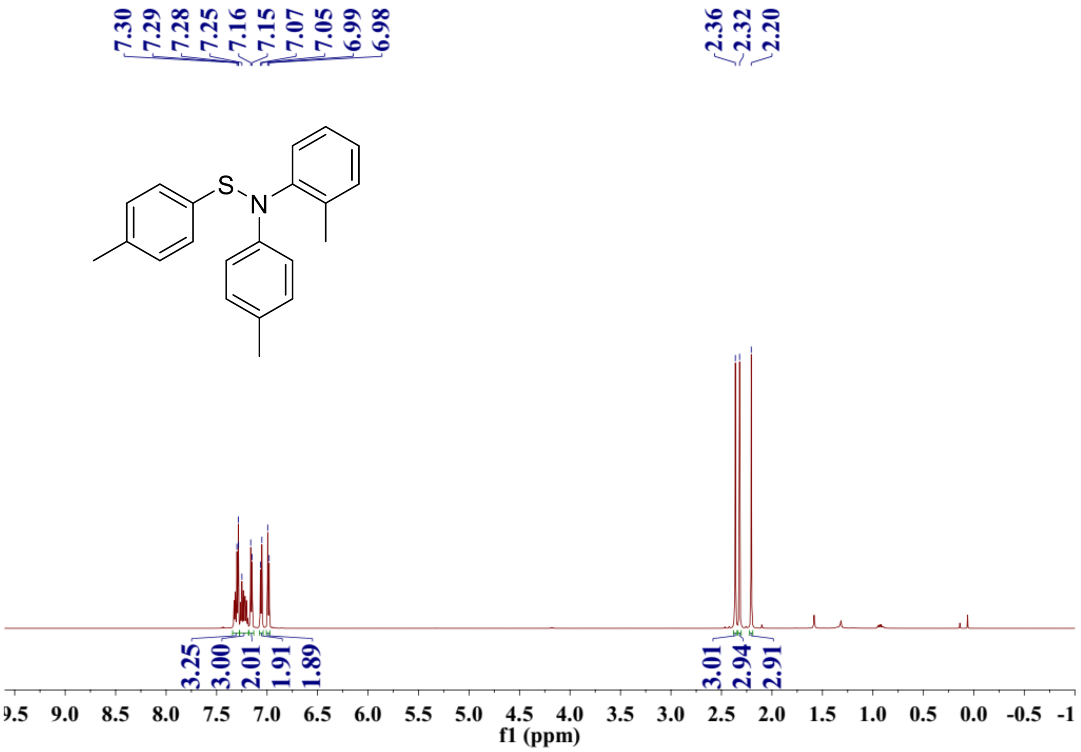

^{13}C NMR (150 MHz, $CDCl_3$) of *N*-(*o*-Tolyl)-*N*,*S*-di-*p*-tolylthiohydroxylamine (3qa)

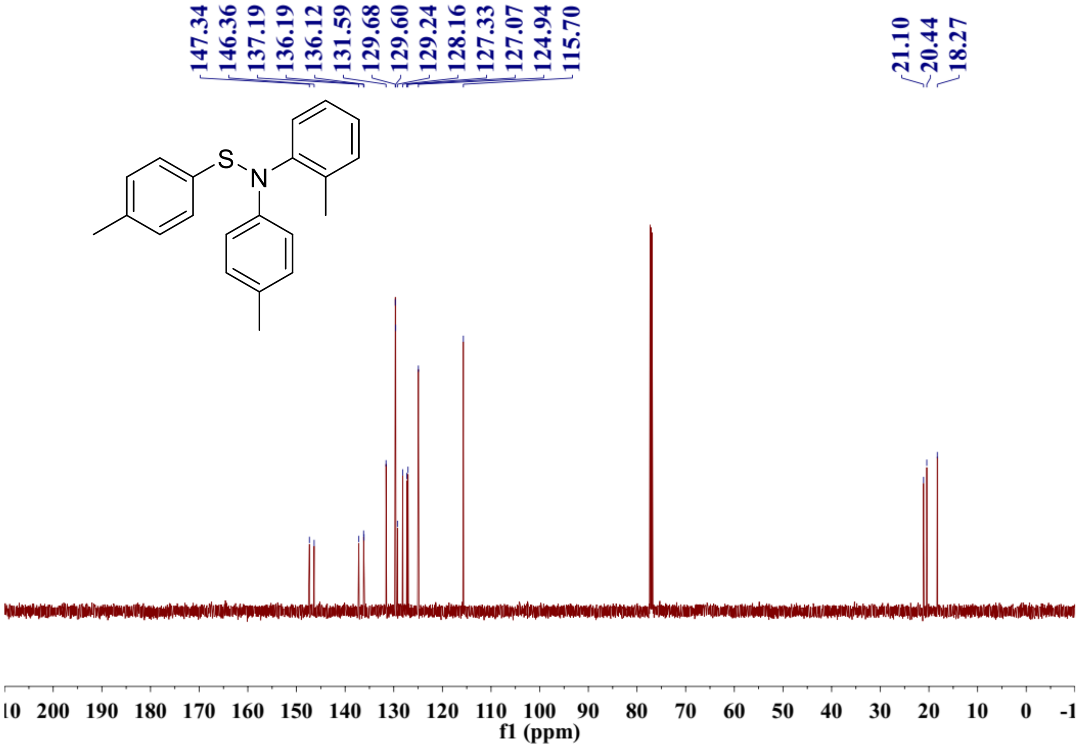

^{1}H NMR (400 MHz, $CDCl_3$) of *N*-(2-Fluorophenyl)-*N*,*S*-di-*p*-tolylthiohydroxylamine (3ra)

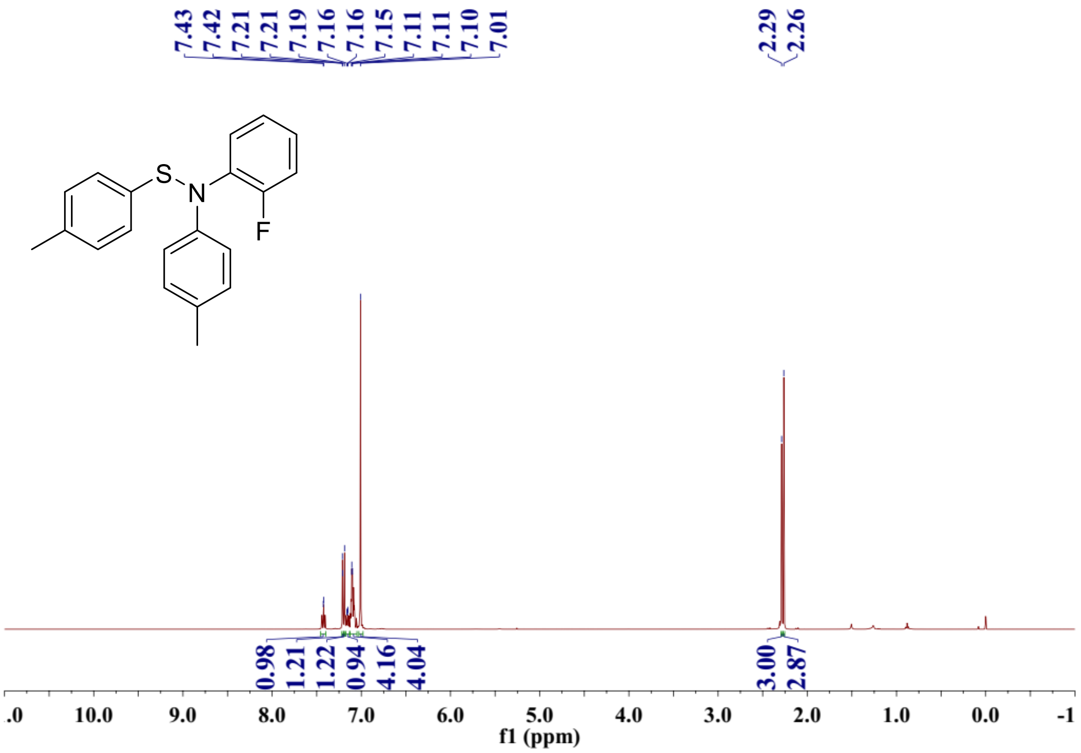

^{13}C NMR (100 MHz, $CDCl_3$) of *N*-(2-Fluorophenyl)-*N*,*S*-di-*p*-tolylthiohydroxylamine (3ra)

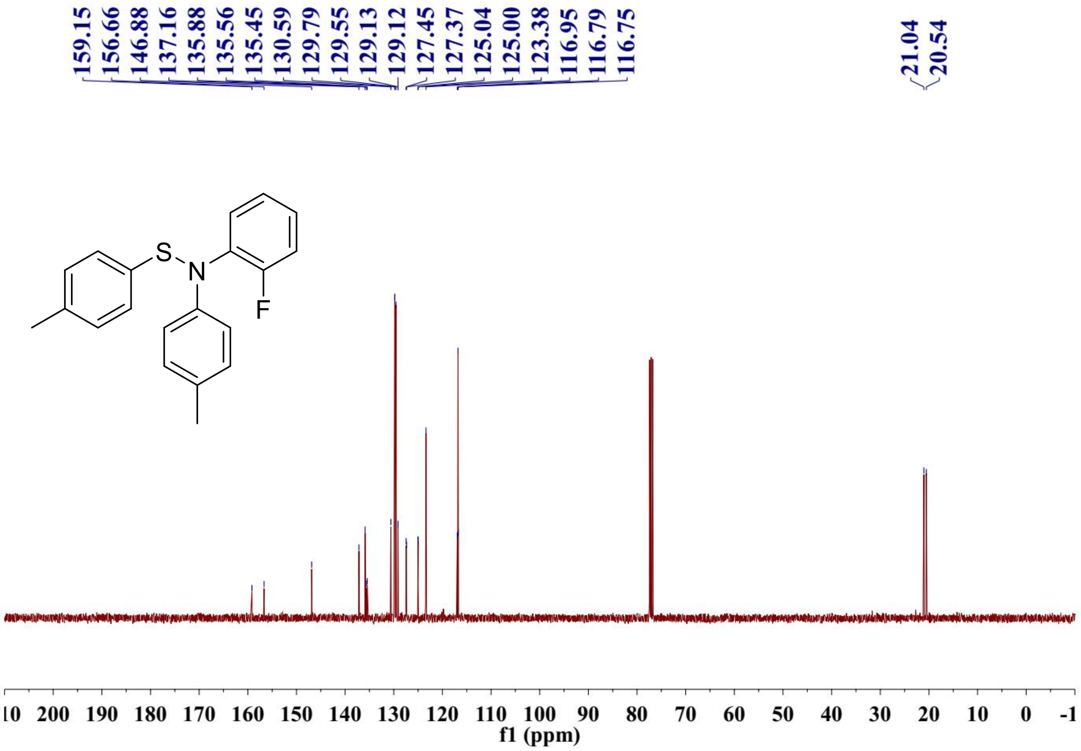

^{19}F NMR (377 MHz, $CDCl_3$) of *N*-(2-Fluorophenyl)-*N*,*S*-di-*p*-tolylthiohydroxylamine (3ra)

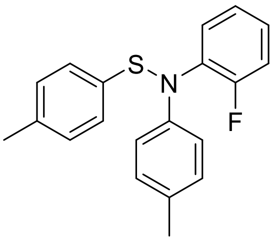

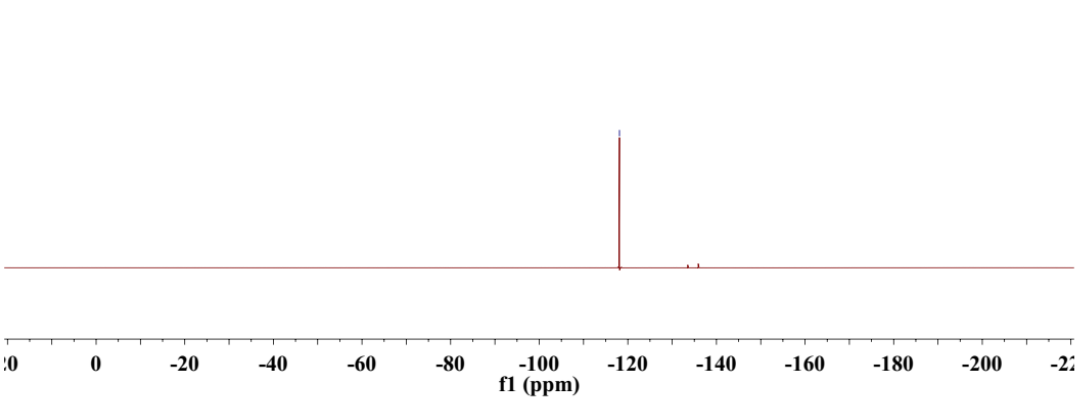

^{1}H NMR (400 MHz, $CDCl_3$) of Methyl 4-(*p*-tolyl(*p*-tolylthio)amino)benzoate (3sa)

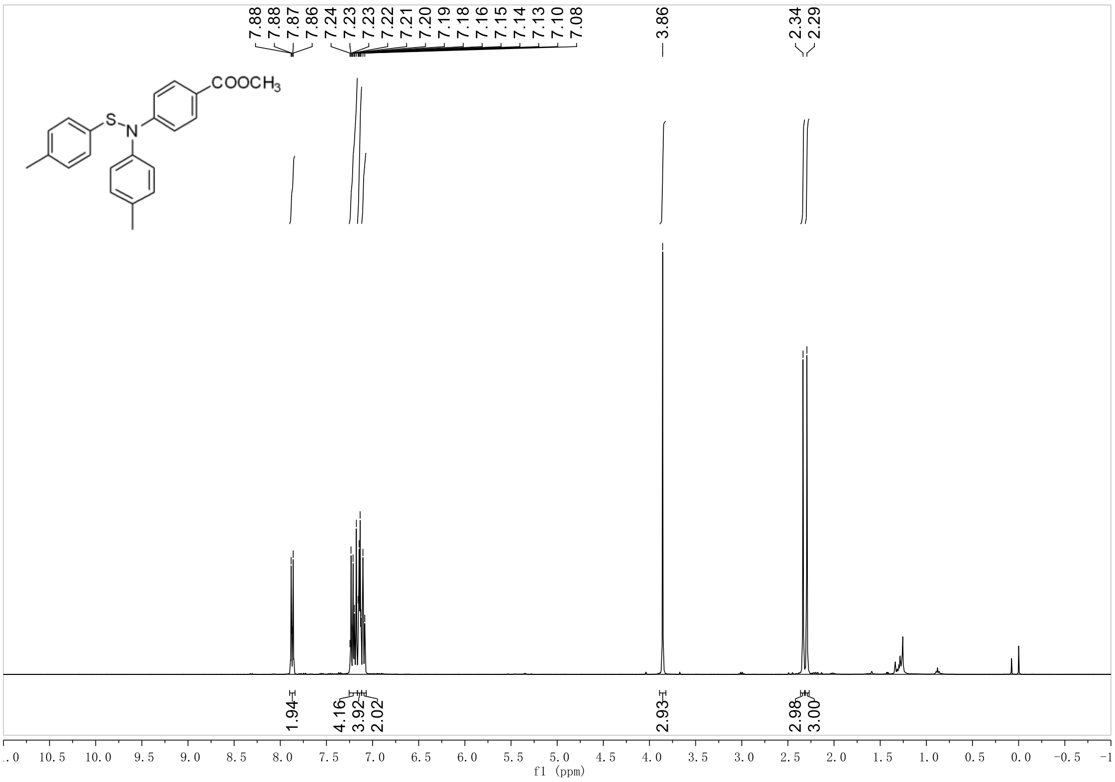

^{13}C NMR (100 MHz, $CDCl_3$) of Methyl 4-(*p*-tolyl(*p*-tolylthio)amino)benzoate (3sa)

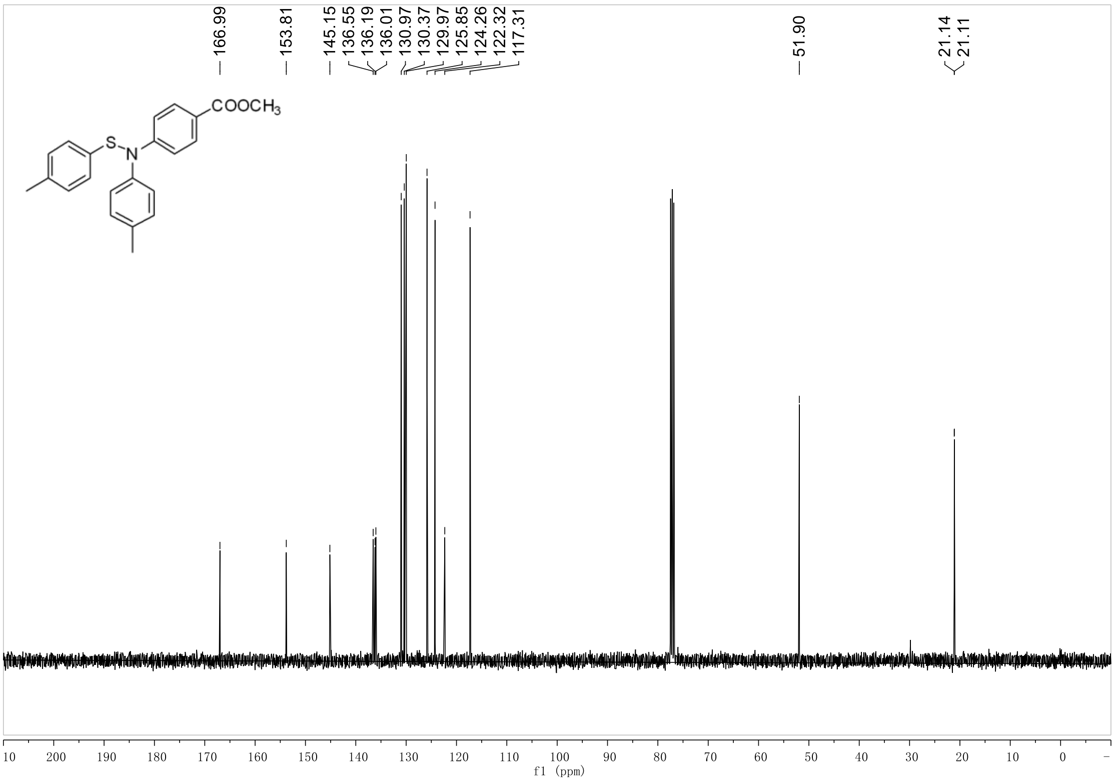

^{1}H NMR (400 MHz, $CDCl_3$) of 4-(*p*-Tolyl(*p*-tolylthio)amino)benzonitrile (3ta)

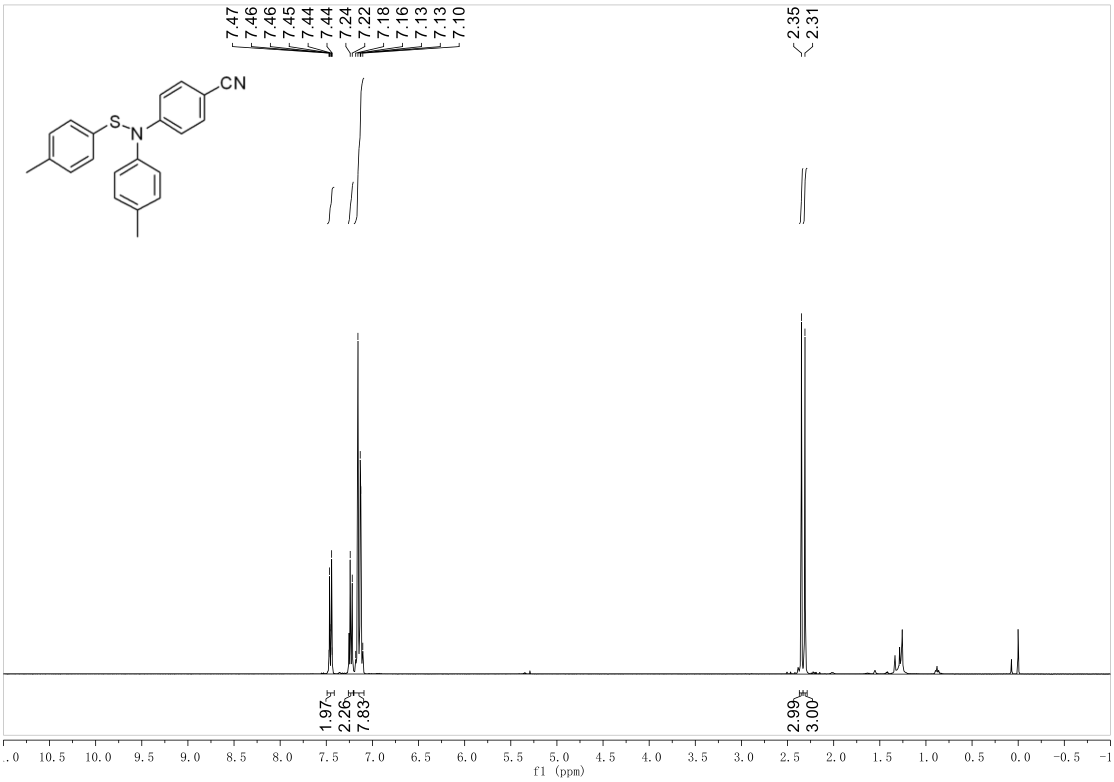

^{13}C NMR (100 MHz, $CDCl_3$) of 4-(*p*-Tolyl(*p*-tolylthio)amino)benzonitrile (3ta)

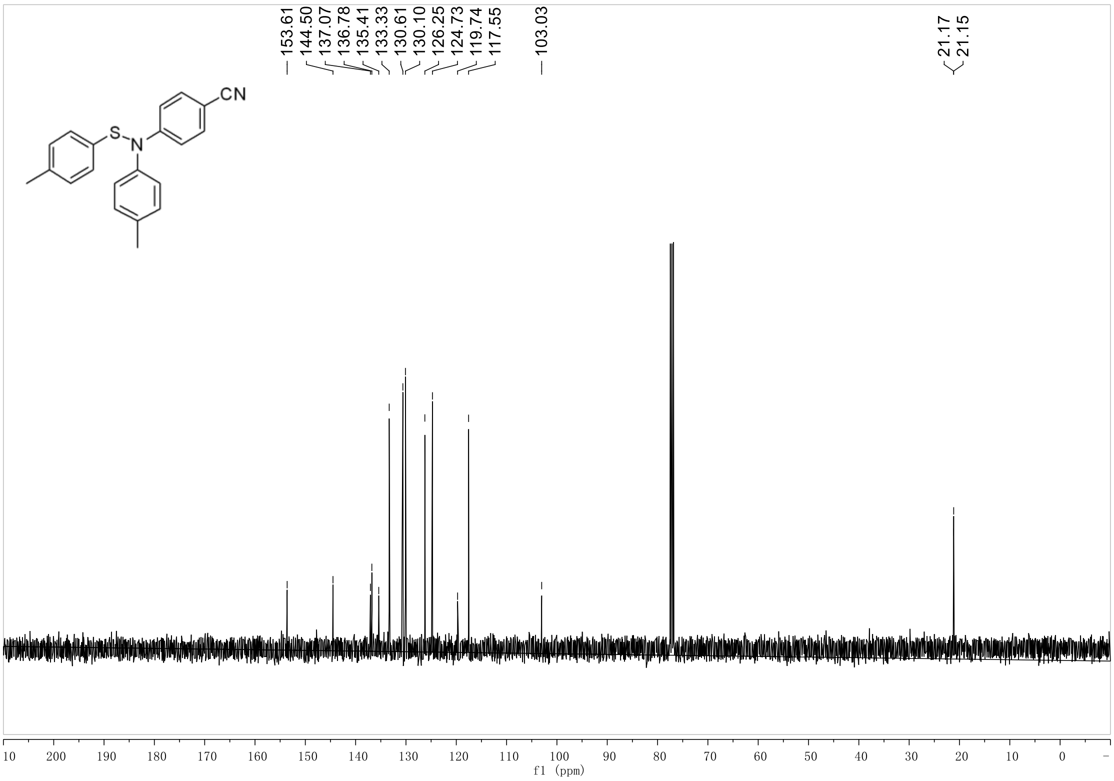

^{1}H NMR (400 MHz, $CDCl_3$) of 1-(4-(*p*-Tolyl(*p*-tolylthio)amino)phenyl)ethan-1-one (3ua)

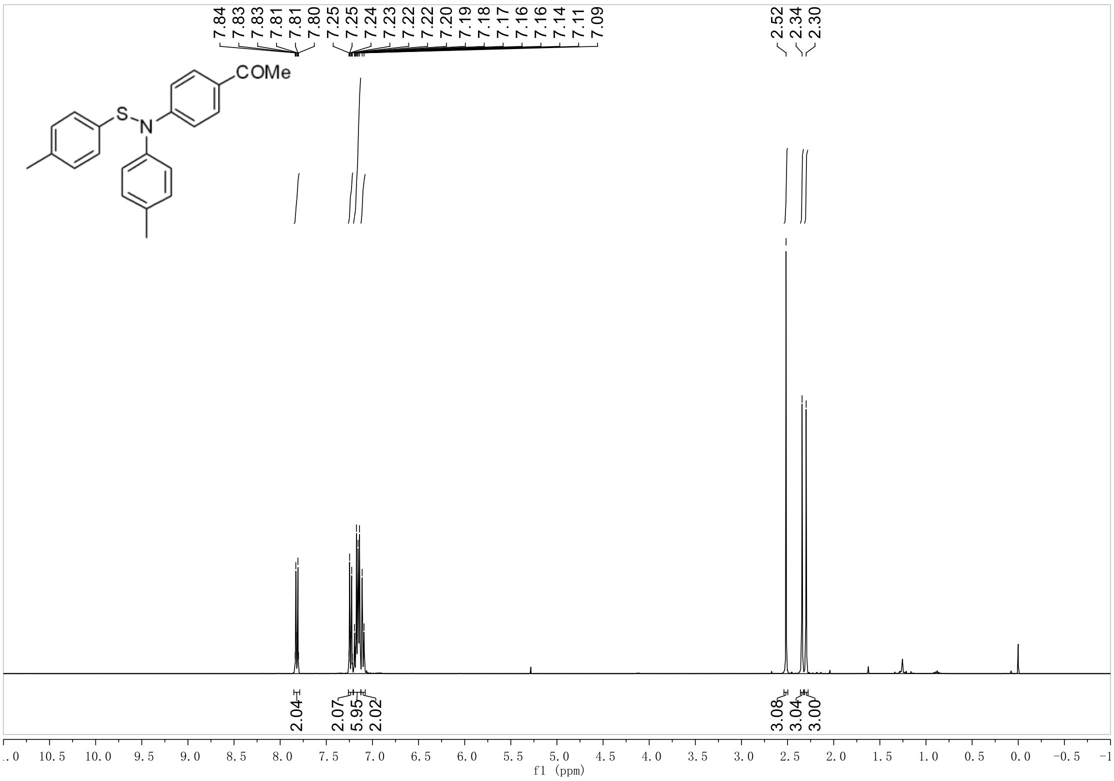

^{13}C NMR (100 MHz, $CDCl_3$) of 1-(4-(*p*-Tolyl(*p*-tolylthio)amino)phenyl)ethan-1-one (3ua)

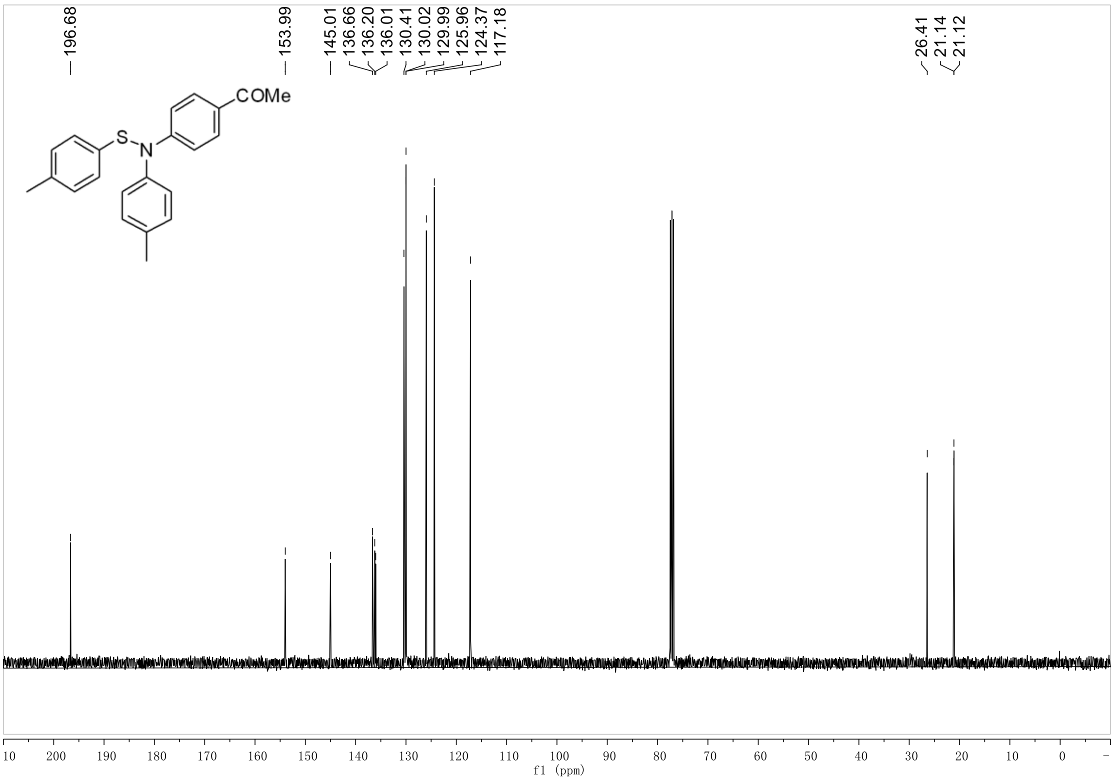

^{1}H NMR (400 MHz, CDCl$_3$) of *N*-(4-(Methylsulfonyl)phenyl)-*N*,*S*-di-*p*-tolylthiohydroxylamine (3va)

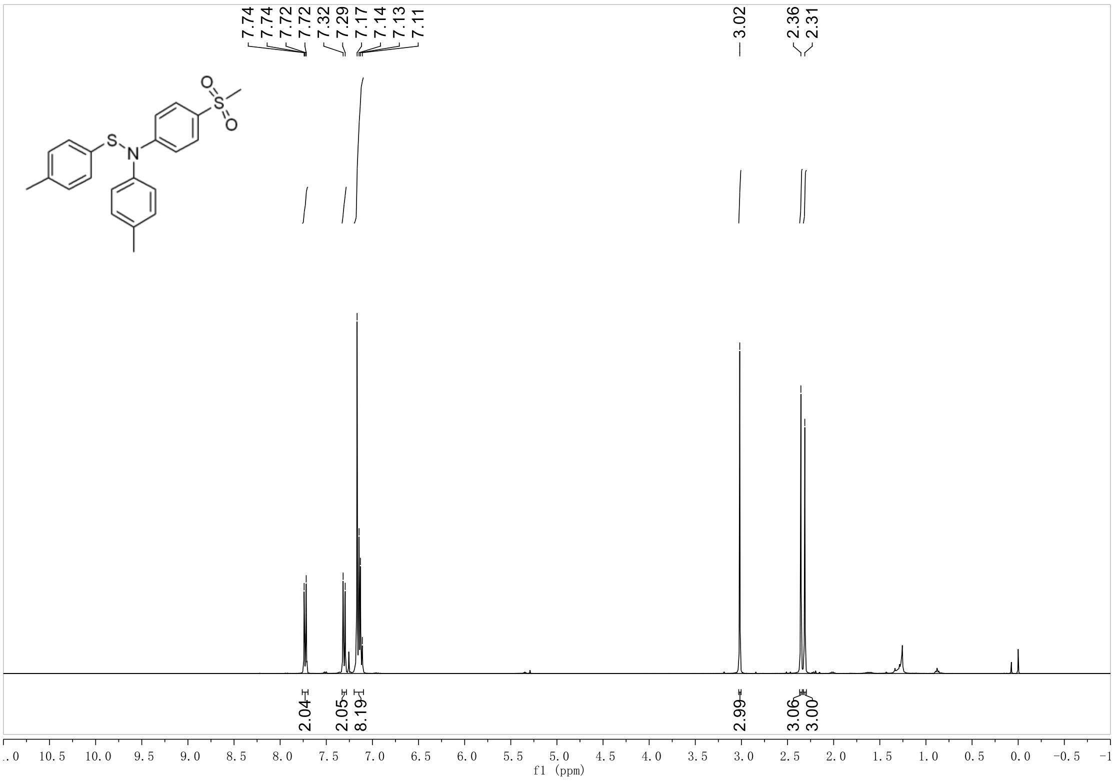

^{13}C NMR (100 MHz, CDCl$_3$) of *N*-(4-(Methylsulfonyl)phenyl)-*N*,*S*-di-*p*-tolylthiohydroxylamine (3va)

`

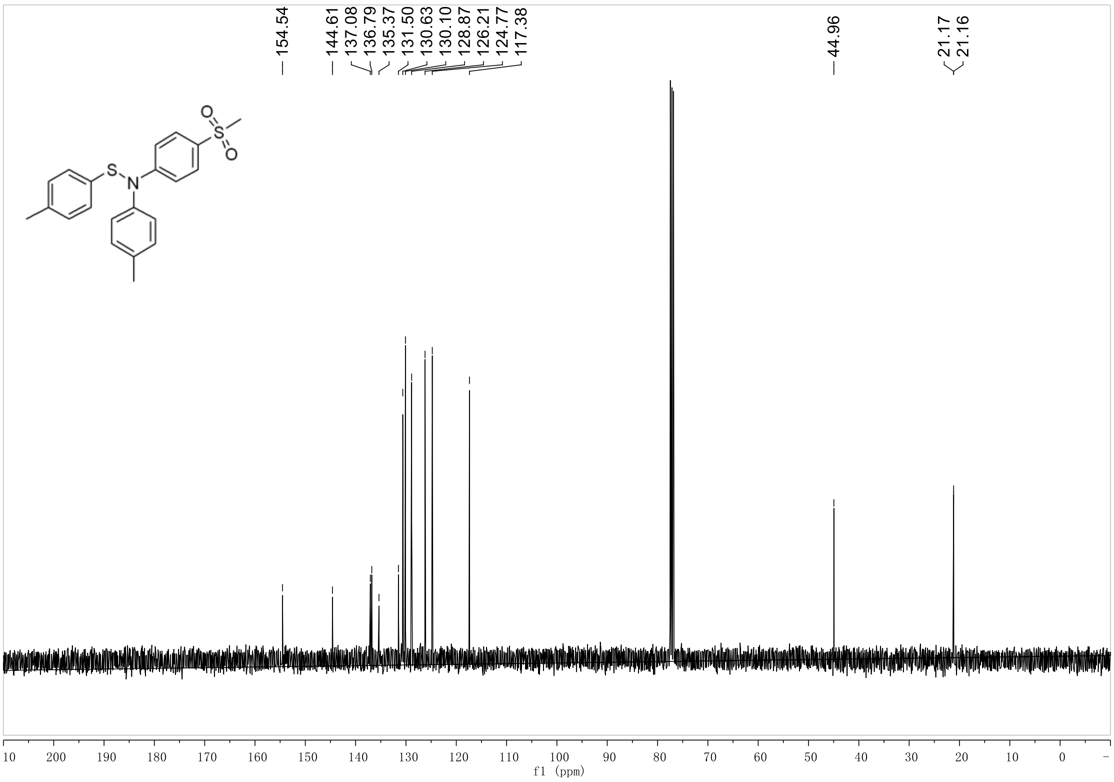

^{1}H NMR (400 MHz, $CDCl_3$) of Morpholino(4-(*p*-tolyl(*p*-tolylthio)amino)phenyl)methanone (3wa)

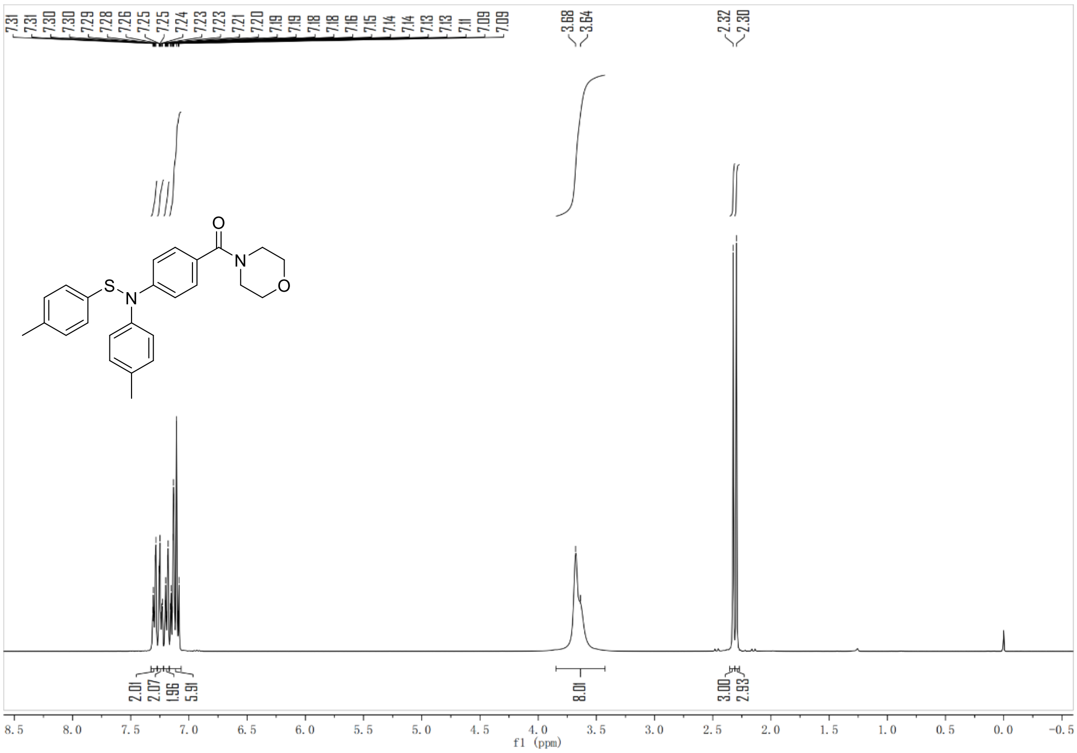

^{13}C NMR (100 MHz, $CDCl_3$) of Morpholino(4-(*p*-tolyl(*p*-tolylthio)amino)phenyl)methanone (3wa)

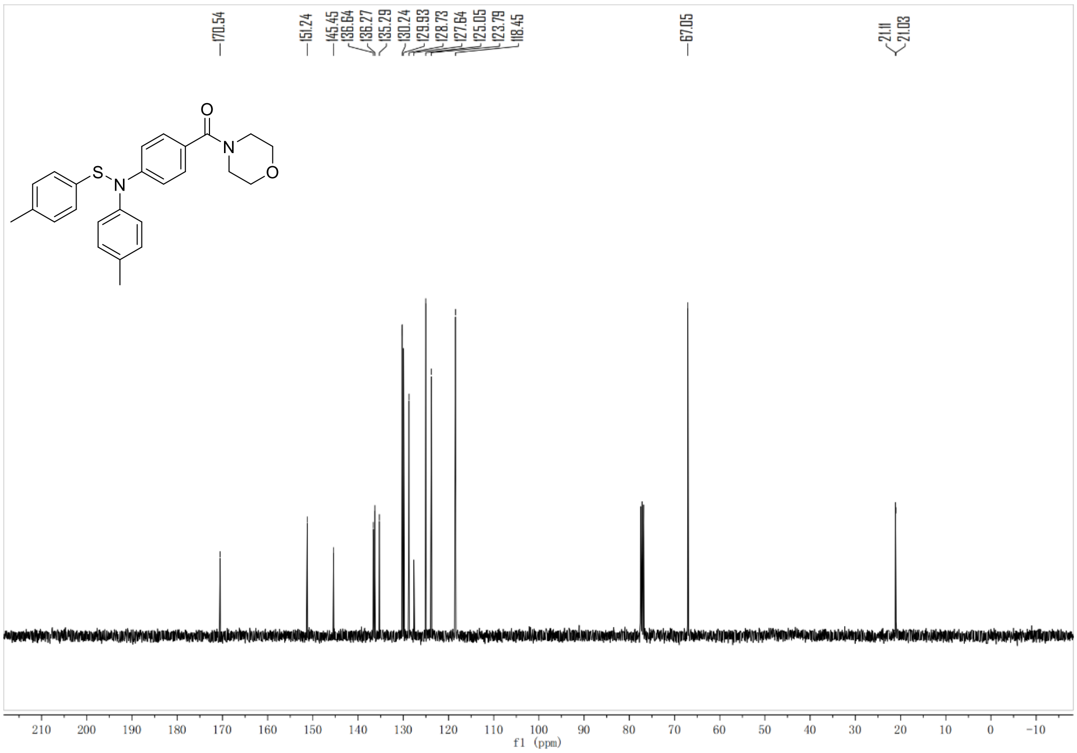

^{1}H NMR (400 MHz, $CDCl_3$) of *tert*-Butyl 5-(*p*-tolyl(*p*-tolylthio)amino)-1*H*-indole-1-carboxylate (3xa)

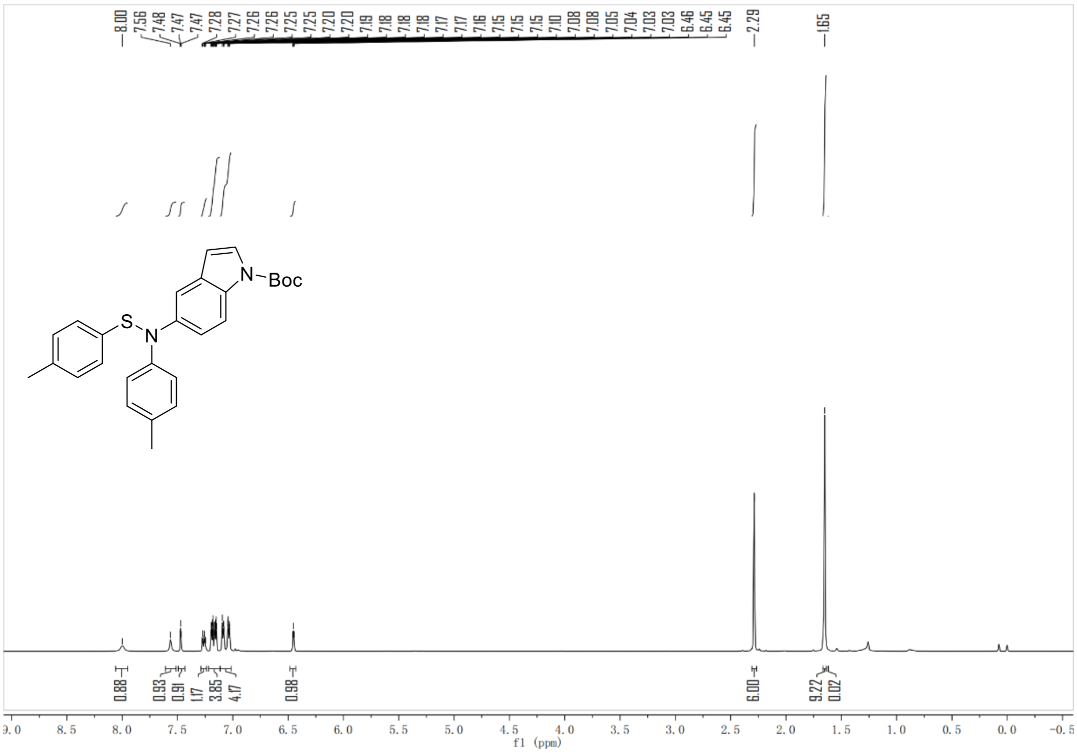

^{13}C NMR (100 MHz, $CDCl_3$) of *tert*-Butyl 5-(*p*-tolyl(*p*-tolylthio)amino)-1*H*-indole-1-carboxylate (3xa)

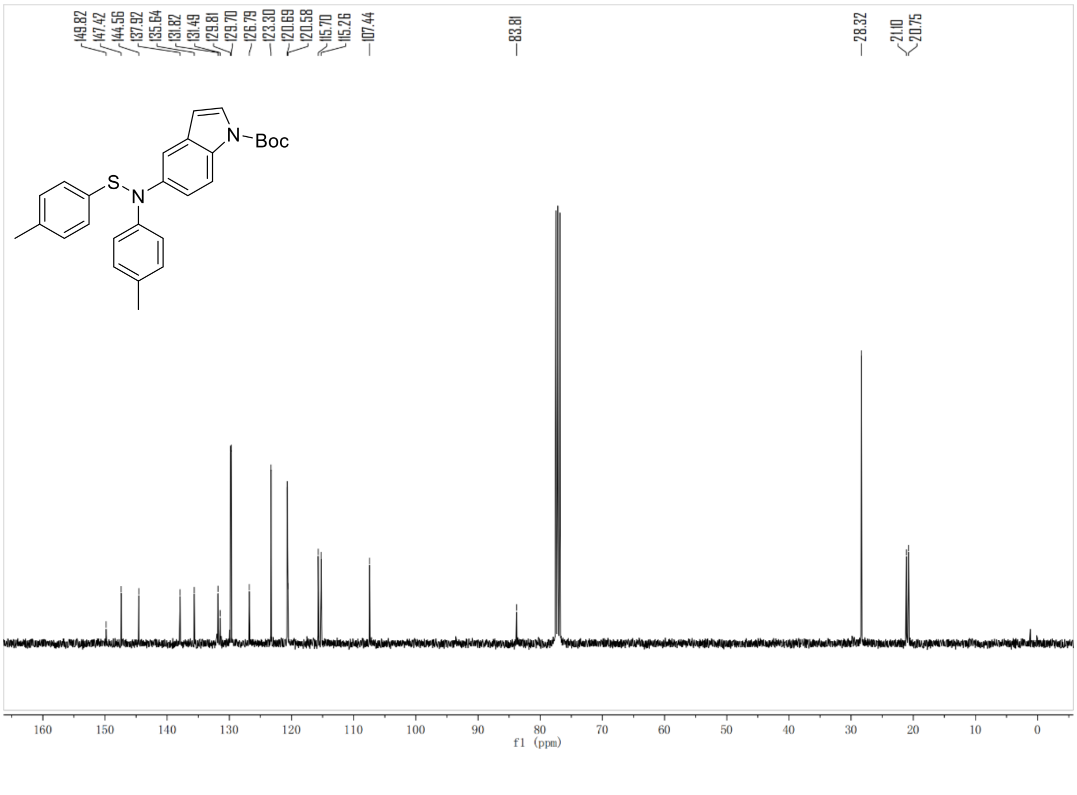

^{1}H NMR (400 MHz, $CDCl_3$) of *N*-(4-Fluorophenyl)-*N*-methyl-*S*-phenylthiohydroxylamine (3yd)

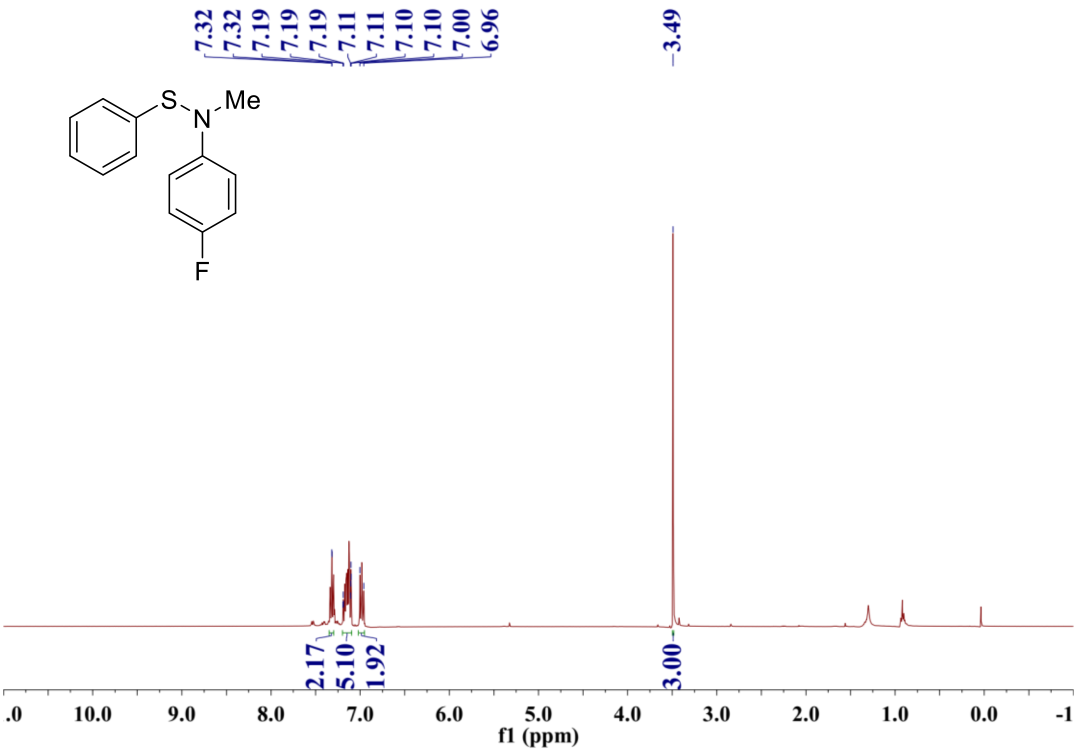

^{13}C NMR (100 MHz, $CDCl_3$) of *N*-(4-Fluorophenyl)-*N*-methyl-*S*-phenylthiohydroxylamine (3yd)

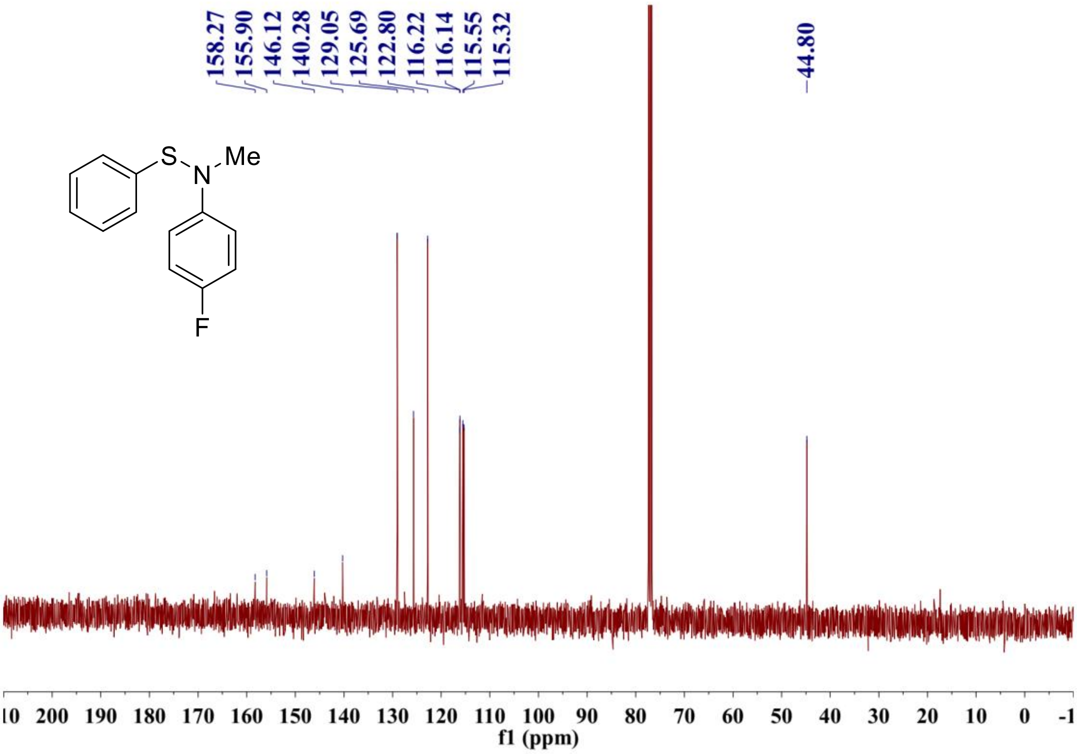

^{19}F NMR (377 MHz, CDCl$_3$) of *N*-(4-Fluorophenyl)-*N*-methyl-*S*-phenylthiohydroxylamine (3yd)

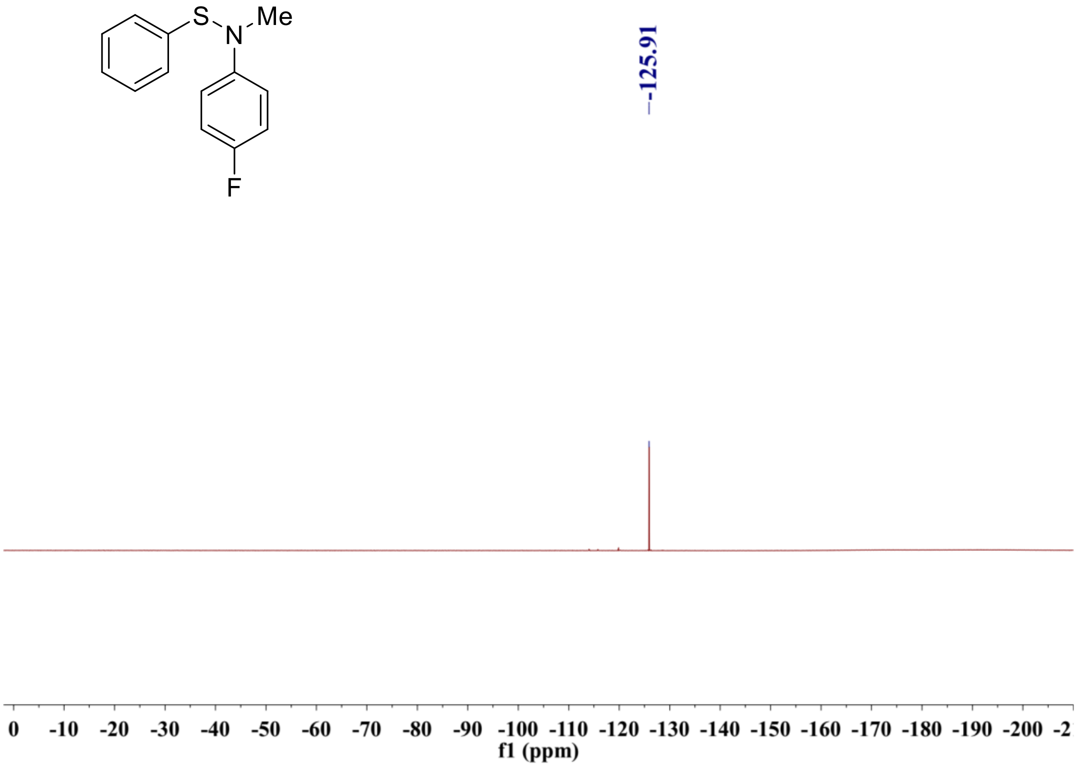

^{1}H NMR (400 MHz, $CDCl_3$) of *N*-(*tert*-Butyl)-*N*-(4-fluorophenyl)-*S*-phenylthiohydroxylamine (3zd)

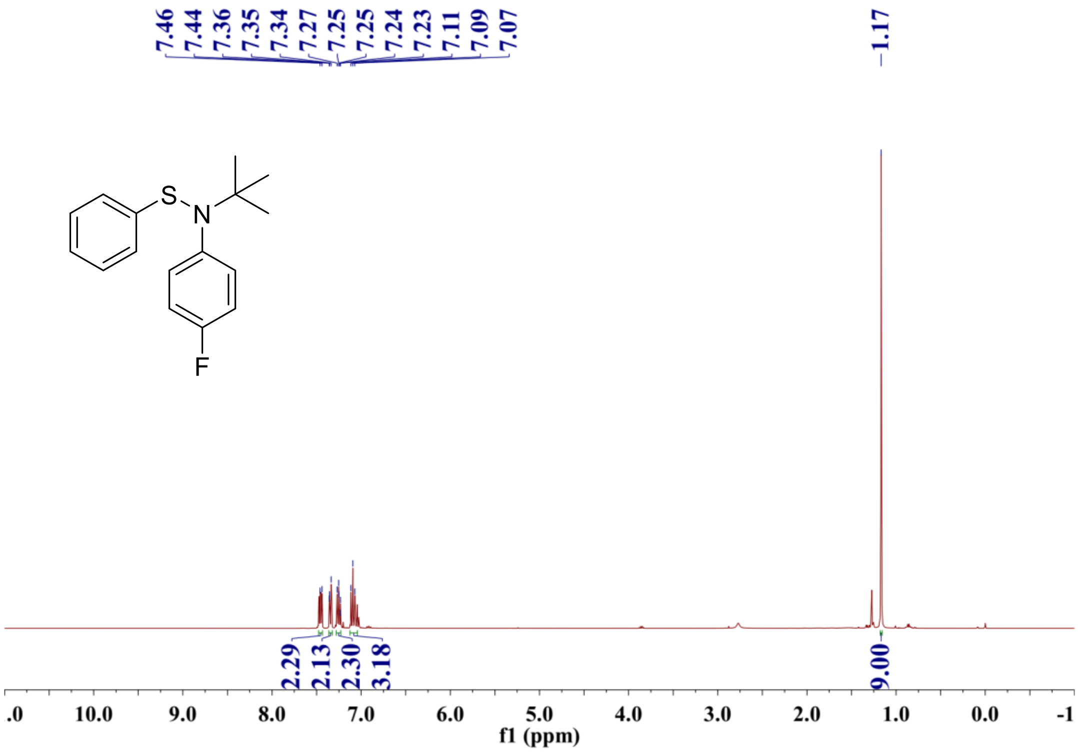

^{13}C NMR (150 MHz, $CDCl_3$) of *N*-(*tert*-Butyl)-*N*-(4-fluorophenyl)-*S*-phenylthiohydroxylamine (3zd)

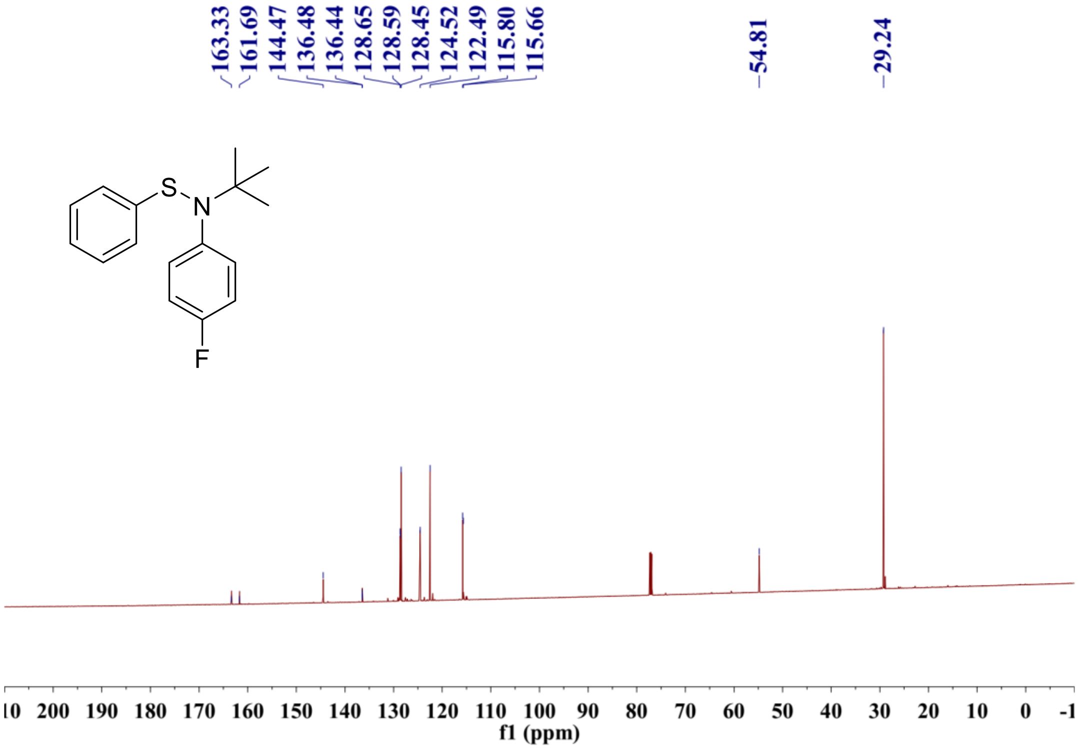

^{19}F NMR (377 MHz, $CDCl_3$) of *N*-(*tert*-Butyl)-*N*-(4-fluorophenyl)-*S*-phenylthiohydroxylamine (3zd)

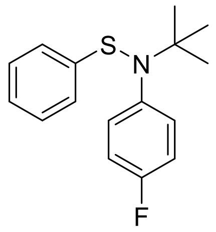

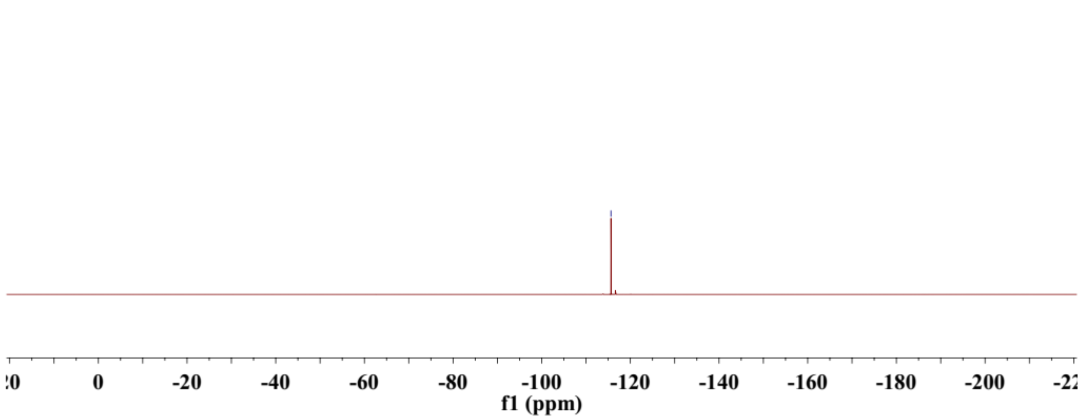

^{1}H NMR (600 MHz, $CDCl_3$) of *N*-Benzyl-*N*-(4-fluorophenyl)-*S*-(*p*-tolyl)thiohydroxylamine (3aad)

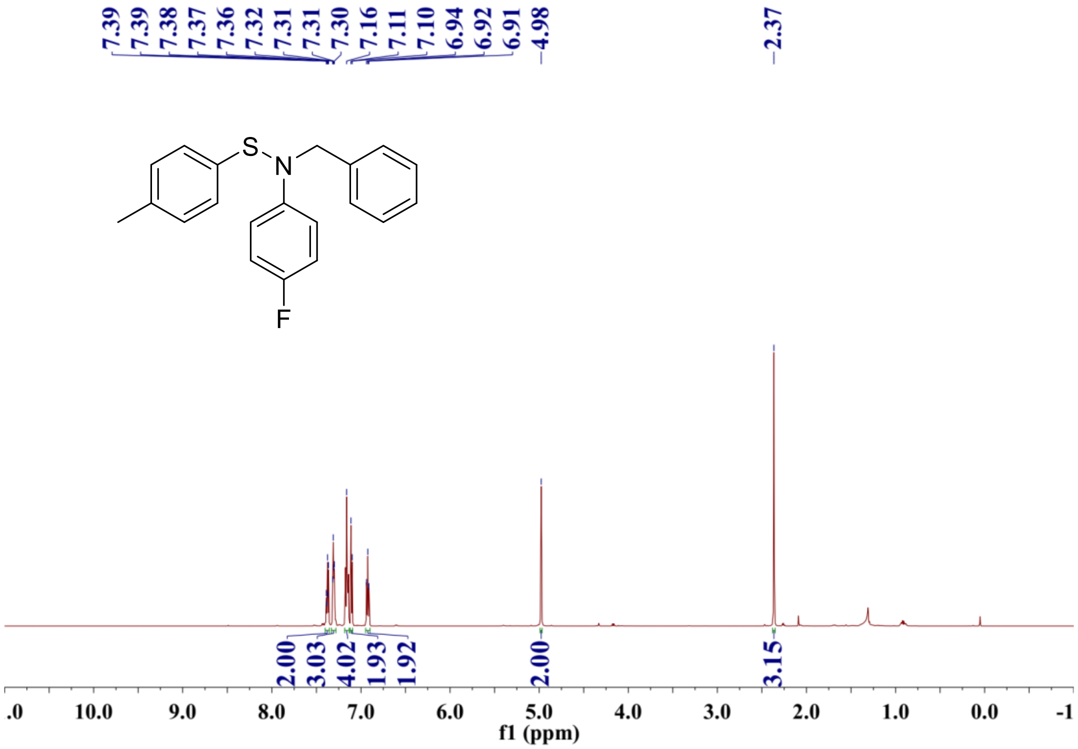

^{13}C NMR (100 MHz, $CDCl_3$) of *N*-Benzyl-*N*-(4-fluorophenyl)-*S*-(*p*-tolyl)thiohydroxylamine (3aad)

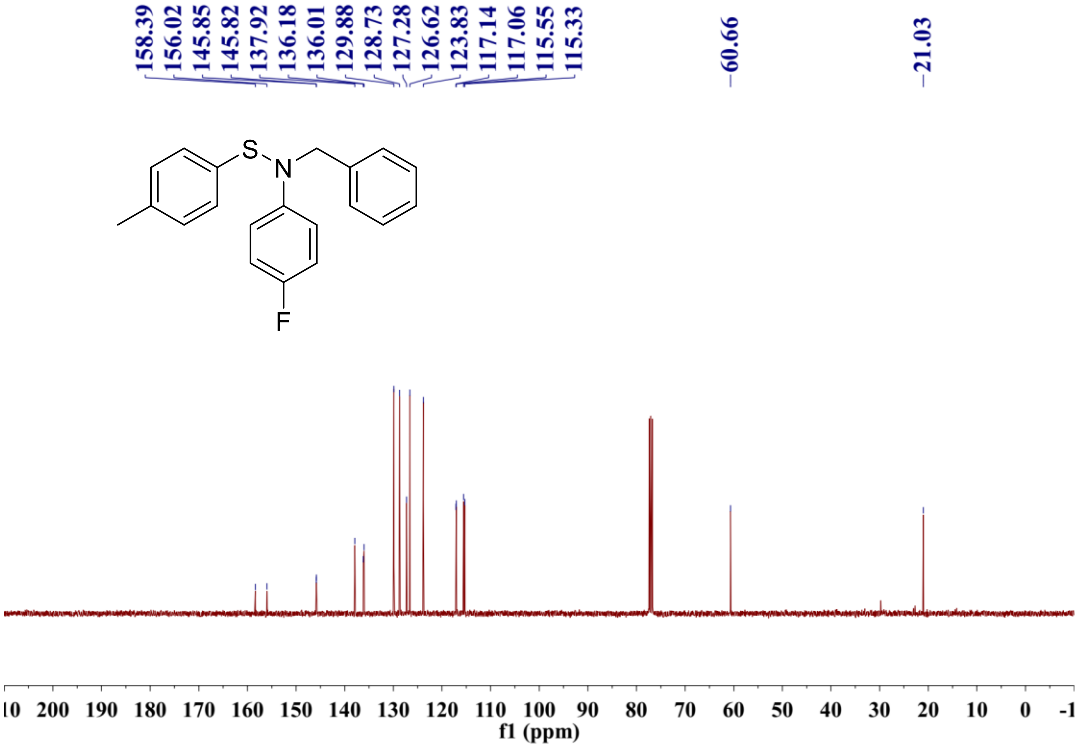

^{19}F NMR (377 MHz, $CDCl_3$) of *N*-Benzyl-*N*-(4-fluorophenyl)-*S*-(*p*-tolyl)thiohydroxylamine (3aad)

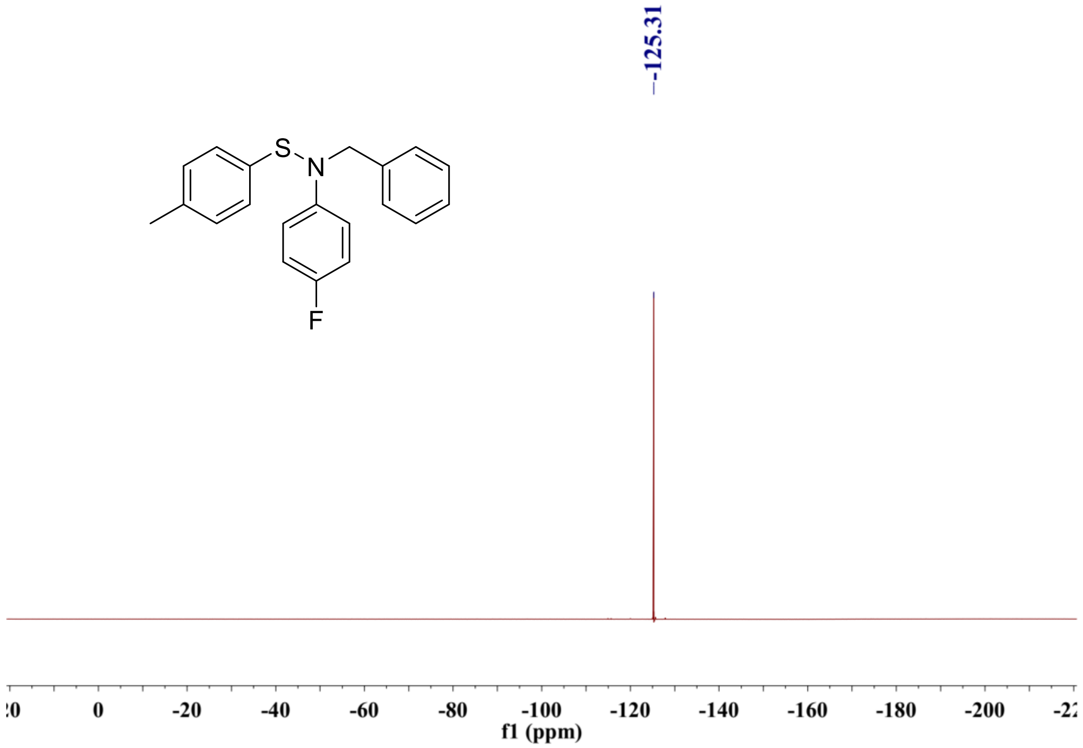

^{1}H NMR (600 MHz, $CDCl_3$) of *N*-(4-Fluorophenyl)-*N*-(2-nitrobenzyl)-*S*-(*p*-tolyl)thiohydroxylamine (3abd)

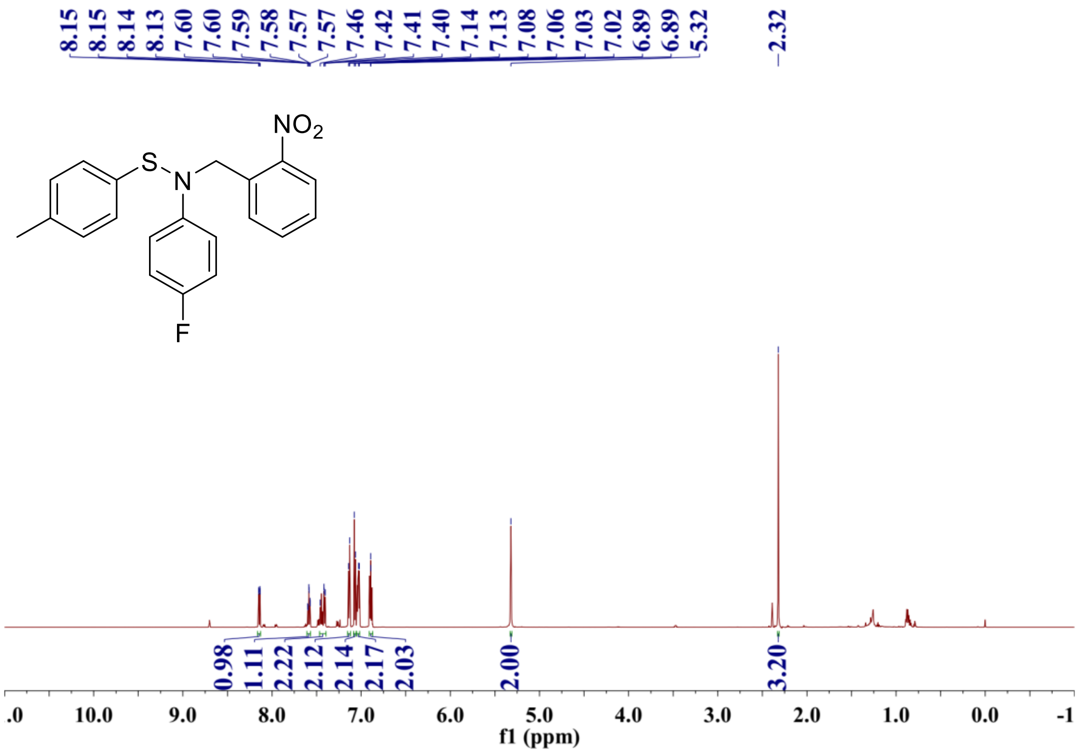

^{13}C NMR (100 MHz, $CDCl_3$) of *N*-(4-Fluorophenyl)-*N*-(2-nitrobenzyl)-*S*-(*p*-tolyl)thiohydroxylamine (3abd)

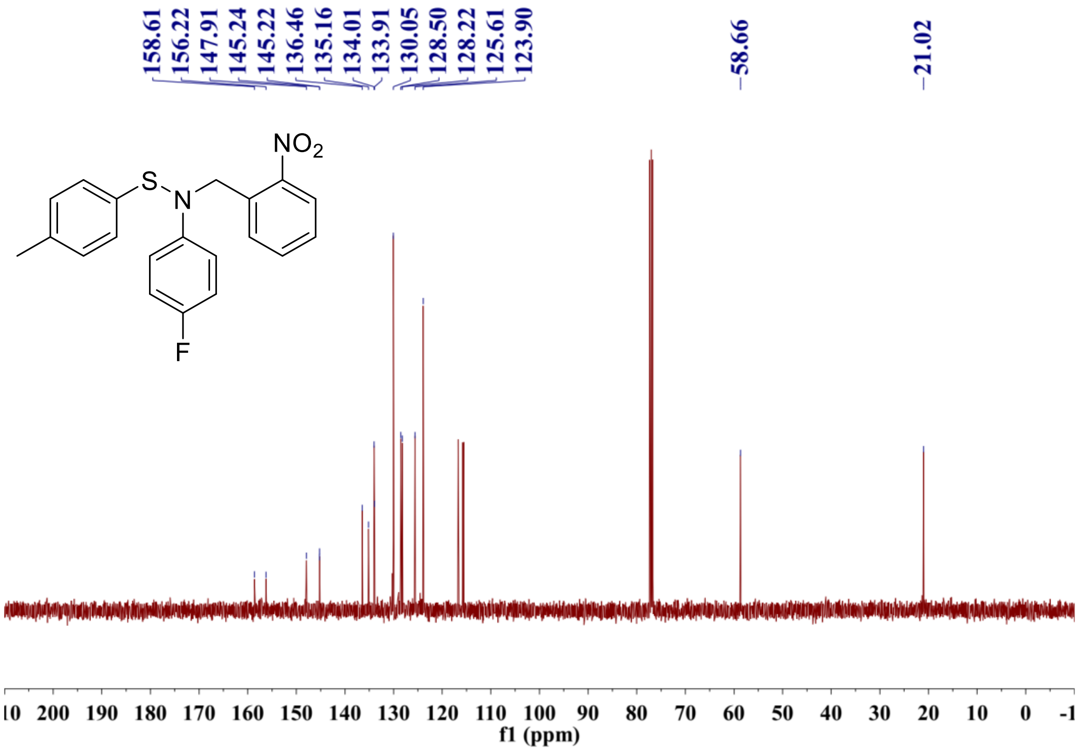

^{19}F NMR (377 MHz, $CDCl_3$) of *N*-(4-Fluorophenyl)-*N*-(2-nitrobenzyl)-*S*-(*p*-tolyl)thiohydroxylamine (3abd)

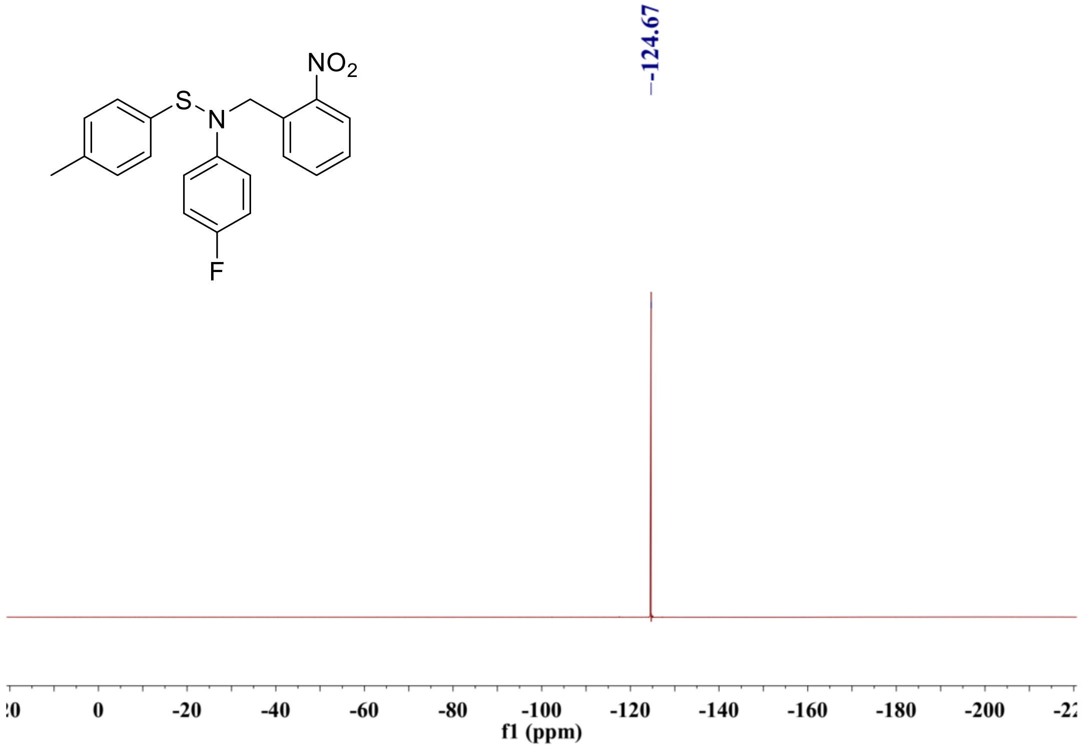

^{1}H NMR (400 MHz, $CDCl_3$) of *N*-(4-Fluorophenyl)-*N*-(pyridin-3-ylmethyl)-*S*-(*p*-tolyl)thiohydroxylamine (3acd)

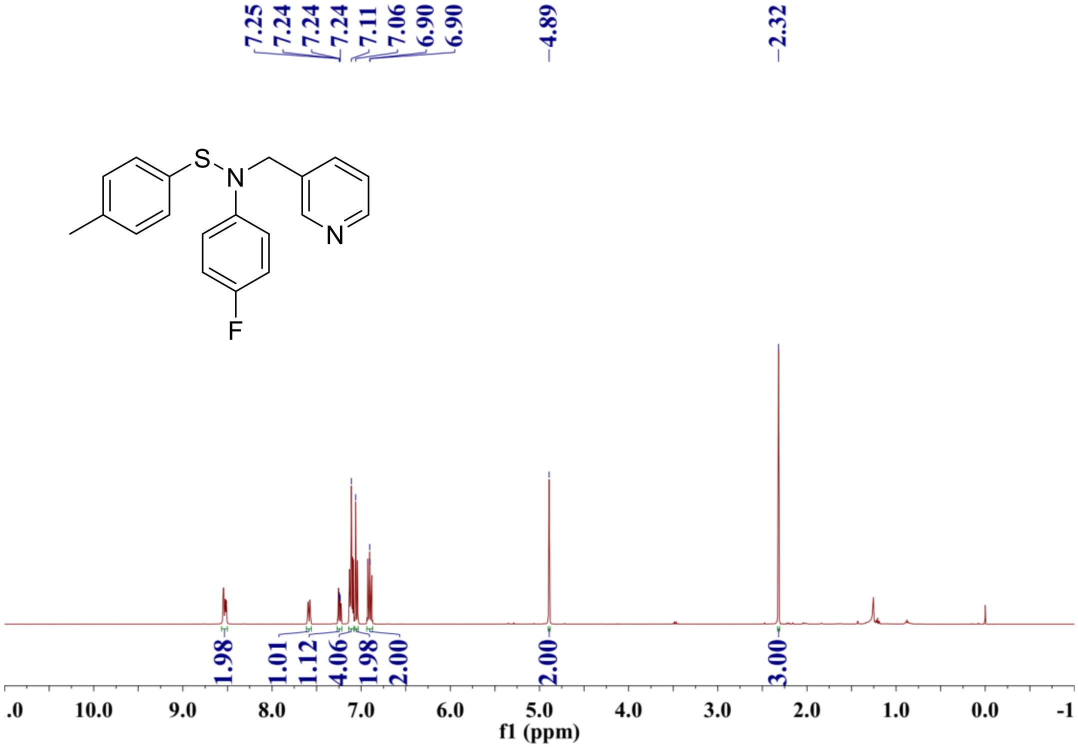

^{13}C NMR (100 MHz, $CDCl_3$) of *N*-(4-Fluorophenyl)-*N*-(pyridin-3-ylmethyl)-*S*-(*p*-tolyl)thiohydroxylamine (3acd)

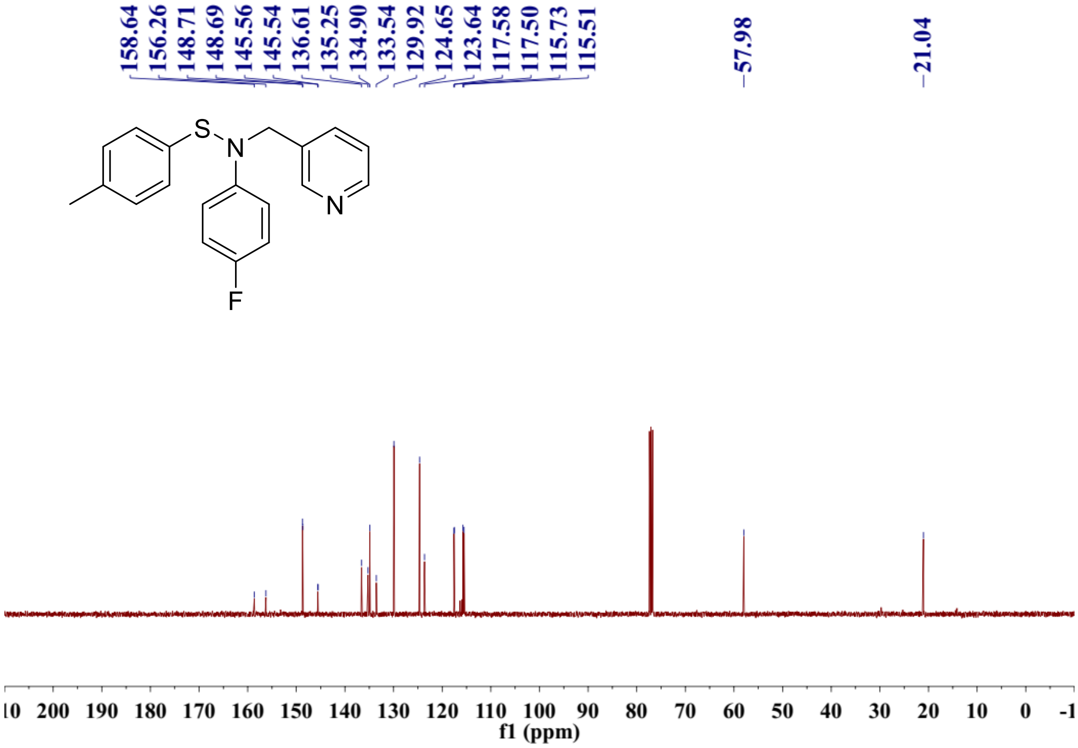

^{19}F NMR (377 MHz, $CDCl_3$) of *N*-(4-Fluorophenyl)-*N*-(pyridin-3-ylmethyl)-*S*-(*p*-tolyl)thiohydroxylamine (3acd)

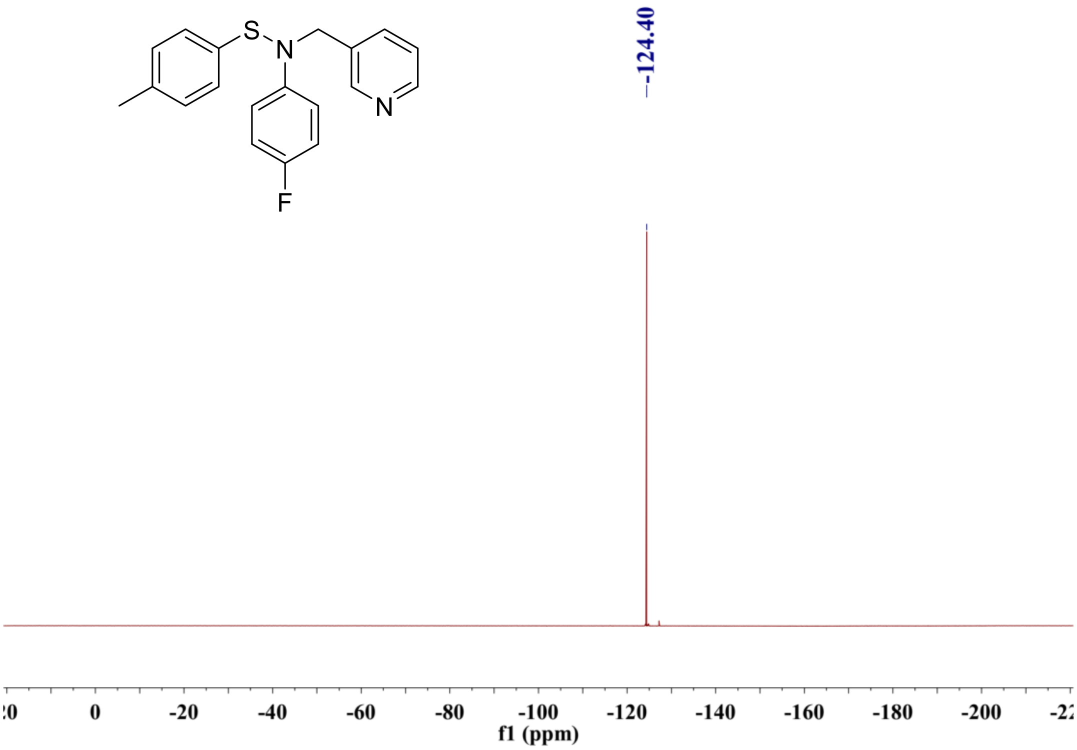

^{1}H NMR (400 MHz, CDCl$_3$) of *N*-(4-Fluorophenyl)-*N*-(thiophen-2-ylmethyl)-*S*-(*p*-tolyl)thiohydroxylamine (3add)

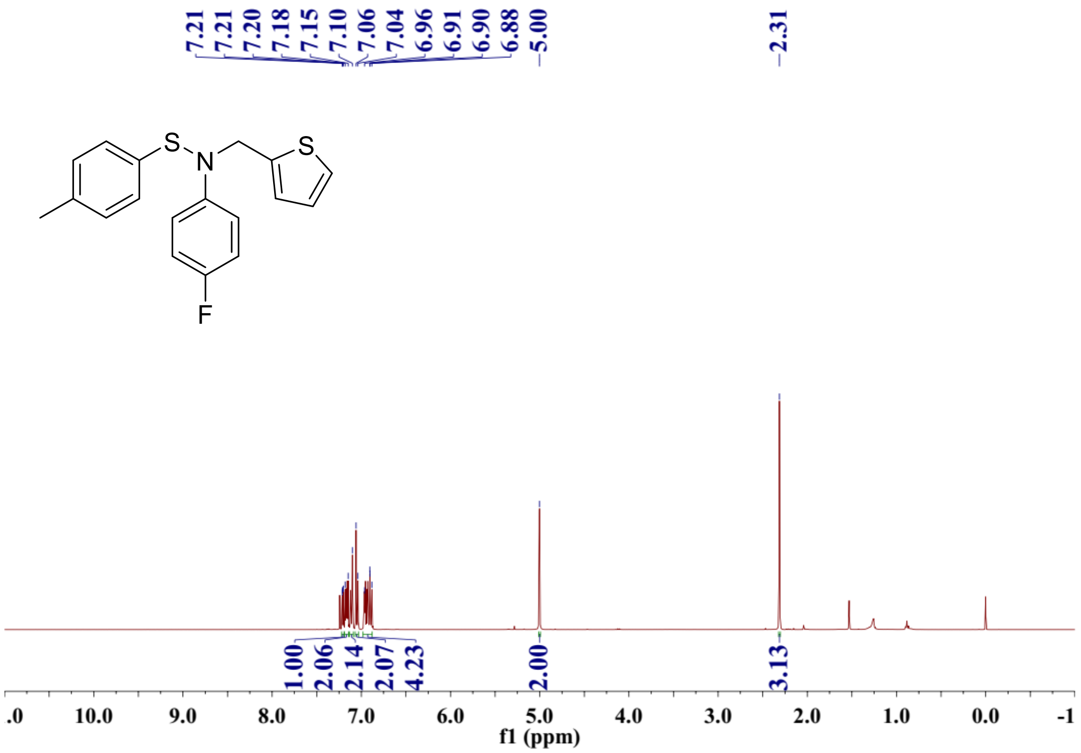

^{13}C NMR (100 MHz, CDCl$_3$) of *N*-(4-Fluorophenyl)-*N*-(thiophen-2-ylmethyl)-*S*-(*p*-tolyl)thiohydroxylamine (3add)

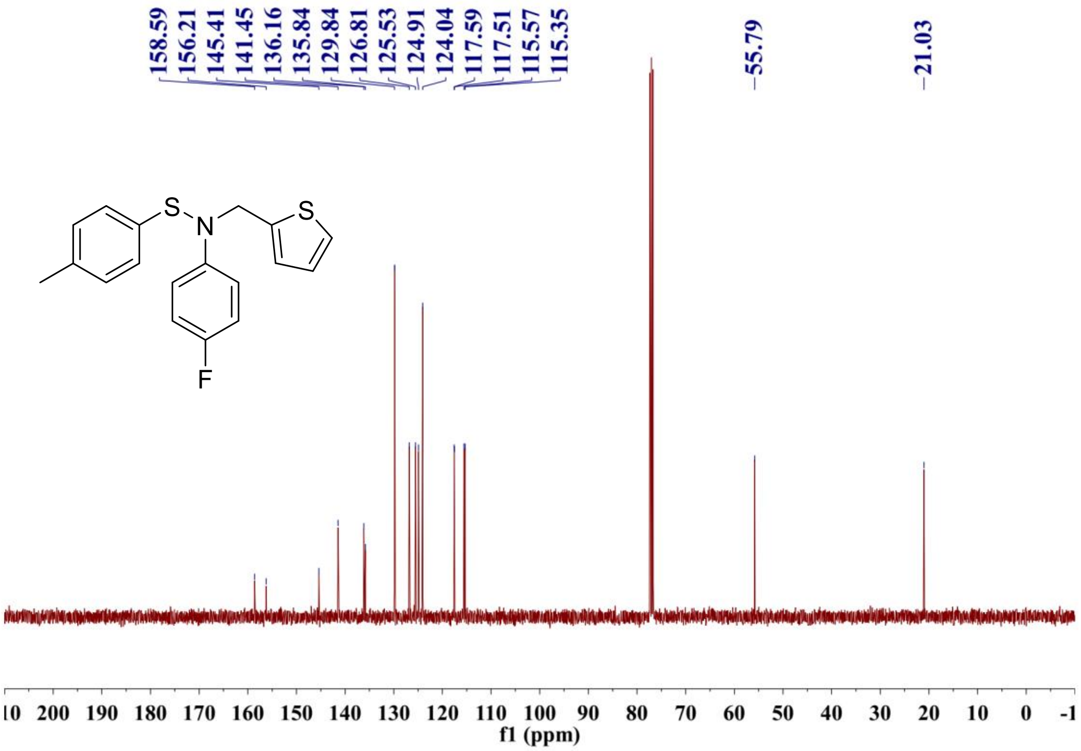

^{19}F NMR (377 MHz, CDCl$_3$) of *N*-(4-Fluorophenyl)-*N*-(thiophen-2-ylmethyl)-*S*-(*p*-tolyl)thiohydroxylamine (3add)

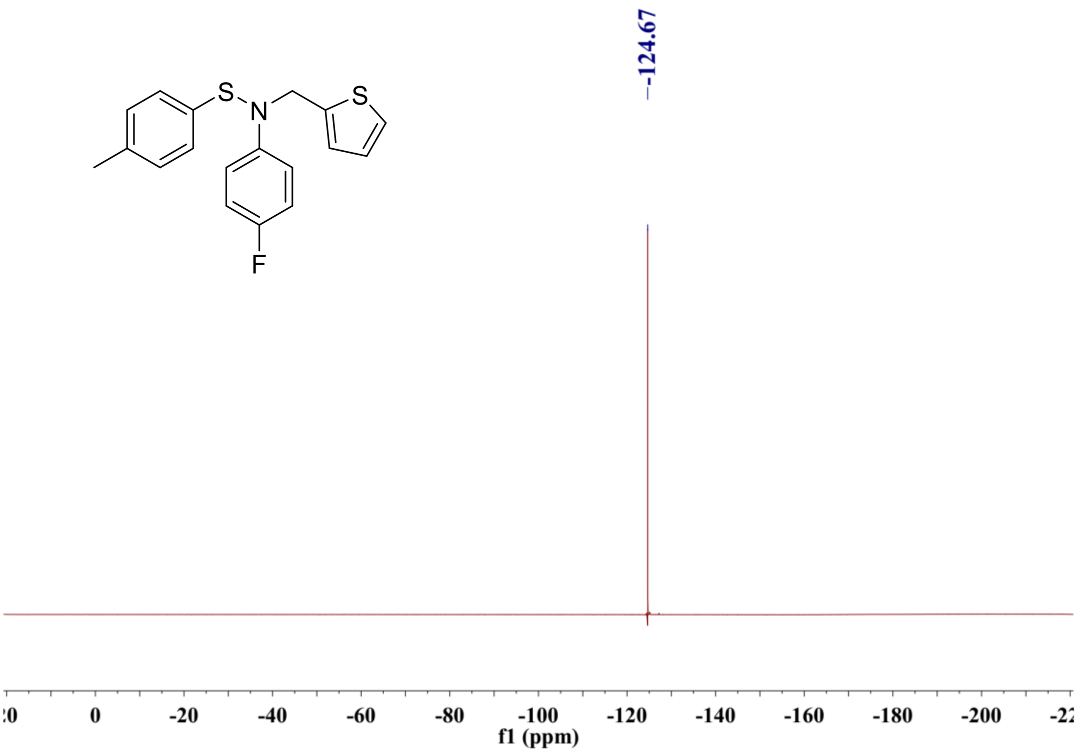

^{1}H NMR (600 MHz, $CDCl_3$) of ***N*-(4-Fluorophenyl)-*N*-(furan-2-ylmethyl)-*S*-(*p*-tolyl)thiohydroxylamine (3aed)**

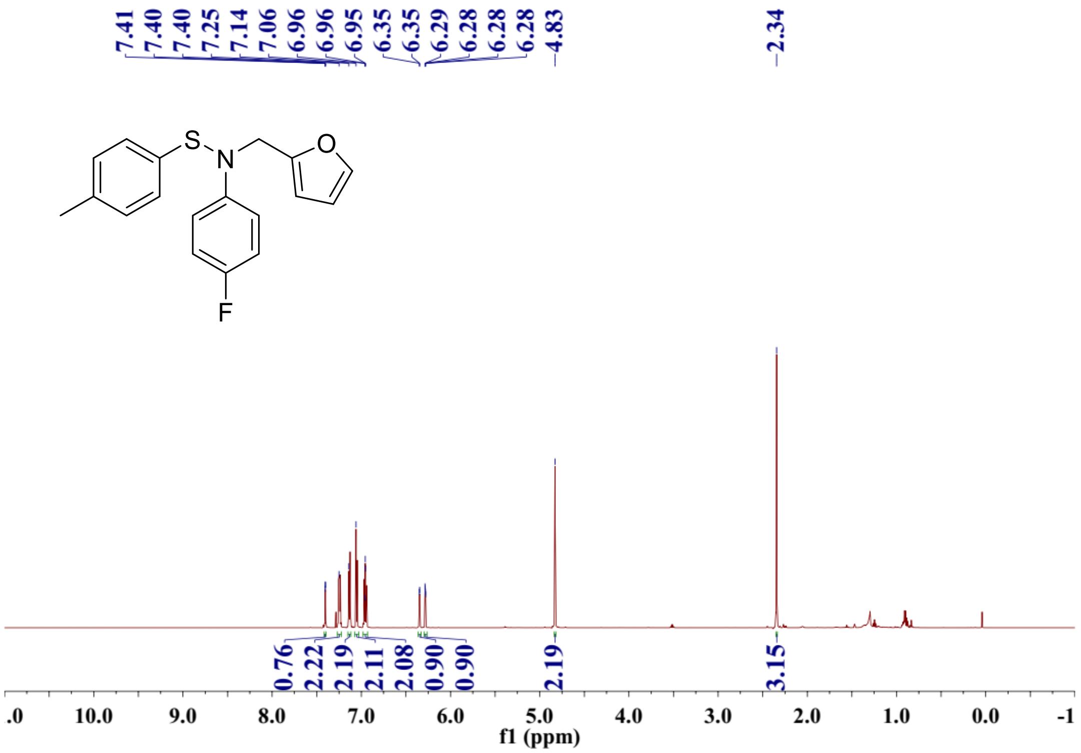

^{13}C NMR (150 MHz, $CDCl_3$) of ***N*-(4-Fluorophenyl)-*N*-(furan-2-ylmethyl)-*S*-(*p*-tolyl)thiohydroxylamine (3aed)**

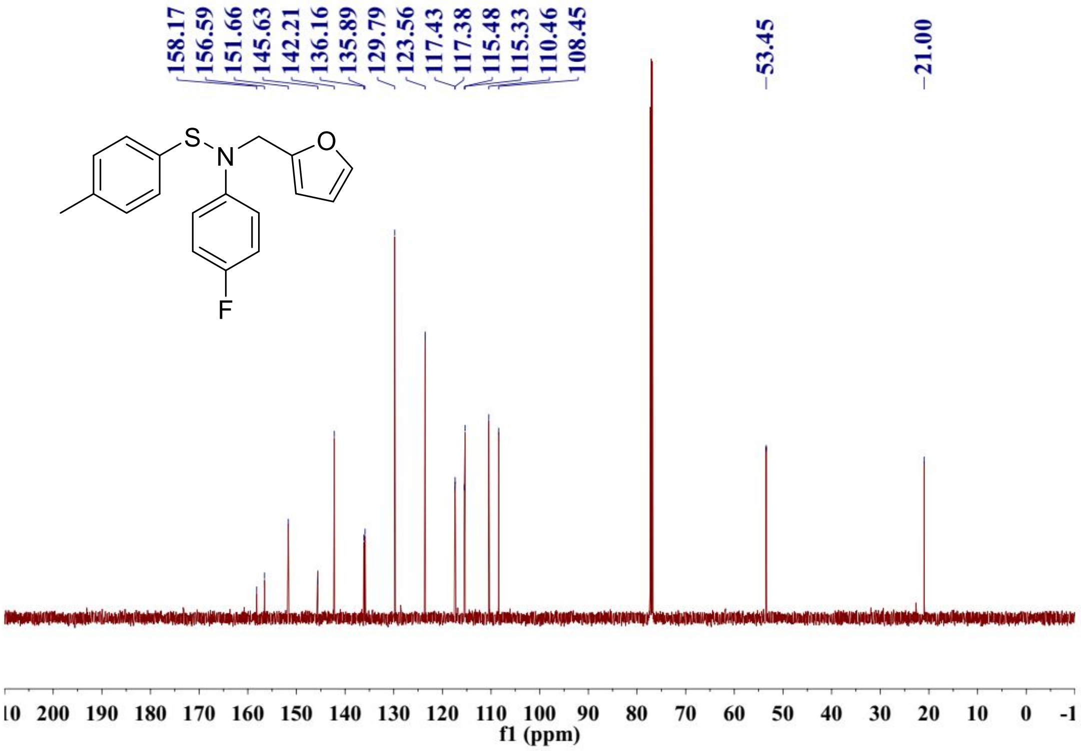

^{19}F NMR (377 MHz, $CDCl_3$) of *N*-(4-Fluorophenyl)-*N*-(furan-2-ylmethyl)-*S*-(*p*-tolyl)thiohydroxylamine (3aed)

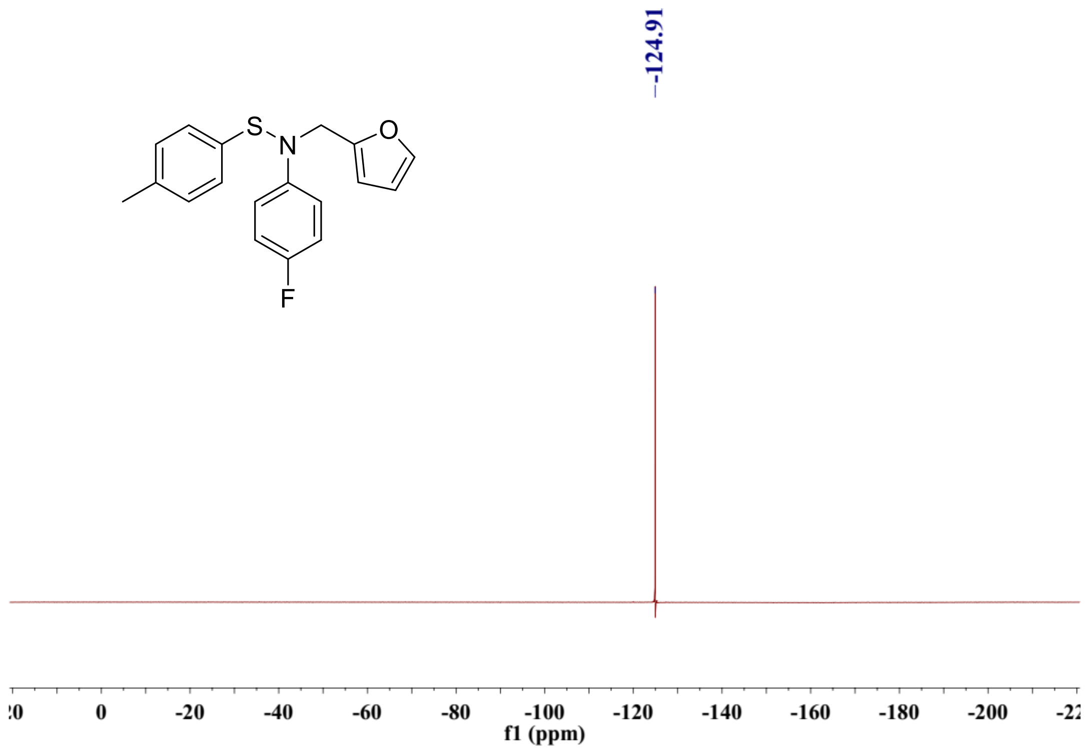

^{1}H NMR (600 MHz, DMSO-d_6) of 4-Methyl-*N*-phenyl-*N*-(*p*-tolyl)benzenesulfinamide (4)

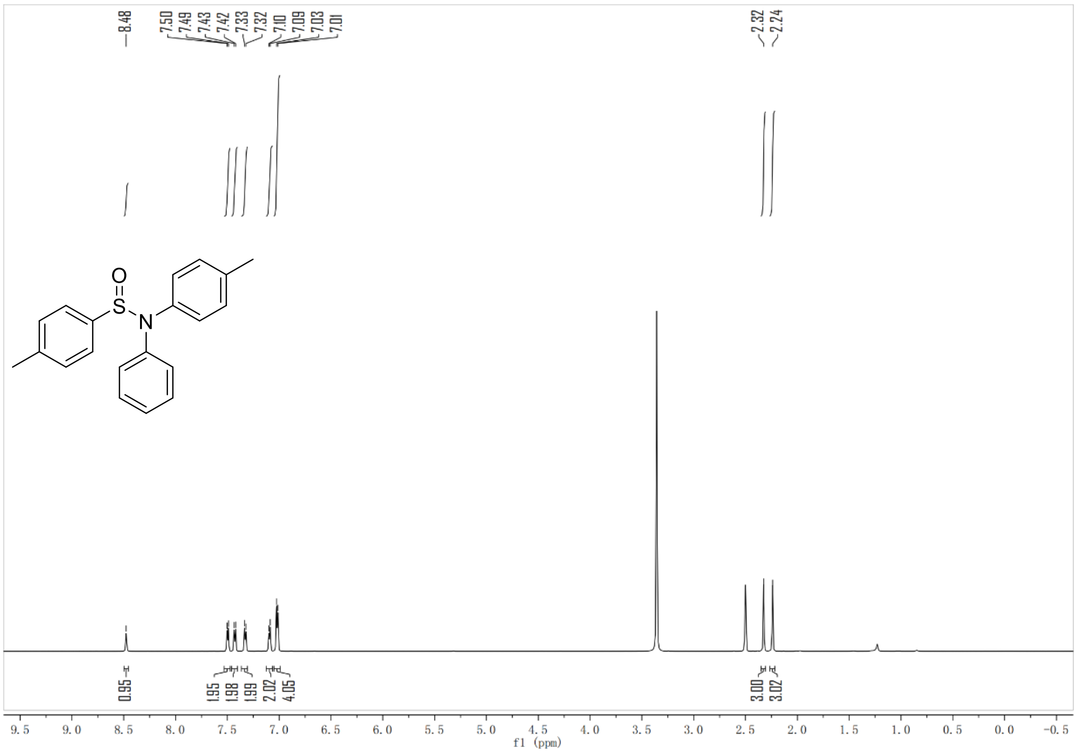

^{13}C NMR (150 MHz, DMSO-d_6) of 4-Methyl-*N*-phenyl-*N*-(*p*-tolyl)benzenesulfinamide (4)

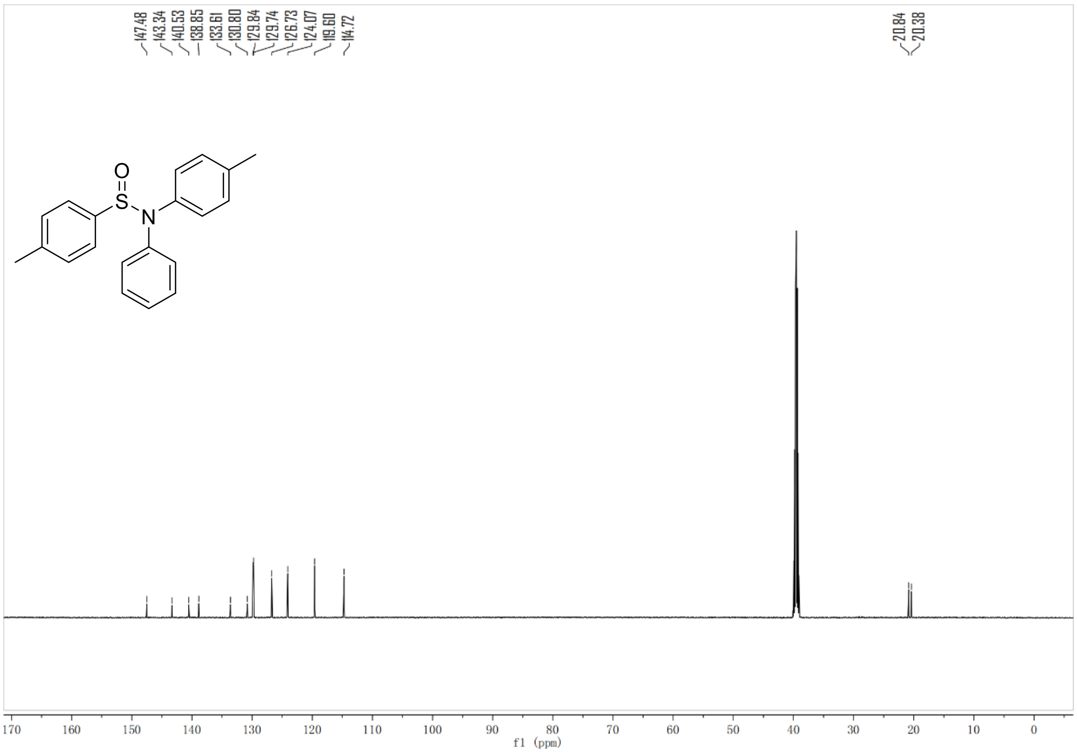

^{1}H NMR (400 MHz, $CDCl_3$) of 4-Methyl-*N*-phenyl-*N*-(*p*-tolyl)benzenesulfonamide (5)

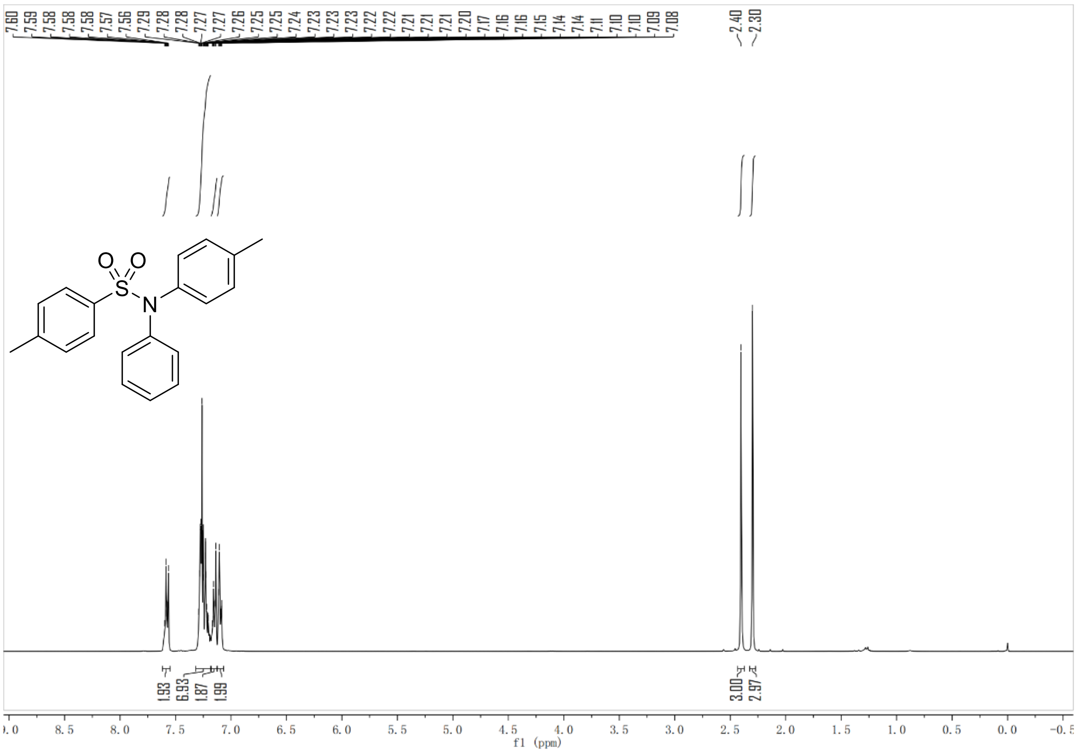

^{13}C NMR (100 MHz, $CDCl_3$) of 4-Methyl-*N*-phenyl-*N*-(*p*-tolyl)benzenesulfonamide (5)

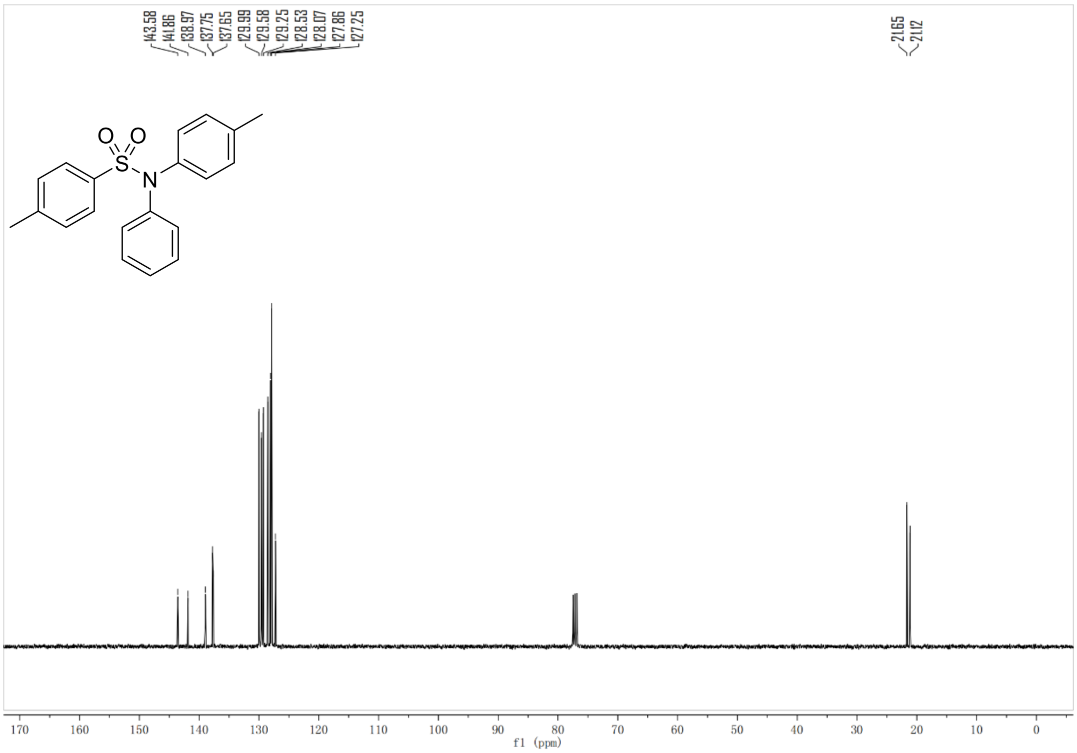

^{1}H NMR (400 MHz, DMSO-d_6) of *N*-((4-((*p*-Tolylthio)amino)phenyl)sulfonyl) acetamide (7)

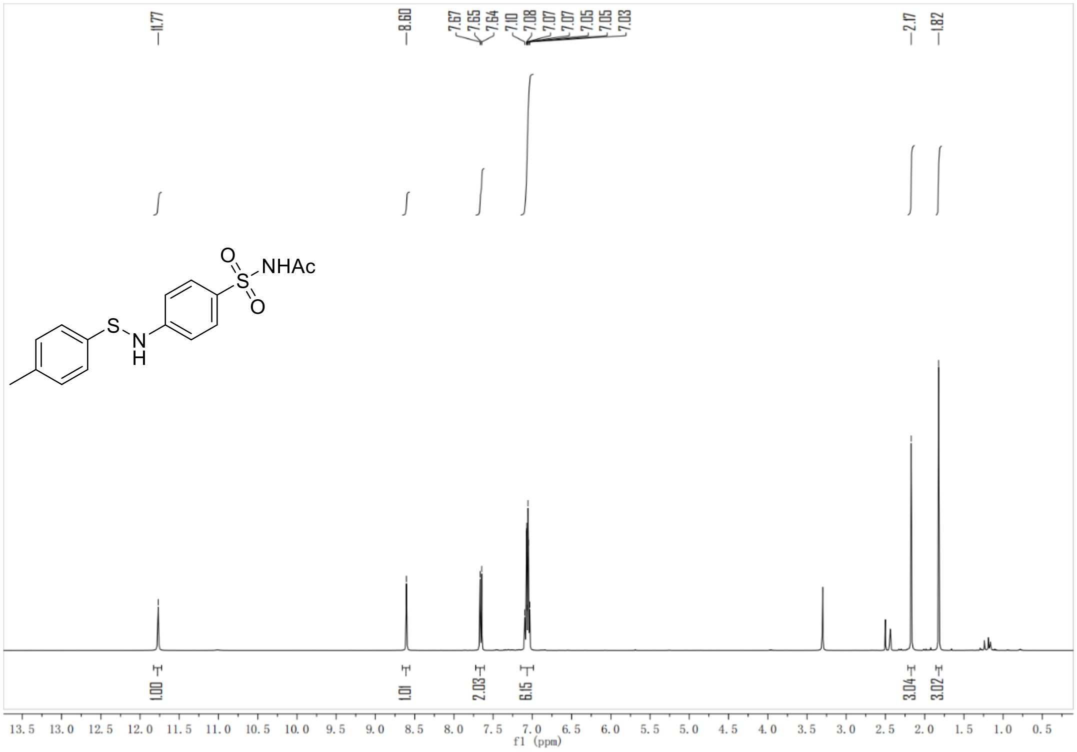

^{13}C NMR (100 MHz, DMSO-d_6) of *N*-((4-((*p*-Tolylthio)amino)phenyl)sulfonyl) acetamide (7)

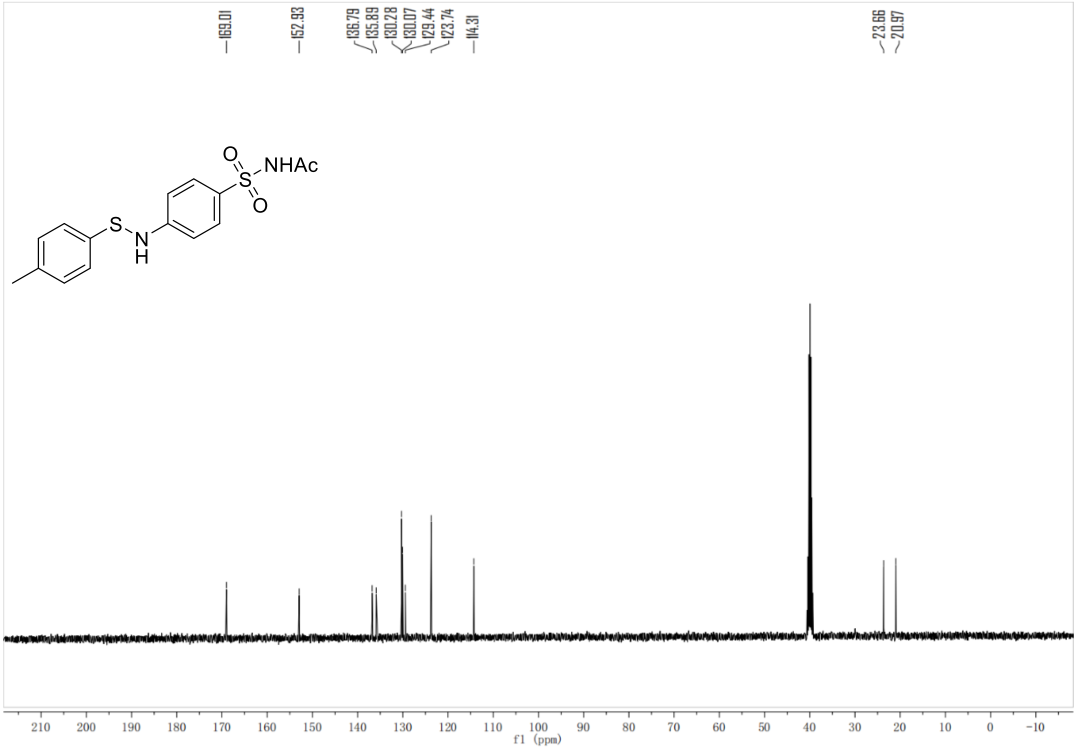

^{1}H NMR (400 MHz, DMSO-d_6) of *N*-((4-(*p*-Tolyl(*p*-tolylthio)amino)phenyl)sulfonyl)acetamide (8)

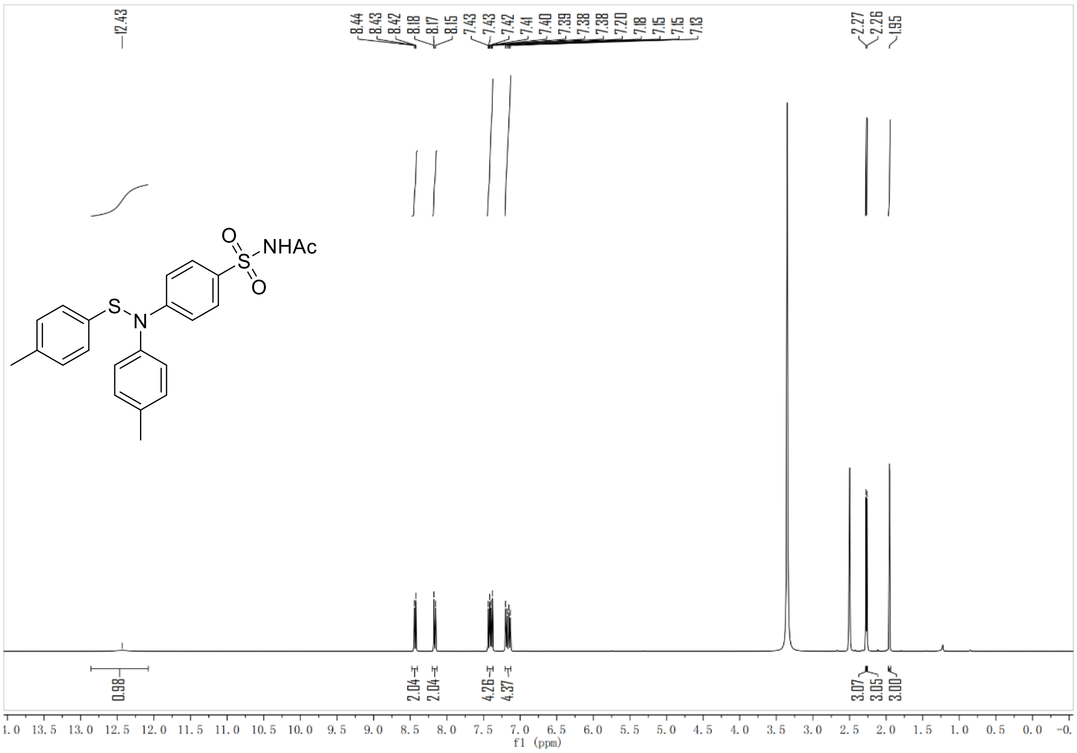

^{13}C NMR (100 MHz, DMSO-d_6) of *N*-((4-(*p*-Tolyl(*p*-tolylthio)amino)phenyl)sulfonyl)acetamide (8)

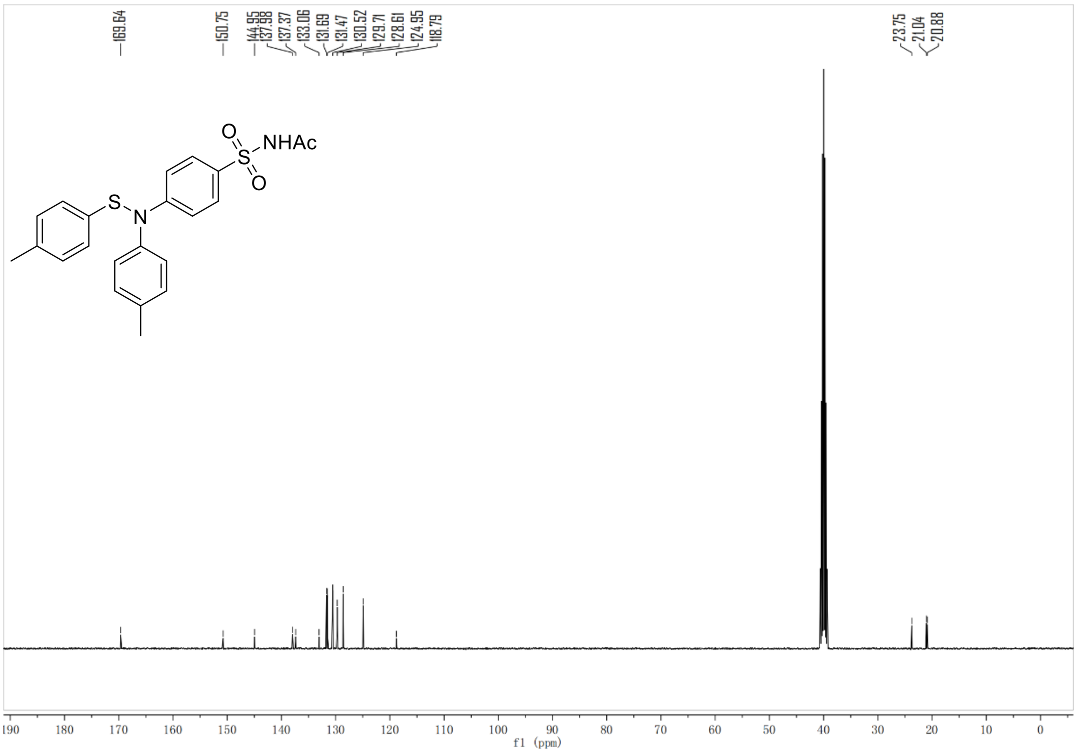

^{1}H NMR (400 MHz, $CDCl_3$) of Methyl 3-butoxy-4-nitrobenzoate (10)

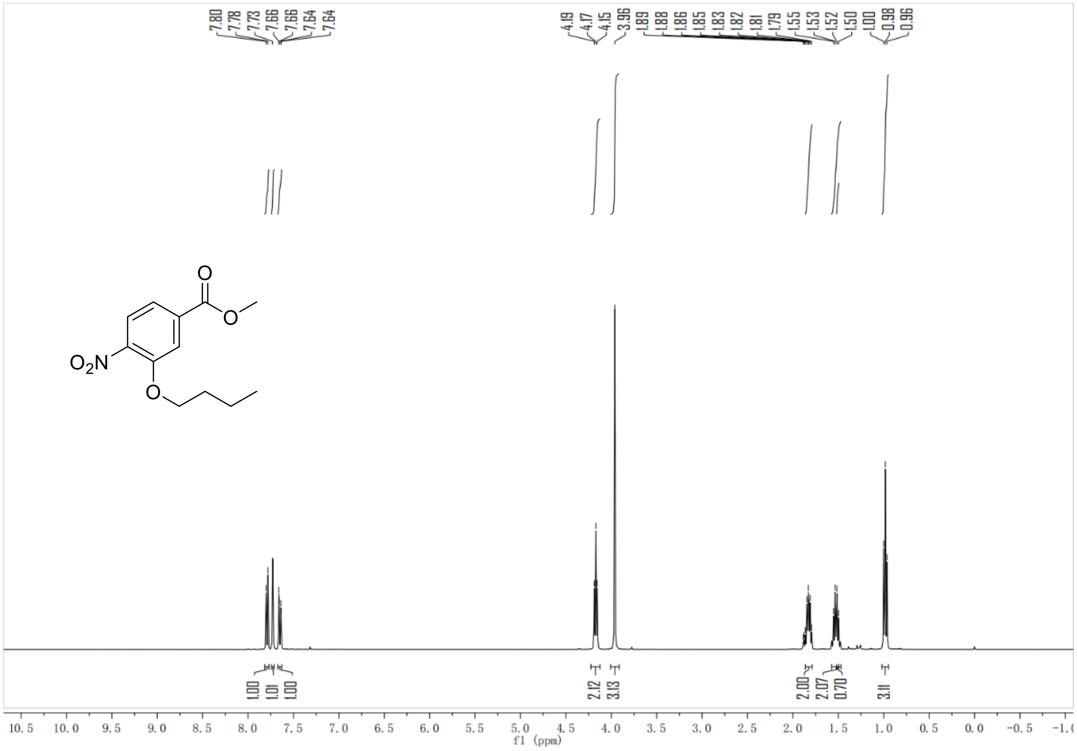

^{13}C NMR (100 MHz, $CDCl_3$) of Methyl 3-butoxy-4-nitrobenzoate (10)

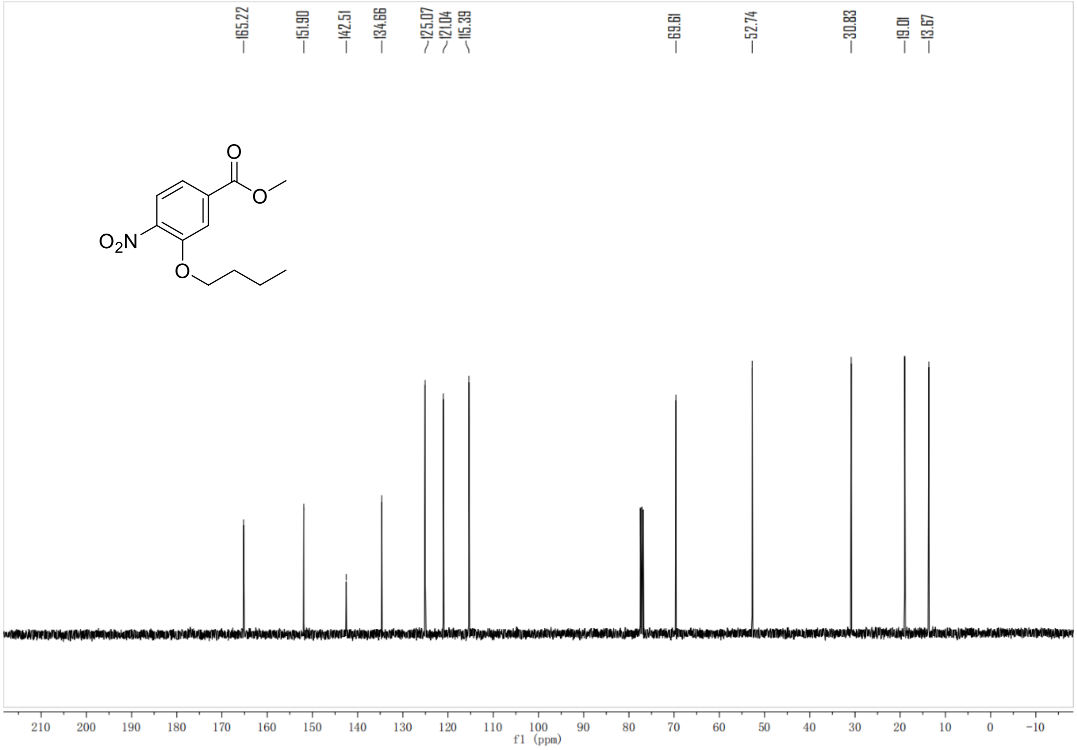

^{1}H NMR (400 MHz, MeOD) of 3-Butoxy-4-nitrobenzoic acid (11)

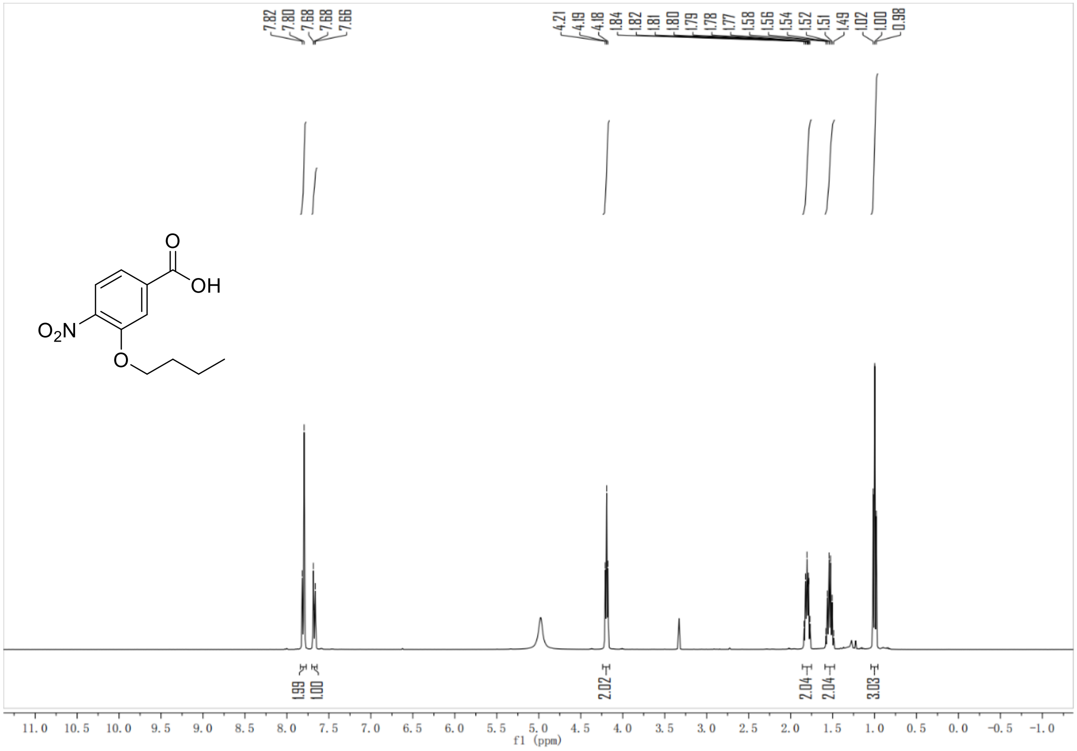

^{13}C NMR (100 MHz, MeOD) of 3-Butoxy-4-nitrobenzoic acid (11)

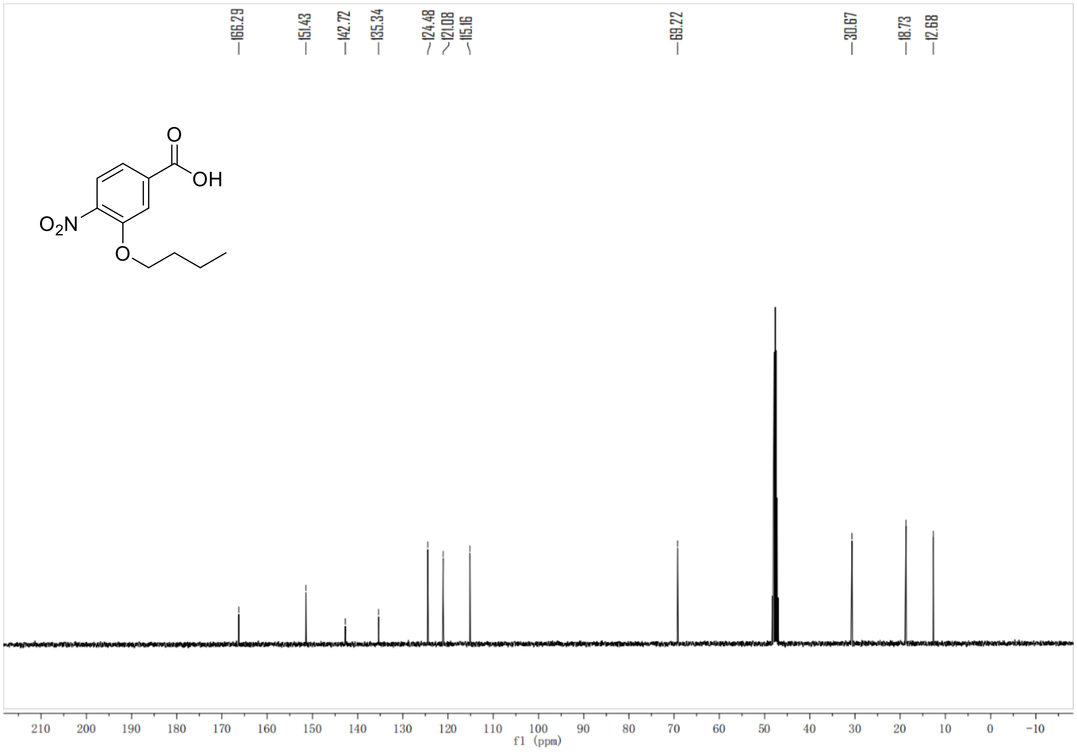

^{1}H NMR (600 MHz, $CDCl_3$) of 2-(Diethylamino)ethyl 3-butoxy-4-nitrobenzoate (12)

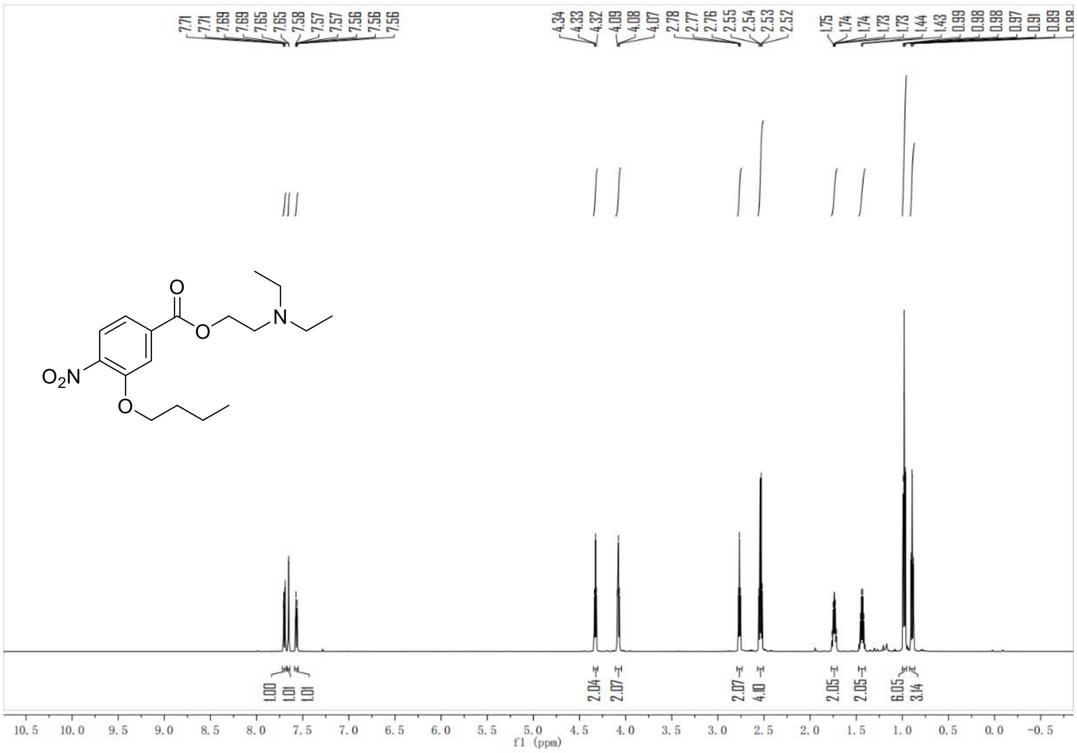

^{13}C NMR (150 MHz, $CDCl_3$) of 2-(Diethylamino)ethyl 3-butoxy-4-nitrobenzoate (12)

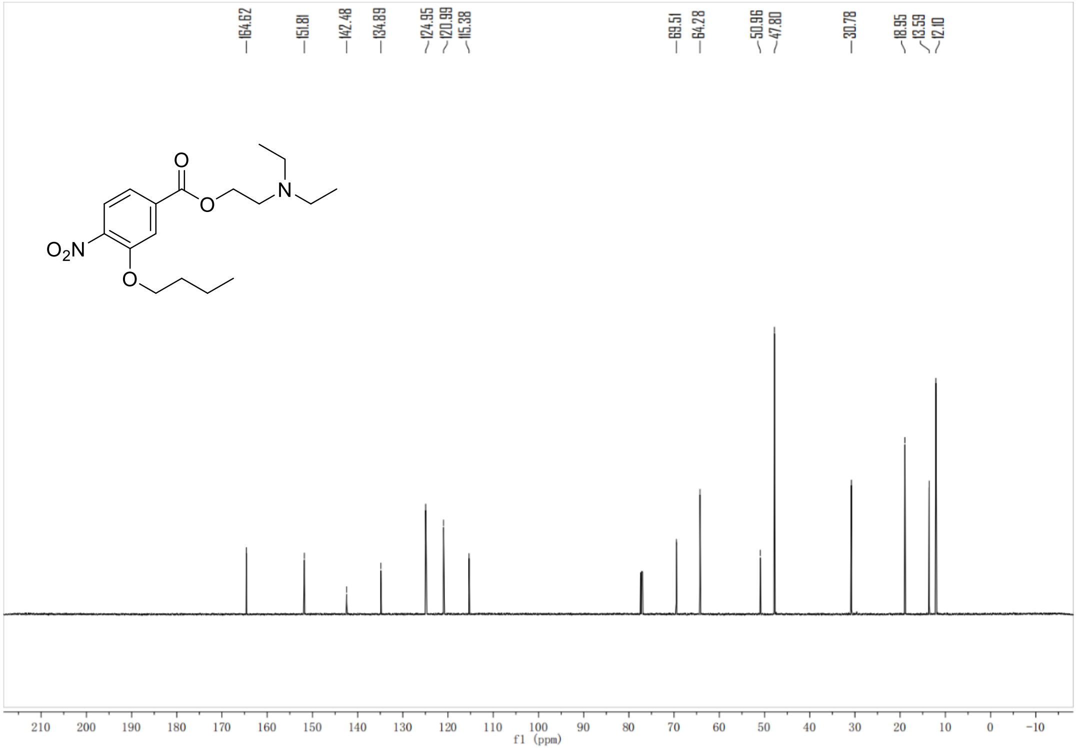

^{1}H NMR (600 MHz, $CDCl_3$) of 2-(Diethylamino)ethyl 4-amino-3-butoxybenzoate (13)

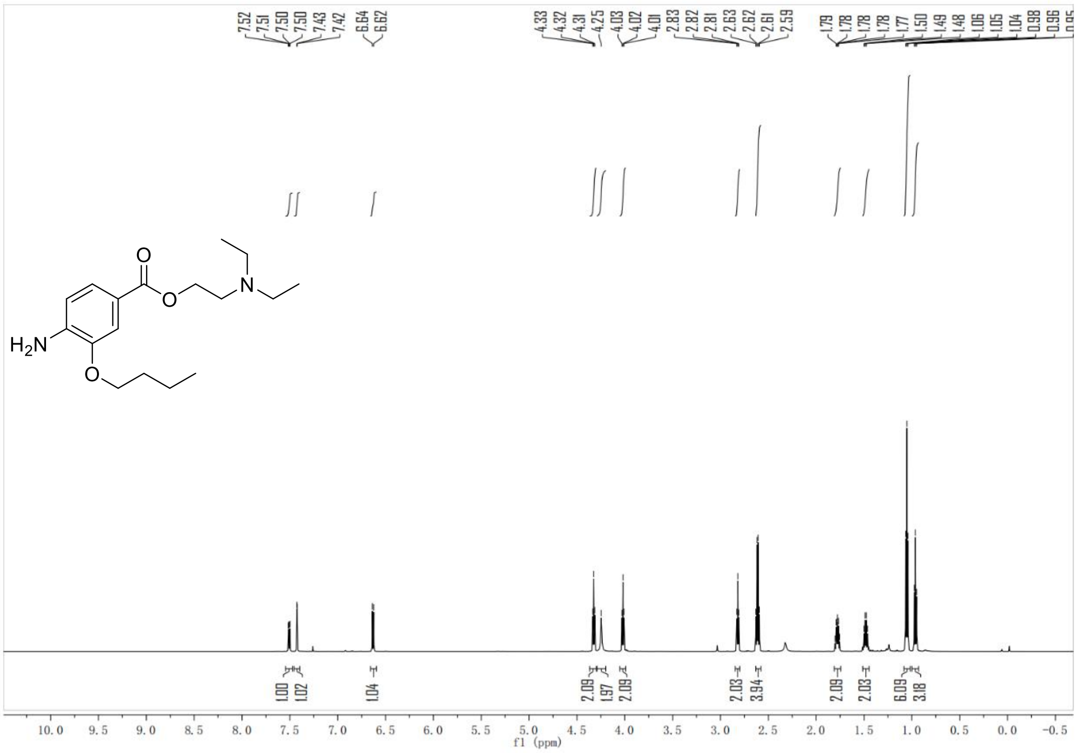

^{13}C NMR (100 MHz, $CDCl_3$) of 2-(Diethylamino)ethyl 4-amino-3-butoxybenzoate (13)

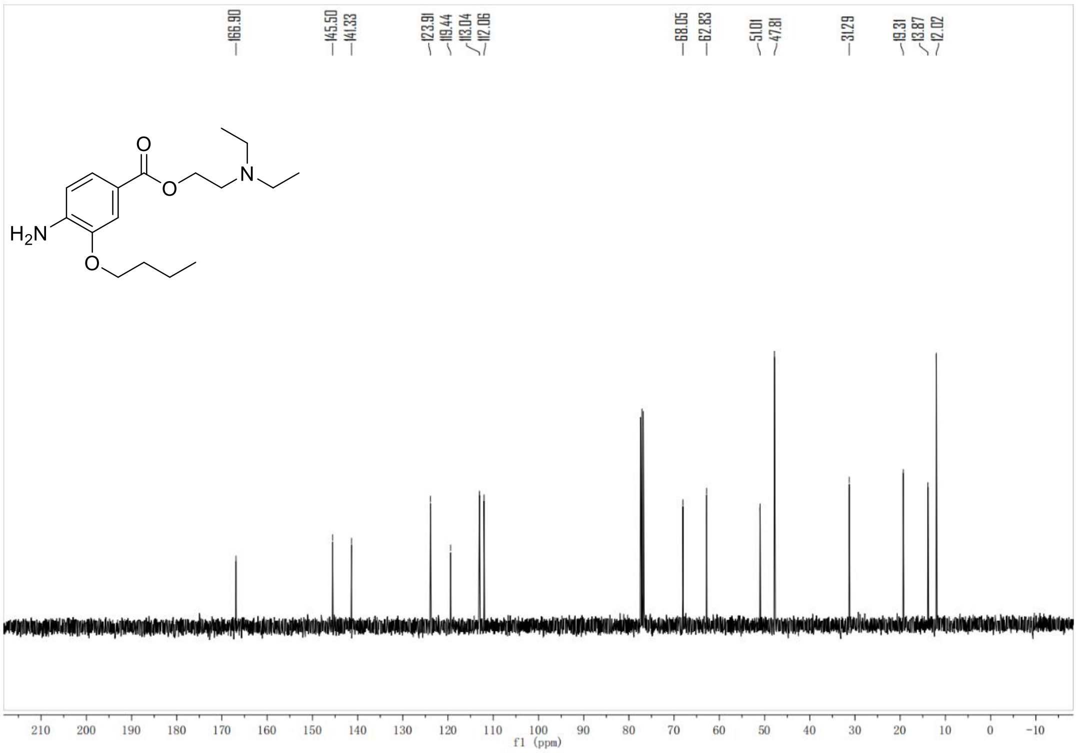

^{1}H NMR (600 MHz, $CDCl_3$) of 2-(Diethylamino)ethyl 3-butoxy-4-((p-tolylthio) amino)benzoate (14)

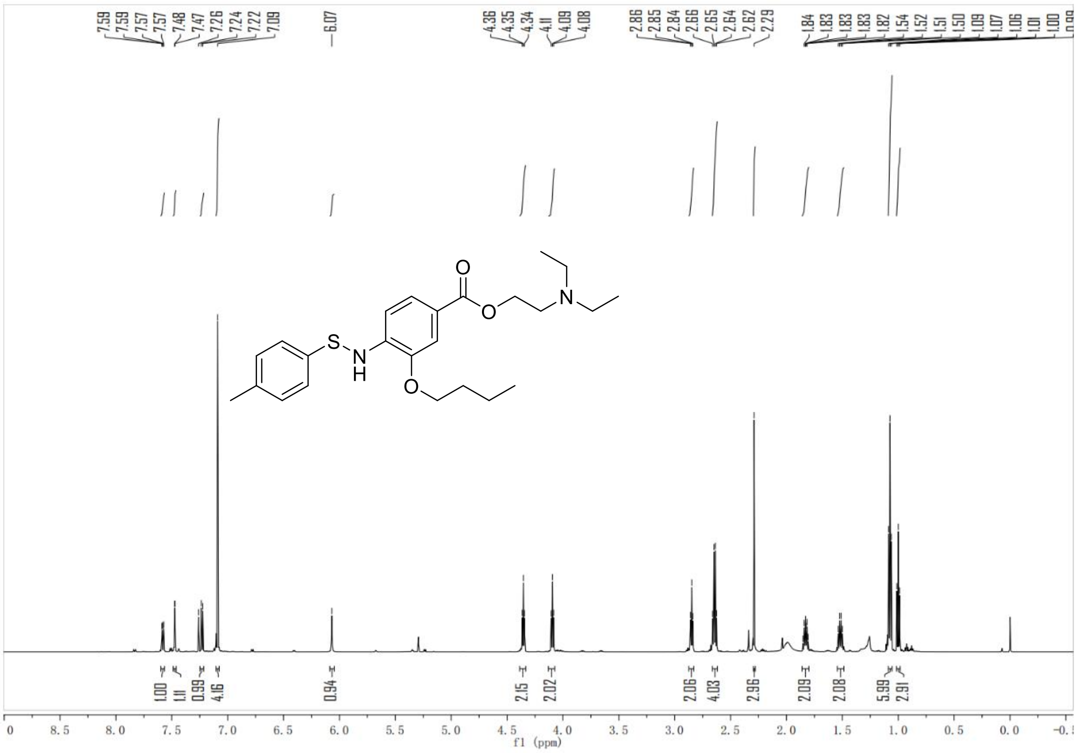

^{13}C NMR (150 MHz, $CDCl_3$) of 2-(Diethylamino)ethyl 3-butoxy-4-((p-tolylthio) amino)benzoate (14)

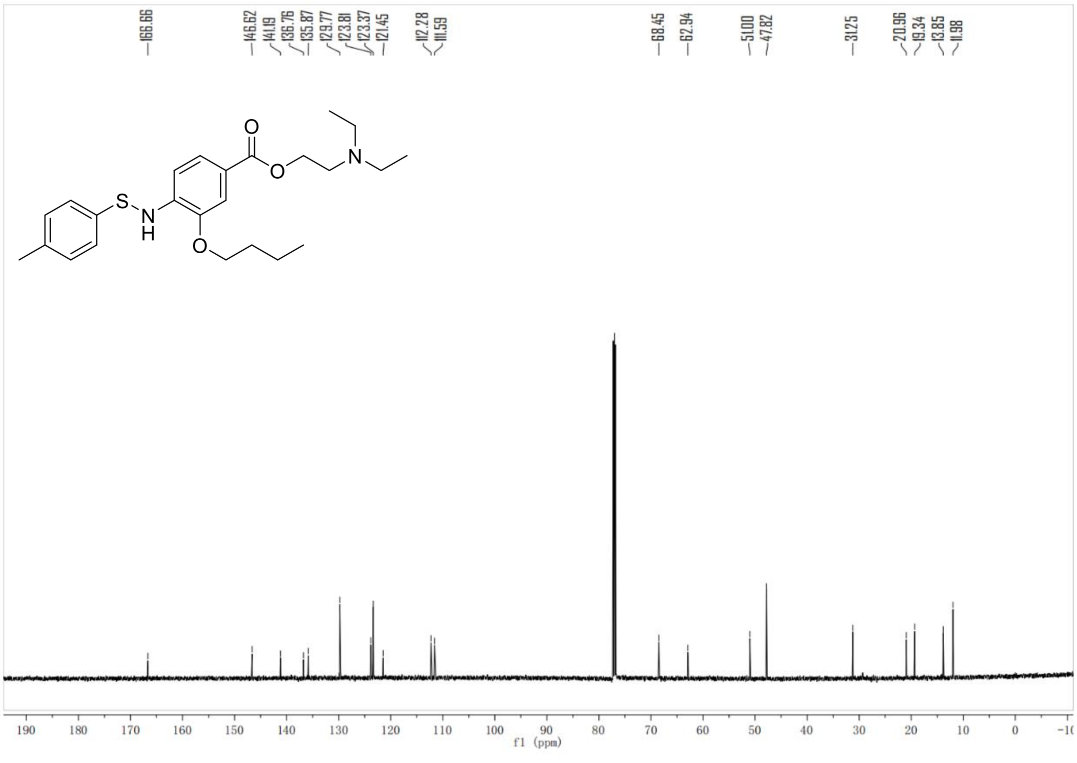

^{1}H NMR (400 MHz, $CDCl_3$) of 2-(Diethylamino)ethyl 3-butoxy-4-(*p*-tolyl(*p*-tolylthio)amino)benzoate (15)

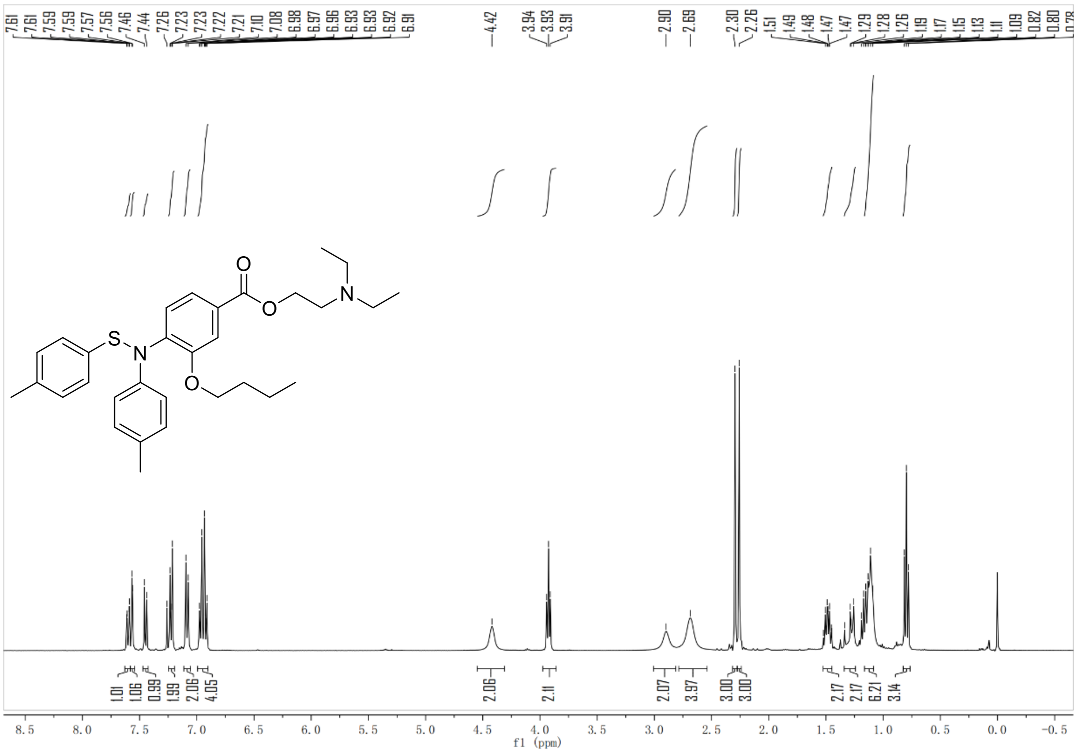

^{13}C NMR (100 MHz, $CDCl_3$) of 2-(Diethylamino)ethyl 3-butoxy-4-(*p*-tolyl(*p*-tolylthio)amino)benzoate (15)

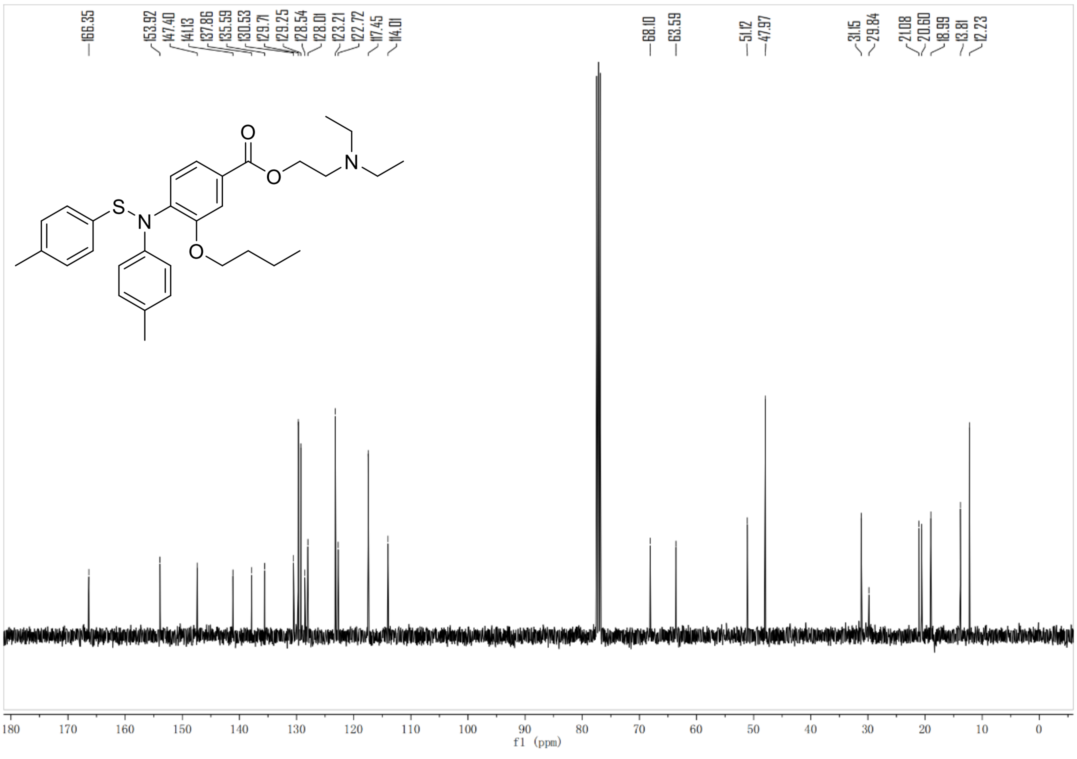

5. Supplementary References

1. Craine, L. & Raban, M. The chemistry of sulfenamides. *Chem. Rev.* **89**, 689-712 (2002).
2. Davis, F. A. et al. Chemistry of the sulfur-nitrogen bond. 12. Metal-assisted synthesis of sulfenamide derivatives from aliphatic and aromatic disulfides. *J. Org. Chem.* **42**, 967-972 (1977).
3. Taniguchi, N. Copper-catalyzed formation of sulfur–nitrogen bonds by dehydrocoupling of thiols with amines. *Eur. J. Org. Chem.* **2010**, 2670-2673 (2010).
4. Li, Y. et al. Catalytic Thia-Sommelet−Hauser rearrangement: application to the synthesis of oxindoles. *Org. Lett.* **13**, 1210–1213 (2011).
5. Dolomanov, O. V., Bourhis, L. J., Gildea, R. J., Howard, J. A. K. & Puschmann, H. OLEX2: A complete structure solution, refinement and analysis program. *J. Appl. Cryst.* **42**, 339-341 (2009).
6. Sheldrick, G. M. SHELXT – integrated space-group and crystalstructure determination. *Acta Cryst.* **A71**, 3-8 (2015).
7. Sheldrick, G. M. A short history of SHELX. *Acta Cryst.* **A64**, 112-122 (2008).
8. Geng, X. et al. Copper-catalyzed direct *N*-arylation of *N*-arylsulfonamides using diaryliodonium salts in water. *Tetrahedron Lett.* **55**, 3856-3859 (2014).
9. Stoll, S. & Schweiger, A. EasySpin, a comprehensive software package for spectral simulation and analysis in EPR. *J. Magn. Reson.* **178**, 42-55 (2006).
10. Stoll, S. & Britt, R. D. General and efficient simulation of pulse EPR spectra. *Phys. Chem. Chem. Phys.* **11**, 6614-6625 (2009).
11. Lee, C., Yang, W. & Parr, R. G. Development of the Colle-Salvetti correlation-energy formula into a functional of the electron density. *Phys. Rev. B* **37**, 785-789 (1988).
12. Becke, A. D. Density-functional thermochemistry. III. The role of exact exchange. *J. Chem. Phys.* **98**, 5648-5652 (1993).
13. Grimme, S., Antony, J., Ehrlich, S. & Krieg, H. A consistent and accurate ab initio parametrization of density functional dispersion correction (DFT-D) for the 94 elements H-Pu. *J. Chem. Phys.* **132**, 154104 (2010).
14. Petersson, G. A. & Al-Laham, M. A. A complete basis set model chemistry. II. Open-shell systems and the total energies of the first-row atoms. *J. Chem. Phys.* **94**, 6081-6090 (1991).
15. Andrae, D., Huermann, U., Dolg, M., Stoll, H. & Preu, H. Energy-adjustedab initio pseudopotentials for the second and third row transition elements. *Theoret. Chim. Acta* **77**, 123-141 (1990).
16. Zhao, Y. & Truhlar, D. G. The M06 suite of density functionals for main group thermochemistry, thermochemical kinetics, noncovalent interactions, excited states, and transition elements: two new functionals and systematic testing of four M06-class functionals and 12 other functionals. *Theor. Chem. Acc.* **120**, 215-241 (2007).
17. McLean, A. D. & Chandler, G. S. Contracted Gaussian basis sets for molecular calculations. I. Second row atoms, Z=11–18. *J. Chem. Phys.* **72**,

5639-5648 (1980).
18. Krishnan, R., Binkley, J. S., Seeger, R. & Pople, J. A. Self-consistent molecular orbital methods. 20 Basis set for correlated wave functions. *J. Chem. Phys.* **72**, 650-654 (1980).
19. Cossi, M., Rega, N., Scalmani, G. & Barone, V. Energies, structures, and electronic properties of molecules in solution with the C-PCM solvation model. *J. Comput. Chem.* **24**, 669-681 (2003).
20. CYLview20. Legault, C. Y. Université de Sherbrooke. (http://www.cylview.org) (2020).
21. Lu, T. & Chen, F. Multiwfn: a multifunctional wavefunction analyzer. *J. Comput. Chem.* **33**, 580-592 (2012).
22. Humphrey, W., Dalke, A. & Schulten, K. VMD: visual molecular dynamics. *J. Mol. Graph.* **14**, 33-38, 27-38 (1996).
